# Hydrogenative Cycloisomerization and Sigmatropic Rearrangement Reactions of Cationic Ruthenium Carbenes Formed by Catalytic Alkyne *gem*‐Hydrogenation

**DOI:** 10.1002/anie.202113827

**Published:** 2022-01-03

**Authors:** Tobias Biberger, Stephan N. Hess, Markus Leutzsch, Alois Fürstner

**Affiliations:** ^1^ Max-Planck-Institut für Kohlenforschung 45470 Mülheim/Ruhr Germany

**Keywords:** Cycloisomerizations, *gem*-Hydrogenation, NMR Spectroscopy, Rearrangements, Ruthenium Carbenes

## Abstract

*gem*‐Hydrogenation of propargyl alcohol derivatives with [Cp^X^Ru(MeCN)_3_]PF_6_ (Cp^X^=substituted cyclopentadienyl) as catalysts affords cationic pianostool ruthenium carbene complexes which are so electrophilic that they attack a tethered olefin to furnish cyclopentene products; cyclopropanation or metathesis do not compete with this novel transformation. If the transient carbenes carry appropriate propargylic substituents, however, they engage in ([2,3]‐sigmatropic) rearrangements to give enol esters (carbonates, carbamates, sulfonates) or alkenyl halides. Both pathways are unprecedented in the vast hydrogenation literature. The proposed mechanistic scenarios are in line with labeling experiments and spectroscopic data; most notably, PHIP NMR spectroscopy (PHIP=parahydrogen induced polarization) provides compelling evidence that the reactions are indeed triggered by highly unorthodox gem‐hydrogenation events.

## Graphical abstract

Two unprecedented hydrogenation reactions are disclosed, commencing with geminal delivery of both H‐atoms of H_2_ to the triple bond of a propargyl alcohol derivative. When catalyzed with cationic ruthenium complexes, the resulting pianostool carbenes are so “hot” that they are readily intercepted by tethered nucleophiles, resulting in cycloisomerization or [2,3]‐sigmatropic rearrangement reactions.

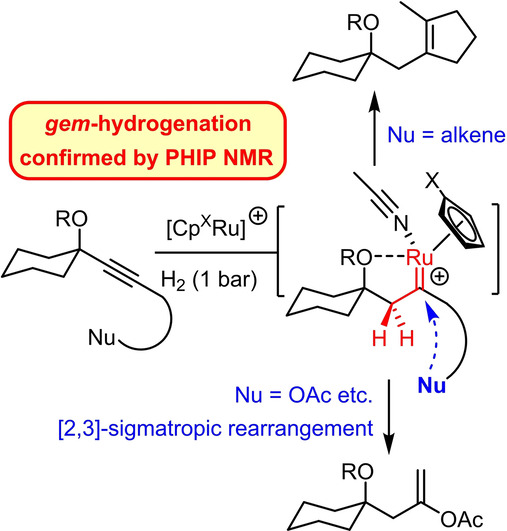

After more than a century of intense research into all aspects of catalytic hydrogenation in academic and industrial laboratories,[^1^, ^2^, ^3^, ^4^, ^5^, ^6^, ^7^] our group managed to find a fundamentally new reactivity mode. *gem*‐Hydrogenation is distinguished by the delivery of both H‐atoms of H_2_ to the same C‐atom of an alkyne with formation of a methylene group; the adjacent position is concomitantly transformed into a discrete metal carbene entity.^[8]^ The original discovery was made using [Cp*RuCl(cod)], [Cp*RuCl]_4_ or [Cp*Ru(MeCN)_3_]PF_6_ as catalyst, which afford electrophilic pianostool ruthenium half‐sandwich complexes of the Fischer‐carbene type.[^9^, ^10^] A second system employing [(NHC)(η^6^‐cymene)MCl_2_] (M=Ru, Os; NHC=N‐heterocyclic carbene) is photochemically driven; it opens an unconventional hydrogenative entry into “second generation” Grubbs carbenes for use in olefin metathesis.[^11^, ^12^]

For the likely involvement of excited states, the mechanism of this photochemical *gem*‐hydrogenation reaction is intricate and by no means fully understood. In contrast, combined experimental, spectroscopic and computational studies allow a fairly detailed picture to be drawn of the way how the [Cp*Ru]‐based catalysts exert their function (Scheme 1).[^8^, ^10^] The ruthenium atom serves as a carbophilic Lewis acid that renders the bound alkyne in a loaded complex of type **A** ^[13]^ sufficiently activated for a first hydrogen transfer from H_2_ initially ligated as σ‐complex.[^10^, ^14^] Either end of the resulting metallacyclopropene **B** is then capable of accepting the second H‐atom: whereas delivery to the C_α_‐atom ultimately affords the corresponding *trans*‐alkene (**B**→**E**→**F**),^[14]^ transfer to C_β_ completes the actual *gem*‐hydrogenation event (**B**→**C**). For a regular internal alkyne, these processes have similar barriers and therefore usually run in parallel;^[10]^ substituents on the triple bond or at the propargylic position, however, can provide sufficient bias for *gem*‐hydrogenation to become the predominant or even exclusive pathway.

**Scheme 1:**
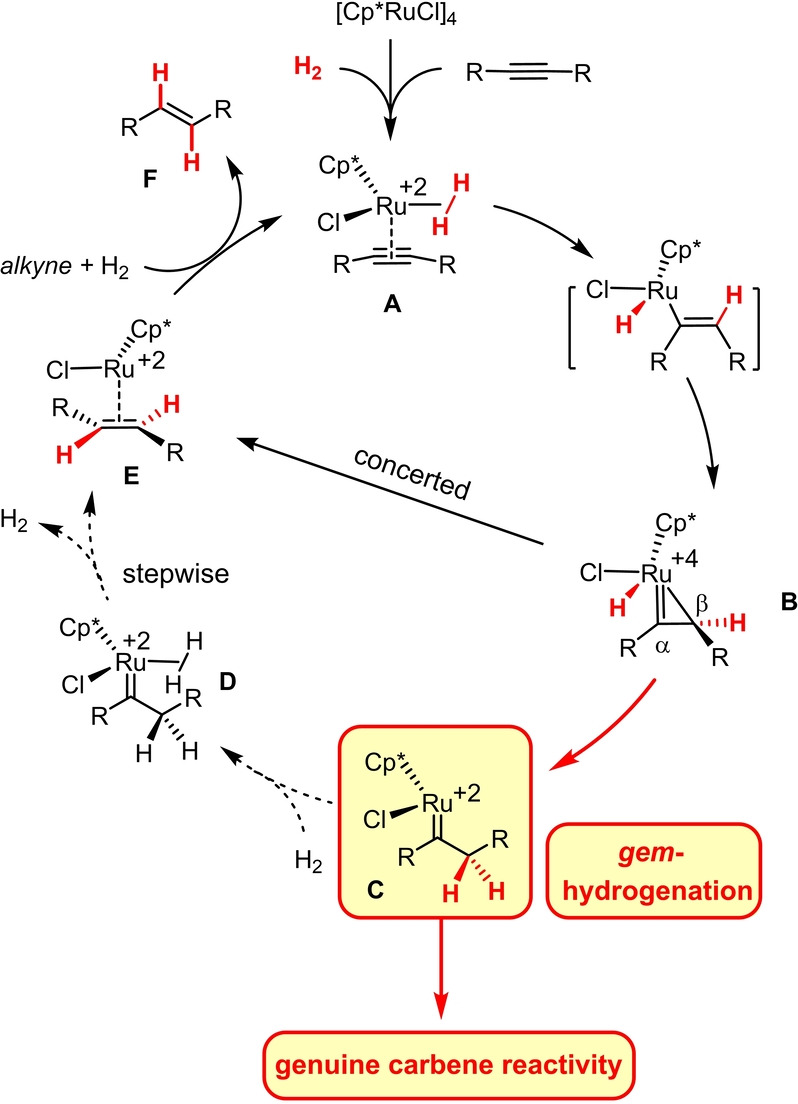
Mechanism of the innately intertwined *trans*‐hydrogenation and *gem*‐hydrogenation of internal alkynes; the dotted lines indicate those steps that need to be blocked or outperformed downstream of *gem*‐hydrogenation in order to harness genuine carbene reactivity; Cp*=pentamethylcyclopentadienyl.

The ability to generate pianostool ruthenium carbenes **C** in situ by catalytic hydrogenation is enabling; it allowed us to discover entirely new transformations, including hydrogenative cyclopropanation, hydrogenative metathesis, various hydrogenative heterocycle syntheses, and hydrogenative C−H insertion.[^10^, ^15^, ^16^, ^17^, ^18^] Except for a few cases, these reactions were effected with [Cp*RuCl]_4_ as the catalyst,^[19]^ which is commercial and easy to use. Yet, NMR data clearly showed that other (substituted) cyclopentadienyl rings, which are less electron‐rich than Cp*, favor *gem*‐hydrogenation over the competing *trans*‐hydrogenation to a larger extent and hence likely provide additional opportunities.[^20^, ^21^] As outlined below, this is indeed the case.

Reactions of metal carbenes with olefins other than cyclopropanation or metathesis are exceedingly rare.[^22^, ^23^] While *gem*‐hydrogenation of enyne **1 a** with complex [Cp*RuCl]_4_ (**C1**) afforded the expected cyclopropane **3** in high yield,[^15^, ^17^] switch to the *cationic* precatalyst [Cp*Ru(MeCN)_3_]PF_6_ (**C2**) led to a surprisingly different result in that the cyclopentene derivative **4 a** was formed as the major product (**3**:**4 a**=18:82, NMR). The outcome could be further optimized by recourse to the even less electron rich complexes [CpRu(MeCN)_3_]PF_6_ (**C3**)^[24]^ or [Cp^T^Ru(MeCN)_3_]PF_6_ (**C4**),^[25]^ in which the donor ability of the cyclopentadienyl ligand is reduced by conjugation to an ester substituent; their use allows cyclopropanation to be completely suppressed and product **4 a** to be isolated in analytically pure form in 93 % yield. The additional examples shown in Scheme 2 illustrate the scope.

**Scheme 2:**
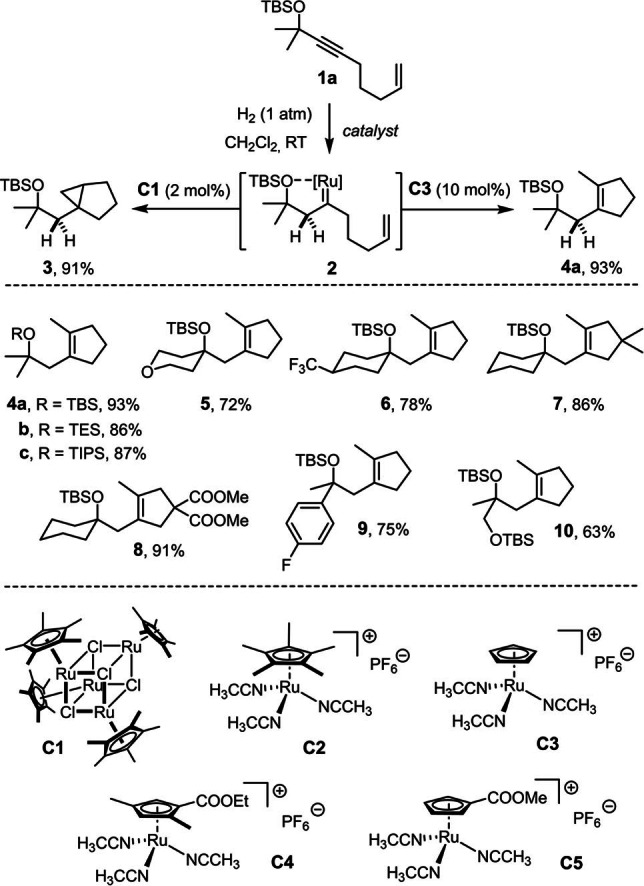
Hydrogenative cyclopropanation versus hydrogenative cycloisomerization (**C3** (10 mol %), CH_2_Cl_2_, RT).

Since we are unaware of any precedent, several control experiments were carried out to gain insights into the mechanism of this catalytic carbocycle synthesis (Scheme 3). First, it was shown that cyclopentene **4 a** is not a secondary product derived from cyclopropane **3** by downstream ring‐opening: no evolution was observed and the compound recovered unchanged in 96 % yield after 18 h reaction time. As the molecular formula of **4 a** suggests, cyclopentene formation proceeds only in the presence H_2_; under Ar atmosphere, enyne **1 a** was slowly converted into a complex mixture, from which only cycloheptadiene **11** could be isolated and characterized. It is reasonable to assume that this compound is the result of π‐acid catalysis,^[26]^ in which coordination of the [CpRu]^+^ fragment to the triple bond engenders an outer‐sphere attack by the tethered alkene moiety. The resulting homoallyl cation **F** is one resonance extreme of a multi‐faceted non‐classical carbocation/carbene intermediate that stabilizes itself, inter alia, in form of product **11**.[^27^, ^28^] Additional control experiments showed that the silyl ether in **1 a** also plays an important role, in that the analogous methyl ether derivative **1 d** gave the cyclohexene derivative **12** as the major product admixed with cyclopentene **4 d**.^[29]^ Particularly relevant is the outcome of the reaction of [D]‐**1 b** labeled at the internal position of the olefin, which furnished product [D]‐**4 b**, in which the label has migrated by one positon to the C‐atom derived from the olefin terminus; within the error bar of ^1^H NMR (ca. ±2 %), no deuterium is lost during this 1,2‐shift.

**Scheme 3:**
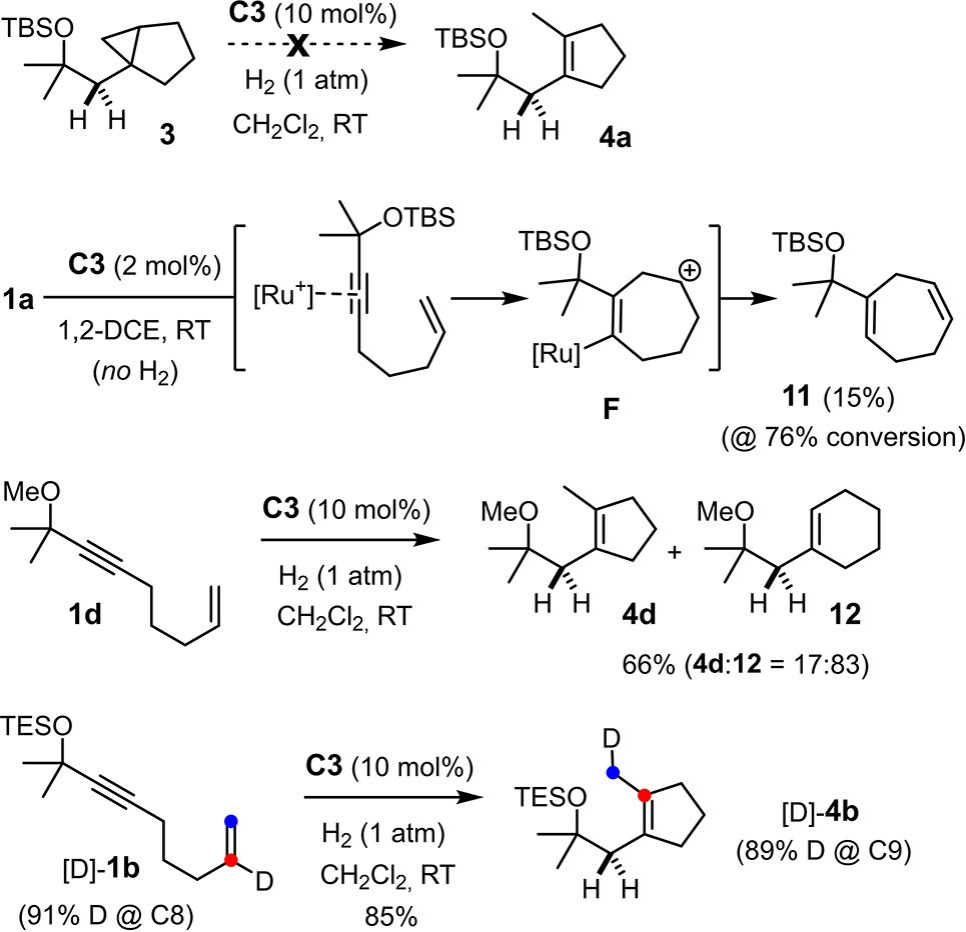
Control experiments.

All attempts to characterize the putative intermediate **2** derived from **1** (or related enynes) by spectroscopic means were to no avail. Therefore, indirect evidence was sought to support the notion that the reaction is triggered by *gem*‐hydrogenation. To this end, compound **13 a** devoid of the tethered olefin serving as the trap for the emerging cationic carbene was hydrogenated using parahydrogen (*p*H_2_) and the reaction monitored by PHIP NMR spectroscopy (PHIP=parahydrogen induced polarization),[^31^, ^32^, ^33^] which is known to be exquisitely sensitive and has proven highly informative during our previous investigations.[^9^, ^10^, ^20^] As expected, the characteristic hyperpolarized signals of a methylene group formed by regioselective *gem*‐hydrogenation were detected when the reaction was performed with either catalytic [CpRu(MeCN)_3_]PF_6_ (**C3**, see the Supporting Information) or the ester‐bearing complex **C5** ^[34]^ (Scheme 4). The distinctive “down‐up” pattern of the antiphase signals proves the methylene character of the site formed in the actual hydrogenation event. For the underlying physics,[^31^, ^32^, ^33^] this spectroscopic fingerprint implies that both hydrogen atoms derive from the same molecule of H_2_ and must have been transferred in an (essentially) concerted step. Moreover, the large ^2^ *J* coupling constant shows that the two H‐atoms are diastereotopic and the adjacent carbene center of **14 a** hence chiral‐at‐metal. This situation is best explained by assuming a donor/acceptor interaction between the silyl ether and the ruthenium center that locks the reactive intermediate in form of a cyclic array and prevents free rotation about the newly formed single bond from occurring.^[35]^ Additional information was gained when the PHIP spectra were “cleared” by application of the OPSY pulse sequence (OPSY=only parahydrogen spectroscopy).^[30]^ A notably better signal‐to‐noise ratio was observed with **C5** as catalyst, which suggests that the ester substituent on the ancillary cyclopentadienyl ring renders the corresponding cationic pianostool carbene intermediate **14 a** somewhat more stable and hence longer‐lived on the NMR timescale than that derived from the parent complex [CpRu(MeCN)_3_]PF_6_ (**C3**) (see the Supporting Information), perhaps as a result of a stronger O⋅⋅⋅Ru interaction.^[36]^

**Scheme 4:**
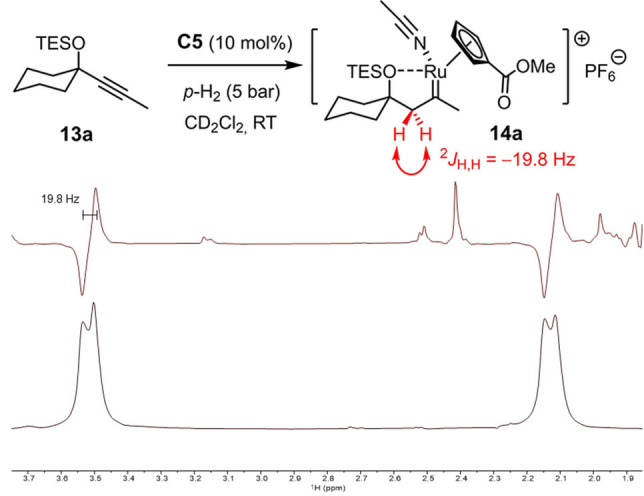
Use of *p*H_2_ proves the formation of a chiral‐at‐metal ruthenium carbene complex by alkyne *gem*‐hydrogenation under catalytic conditions; the excerpts show the hyperpolarized signals in the methylene region (1.9‐3.7 ppm) after excitation with a π/4 pulse (top) and after using an OPSY filter (bottom),^[30]^ which are attributed to complex **14 a** formed by *gem*‐hydrogenation.

Based on these experimental data, we propose that the hydrogenative cyclopentene synthesis commences with formation of a ruthenium carbene intermediate **G** that is highly electrophilic by virtue of the positive charge and the fairly poor electron donor ligands that it carries (Cp or Cp^COOMe^ instead of Cp*; R_3_SiO‐ instead of MeO‐ as the steering substituent) (Scheme 5).^[20]^ Previous computations at the CCSD(T)/def2‐TZVPP level of theory showed that pianostool ruthenium carbene complexes invariably react with tethered olefins to give “kite‐shaped” metallacycles of type **H** in the first place, in which all three C‐atoms entertain bonding interactions with the metal;^[17]^ in the present case, formation of such an intermediate comes along with delocalization of the positive charge.^[37]^ As this intermediate evolves and a Ru−C2 bond is forming, the H‐atom is forced to migrate to the adjacent terminal position, as proven by the labeling experiment. This 1,2‐H shift can formally be rationalized by considering carbocation **H′**, which, however, must not be mistaken for a discrete intermediate but only represents a resonance extreme of the Lewis structure.^[38]^ Decoordination of the metal fragment from **I** releases the product and closes the catalytic cycle. The overall outcome likely reflects the kinetic preference for the formation of a five‐membered ring by 5‐*exo*‐*trig* cyclization, which has also been observed in related transformations.^[39]^

**Scheme 5:**
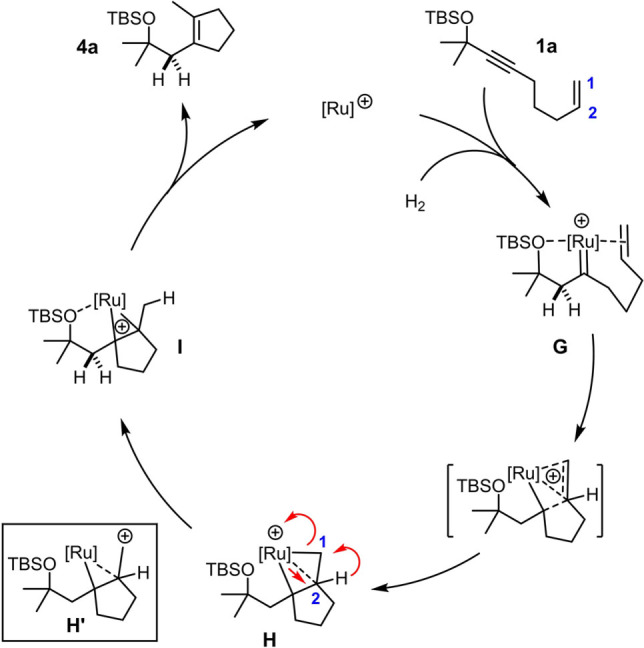
Tentative mechanism of the hydrogenative cycloisomerization (arbitrary numbering).

Additional evidence for the intervention of an exceptionally electrophilic intermediate comes from some of the limitations encountered when exploring the scope of the reaction. It is pointed out that all successful examples compiled in Scheme 2 comprise a *carbocyclic* ring formed by *gem*‐hydrogenation/cycloisomerization; actually, placement of an oxygen or nitrogen linker in between the alkyne and alkene site in a substrate of type **1** resulted in decomposition. Though tentative, it is reasonable to assume that the presence of such innately nucleophilic sites adjacent to the emerging electrophilic carbene might explain the failure. Conversely, one could perceive opportunities to trap the putative reactive intermediate by properly placed substituents in a productive manner. In line with this notion, hydrogenation of the propargylic acetate derivative **15 a**, preferentially with the aid of **C5** as catalyst, cleanly furnished the corresponding enol acetate **17 a** as the result of a [2,3]‐sigmatropic rearrangement of the transient cationic ruthenium carbene intermediate **16 a** (Scheme 6).[^40^, ^41^, ^42^] The reaction scaled well (89 %, 1.47 g of product) and proved more flexible with regard to the steering substituent (ether, silyl ether, acetal) than the hydrogenative cycloisomerization outlined above (cf. **17 a**–**d**). Moreover, groups other than carboxylic acid esters are equally prone to hydrogenative rearrangement, including carbonates (**20 a**, **b**), carbamates (**21**), and a weakly nucleophilic sulfonate (**22**). Even a propargylic bromide could be engaged in an analogous 1,2‐shift without competing hydrogenolytic cleavage, as illustrated by the formation of alkenyl bromide **23**, although the corresponding chloride (unreactive) and iodide (decomposition) proved inadequate. In line with earlier results on hydrogenative heterocycle syntheses,^[16]^ a substrate bearing a malonate terminus readily cycloisomerized to the trisubstituted furan derivative **25** by attack of the ester carbonyl group onto the electrophilic carbene species. Attempted transpositions of a propargylic thioether, silane, or phosphonate, however, basically met with failure.

**Scheme 6:**
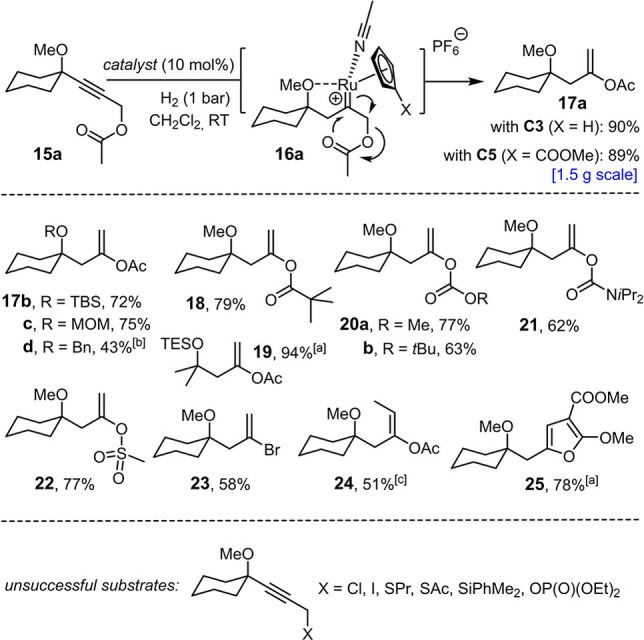
Hydrogenative rearrangements; unless stated otherwise, all reactions were performed with **C5** as the catalyst; ^[a]^ with **C3** as the catalyst; ^[b]^ with 20 mol % of **C3**; ^[c]^ yield of the pure *E*‐isomer after flash chromatography; *E:Z*=7:3 (crude product).

Once again, PHIP spectra provide compelling evidence for *gem*‐hydrogenation as the decisive trigger and hence help to rule other conceivable scenarios out.^[43]^ Figure 1 shows the relevant methylene region of the OPSY spectra recorded upon hydrogenation of a set of substrates with **C5** as the catalyst: in each case, the characteristic PHIP‐enhanced AB‐pattern of the CH_2_‐group of a reactive intermediate of type **16** was detected, which derives from geminal delivery of both H‐atoms of H_2_ to the same C‐atom of the corresponding alkyne substrate **15**. This observation also implies that *gem*‐hydrogenation must be remarkably facile and that the turnover‐limiting step of the catalytic transformation lies downstream of carbene formation.

**Figure 1:**
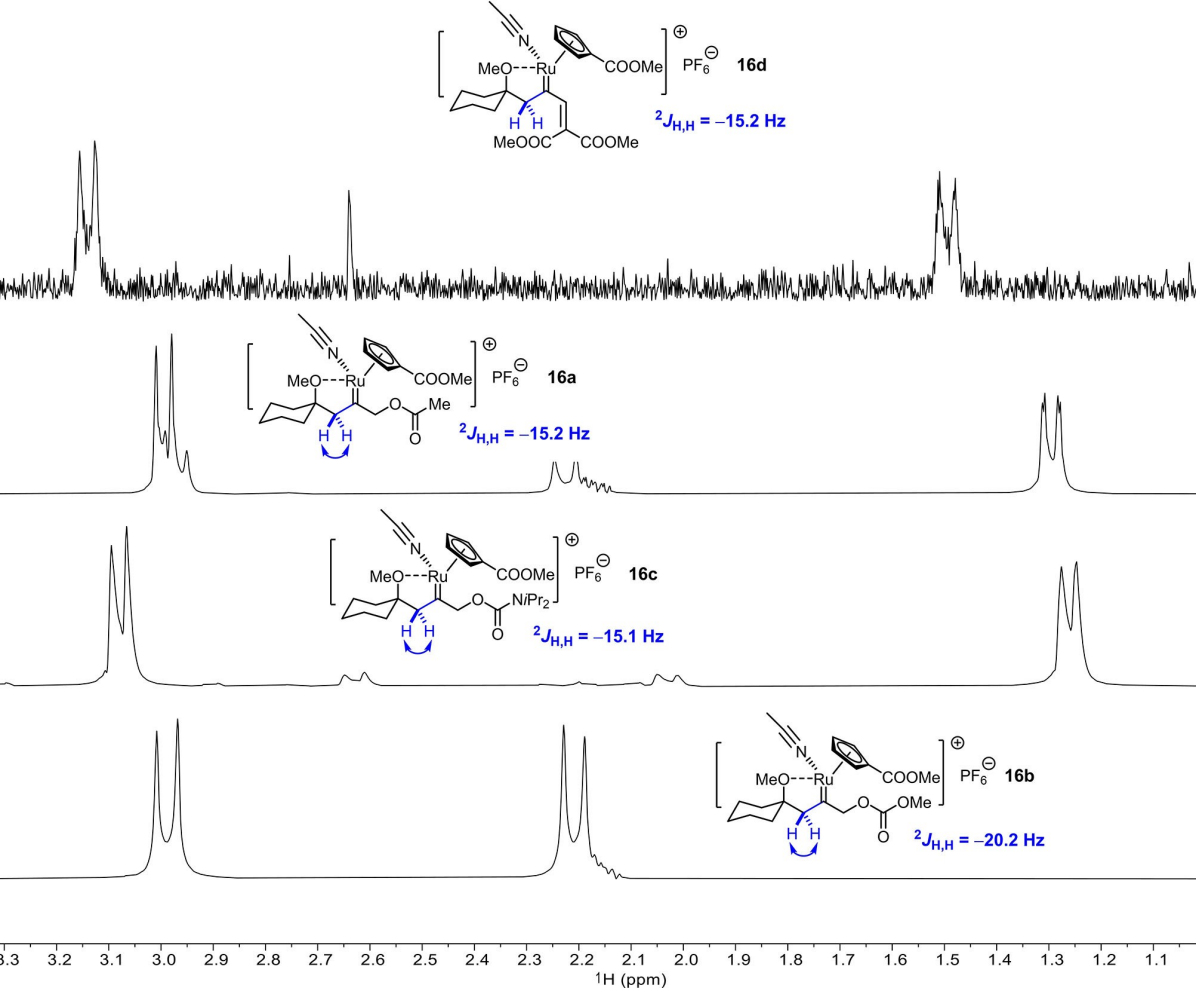
Excerpt of the OPSY NMR spectra of reaction mixtures derived from substrates of type **15**, which invariably show that the catalytic formation of pianostool ruthenium carbenes by *gem*‐hydrogenation precedes [2,3]‐sigmatropic rearrangement or cycloisomerization.

In summary, two unprecedented hydrogenation reactions are described, which rely on the ability of [Cp^X^Ru]‐complexes to catalyze geminal transfer of H_2_ to a triple bond with formation of a discrete ruthenium pianostool carbene flanked by a methylene group. Upon deliberate upregulation of the electrophilicity by proper choice of the ancillary Cp^X^ ligand on a cationic ruthenium fragment, these reactive intermediates do not engage a tethered alkene in cyclopropanation or metathesis any longer, as previously described by our group using [Cp*RuCl]_4_ as the catalyst[^15^, ^17^] but lead to cycloisomerization. Alternatively, polar substituents at the propargylic position are able to migrate onto the carbene site to give valuable enol ester or alkenyl halide derivatives. These new transformations increase the portfolio of *gem*‐hydrogenation and encourage further investigations into this perplexing yet arguably enabling mode of hydrogen transfer and orthogonal gateway to metal carbene chemistry.

## Conflict of interest

The authors declare no conflict of interest.

## Supporting information

As a service to our authors and readers, this journal provides supporting information supplied by the authors. Such materials are peer reviewed and may be re‐organized for online delivery, but are not copy‐edited or typeset. Technical support issues arising from supporting information (other than missing files) should be addressed to the authors.

Supporting Information
