# Supplementary material for: Hydrogenative Cycloisomerization and Sigmatropic Rearrangement Reactions of Cationic Ruthenium Carbenes Formed by Catalytic Alkyne gem‐Hydrogenation
Source: Angew Chem Int Ed Engl. 2022 Jan 3;61(8):e202113827. doi: 10.1002/anie.202113827 (PMC9306539; doi:10.1002/anie.202113827)
Supplement: Supplementary file 1 — Supporting Information [file ANIE-61-0-s001.pdf]

Angewandte Chemie
Eine Zeitschrift der Gesellschaft Deutscher Chemiker

Supporting Information

Hydrogenative Cycloisomerization and Sigmatropic Rearrangement Reactions of Cationic Ruthenium Carbenes Formed by Catalytic Alkyne *gem*-Hydrogenation

*T. Biberger, S. N. Hess, M. Leutzsch, A. Fürstner**

General

Unless stated otherwise, all reactions were carried out under argon atmosphere in flame-dried Schlenk glassware. The solvents were purified by distillation over the indicated drying agents under argon: THF, Et_2O (Mg/anthracene), hexanes (Na/K); EtOH, MeOH (Mg); 1,2-dichloroethane (1,2-DCE), CH_2Cl_2, EtOAc, tetrahydropyran (CaH_2); 2-butanone (B_2O_3). DMF, MeCN and Et_3N were dried by an absorption solvent purification system based on molecular sieves. 1,2-Dichloroethane, CD_2Cl_2, tetrahydropyran and 2-butanone were degassed by freeze-pump-thaw (3 x) and stored over molecular sieves (except 2-butanone).

Flash chromatography was performed with silica gel 60 (0.015 – 0.04 mm) purchased from Macherey-Nagel or with silica gel cyano 60 (0.035-0.07 mm, 400-220 mesh) purchased from Carl Roth.

NMR spectra were recorded on Bruker DPX 300, AMX 300, AV 400 or AV III 600 spectrometers in the solvents indicated; chemical shifts (δ) are given in ppm relative to TMS, coupling constants (*J*) in Hz. The solvent signals were used as references and the chemical shifts converted to the TMS scale ($CDCl_3$: δ_C = 77.16 ppm; residual $CHCl_3$: δ_H = 7.26 ppm; CD_2Cl_2: δ_C = 54.00 ppm; residual $CHDCl_2$: δ_H = 5.32 ppm; C_6D_6: δ_C = 128.06 ppm; residual C_6HD_5 : δ_H = 7.16 ppm). Proton and carbon assignments were established using HSQC, HMBC and NOESY experiments.

OPSY (Only ParaHydrogen SpectroscopY)[1] spectra were acquired on a Bruker Advance III 500 MHz spectrometer equipped with a BBFO probe using the following parameters: number of scans: 16, spectral width: 20 kHz (40 ppm), fid size: 32768 data points, relaxation delay: 0 s. The PHIP NMR data was processed with MNova 11.0.4 (Mestrelab Research Santiago de Compostela, Spain). After all the dissolved hydrogen had been fully consumed, samples were usually reshaken and reinserted and the acquisition restarted. Depending on the substrate, samples can be reshaken until all the hydrogen in the gas phase or all the starting material is fully consumed. Generally, hyperpolarized species were observed until the reactants were fully consumed.

Parahydrogen enrichment to ≈90% was achieved with the commercially available *Parahydrogen pH_2 Generator* from *Bruker BioSpin GmbH.*

IR: Alpha Platinum ATR (Bruker), wavenumbers (ṽ) in cm^{-1}.

MS (EI): Finnigan MAT 8200 (70 eV), ESI-MS: ESQ 3000 (Bruker) or Thermo Scientific LTQ-FT or Thermo Scientific Exactive. HRMS: Bruker APEX III FT-MS (7 T magnet) or MAT 95 (Finnigan)

or Thermo Scientific LTQ-FT or Thermo Scientific Exactive. GC-MS was measured on a Shimadzu GCMS-QP2010 Ultra instrument.

Unless stated otherwise, all commercially available compounds (abcr, Acros, TCI, Aldrich, Alfa Aesar) were used as received. The ruthenium complexes [Cp*RuCl]$_4$ (**C1**),[2,3] [Cp*Ru(MeCN)$_3$]PF$_6$ (**C2**),[4] [CpRu(MeCN)$_3$]PF$_6$ (**C3**),[5] [Cp^{T}Ru(MeCN)]PF$_6$ (**C4**),[6,7] and [Cp^{COOMe}Ru(MeCN)$_3$]PF$_6$ (**C5**)[7] were prepared according to literature procedures, in part modified as previously described by our group. They were stored under argon but were weighed in air.

Hydrogen gas (N50, ≥99.999 Vol.%) was purchased from AirLiquide and was used without further purification.

Kinetic Studies on the Decomposition of Cationic Pianostool Carbenes

Kinetic studies were conducted with isolated carbene complexes that had been previously characterized.[7] A representative procedure is outlined below.

Preparation of the Cationic Ruthenium Carbenes

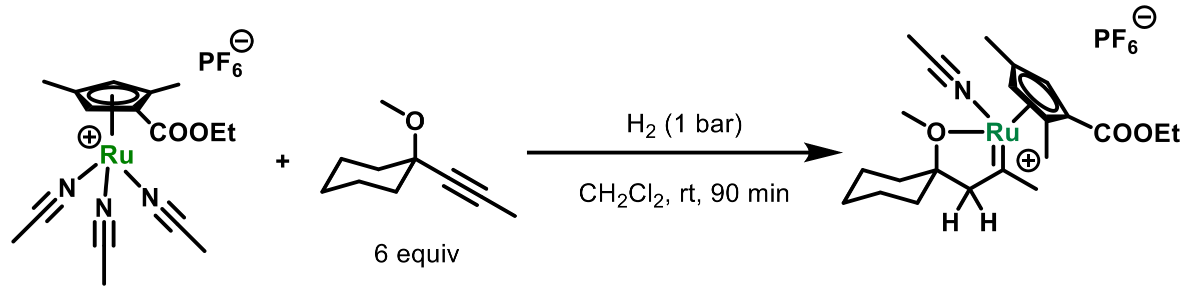

In a Schlenk tube, tris(acetonitrile)(η^5-1-ethoxycarbonyl-2,4-dimethylcyclopentadienyl)ruthenium hexafluorophosphate (**C4**, 169 mg, 0.316 mmol) was dissolved in CH_2Cl_2 (10 mL). 1-Methoxy-1-(prop-1-yn-1-yl)cyclohexane (288 mg, 1.89 mmol) was added via syringe and the tube was then closed with a septum. Hydrogen gas was flushed through the solution with a long cannula for 3 min, causing a color change from orange to cherry red. The solution was stirred for another 90 min before the volume was reduced to about 1 mL under high vacuum. Pentane (10 mL) was quickly added to the mixture causing the formation of a dark red oil. The supernatant was removed with a filter cannula and the residual oil was washed with pentane (3 x 10 mL). The residue was dried under high vacuum to provide the cationic ruthenium carbene as a dark red foamy, waxy

solid. The analytical data was in accordance with those reported previously (*Note: Due to the planar chiral Cp ligand, the ruthenium carbene was obtained as a 1.8:1 mixture of diastereomers*).[7]

prepared analogously

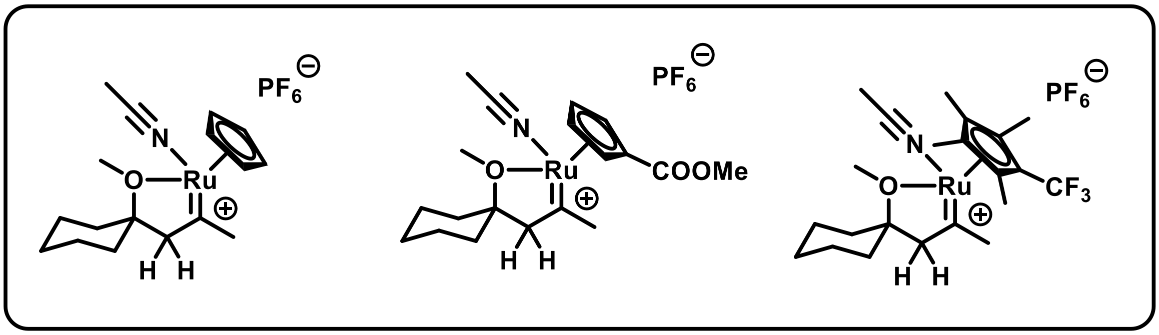

To study the intrinsic stability of these carbenes, their decomposition was monitored by means of ^1H NMR spectroscopy.

Decomposition Kinetics. A flame-dried J. Young NMR tube was charged under Ar with the corresponding carbene complex (270 µmol) and CD_2Cl_2 (0.7 mL). The resulting solution was quickly frozen by immersing the tube into liquid nitrogen for transport to the NMR spectrometer. Once inserted into the probe head, the sample was equilibrated at 25 ºC before ^1H NMR spectra were acquired every 60 sec over the course of 12 hours. The obtained raw data was processed using the MestReNova reaction monitoring plugin. The decay of ^1H NMR signals was used to prepare conversion/time plots, with the first acquired spectra accounting for 100% residual carbene.

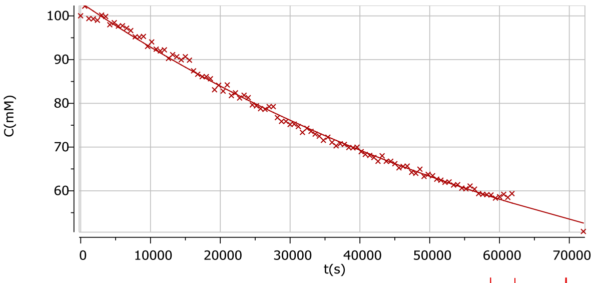
C(mM)
t(s)

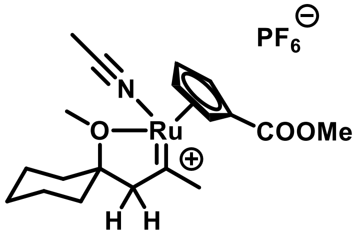
PF6
N
O
Ru
COOMe
H H

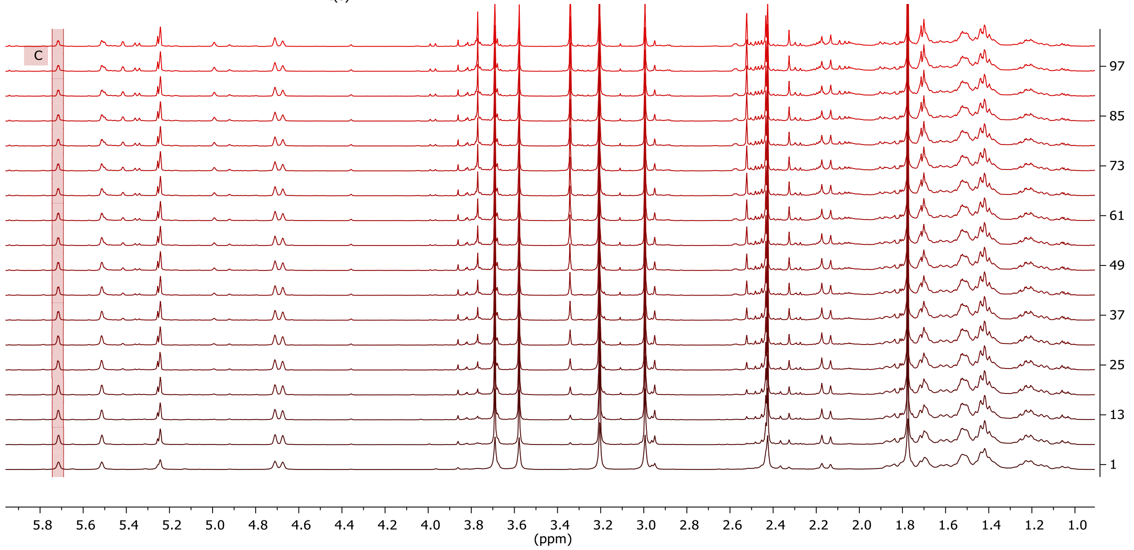
C
(ppm)

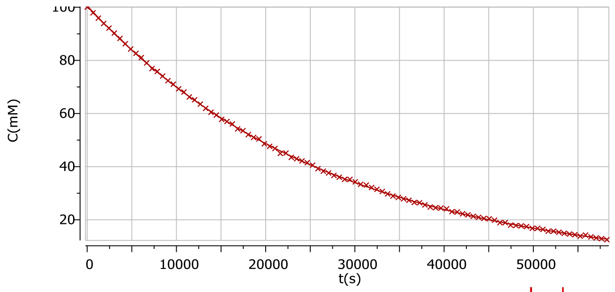
C(mM)
t(s)

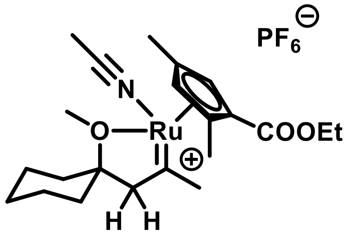
PF6
N
O
Ru
COOEt
H H

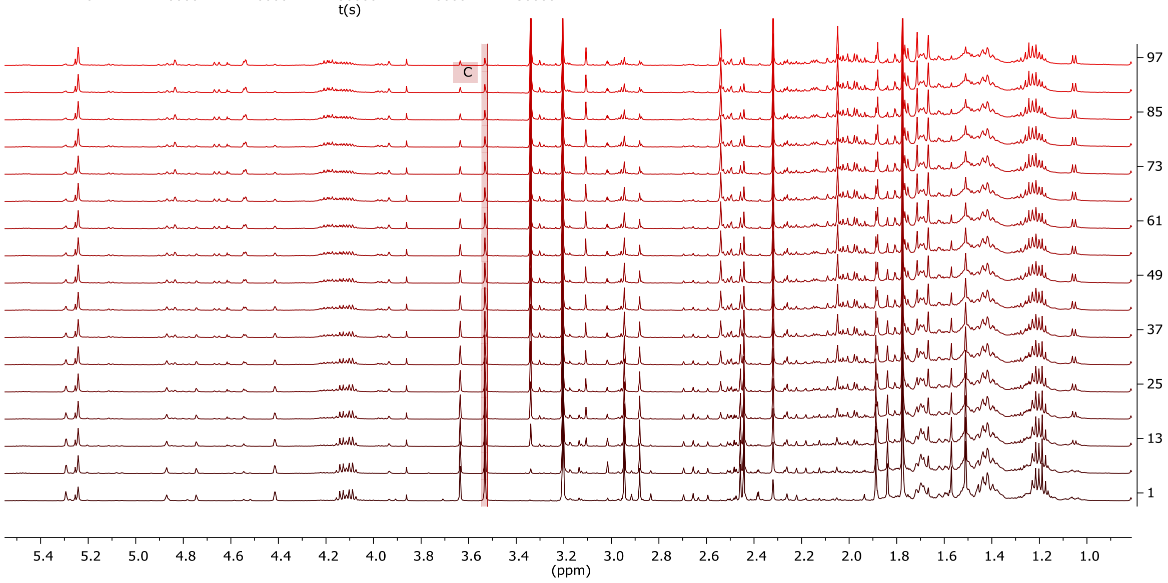
C
(ppm)

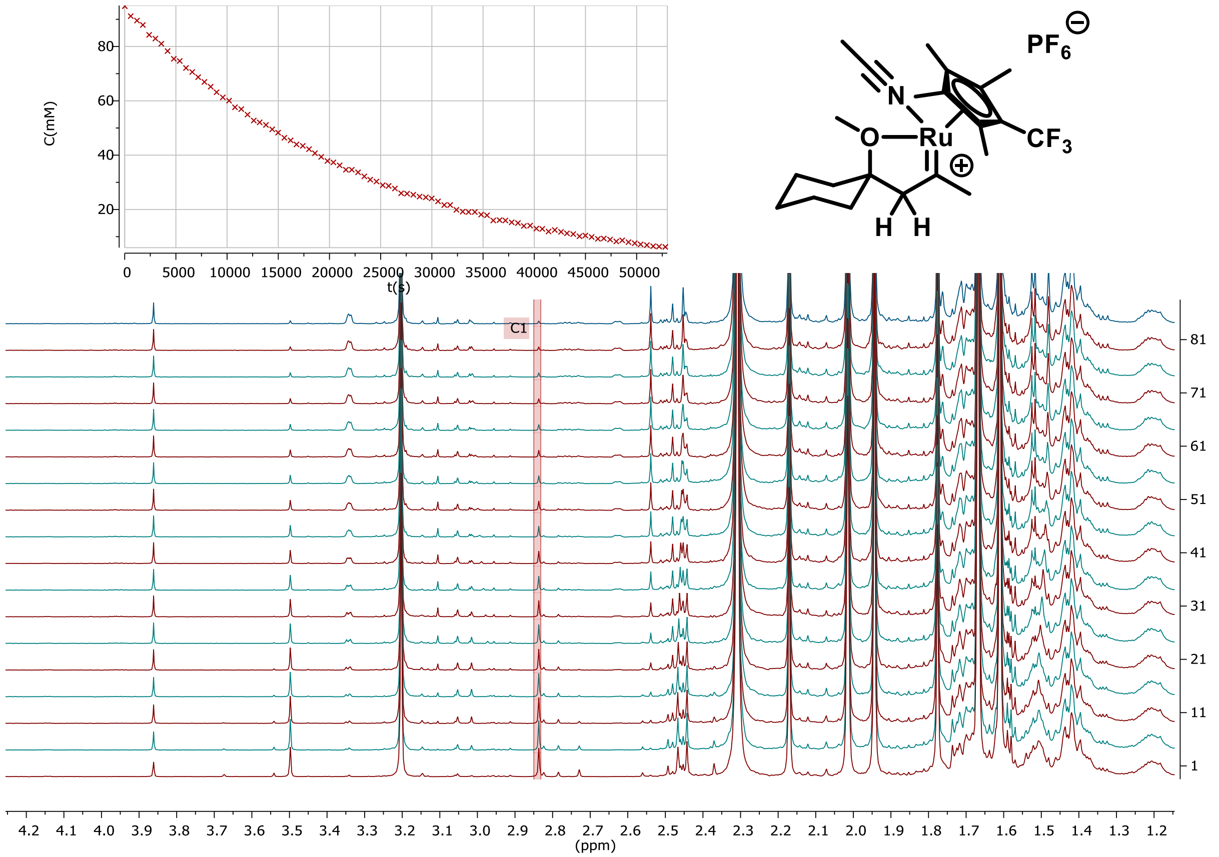
C(mM)
t(s)
PF6
N
O
Ru
CF3
H H
C1
(ppm)

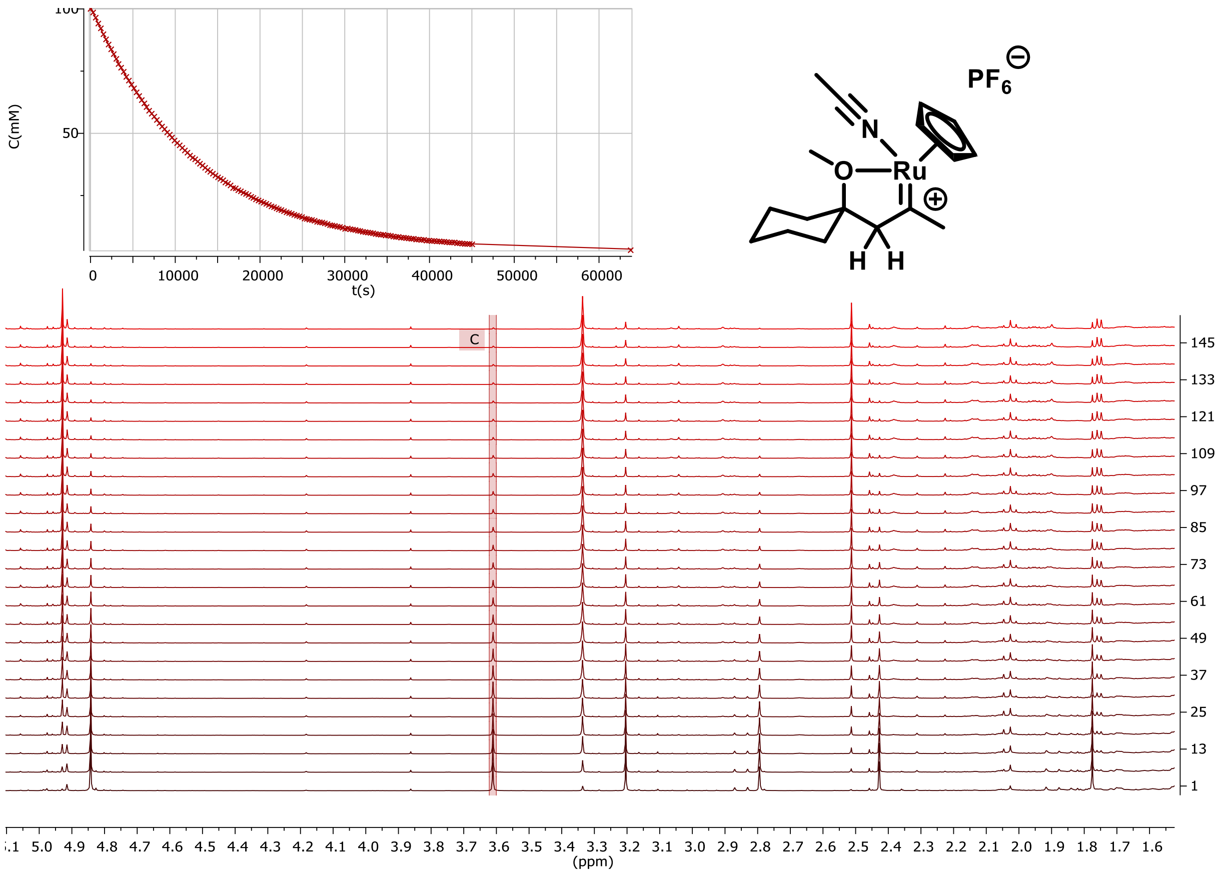
C(mM)
t(s)
PF6
N
O
Ru
H H
C
(ppm)

Data Processing. The raw data was then imported into Excel and plots of log(conc.) vs. time were prepared. The resulting linear graphs indicate that the decomposition of the carbene complexes follow first-order kinetics. The slope of the obtained lines gives rise to the $-k_{obs}$ values of the individual decomposition reaction, which reflect the different thermodynamic stabilities.

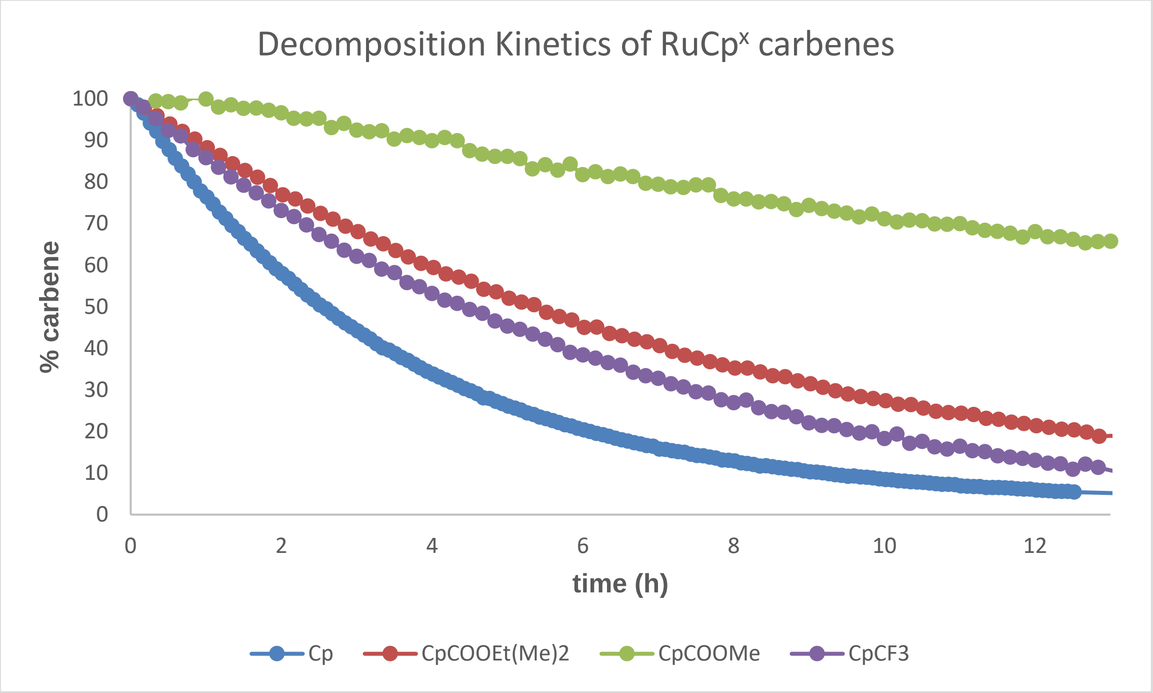

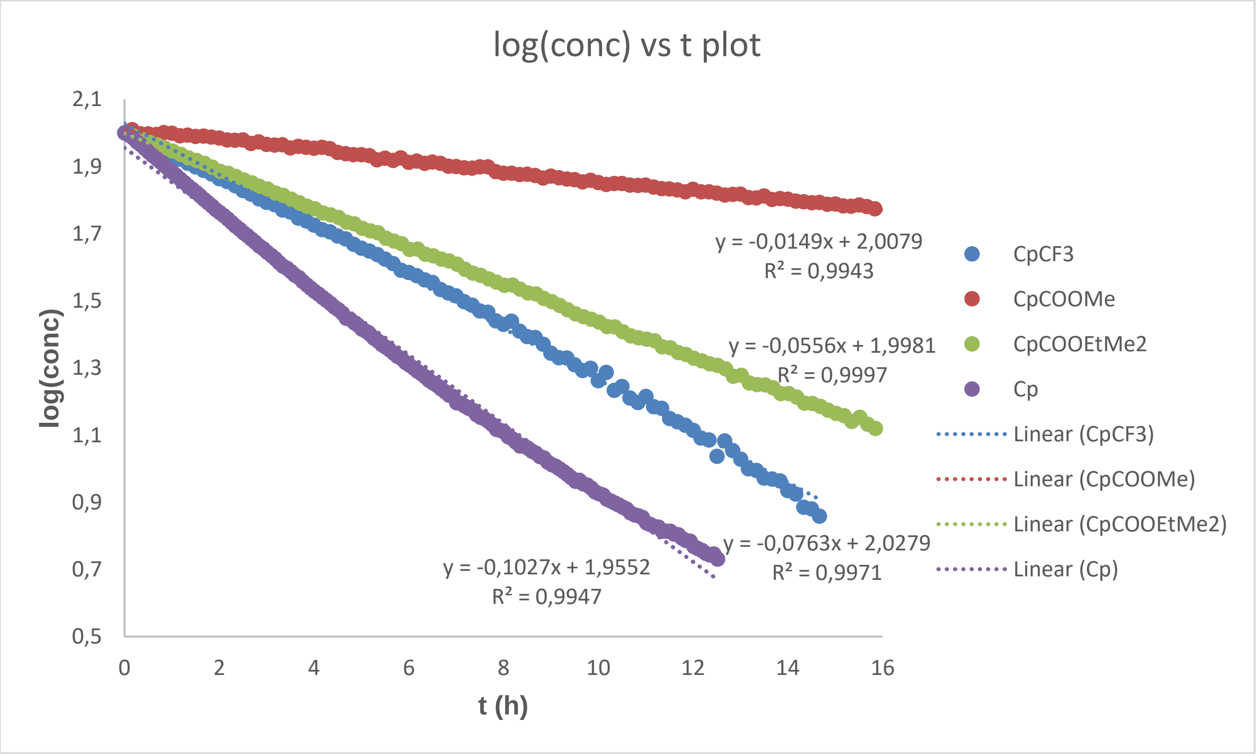

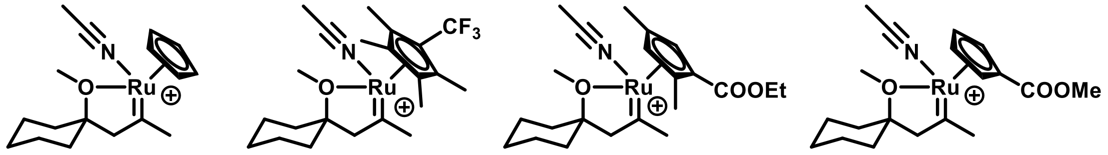

$E_{1/2}$ (V)	0.652	0.711	0.754	0.903
k_{obs} (h^{-1})	0.1027	0.0763	0.0556	0.0149
k_{rel}	1	1.4	1.9	6.8

The k_{obs} values were also plotted against one-electron oxidation potentials ($E_{1/2}$) of the corresponding $[Cp^XRu(MeCN)_3]PF_6$ complexes measured by cyclic voltametry.[7] The good correlation (R^2=0.98) between these two variables indicates that the electronic properties of the Cp^x ligand is the prime factor that influences their thermodynamic stability.

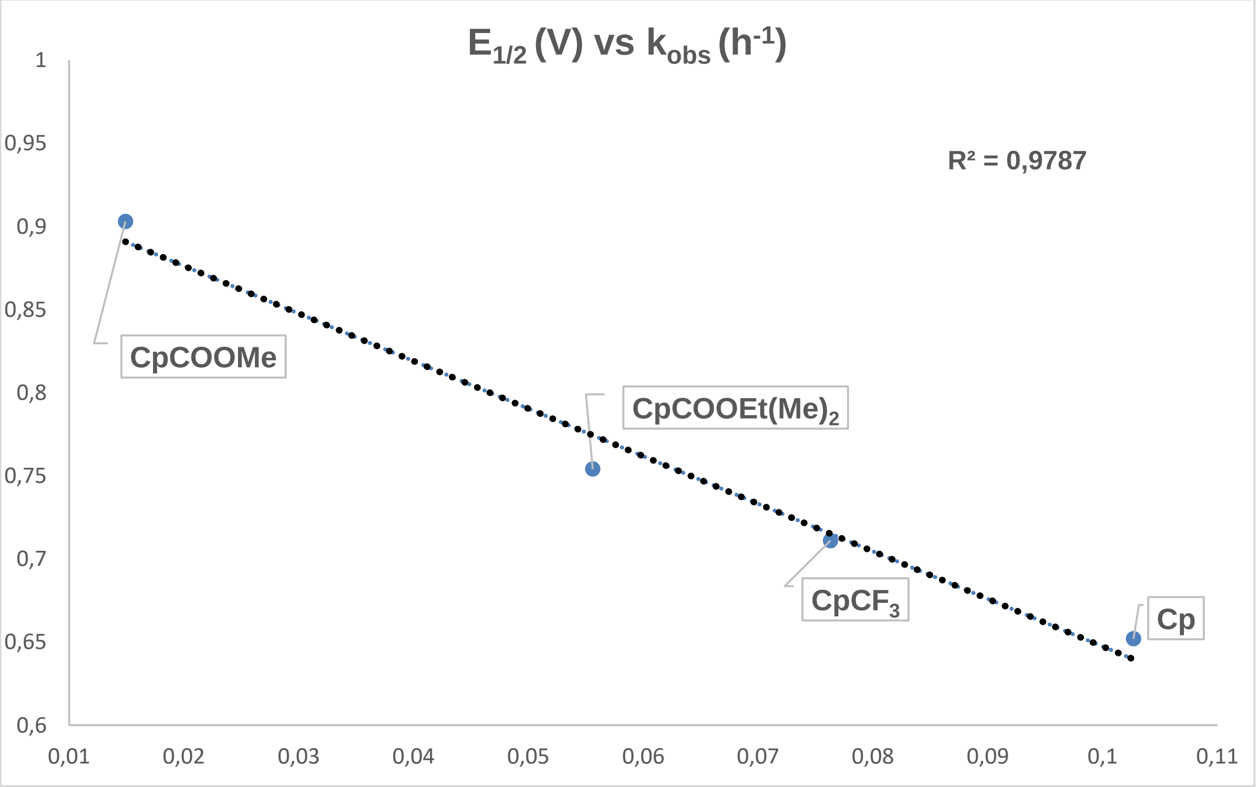

Analysis of the Decomposition Products. We attempted to identify the decomposition product(s) of the cationic Fischer carbenes. Extensive NMR analysis of the sample after 24 h reaction time suggests that the carbene fragment transforms into the diene complex **S1**. A complete NMR analysis supporting this structure is given below.

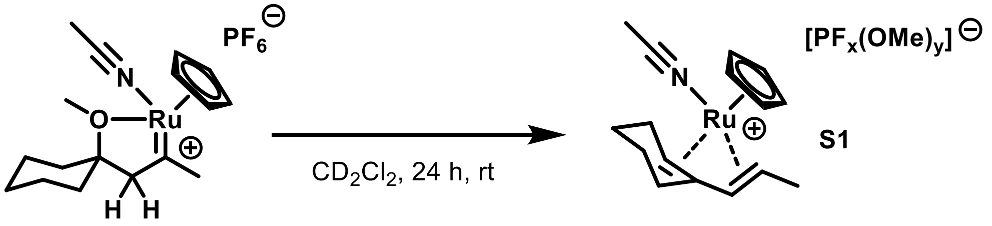

Whilst the assignment of the cationic part of the complex is unambiguous, we are uncertain about the constitution of the anion. Several new minor PF_xOMe_y species were detected, indicating that the released methanol reacts with the PF_6 anion. Likely for this heterogeneity, isolation of this complex in analytically pure form was not successful.

The connectivities observed in the HMBC and NOESY datasets support the structure of the cation.

A similar complex is known in the literature:
Chem. Commun**,** 2000, 217-218
https://doi.org/10.1039/A909355I

The peaks at 3.42 ppm (1H) and 50.8 ppm (13C) match quite well with free MeOH in DCM so that it can be almost exculded that it is attached to the complex.

The nature of the anion is unclear. There is BF_4^- as well as PF_6^- found in the sample. Furthermore the free MeOH seems to react over time (see later pages) with PF6 as there are small 31P couplings on the newly formed signals visible in the 1H and 13C. There were no signs of decompostion of the cation found after 24h.

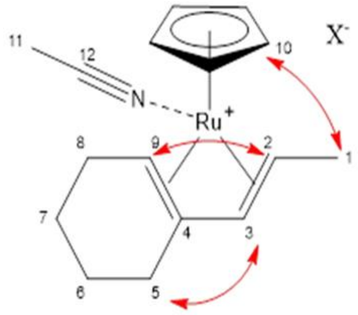

Relevant NOE cross peaks

Atom	δ (ppm)	J	COSY	HSQC	HMBC	NOESY
1 C	20.94			1	2, 3	
H3	1.83	0.50(3), 6.17(2)	2, 3	1	2, 3	3, 10
2 C	69.56			2	1, 3, 5''	
H	2.02	9.70(3), 6.17(1)	1, 3	2	1, 3, 4	9
3 C	85.68			3	1, 2, 5', 9	
H	5.39	0.50(1), 9.70(2)	1, 2	3	1, 2, 5, 9	1, 5', 5''
4 C	106.72				2, 5', 5'', 6', 6'', 8, 9	
5 C	30.02			5', 5''	3, 6', 6'', 7', 7'', 9	
H'	2.97		5'', 6', 6''	5	3, 4, 6, 7, 9	3
H''	2.15		5', 6', 6''	5	2, 4, 6, 7	3, 10
6 C	22.35			6', 6''	5', 5'', 7', 8	
H'	1.68		5', 5'', 7', 7''	6	4, 5, 7, 8	
H''	1.76		5', 5'', 7', 7''	6	4, 5, 7, 8	
7 C	22.03			7', 7''	5', 5'', 6', 6'', 7'', 8, 9	
H'	1.76		6', 6'', 7'', 8	7	5, 6, 8, 9	
H''	1.49		6', 6'', 7', 8	7	5, 7, 8, 9	
8 C	27.97			8	6', 6'', 7', 7'', 9	
H2	2.21		7', 7'', 9	8	4, 6, 7, 9	
9 C	77.66			9	3, 5', 7', 7'', 8	
H	2.44		8	9	3, 4, 5, 7, 8	2
10 C	84.89			10	10	
H	4.99			10	10	1, 5''
11 C	4.97			11		
H3	2.57			11	12	
12 C	133.01				11	

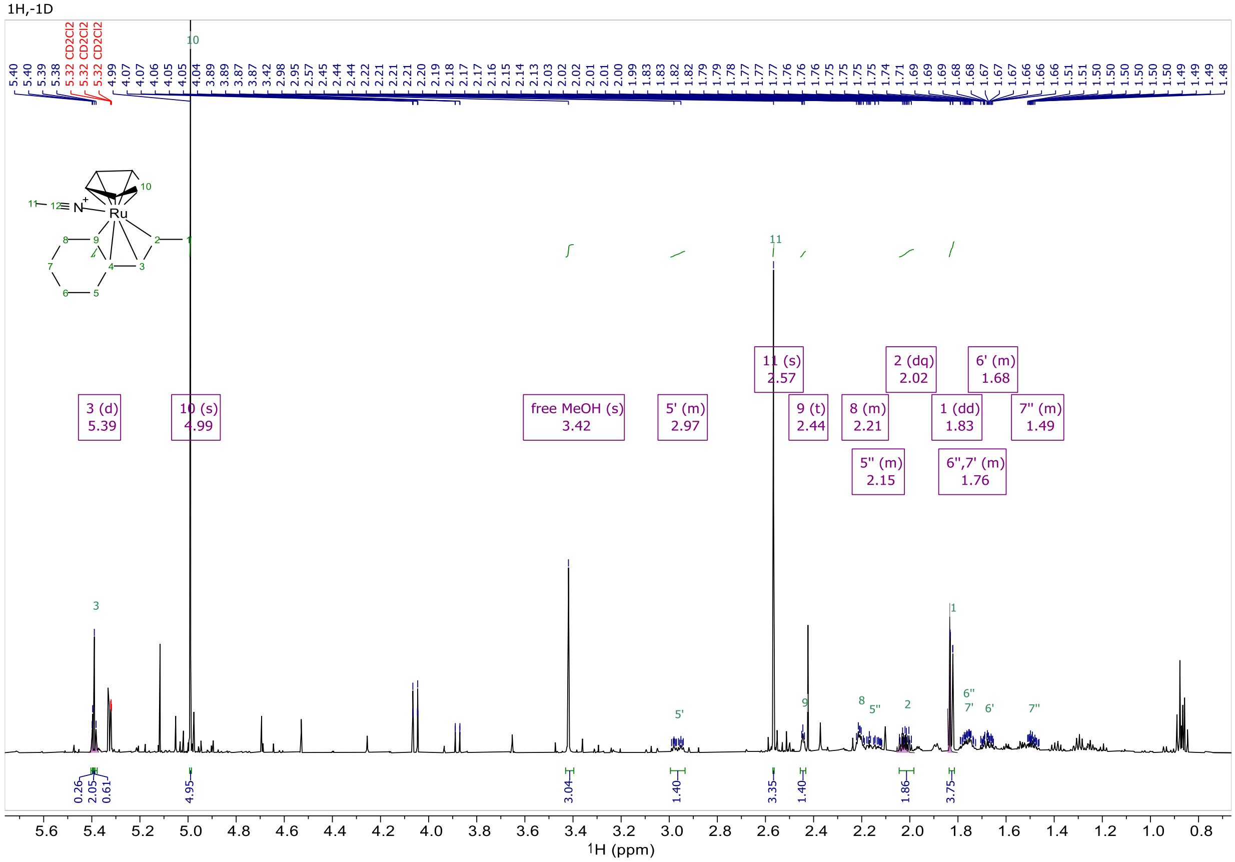
1H,-1D
3 (d)
5.39
10 (s)
4.99
free MeOH (s)
3.42
5' (m)
2.97
11 (s)
2.57
9 (t)
2.44
8 (m)
2.21
5'' (m)
2.15
2 (dq)
2.02
1 (dd)
1.83
6'',7' (m)
1.76
6' (m)
1.68
7'' (m)
1.49
0.26
2.05
0.61
4.95
3.04
1.40
3.35
1.40
1.86
3.75
1H (ppm)

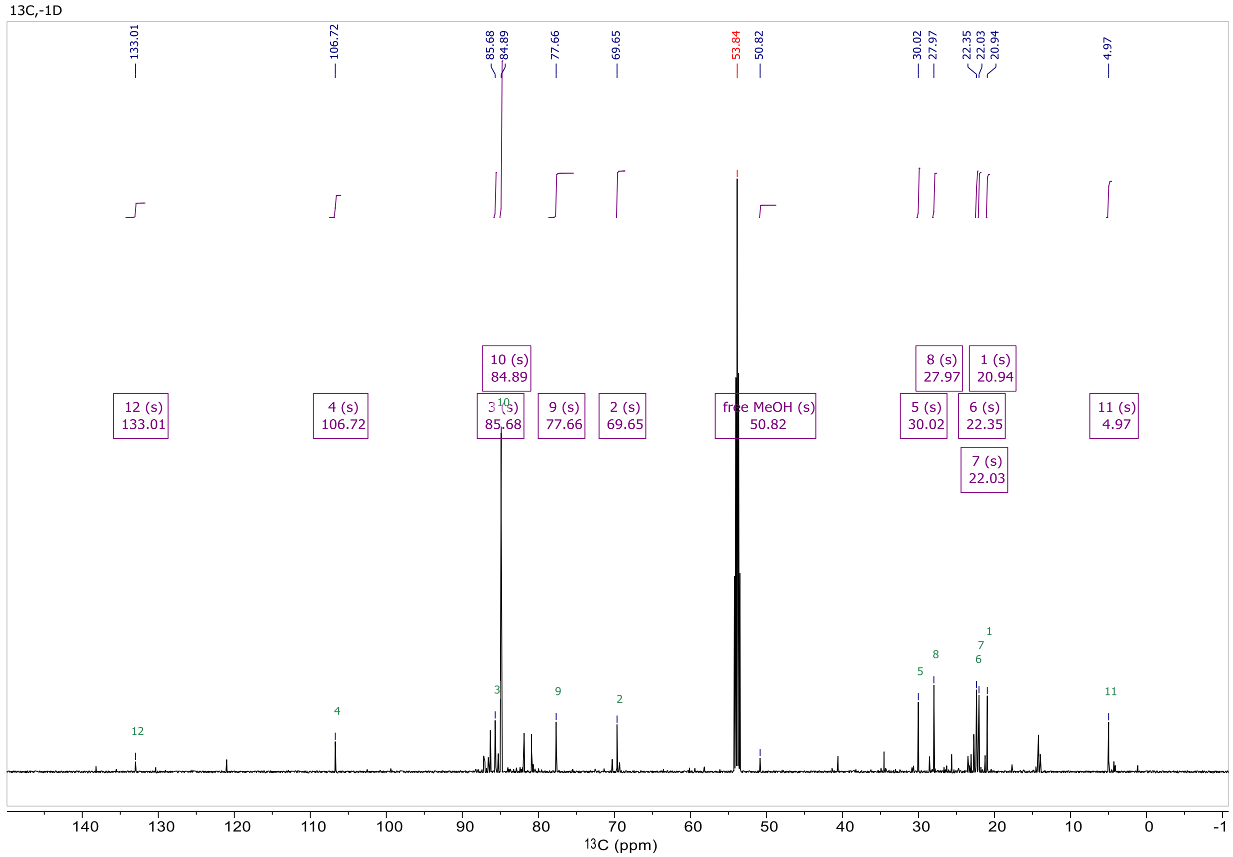
13C,-1D
133.01
106.72
85.68
84.89
77.66
69.65
53.84
50.82
30.02
27.97
22.35
22.03
20.94
4.97
12 (s)
133.01
4 (s)
106.72
10 (s)
84.89
3 (s)
85.68
9 (s)
77.66
2 (s)
69.65
free MeOH (s)
50.82
8 (s)
27.97
1 (s)
20.94
5 (s)
30.02
6 (s)
22.35
7 (s)
22.03
11 (s)
4.97
13C (ppm)

1H,1H-COSY

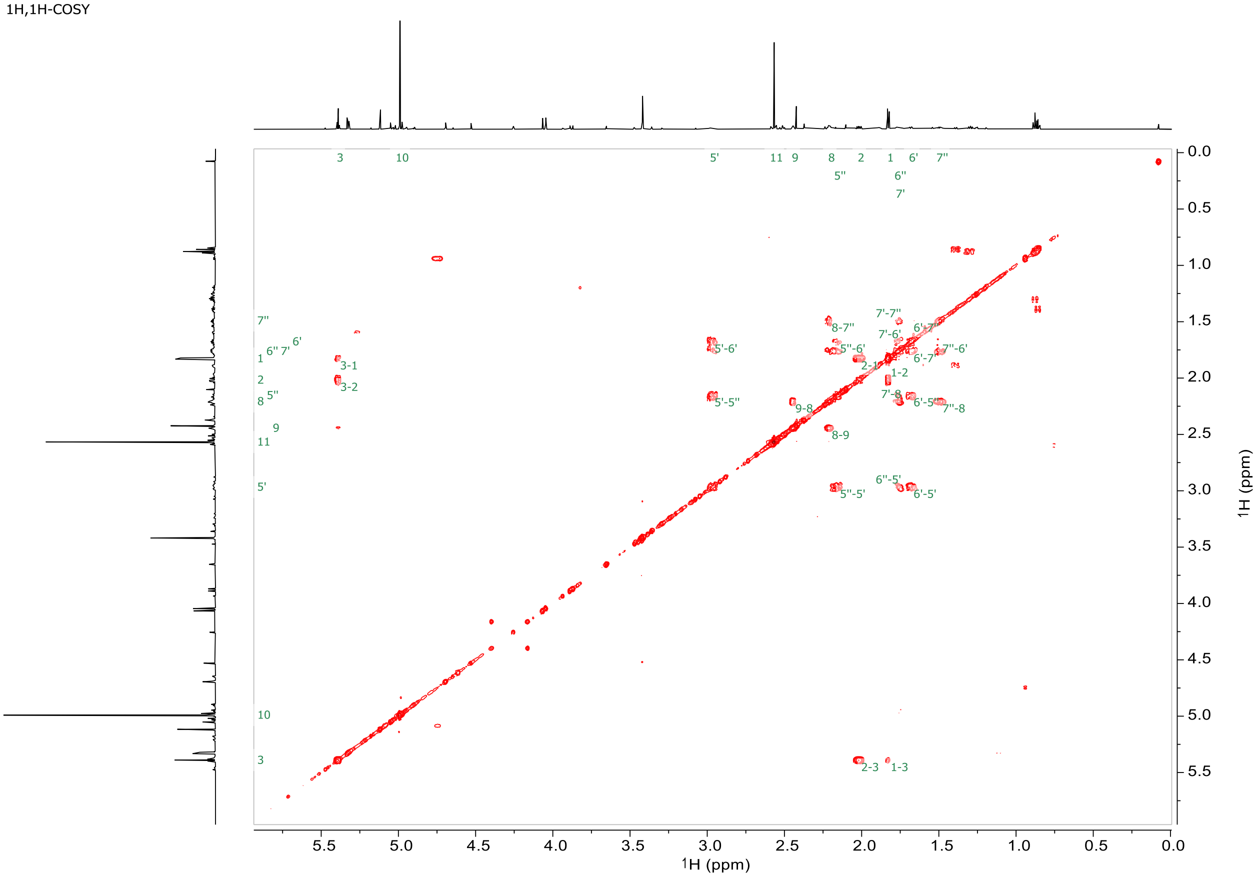

1H,13C-HMBC

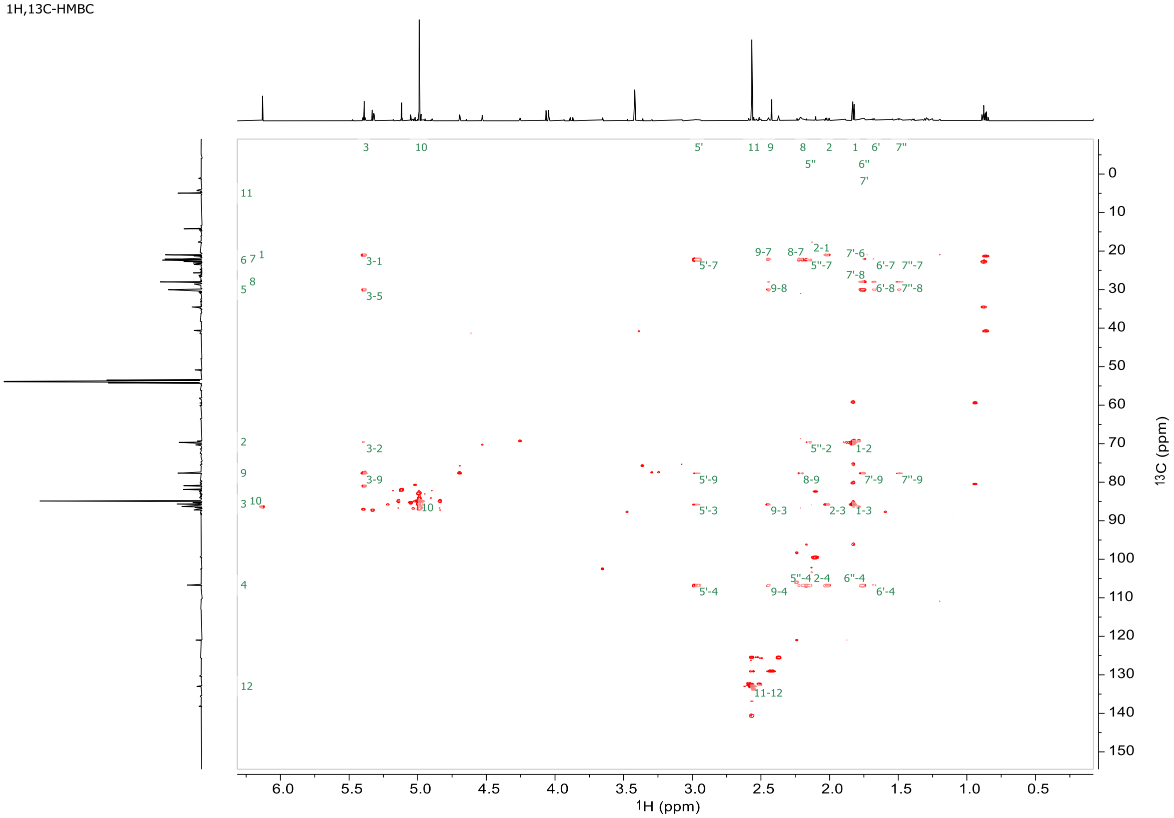

1H,1H-NOESY

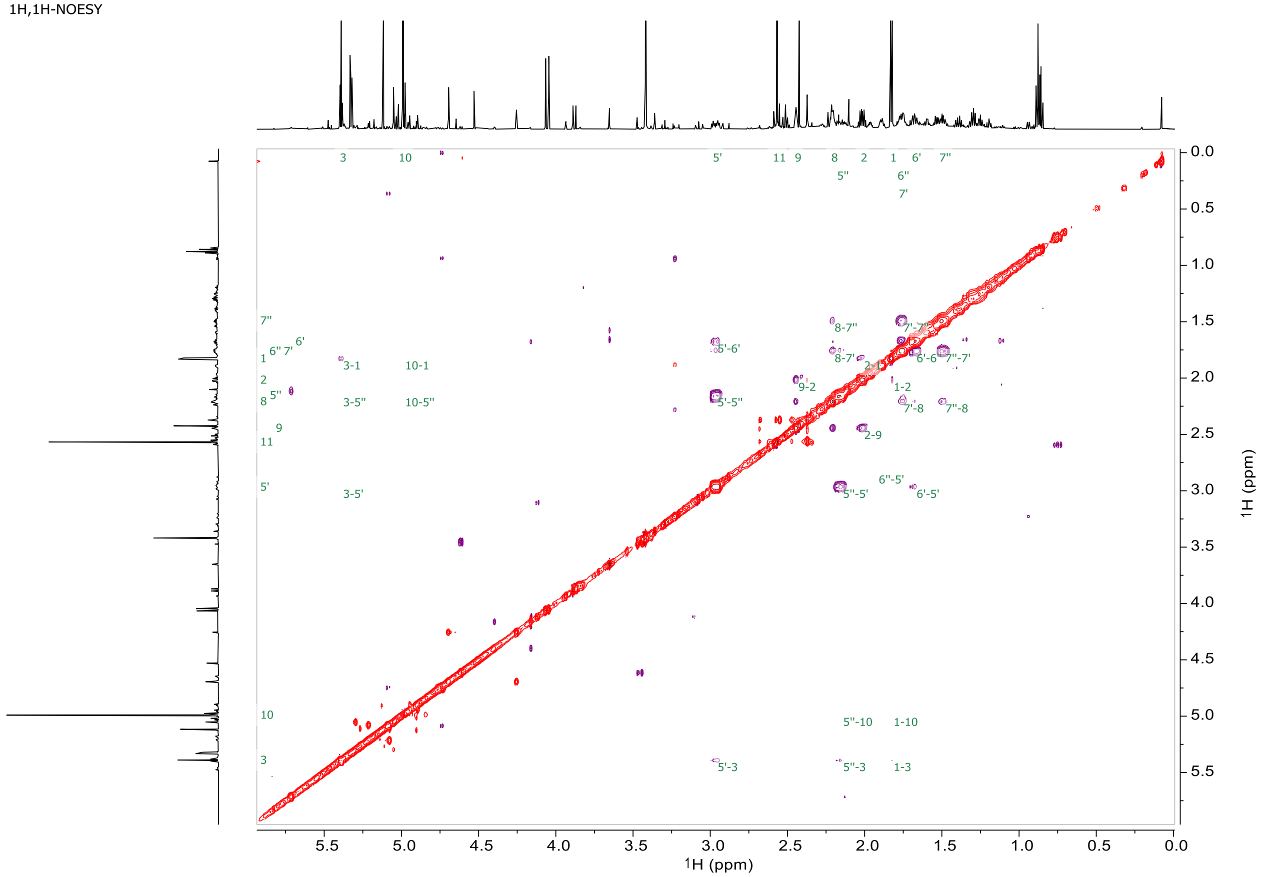

1H,13C-HSQC-EDITED

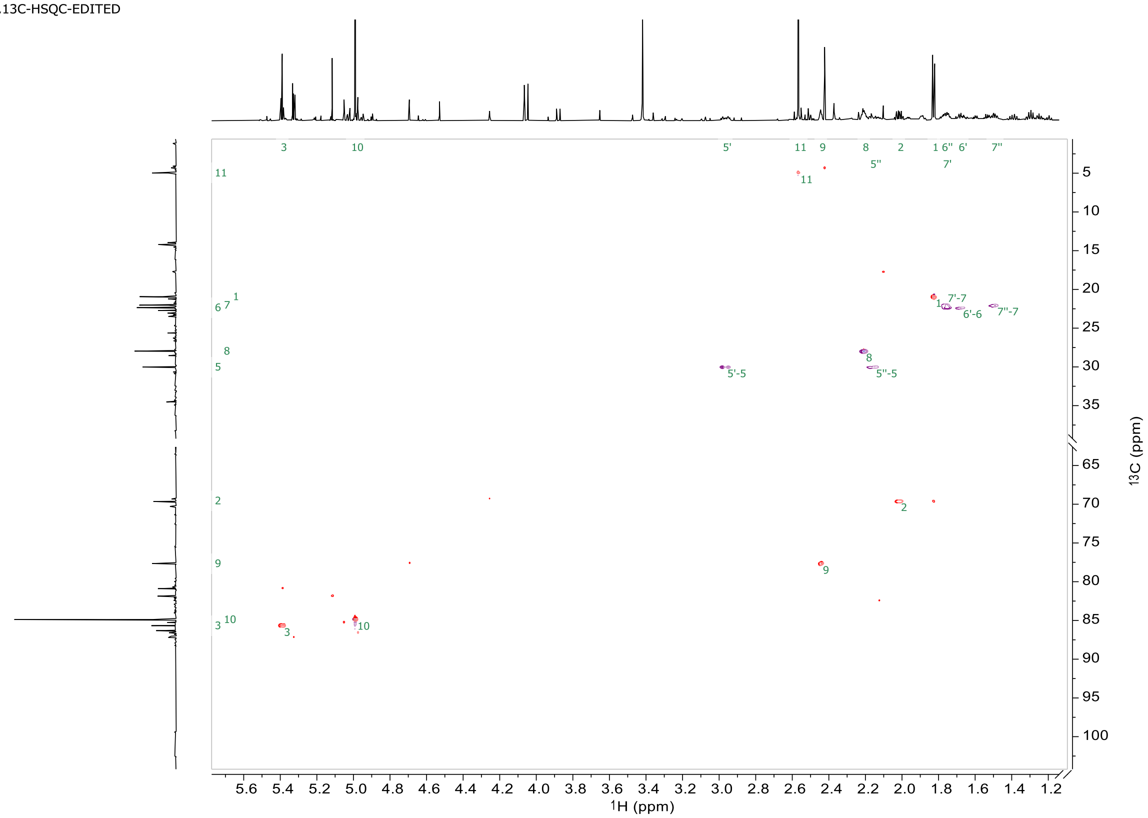

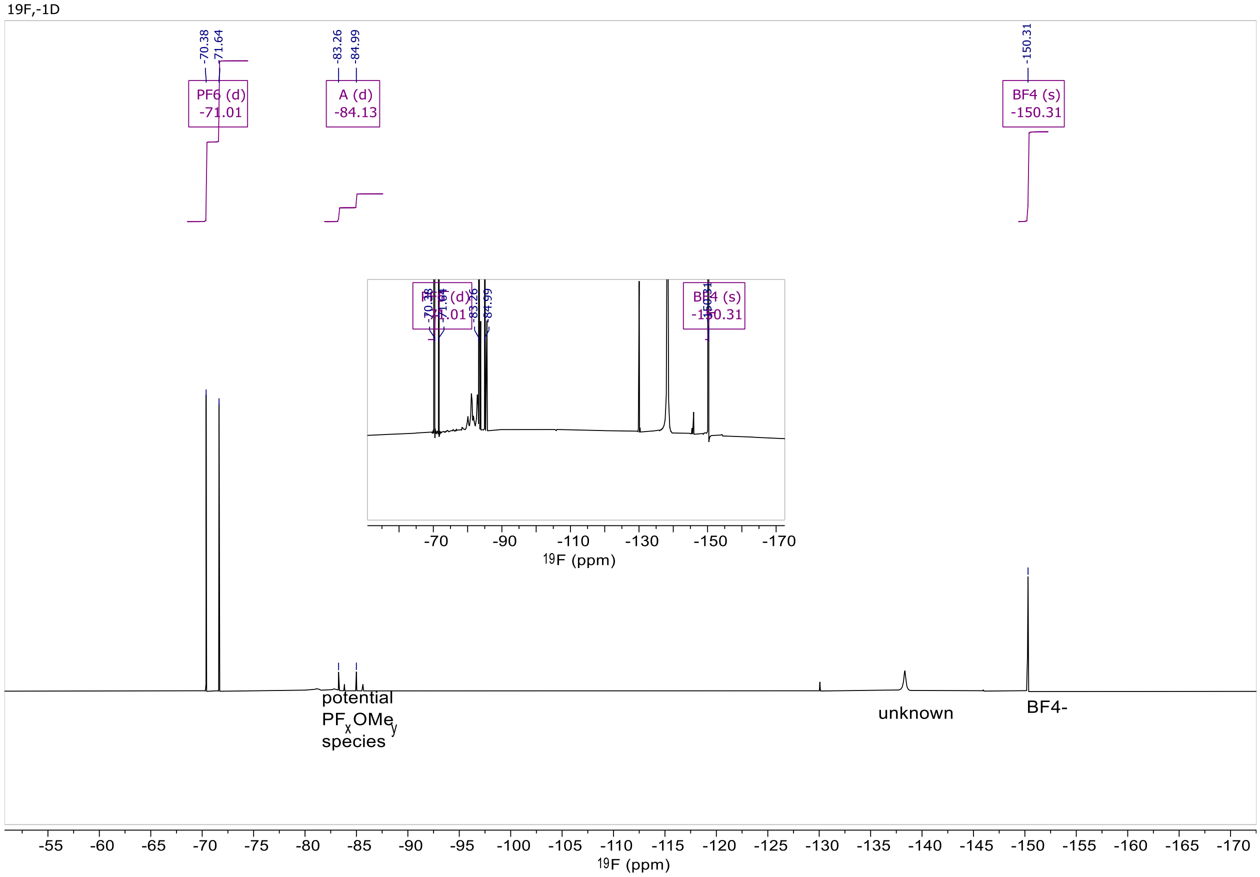
19F,-1D
-70.38
-71.64
-83.26
-84.99
-150.31
PF6 (d)
-71.01
A (d)
-84.13
BF4 (s)
-150.31
potential
PFxOMey
species
unknown
BF4-
19F (ppm)

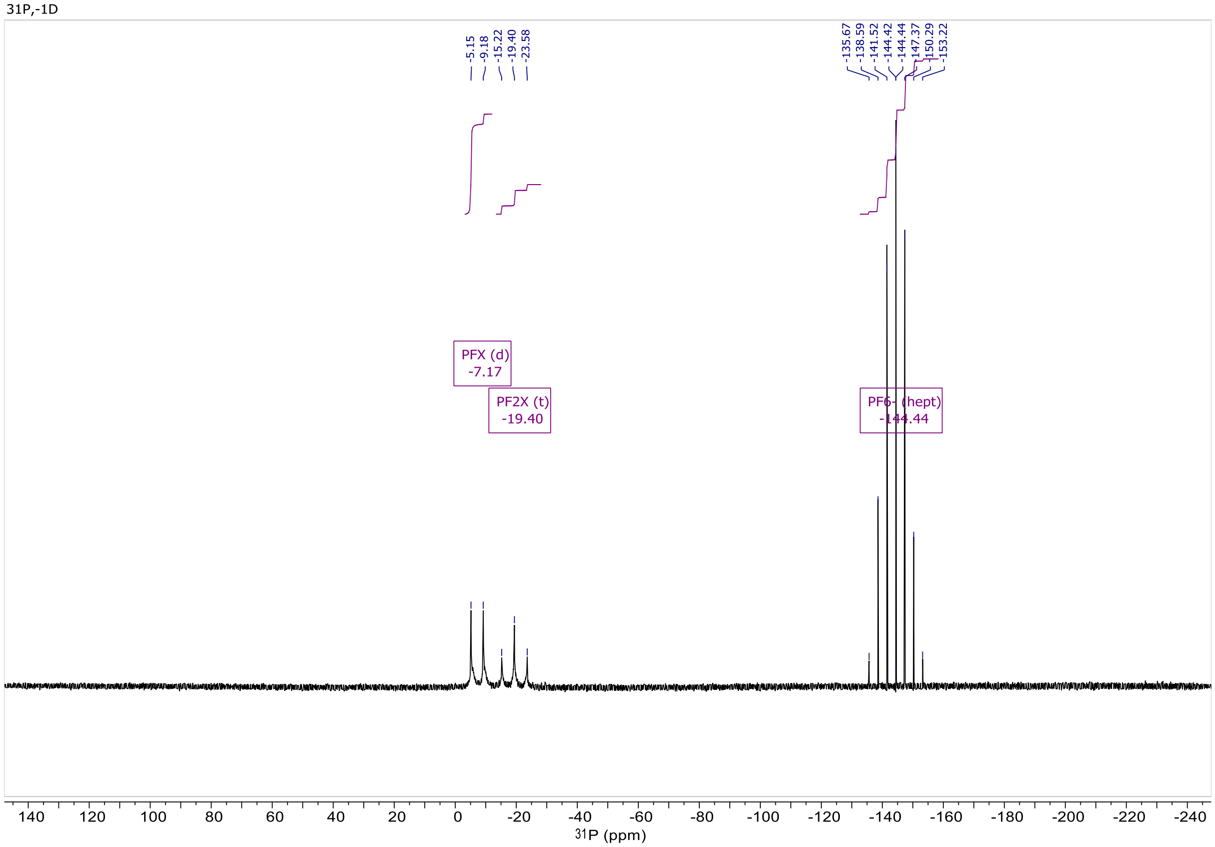
31P,-1D
-5.15
-9.18
-15.22
-19.40
-23.58
-135.67
-138.59
-141.52
-144.42
-144.44
-147.37
-150.29
-153.22
PFX (d)
-7.17
PF2X (t)
-19.40
PF6- (hept)
-144.44
31P (ppm)

Control Experiments

***tert*-Butyl((2-(cyclohepta-1,5-dien-1-yl)propan-2-yl)oxy)dimethylsilane (11).** A flame-dried Schlenk tube under argon was charged with $[CpRu(MeCN)_3]PF_6$ (**C3**, 8.6 mg, 0.020 mmol, 10 mol%). Methylene chloride (2.0 mL) and enyne **1a** (0.18 mmol) were added and the Schlenk tube was sealed with a septum. After 8 days of stirring at ambient temperature, the solution was diluted with pentane (4.0 mL) and filtered through a silica plug. The plug was washed with pentane/*tert*-butyl methyl ether (5:1, 12 mL) and the combined filtrates were evaporated. The residue was subjected to flash chromatography (SiO_2, pentane:*tert*-butyl methyl ether, 100:1) to provide the title compound (7 mg, 15%) as a colorless oil. The compound is unstable and decomposes within 12 h when stored neat or in CD_2Cl_2/$CDCl_3$ solution. **^{1}H NMR** (400 MHz, CD_2Cl_2) δ 5.78 (t, *J* = 6.9 Hz, 1H), 5.63 – 5.54 (m, 1H), 5.56 – 5.50 (m, 1H), 2.94 – 2.85 (m, 2H), 2.33 – 2.26 (m, 2H), 2.15 – 2.08 (m, 2H), 1.30 (s, 6H), 0.87 (s, 9H), 0.06 (s, 6H). **^{13}C NMR** (101 MHz, $CDCl_3$) δ 150.8, 130.3, 126.9, 121.4, 75.3, 29.0, 26.7, 26.3, 25.6, 25.0, 18.1, –2.7. **HRMS** (ESI) for $C_{16}H_{30}OSiNa$ $[M+Na]^+$: calcd. 289.19581, found 289.19604.

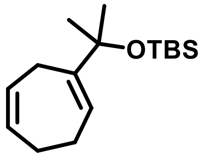

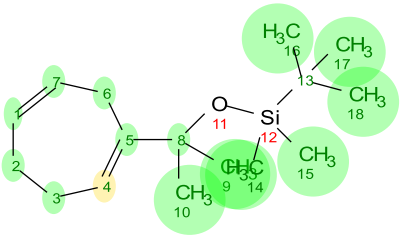

Assignments

Atom	Chemical Shift	J	COSY	HSQC	HMBC
1 C	130.25			1	2, 3, 6
H	5.54		7	1	
2 C	26.67			2	3, 4, 7
H2	2.12		3, 6, 7	2	1, 3, 4, 7
3 C	25.03			3	2, 4
H2	2.30	6.90(4)	2	3	1, 2, 4, 5
4 C	121.43			4	2, 3, 6
H	5.78	6.90(3)		4	2, 3, 5, 6, 8
5 C	150.82				3, 4, 6, 7, 10
6 C	26.28			6	4, 7
H2	2.90		2	6	1, 4, 5, 7, 8
7 C	126.92			7	2, 6
H	5.58		1, 2	7	2, 5, 6
8 C	75.29				4, 6, 10
9 C	29.03			9	10
H3	1.30			9	10

Assignments

Atom	Chemical Shift	J	COSY	HSQC	HMBC
10 C	29.03			10	9
H3	1.30		10	10	5, 8, 9
11 O					
12 Si					
13 C	18.07				14, 15, 16, 17, 18
14 C	-2.67			14	15
H3	0.06			14	13, 15
15 C	-2.67			15	14
H3	0.06			15	13, 14
16 C	25.64			16	17, 18
H3	0.87			16	13, 17, 18
17 C	25.64			17	16, 18
H3	0.87			17	13, 16, 18
18 C	25.64			18	16, 17
H3	0.87			18	13, 16, 17

^{13}C NMR

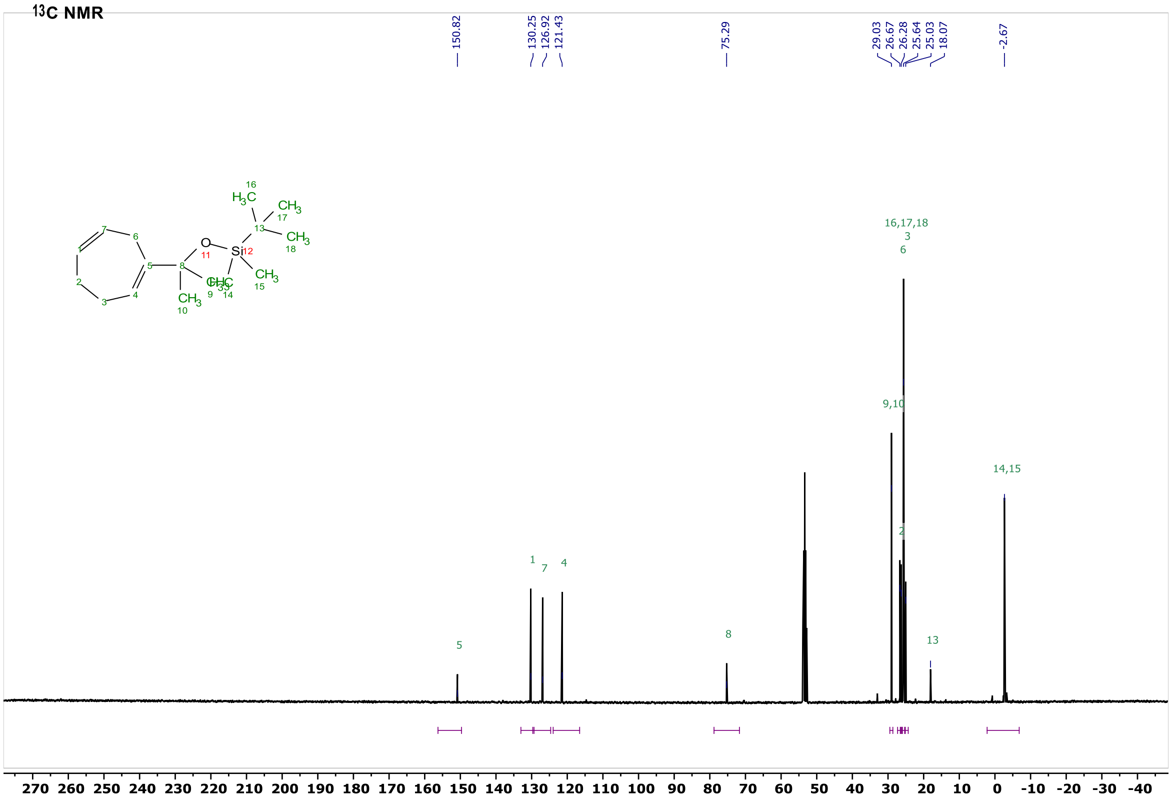

^{1}H NMR

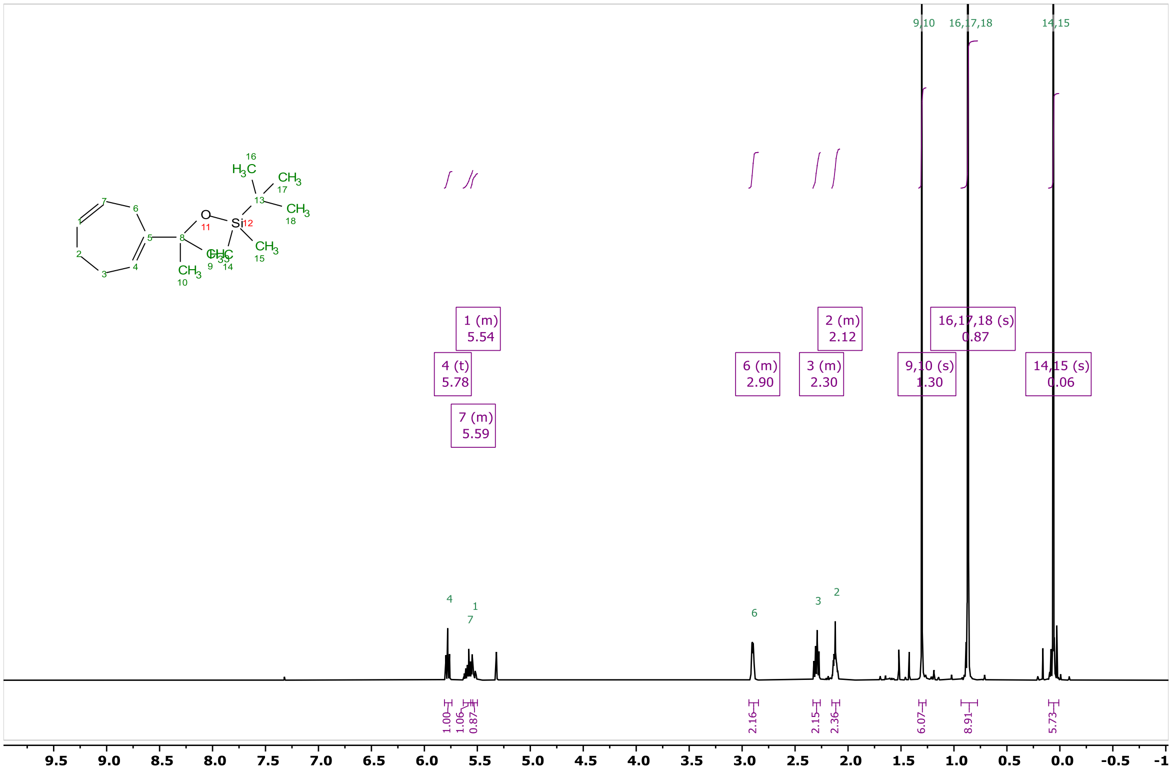

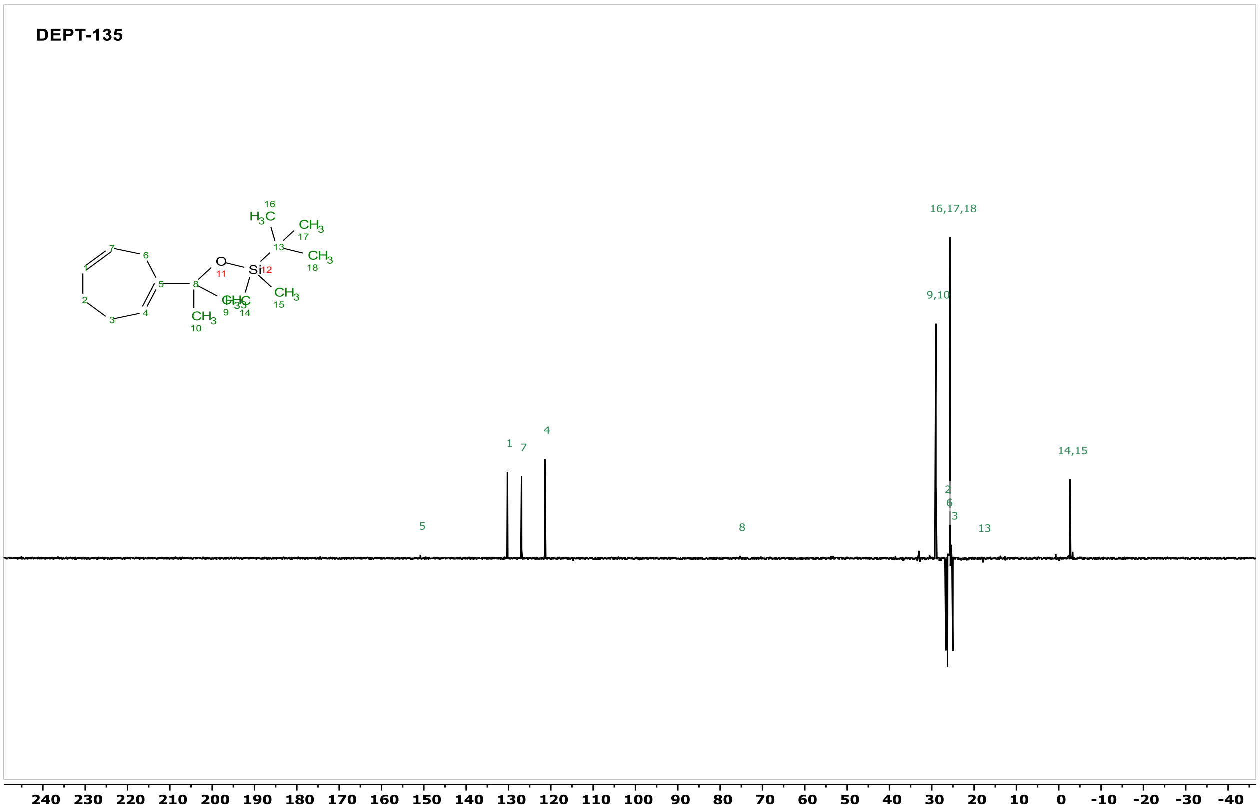
DEPT-135
16,17,18
9,10
1
7
4
5
8
2
6
3
13
14,15
240 230 220 210 200 190 180 170 160 150 140 130 120 110 100 90 80 70 60 50 40 30 20 10 0 -10 -20 -30 -40

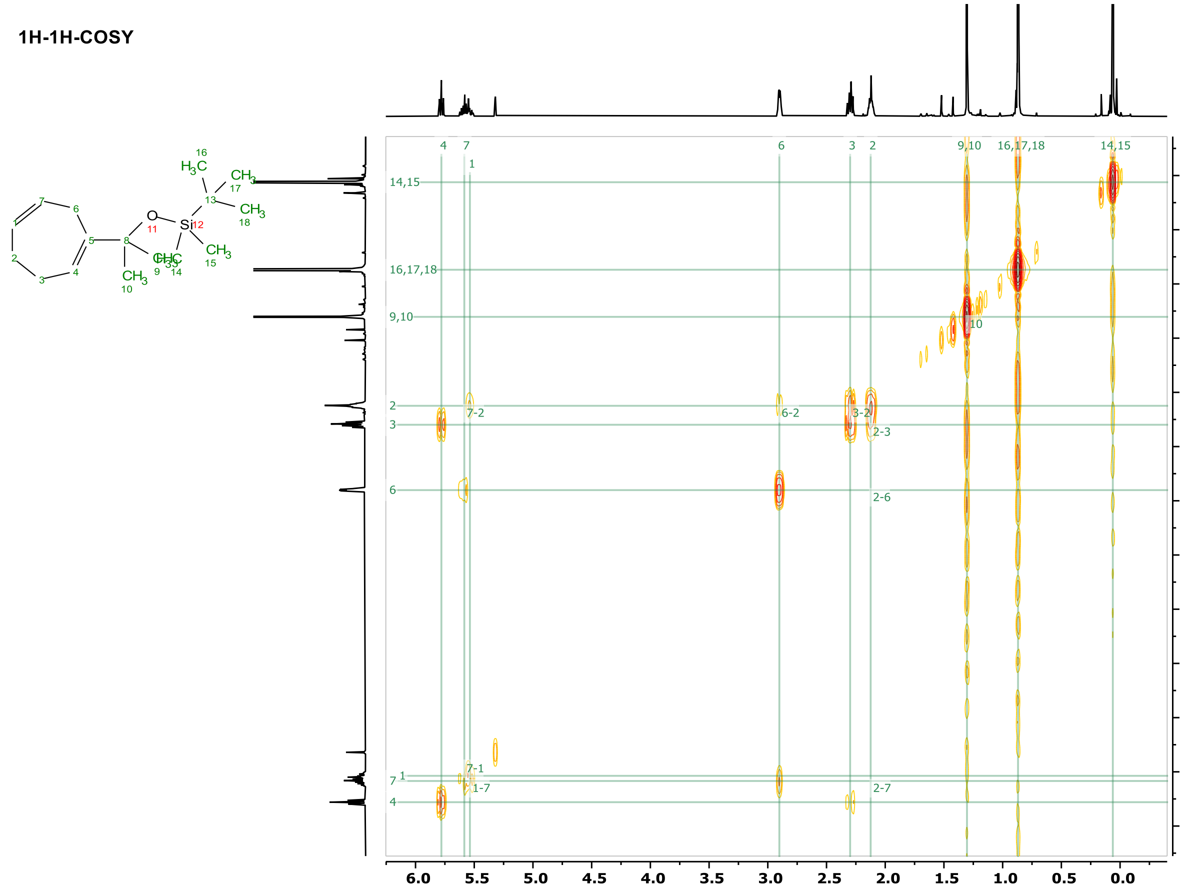
1H-1H-COSY
14,15
16,17,18
9,10
7-2
6-2
3-2
2-3
2-6
7-1
1-7
2-7
6.0 5.5 5.0 4.5 4.0 3.5 3.0 2.5 2.0 1.5 1.0 0.5 0.0

DEPT-45

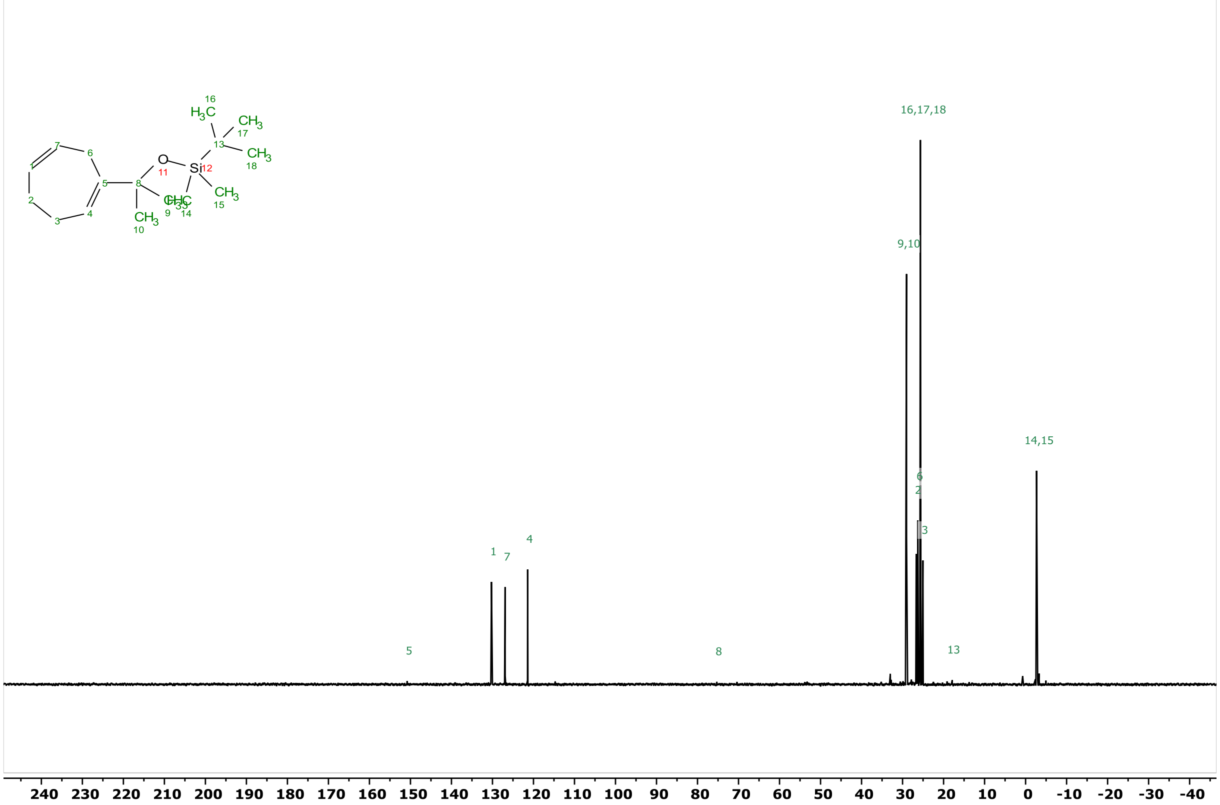

HSQC

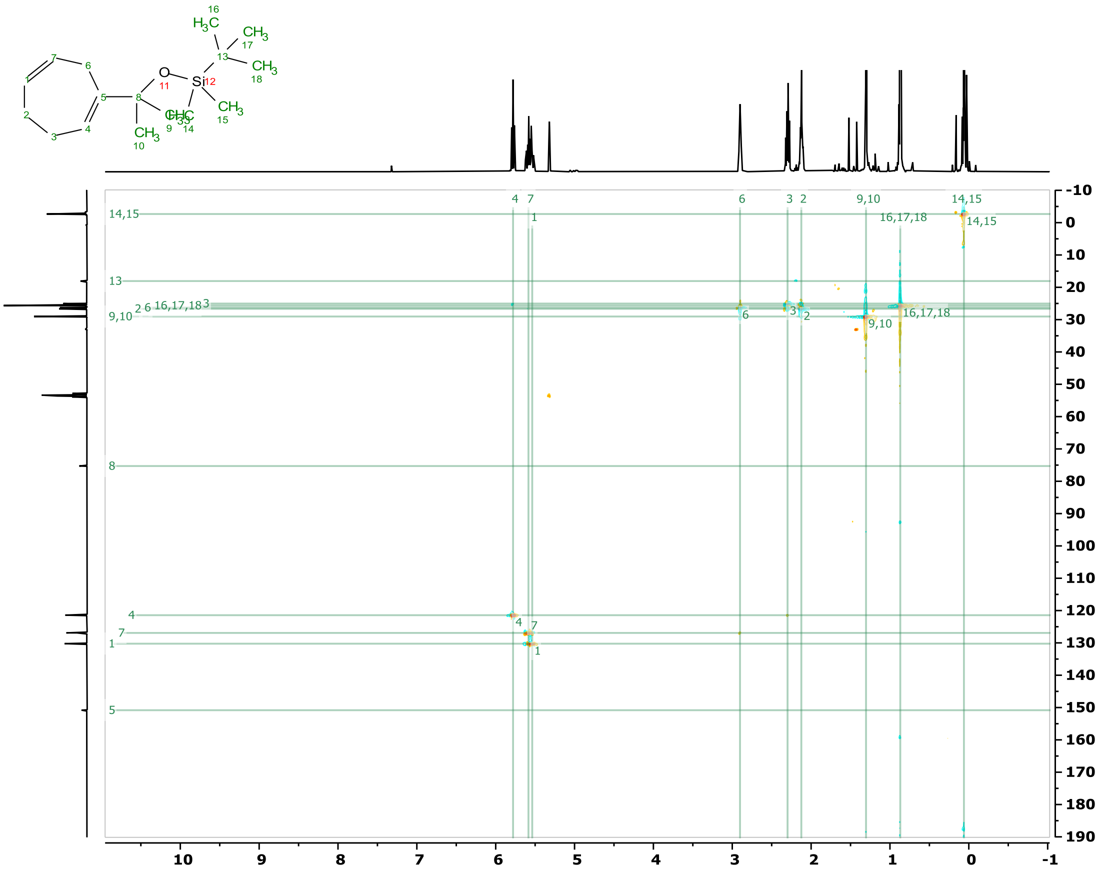

1-((1-Methoxycyclohexyl)methyl)cyclohex-1-ene (12) and 1-Methoxy-1-((2-methylcyclopent-1-en-1-yl)methyl)cyclohexane (4d).

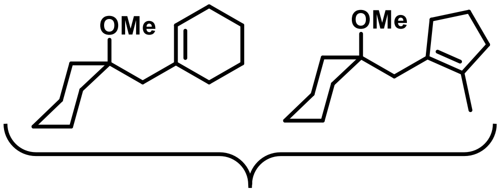

86:14

Colorless oil of inseparable isomers (32 mg, 80%); spectral and analytical data of compound **12**: **^{1}H NMR** (400 MHz, $CDCl_3$) δ 5.44 – 5.34 (m, 1H), 3.17 (s, 3H), 2.08 – 1.94 (m, 5H), 1.73 – 1.63 (m, 2H), 1.62 – 1.36 (m, 9H), 1.33 – 1.15 (m, 4H). **^{13}C NMR** (101 MHz, $CDCl_3$) δ 134.6, 125.0, 75.8, 48.2, 44.1, 34.4, 30.3, 26.0, 25.6, 23.3, 22.4, 22.1. **HRMS** (ESI) for $C_{14}H_{24}O_1$ $[M+Na]^+$: calcd: 231.17193, found: 231.17224.

Spectral and analytical data of compound **4d**: **^{1}H NMR** (400 MHz, $CDCl_3$) δ ^{1}H NMR (400 MHz, $CDCl_3$) δ 3.19 (s, 3H), 2.43 – 2.35 (m, 2H), 2.30 – 2.23 (m, 2H), 2.18 (s, 2H), 1.75 (p, *J* = 7.6 Hz, 2H), 1.63 – 1.37 (m, 13H). **^{13}C NMR** (101 MHz, $CDCl_3$) δ 133.17, 130.77, 76.20, 75.45, 47.22, 37.32, 36.87, 34.37, 24.83, 20.83, 13.59, 13.04. **HRMS** (ESI) for $C_{14}H_{24}O$ $[M+Na]^+$: calcd: 231.17193, found: 231.17224.

Two additional examples showed that the hydrogenative cycloisomerization of enynes with an O-alkyl substituent at the propargylic position leads to unselective formation of both the 6- and 5-membered cycloalkenes. O-silyl substituted enynes are hence necessary to achieve high selectivities for the 5-membered ring.

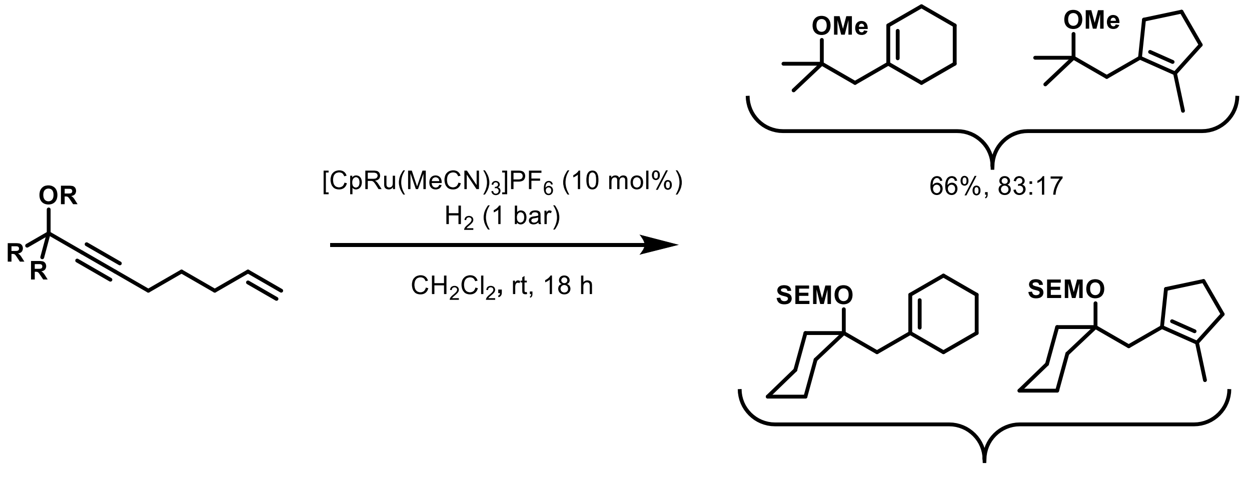

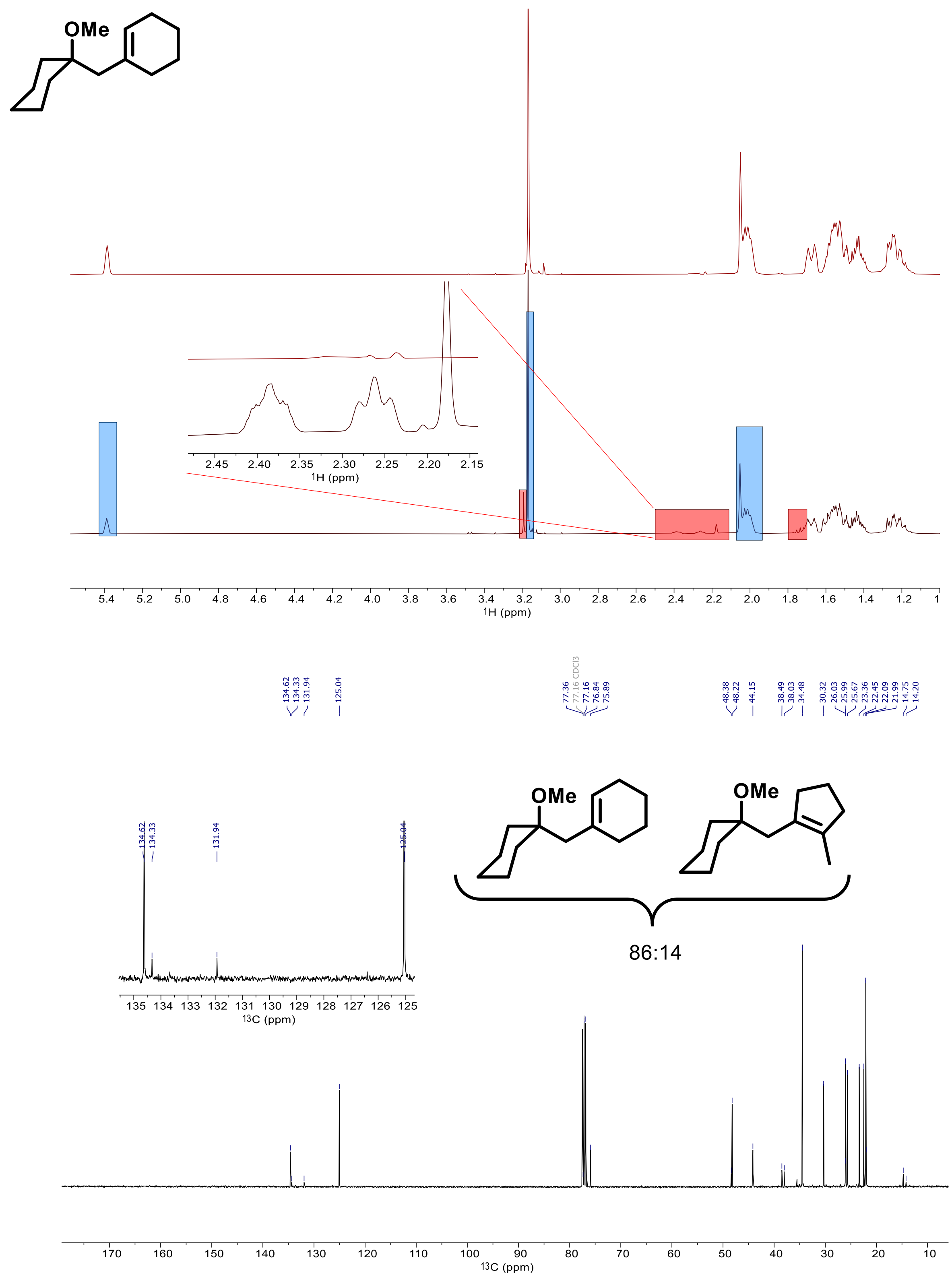

Figure S1. Top: Comparison of the ^{1}H NMR spectrum of authentic **12**[8] prepared according to a literature procedure with ^{1}H NMR spectrum of the crude reaction mixture containing **12** and **4d** (86:14); bottom: ^{13}C NMR of the same crude product

PHIP NMR Studies.

General Procedure. Triethyl((1-(prop-1-yn-1-yl)cyclohexyl)oxy)silane (**13**, 15 mg, 0.098 mmol) was added to a solution of the corresponding [Cp^{x}Ru] complex (5 mol%) in CD_2Cl_2 (0.2 M). The solution was transferred into a pressure NMR tube (5 mm medium wall precision pressure/vacuum valve NMR sample tube, Wilmad-LabGlass), which was tightly closed and then taken out of the glovebox. The tube was connected to the parahydrogen (pH$_2$) generator and all tubings were evacuated and backfilled with pH$_2$ three times.The pressure was then increased to 5 bar and the valve was opened to fill the tube with pH$_2$ to a total pressure of ca. 6 bar [the insert of the monitor of the pH$_2$ generator shows the pressure in barg (pressure above atmosphere)]. After closing the valve, the tube was shaken and inserted into the NMR magnet.

We have previously communicated that electron-deficient Cp^{x}Ru complexes are particularly good catalysts for *gem*-hydrogenation.[7] This notion is corroborated by the better signal-to-noise ratio and the narrower line width in the OPSY NMR of complex **14a** formed from [Cp^{COOMe}Ru(MeCN)$_3$]PF$_6$ (**C5**). The broad signals in the OPSY NMR of complex **14b** derived from [CpRu(MeCN)$_3$]PF$_6$ (**C3**) suggest that the resulting carbene intermediate is relatively short-lived.

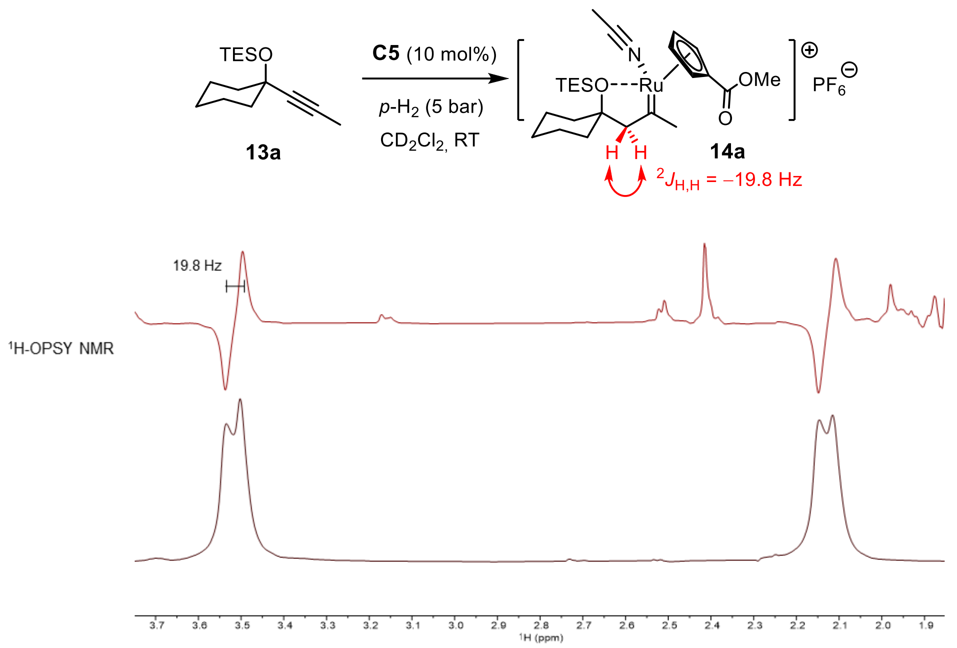

Figure S2. Methylene region of the PHIP NMR and OPSY NMR spectra of complex **14a** formed from compound **13a** and catalyst **C5**; the upper spectrum was obtained after a $\pi/4$ pulse (PASADENA signals), the lower spectrum with an OPSY filter prior to acquisition of the FID

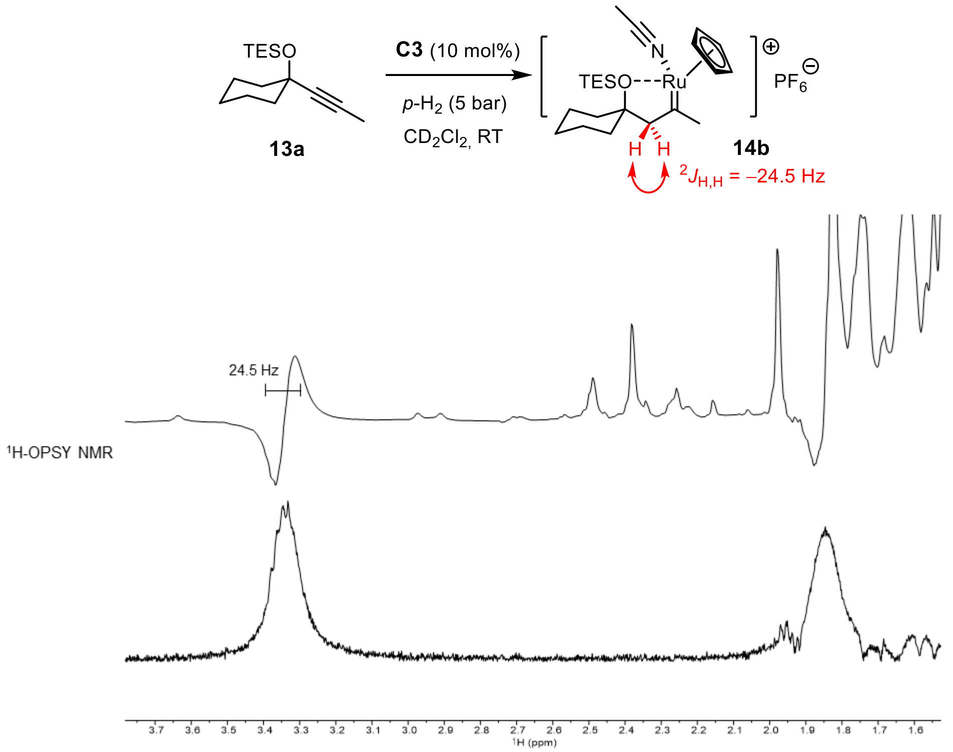

Figure S3. Methylene region of the PHIP NMR and OPSY NMR spectra of complex **14b** formed from compound **13a** and catalyst **C3**; the upper spectrum was obtained after a $\pi/4$ pulse (PASADENA signals), the lower spectrum with an OPSY filter prior to acquisition of the FID

Deuterium Labelling Study

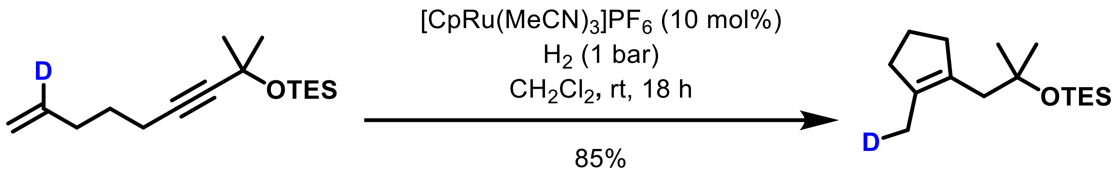

91% D **89% D**

Analyses of the degree of deuteration of the starting material and the product are provided below. They show an essentially quantitative migration of the deuterium label from the internal vinylic position to the terminal site.

Triethyl((2-methyl-1-(2-(methyl-d)cyclopent-1-en-1-yl)propan-2-yl)oxy)silane ([D]-4b).

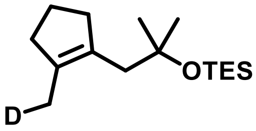

According to the Representative Procedure outlined above from substrate **[D]-1b** (46 mg, 85%). **^{1}H NMR** (400 MHz, $CDCl_3$) δ 2.44 – 2.34 (m, 2H), 2.31 – 2.23 (m, 2H), 2.20 (s, 2H), 1.72 (dq, J = 8.2, 7.4 Hz, 2H), 1.60 – 1.57 (m, 2H), 1.19 (s, 6H), 0.95 (t, J = 7.9 Hz, 9H), 0.57 (q, J = 7.9 Hz, 6H). **^{13}C NMR** (101 MHz, $CDCl_3$) δ 134.0, 133.2, 75.1, 44.0, 38.5, 38.5, 30.4, 22.3, 14.7 – 14.3 (t, J = 19.6 Hz), 7.2, 6.9. **IR** (Microscope, cm^{-1}): 2953, 2910, 2876, 1221, 1172, 1153, 1037, 1008, 740, 719. **HRMS** (ESI) for $C_{16}H_{31}DOSiNa$ [M+Na]: calcd. 292.21774, found 292.21738.

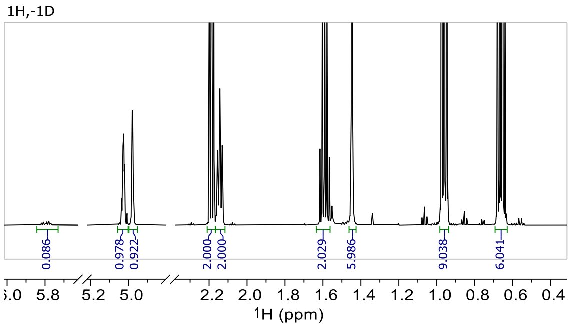

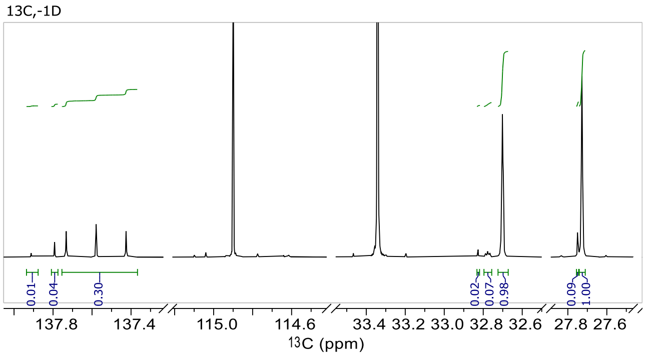

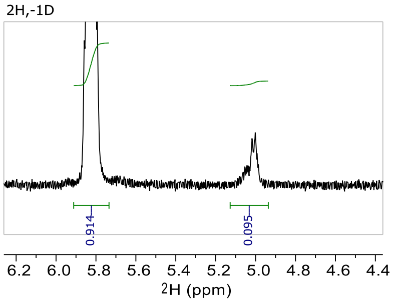

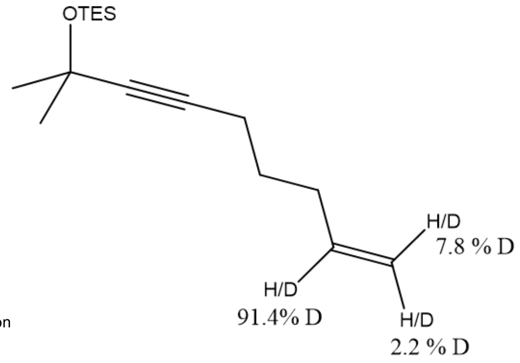

The deuteration grade was determined by ^{1}H NMR with a long relaxation delay (d1=20s)
Due to the presence of multiple isotopomers, it was difficult to extract the deuteration grade by ^{13}C NMR.
The relative data from ^{2}H NMR matches the 1H data +- 1%.

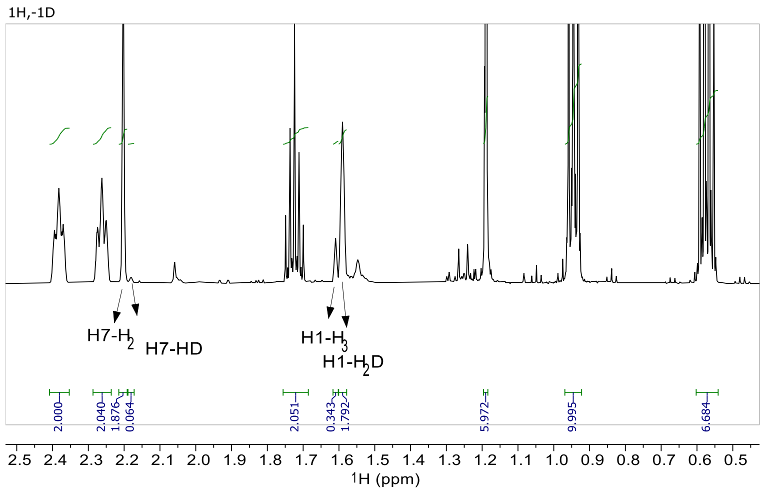

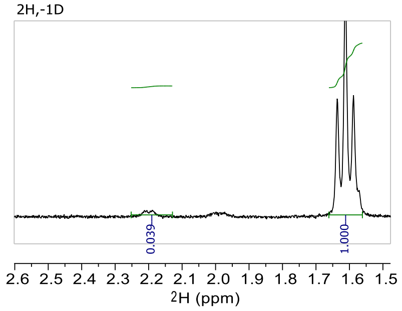

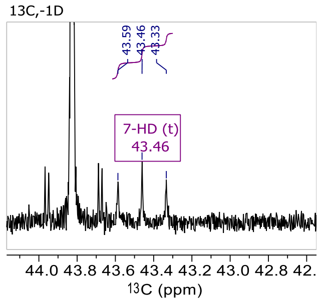

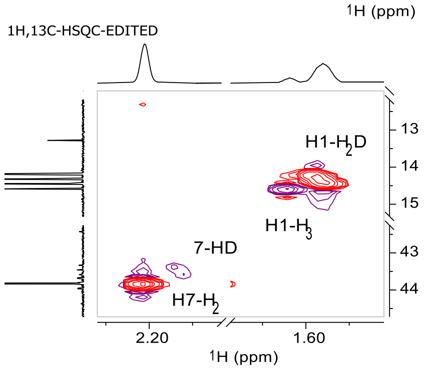

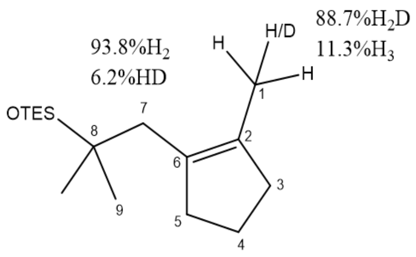

The deuteration grade was determined by 1H NMR with a long relaxation delay (d1=20s)
Due to the presence of multiple isotopomers, it was difficult to extract the deuteration grade by 13C NMR.
The relative data from 2H NMR matches the 1H data +- 3%.

Ligand Effects in Hydrogenative Rearrangements

Because *gem*-hydrogenation of alkynes is mechanistically intertwined with *trans*-hydrogenation, selectivity issues can arise. Whilst *trans*-hydrogenation was rarely observed in the hydrogenative cycloisomerization, it did become a notable side reaction in the hydrogenative sigmatropic rearrangements when [CpRu(MeCN)$_3$]PF$_6$ (**C3**) was used as the catalyst. Previous work in the group had established that more electron-deficient Cp^{X}Ru catalysts can suppress the *trans*-hydrogenation channel.[7]

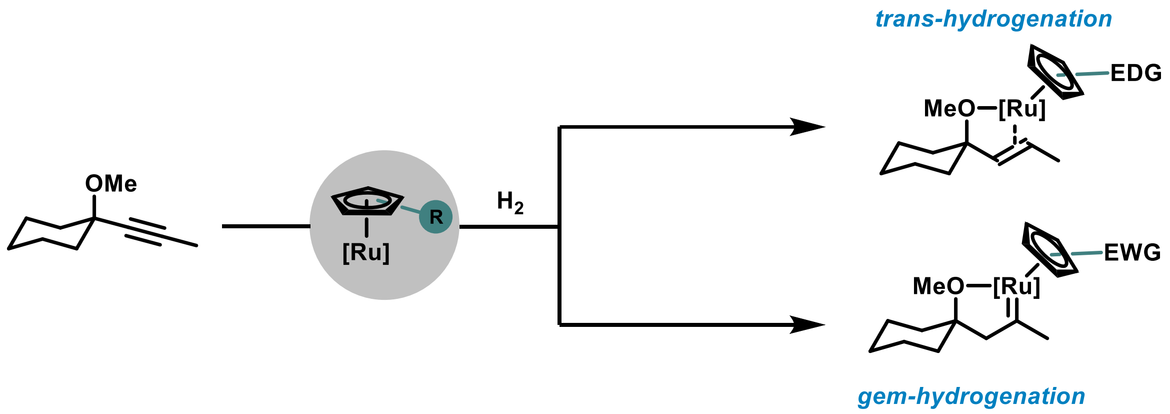

In line with this previous conclusion, it was found that the use of [Cp^{COOMe}Ru(MeCN)$_3$]PF$_6$ (**C5**) as the catalyst resulted in consistently better *gem*-hydrogenation/*trans*-hydrogenation ratios than the parent complex **C3**. The NMR spectra shown below are representative.

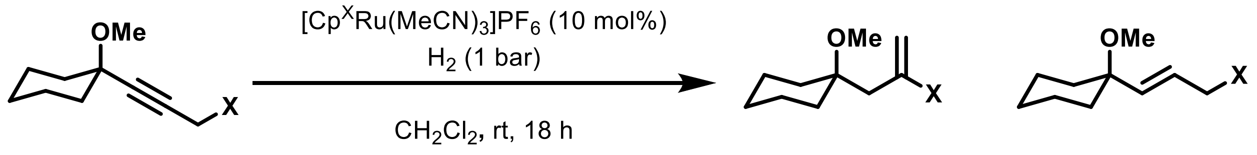

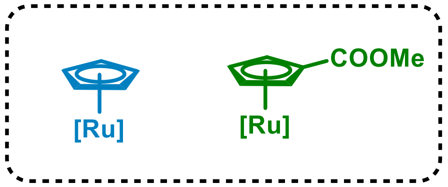

X = OC(O)OMe	90	:	10
X = OC(O)OMe	96	:	4
X = OC(O)*t*Bu	92	:	8
X = OC(O)*t*Bu	96	:	4
X = OC(O)N(*i*Pr)$_2$	88	:	12
X = OC(O)N(*i*Pr)$_2$	95	:	5

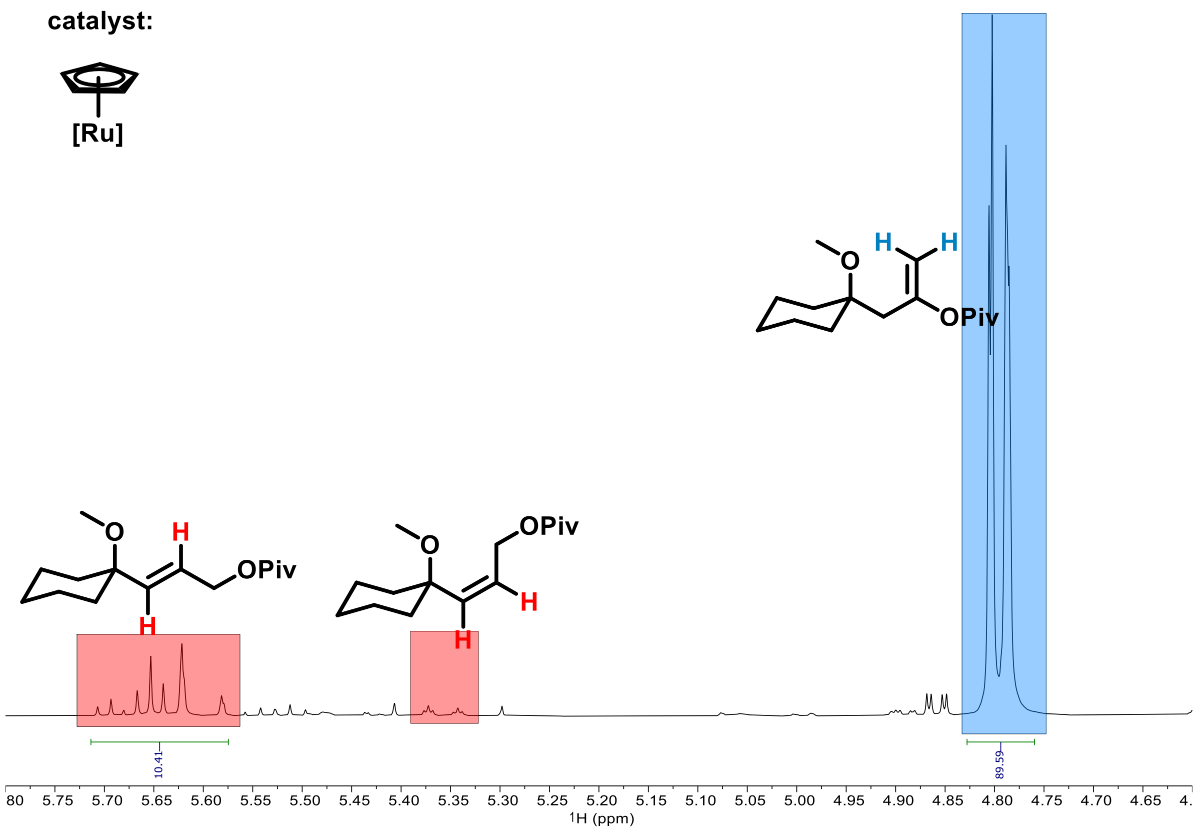
catalyst:
[Ru]
H
H
O
OPiv
H
OPiv
H
O
OPiv
H
H
10.41
89.59
80 5.75 5.70 5.65 5.60 5.55 5.50 5.45 5.40 5.35 5.30 5.25 5.20 5.15 5.10 5.05 5.00 4.95 4.90 4.85 4.80 4.75 4.70 4.65 4.
1H (ppm)

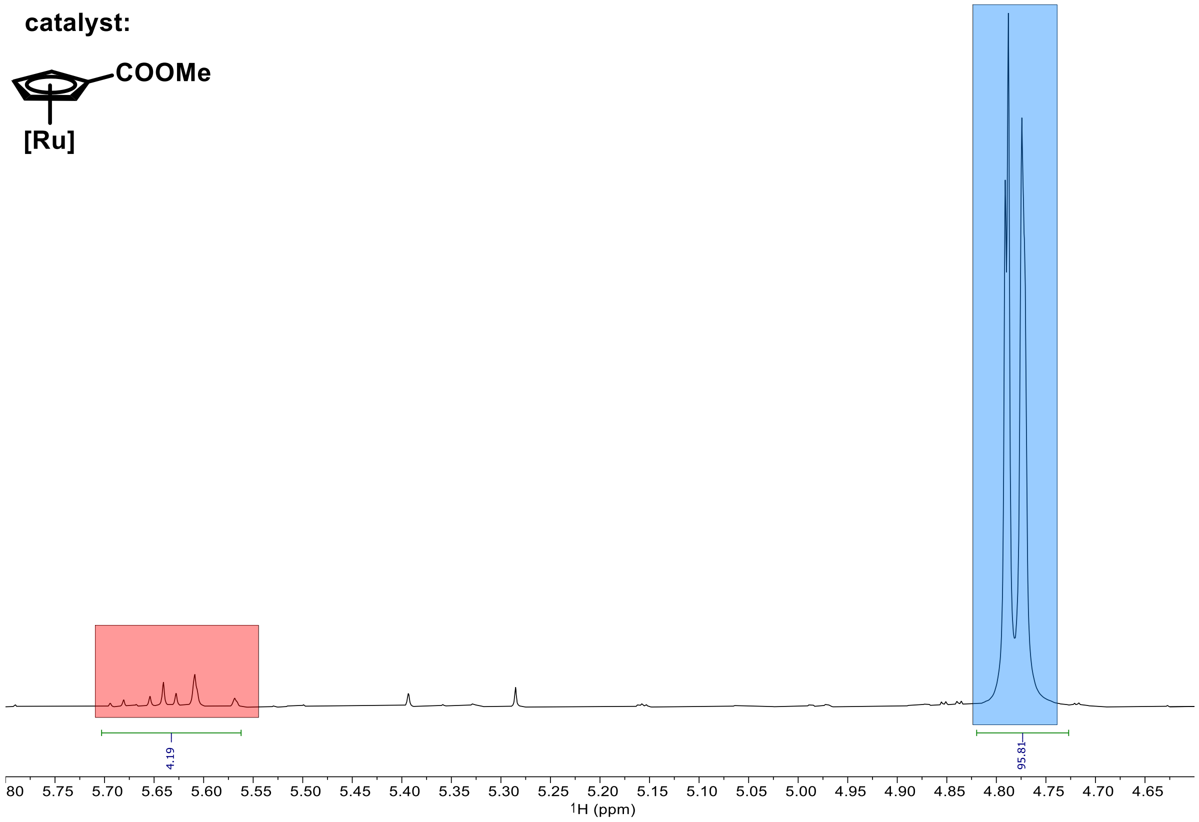
catalyst:
COOMe
[Ru]
4.19
95.81
80 5.75 5.70 5.65 5.60 5.55 5.50 5.45 5.40 5.35 5.30 5.25 5.20 5.15 5.10 5.05 5.00 4.95 4.90 4.85 4.80 4.75 4.70 4.65
1H (ppm)

Hydrogenative Rearrangement and Cycloisomerization Reactions: PHIP NMR Studies.
At the outset of this project, we surmised that hydrogenative rearrangements could proceed via two distinct pathways. On the one hand, *gem*-hydrogenation could generate competent ruthenium carbenes. Alternatively, π-acid activation of the alkyne would entail nucleophilic attack of the propargylic substituent to yield a vinyl metal species, which could further evolve into a vinyl carbene; hydrogenolytic cleavage would then release the product and close the catalytic cycle. It is important to note that a closely related reactivity mode was observed in one of our previous publications.[9]

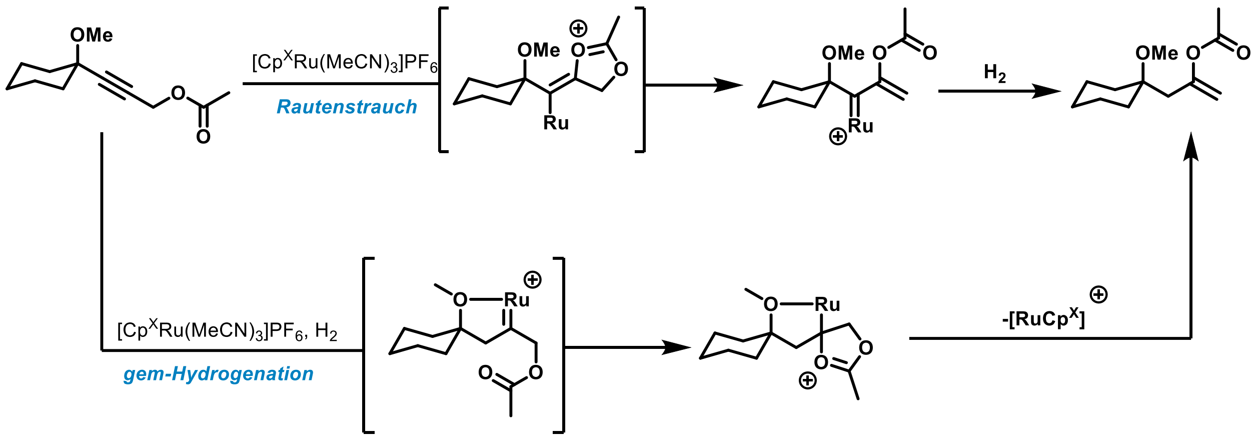

In order to clarify which pathway is operative, PHIP NMR was again used as the analytical tool. To this end, four different substrates were hydrogenated using parahydrogen (pH_2) under standard conditions and the reaction course monitored by PHIP NMR. The characteristic hyperpolarized signals of the methylene group formed by alkyne *gem*-hydrogenation was clearly observed in all four cases, which indicate formation of the corresponding ruthenium carbenes.

Representative Procedure. Substrate **15a**, **15b**, **15c**, or **15d** (0.098 mmol, 1 equiv) was added under Ar to a solution of $[Cp^{COOMe}Ru(MeCN)_3]PF_6$ (**C5**, 10 mol%) in CD_2Cl_2 (0.2 M) in a Schlenk tube. The solution was transferred into a pressure NMR tube (5 mm medium wall precision pressure/vacuum valve NMR sample tube, Wilmad-LabGlass), which was tightly closed and then taken out of the glovebox. The tube was connected to the pH_2 generator and all tubings were evacuated and backfilled with pH_2 three times. The pressure was then increased to 5 bar and the valve was opened to fill the tube with pH_2 to a total pressure of ca. 6 bar [the insert of the monitor of the parahydrogen generator shows the pressure in barg (pressure above atmosphere)]. After closing the valve, the tube was shaken and inserted into the NMR magnet.

As additional control experiments, ^{1}H NMR spectra were recorded before the NMR tubes were pressurized with hydrogen. If a π-acid catalyzed Rautenstrauch rearrangement were operative, one would expect signals of vinyl carbene intermediates, which could not be detected. Taken together, these experimental data suggest that *gem*-hydrogenation with formation of ruthenium carbenes is responsible for the observed [2,3]-sigmatropic rearrangements.

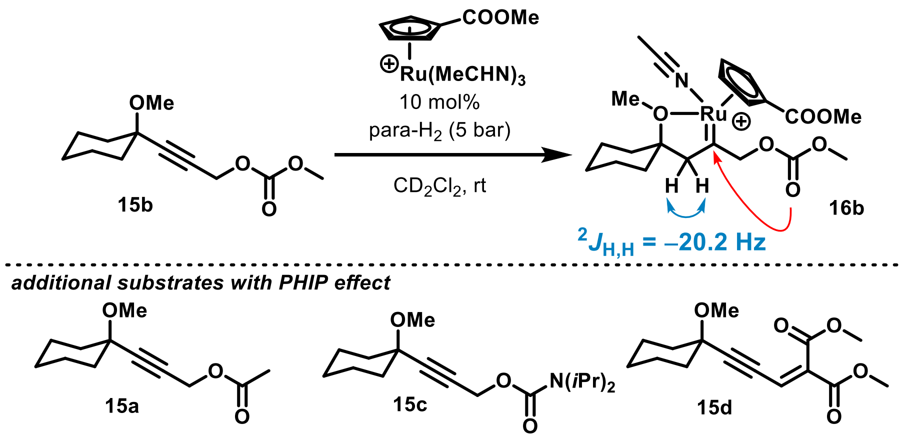

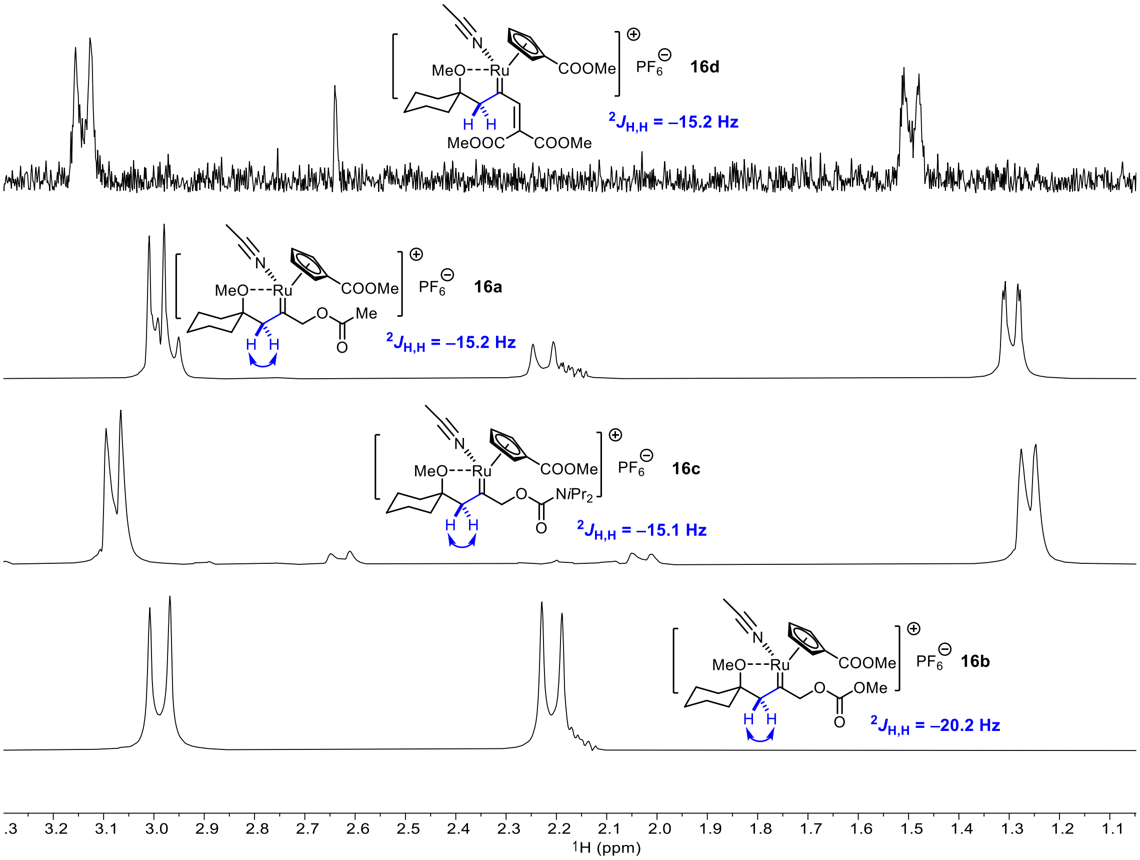

Figure S4. Relevant excerpts of the OPSY NMR spectra recorded upon catalytic hydrogenation of substrates of type **15** with pH$_2$

Substrates

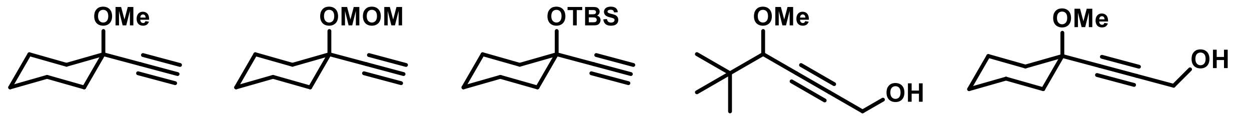

S2 **S3** **S4** **S5** **S6**

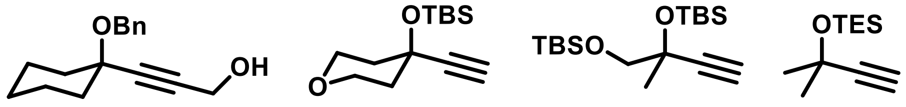

S7 **S8** **S9** **S10**

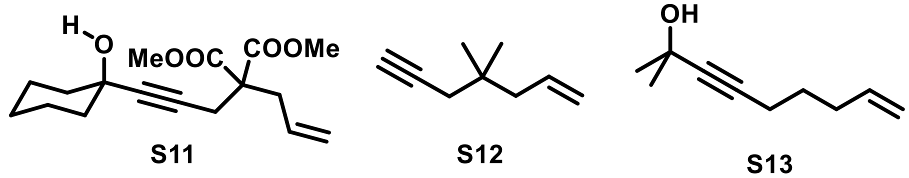

S11 **S12** **S13**

Compounds **S1**[10], **S2**[9], **S3**[9], **S4**[11], **S5**[12], **S6**[13], **S8**[14], **S9**[15], **S10**[12], **S11**[16], **S12**[17], **S13**[12] were prepared according to literature procedures.

2-Deutero-5-bromopent-1-ene (S14). Prepared according to a method described by Hoveyda

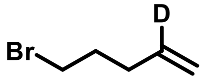

and coworkers:[18] Neat DIBAL-H (4.95 mL, 27.8 mmol) was added dropwise to a solution of 1,3-bis(diphenylphosphino)-propane nickel(II) chloride (347 mg, 0.64 mmol) in THF (20 mL) at ambient temperature. The mixture was cooled to 0°C before a solution of 4-pentyn-1-ol (1.00 g, 11.8 mmol) in THF (3 mL) was added. After stirring the solution for 3.5 h, D_2O (5 mL) was added and the mixture was stirred for another hour. The resulting suspension was diluted with diethyl ether (30 mL) and an aqueous solution of Rochelle salt was added. The phases were separated and the aqueous phase was extracted with diethyl ether (3 x 30 mL). The combined organic phases were washed with brine and dried over Na_2SO_4. All volatile materials were removed *in vacuo* and the resulting alcohol was directly used in the next step.

Bromine (0.71 mL, 13.8 mmol) was added dropwise at 0°C to a solution of triphenylphosphine in acetonitrile (20 mL) and diethyl ether (40 mL) and the resulting mixture was stirred for 20 min at this temperature. Imidazole (1.0 g, 14.8 mmol) was then added in portions before addition of the crude alcohol. The resulting slurry was vigorously stirred for 30 min at 0°C and for 2 h at ambient temperature. The reaction was quenched with sat. aq. $NaHCO_3$ and the aqueous phase was extracted with pentane (3 x 30 mL). The combined organic layers were washed with brine and dried over Na_2SO_4. Careful evaporation (500 mbar, 30 °C bath temperature) yielded a white slurry that was suspended in pentane and filtered through a pad of silica. The filtrate was evaporated

(500 mbar, 30 °C bath temperature) to yield the title compound (1.10 g, 68%) as a colorless, *very volatile* liquid which was used without further purification.

***tert*-Butyl(((1s,4s)-1-ethynyl-4-(trifluoromethyl)cyclohexyl)oxy)dimethylsilane (S15).**

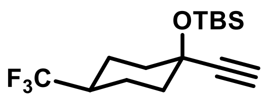

Ethynylmagnesium bromide (0.5 M in THF, 12.9 mL) was added dropwise to a solution of 4-trifluoromethylcyclohexanone (830 mg, 4.99 mmol) in THF (15 mL) at −78 °C. The mixture was stirred for 1 h at −78 °C and for 2 h at ambient temperature. sat. NH_4Cl solution was introduced and the phases were separated. The aqueous phase was extracted with *tert*-butyl methyl ether (3 x 20 mL) and the combined organic layers were washed with brine and dried over Na_2SO_4. All volatile materials were removed *in vacuo* and the residue was directly used in the next step without further purification.

2,6-Lutidine (0.26 mL, 2.28 mmol) and *tert*-butyldimethylsilyl triflate (0.39 mL, 1.71 mmol) were added to a solution of the crude alcohol (220 mg, 1.14 mmol) in methylene chloride (10 mL) at −78°C. The solution was allowed to reach ambient temperature within 15 h. Water (10 mL) was added and the phases were separated. The aqueous phase was extracted with methylene chloride (3 x 10 mL) and the combined organic layers were washed with brine and dried over Na_2SO_4. All volatile materials were removed *in vacuo* and the residue was purified by flash chromatography (SiO_2, pentane:*tert*-butyl methyl ether, 100:1) to provide the title compound as a colorless liquid (62 mg, 18%). **^{1}H NMR** (400 MHz, $CDCl_3$) δ 2.54 (s, 1H), 2.10 – 2.02 (m, 2H), 2.01 – 1.95 (m, 1H), 1.91 – 1.82 (m, 2H), 1.76 – 1.63 (m, 2H), 1.56 – 1.44 (m, 2H), 0.86 (s, 9H), 0.18 (s, 6H). **^{13}C NMR** (101 MHz, $CDCl_3$) δ 129.1 (q, *J* = 278.7 Hz), 86.3, 74.9, 69.4, 40.9 (q, *J* = 26.7 Hz), 39.6, 25.1, 22.5 (q, *J* = 2.6 Hz), 18.0, -2.6. **^{19}F NMR** (282 MHz, $CDCl_3$) δ -73.22. **IR** (Microscope, cm^{-1}): 2955, 2349, 2332, 2155, 2068, 1961, 1282, 1177, 1091, 1106, 865, 778. 440. **HRMS** (ESI) for $C_{15}H_{26}OSiF_3$ [M+H]: calcd. 307.16995, found 307.17007.

***tert*-Butyl((2-(4-fluorophenyl)3-butyn-2-yl)oxy)dimethylsilane (S16).** 2,6-Lutidine (0.66 mL,

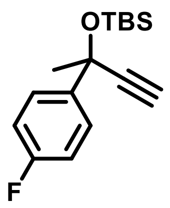

5.72 mmol) and *tert*-butyldimethylsilyl triflate (0.98 mL, 4.29 mmol) were added to a solution of 2-(4-fluorophenyl)-3-butyn-2-ol (522 mg, 2.86 mmol) in methylene chloride (20 mL) at −78°C. The solution was allowed to reach ambient temperature within 15 h. Water (20 mL) was introduced and the phases were separated. The aqueous phase was extracted with methylene chloride (3 x 20 mL) and the combined organic layers were washed with brine and dried over Na_2SO_4. All volatile materials were removed *in vacuo* and the residue was used in the next step without purification.

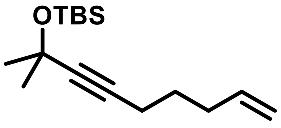

1a

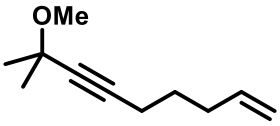

1d

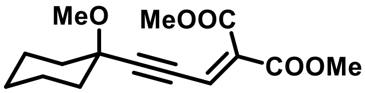

15d

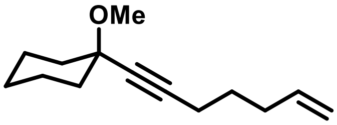

S17

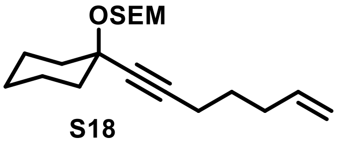

S18

Compounds **1a**,[12] **1d**,[12] **15d**,[9] **S17**,[12] and **S18**[12] were prepared as previously described in the literature.

Synthesis of Propargylic Enynes by C-Alkylation. Triethyl((2-methylnon-8-en-3-yn-2-yl)oxy)silane (1b).

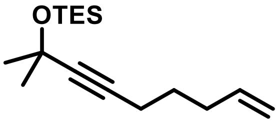

n-Butyllithium (1.6 M in hexanes, 2.24 mL, 1.4 equiv) was slowly added to a solution of the alkyne **S10** (508 mg, 2.56 mmol) in THF (40 mL) at 0 °C. The mixture was stirred for 30 min at 0 °C before a solution of 5-bromopent-1-ene (531 mg, 3.59 mmol) and DMPU (1.95 g, 12.52 mmol) in THF (5 mL) was added. Stirring was continued at room temperature for 15 h before sat. NH_4Cl sol. and *tert*-butyl methyl ether were introduced. The layers were separated, the aqueous phase was extracted with *tert*-butyl methyl ether (3x), and the combined organic layers were washed with brine and dried over Na_2SO_4. The solvent was removed *in vacuo* and the residue was purified by flash chromatography to give the title compound as a colorless liquid (667 mg, 98%). **^{1}H NMR** (400 MHz, $CDCl_3$) δ 5.80 (ddt, *J* = 17.0, 10.1, 6.7 Hz, 1H), 5.08 – 5.00 (m, 1H), 4.98 (ddt, *J* = 10.2, 2.2, 1.2 Hz, 1H), 2.25 – 2.08 (m, 4H), 1.59 (p, *J* = 7.3 Hz, 2H), 1.45 (s, 6H), 1.04 – 0.88 (m, 9H), 0.66 (qd, *J* = 7.8, 0.8 Hz, 6H). **^{13}C NMR** (101 MHz, $CDCl_3$) δ 138.1, 115.2, 86.0, 82.4, 66.4, 33.5, 33.0, 27.9, 18.2, 7.1, 6.1. **IR** (Microscope, cm^{-1}): 2952, 2936, 2876, 1244, 1158, 1035, 1005, 912, 740, 724, 672. **HRMS** (CI) for $C_{16}H_{31}OSi$ [M+H]: calcd. 267.21387, found 267.21373.

Triethyl((2-methylnon-8-en-3-yn-2-yl-8-deutero)oxy)silane ([D]-1b). Prepared analogously

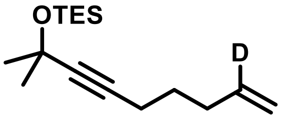

from alkyne **S10** (500 mg, 2.52 mmol) and 2-deutero-5-bromopent-1-ene (**S14**, 620 mg, 3.78 mmol) as a colorless liquid (620 mg, 92%). **^{1}H NMR** (400 MHz, $CDCl_3$) δ 5.05 – 5.01 (m, 1H), 4.98 (dp, *J* = 2.6, 1.4 Hz, 1H), 2.24 – 2.11 (m, 4H), 1.59 (p, *J* = 7.3 Hz, 2H), 1.45 (s, 6H), 1.01 – 0.92 (m, 9H), 0.66 (qd, *J* = 7.8, 0.8 Hz, 6H). **^{13}C NMR** (101 MHz, $CDCl_3$) δ 137.7 (t, *J* = 23.3 Hz), 115.0, 86.0, 82.4, 66.4, 33.5, 32.8, 27.9, 18.2, 7.1, 6.1. **IR** (Microscope, cm^{-1}): 2952, 2936, 2876, 1244, 1158, 1035, 1005, 912, 740, 724, 672. **HRMS** (ESI) for $C_{16}H_{30}OSiD$ [M+H]: calcd. 268.22015, found 268.21991.

Triisopropyl((2-methylnon-8-en-3-yn-2-yl)oxy)silane (1c). 2,6-Lutidine (0.21 mL, 1.76 mmol) and triisopropylsilyl triflate (0.28 mL, 1.05 mmol) were added to a solution of alcohol **S13** (134 mg, 0.88 mmol) in methylene chloride (3 mL) at −15 °C. After stirring at this temperature for 30 min, water (10 mL) was added and the phases were separated. The aqueous layer was extracted with methylene chloride (3 x 5 mL) and the combined organic phases were washed with brine and dried over Na_2SO_4. All volatile materials were removed *in vacuo* and the residue was purified by flash chromatography (SiO_2, hexanes:ethyl acetate, 100:1) to provide the title compound as a colorless oil (240 mg, 88%). **^{1}H NMR** (400 MHz, $CDCl_3$) δ 5.79 (ddt, *J* = 16.9, 10.2, 6.7 Hz, 1H), 5.08 – 4.93 (m, 2H), 2.22 – 2.08 (m, 4H), 1.57 (p, *J* = 7.2 Hz, 2H), 1.48 (s, 6H), 1.18 – 1.04 (m, 21H). **^{13}C NMR** (101 MHz, $CDCl_3$) δ 138.1, 115.1, 86.2, 82.0, 66.3, 33.6, 33.0, 27.8, 18.5, 18.2, 13.1. **IR** (Microscope, cm^{-1}): 2941, 2865, 1463, 1377, 1358, 1245, 1158, 1047, 882, 678, 657. **HRMS** (ESI) for $C_{19}H_{36}OSiNa$ [M+Na]: calcd. 331.24276, found 331.24281.

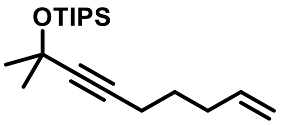

***tert*-Butyl((4-(hept-6-en-1-yn-1-yl)tetrahydro-2H-pyran-4-yl)oxy)dimethylsilane (S19).** Prepared accordingly from alkyne **S8** (500 mg, 2.07 mmol); colorless oil (604 mg, 95%). **^{1}H NMR** (400 MHz, $CDCl_3$) δ 5.79 (ddt, *J* = 16.9, 10.2, 6.7 Hz, 1H), 5.09 – 4.95 (m, 2H), 3.89 – 3.77 (m, 2H), 3.65 (ddd, *J* = 11.2, 7.4, 3.4 Hz, 2H), 2.24 (t, *J* = 7.2 Hz, 2H), 2.16 (dtt, *J* = 8.0, 6.7, 1.4 Hz, 2H), 1.85 (dddd, *J* = 13.3, 6.8, 3.4, 1.2 Hz, 2H), 1.72 (dddd, *J* = 13.0, 7.1, 3.7, 0.8 Hz, 2H), 1.62 (p, *J* = 7.2 Hz, 2H), 0.89 (s, 9H), 0.16 (s, 6H). **^{13}C NMR** (101 MHz, $CDCl_3$) δ 137.9, 115.4, 85.6, 83.8, 66.5, 64.6, 41.6, 33.0, 27.9, 25.9, 18.2, 18.2, −2.7. **IR** (Microscope, cm^{-1}): 2952, 2928, 2856, 1253, 1150, 1103, 1008, 970, 832, 770, 732. **HRMS** (ESI) for $C_{18}H_{33}O_2SiNa$ [M+Na]: calcd. 309.22443, found 309.22410.

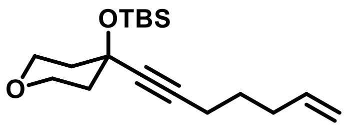

***tert*-Butyl(((1s,4s)-1-(hept-6-en-1-yn-1-yl)-4-(trifluoromethyl)cyclohexyl)oxy)dimethyl-silane (S20)**. Prepared analogously from alkyne **S15** (62 mg, 0.20 mmol) as a colorless oil (63 mg, 83%). **^{1}H NMR** (400 MHz, $CDCl_3$) δ 5.79 (ddt, *J* = 17.0, 10.2, 6.7 Hz, 1H), 5.11 – 4.95 (m, 2H), 2.24 (t, *J* = 7.1 Hz, 2H), 2.17 (dtt, *J* = 7.9, 6.7, 1.4 Hz, 2H), 2.02 – 1.93 (m, 3H), 1.88 – 1.79 (m, 2H), 1.73 – 1.56 (m, 4H), 1.46 (td, *J* = 13.0, 3.6 Hz, 2H), 0.86 (s, 9H), 0.16 (s, 6H). **^{13}C NMR** (101 MHz, $CDCl_3$) δ 137.9, 129.3 (q, *J* = 280.6 Hz), 115.4, 86.9, 82.8, 69.6, 41.0 (q, *J* = 26.6 Hz), 40.0, 32.9, 28.0, 25.8, 22.7 (q, *J* = 2.6 Hz), 18.1, 18.0, −2.6. **^{19}F NMR** (282 MHz, $CDCl_3$) δ -73.24 **IR** (Microscope, cm^{-1}): 2953, 1352, 1288, 1253, 1176, 1108, 837, 777. **HRMS** (CI) for $C_{20}H_{34}OSiF_3$: calcd. 375.23255, found 375.23255.

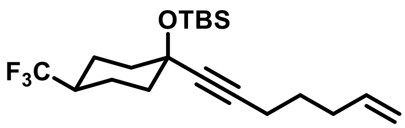

1-(4,4-Dimethylhept-6-en-1-yn-1-yl)cyclohexan-1-ol (S21). *n*-Butyllithium (1.6 M in hexanes, 3.9 mL) was added dropwise to a solution of alkyne **S12** (0.77 g, 6.28 mmol) in THF (20 mL) at −78 °C. The resulting yellow solution was stirred for 30 min at this temperature before cyclohexanone (0.59 mL, 5.71 mmol) was added. The mixture was stirred for 1 h at −78 °C and for 30 min at ambient temperature. sat. NH_4Cl solution was added and the phases were separated. The aqueous phase was extracted with *tert*-butyl methyl ether (3 x 20 mL) and the combined organic layers were washed with brine and dried over Na_2SO_4. All volatile materials were removed *in vacuo* and the residue was purified by flash chromatography (SiO_2, pentane:*tert*-butyl methyl ether, 5:1) to provide the title compound as a colorless oil (1.03 g, 82%). **^{1}H NMR** (400 MHz, $CDCl_3$) δ 5.80 (ddt, *J* = 16.2, 10.9, 7.5 Hz, 1H), 5.18 – 4.95 (m, 2H), 2.13 – 2.02 (m, 4H), 1.94 – 1.81 (m, 3H), 1.77 – 1.63 (m, 2H), 1.62 – 1.49 (m, 5H), 1.22 (bs, 1H), 0.96 (s, 6H). **^{13}C NMR** (101 MHz, $CDCl_3$) δ 135.2, 117.5, 85.8, 82.8, 69.1, 45.8, 42.1, 33.9, 27.1, 26.8, 25.4, 23.7. **IR** (Microscope, cm^{-1}): 2931, 2851, 1469, 1259, 1094, 1053, 998, 908, 877, 834, 769. **HRMS** (EI) for $C_{15}H_{24}O$: calcd. 220.18217, found 220.18214.

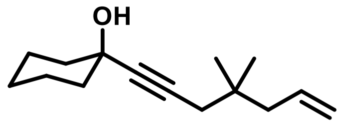

***tert*-Butyl((1-(4,4-dimethylhept-6-en-1-yn-1-yl)cyclohexyl)oxy)dimethylsilane (S22).** 2,6-Lutidine (0.36 mL, 3.10 mmol) and *tert*-butyldimethylsilyl triflate (0.53 mL, 2.32 mmol) were added to a solution of alcohol **S21** (342 mg, 1.55 mmol) in methylene chloride (8 mL) at −78 °C. The solution was allowed to reach ambient temperature within 15 h. Water (10 mL) was introduced and the phases were separated. The aqueous phase was extracted with methylene chloride (3 x 10 mL) and the combined organic layers were washed with brine and dried over Na_2SO_4. All volatile materials were removed *in vacuo* and the residue was purified by flash chromatography (SiO_2, pentane:*tert*-butyl methyl ether, 100:1) to give the title compound as a colorless liquid (440 mg, 85%). **^{1}H NMR** (400 MHz, $CDCl_3$) δ 5.94 – 5.73 (m, 1H), 5.11 – 4.97 (m, 2H), 2.14 – 2.02 (m, 4H), 1.78 (dt, *J* = 10.1, 3.5 Hz, 2H), 1.71 – 1.39 (m, 8H), 0.96 (s, 6H), 0.87 (s, 9H), 0.15 (s, 6H). **^{13}C NMR** (101 MHz, $CDCl_3$) δ 135.3, 117.4, 86.6, 83.0, 69.6, 45.9, 41.7, 34.0, 31.8, 26.8, 26.0, 25.5, 23.1, 18.2, -2.7. **IR** (Microscope, cm^{-1}): 2930, 2856, 1463, 1250, 1095, 1053, 1000, 914, 876, 834, 773, 678. **HRMS** (ESI) for $C_{21}H_{38}OSiNa$ [M+Na]: calcd. 357.25841, found 357.25839.

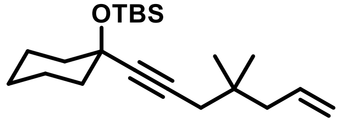

Dimethyl-2-allyl-2-(3-(1-((*tert*-butyldimethylsilyl)oxy)cyclohexyl)prop-2-yn-1-yl)malonate (S23).

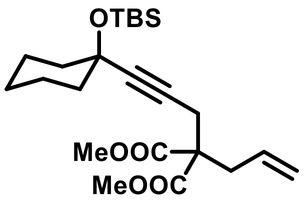

2,6-Lutidine (0.41 mL, 3.58 mmol) and *tert*-butyldimethylsilyl triflate (0.61 mL, 2.68 mmol) were added to a solution of alcohol **S11** (552 mg, 1.79 mmol) in methylene chloride (10 mL) at −78°C. The solution was allowed to reach ambient temperature within 15 h. Water (10 mL) was added and the phases were separated. The aqueous layer was extracted with methylene chloride (3 x 10 mL) and the combined organic phases were washed with brine and dried over Na_2SO_4. All volatile materials were removed *in vacuo* and the residue was purified by flash chromatography (SiO_2, pentane:*tert*-butyl methyl ether, 25:1) to give the title compound as a colorless liquid (608 mg, 80%). **^{1}H NMR** (400 MHz, $CDCl_3$) δ 5.63 (ddt, *J* = 17.3, 9.7, 7.4 Hz, 1H), 5.23 – 5.05 (m, 2H), 3.73 (s, 6H), 2.89 – 2.71 (m, 4H), 1.73 (dt, *J* = 8.6, 4.9 Hz, 2H), 1.61 – 1.39 (m, 8H), 0.86 (s, 9H), 0.12 (s, 6H). **^{13}C NMR** (101 MHz, $CDCl_3$) δ 170.4, 132.0, 119.9, 88.0, 79.3, 69.5, 57.2, 52.8, 41.4, 36.8, 25.9, 25.4, 23.1, 23.0, 18.2, -2.7. **IR** (Microscope, cm^{-1}): 2932, 2856, 1739, 1437, 1287, 1249, 1215, 1097, 1000, 875, 835, 774, 669. **HRMS** (ESI) for $C_{23}H_{38}O_5SiNa$ [M+Na]: calcd. 445.23807, found 445.23779.

***tert*-Butyl((2-(4-fluorophenyl)non-8-en-3-yn-2-yl)oxy)dimethylsilane (S24).** Prepared

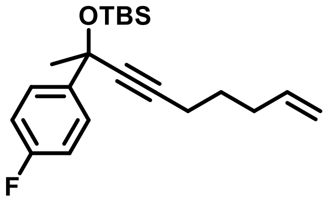

analogously from the crude TBS-protected alkyne **S16** (685 mg, 2.46 mmol) as a colorless oil (713 mg, 72% over two steps). **^{1}H NMR** (400 MHz, $CDCl_3$) δ 7.63 – 7.50 (m, 2H), 7.09 – 6.91 (m, 2H), 5.81 (ddt, *J* = 18.4, 10.0, 6.8 Hz, 1H), 5.21 – 4.94 (m, 2H), 2.34 – 2.25 (m, 2H), 2.24 – 2.14 (m, 2H), 1.73 – 1.61 (m, 5H), 0.92 (s, *J* = 1.7 Hz, 9H), 0.20 (s, 3H), 0.01 (s, 3H). **^{13}C NMR** (101 MHz, $CDCl_3$) δ 161.9 (d, *J* = 244.5 Hz), 143.7 (d, *J* = 3.0 Hz), 137.9, 126.9 (d, *J* = 8.0 Hz), 115.4, 114.7, 114.4, 86.2, 84.3, 70.8, 36.3, 33.1, 27.9, 26.0, 18.3, −2.7, −3.1. **IR** (Microscope, cm^{-1}): 2930, 1505, 1230, 1156, 1089, 991, 913, 831, 776, 577. **HRMS** (ESI) for $C_{21}H_{31}OSiF_3Na$ [M+Na]: calcd. 369.20204, found 369.20200.

5-(Hept-6-en-1-yn-1-yl)-2,2,3,3,5,8,8,9,9-nonamethyl-4,7-dioxa-3,8-disiladecane (S25).

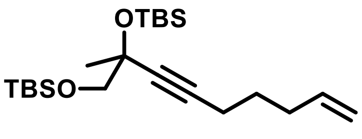

Prepared analogously from alkyne **S9** (120 mg, 0.36 mmol), colorless oil (90 mg, 63%). **^{1}H NMR** (400 MHz, $CDCl_3$) δ 5.79 (ddt, *J* = 17.0, 10.2, 6.7 Hz, 1H), 5.03 (dq, *J* = 17.1, 1.6 Hz, 1H), 4.98 (ddt, *J* = 10.2, 2.2, 1.2 Hz, 1H), 3.49 (d, *J* = 9.4 Hz, 1H), 3.41 (d, *J* = 9.3 Hz, 1H), 2.22 – 2.11 (m, 4H), 1.59 (p, *J* = 7.3 Hz, 2H), 1.37 (s, 3H), 0.90 (s, 9H), 0.86 (s, 9H), 0.16 (s, 3H), 0.14 (s, 3H), 0.05 (d, *J* = 1.1 Hz, 6H). **^{13}C NMR** (101 MHz, $CDCl_3$) δ 138.1, 115.2, 84.1, 83.9, 72.0, 70.1, 33.0, 27.9, 26.0, 25.9, 18.4, 18.2, 18.1, –2.7, –2.8, –5.1, –5.2. **IR** (Microscope, cm^{-1}): 1929, 2857, 1472,

1252, 1112, 1028, 829, 774. **HRMS** (ESI) for $C_{22}H_{44}O_2Si_2Na$ [M+Na]: calcd. 419.27721, found 419.27740.

3-(1-(Methoxymethoxy)cyclohexyl)prop-2-yn-1-ol (S26). *n*-Butyllithium (1.3 mL, 1.6 M in hexanes, 2.0 mmol) was added dropwise to a solution of 1-ethynyl-1-(methoxymethoxy)cyclohexane (**S3**, 0.34 g, 2.0 mmol) in THF (6 mL) at 0 °C. The mixture was stirred at 0 °C for 30 min. Freshly ground paraformaldehyde (72 mg, 2.4 mmol) was added in one portion at 0 °C and stirring continued at 0 °C for 15 min. The mixture was then stirred for 14 h at ambient temperature before the reaction was quenched with saturated aq. NH_4Cl (10 mL). The aqueous layer was extracted with *tert*-butyl methyl ether (3 × 15 mL) and the combined organic layers were washed with brine (15 mL), dried over Na_2SO_4, and concentrated under reduced pressure. The residue was purified by flash chromatography (SiO_2, hexanes/*tert*-butyl methyl ether, 1:1) to give the desired product as a colorless oil (0.38 g, 96%). **^{1}H NMR** (400 MHz, $CDCl_3$) δ 4.92 (s, 2H), 4.33 (s, 2H), 3.41 (s, 3H), 2.00 – 1.88 (m, 2H), 1.78 – 1.61 (m, 5H), 1.60 – 1.47 (m, 3H), 1.36 – 1.21 (m, 1H). **^{13}C NMR** (101 MHz, $CDCl_3$) δ 92.9, 86.8, 85.0, 74.9, 55.9, 51.3, 38.6, 25.4, 23.1. **IR** (Microscope, cm^{-1}): 3430, 2934, 2858, 1448, 1407, 1359, 1294, 1258, 1224, 1173, 1149, 1096, 1070, 1025, 928, 907, 606, 528. **HRMS** (ESI) for $C_{11}H_{18}O_3Na$ [M+Na]+: calcd. 221.1148, found 221.1147.

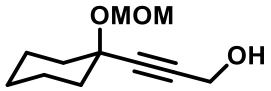

3-(1-((*tert*-Butyldimethylsilyl)oxy)cyclohexyl)prop-2-yn-1-ol (S27). Prepared analogously from *tert*-butyl((1-ethynylcyclo-hexyl)oxy)dimethylsilane (**S4**, 0.48 g, 2.0 mmol); colorless oil (0.50 g, 94%). **^{1}H NMR** (400 MHz, $CDCl_3$) δ 4.31 (d, J = 3.2 Hz, 2H), 1.76 (ddd, J = 10.9, 7.7, 3.7 Hz, 2H), 1.71 – 1.55 (m, 3H), 1.55 – 1.24 (m, 6H), 0.88 (s, 9H), 0.16 (s, 6H). **^{13}C NMR** (101 MHz, $CDCl_3$) δ 90.8, 82.6, 69.3, 51.5, 41.1, 26.0, 25.4, 22.8, 18.3, −2.6. **IR** (Microscope, cm^{-1}): 3315, 2933, 2895, 2856, 1472, 1462, 1446, 1388, 1359, 1290, 1253, 1173, 1135, 1099, 1054, 1022, 1002, 898, 875, 836, 811, 775, 672, 665, 556. **HRMS** (ESI) for $C_{15}H_{28}O_2SiNa$ [M+Na]+: calcd. 291.1751, found 291.1750.

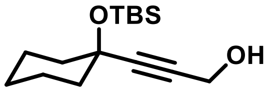

4-(1-Methoxycyclohexyl)but-3-yn-2-ol (S28). *n*-Butyllithium (1.3 mL, 1.6 M in hexanes, 2.0 mmol) was added dropwise to a solution of 1-ethynyl-1-methoxycyclohexane (**S2**, 0.28 g, 2.0 mmol) in THF (6 mL) at 0 °C. The mixture was stirred at 0 °C for 30 min. Acetaldehyde (0.13 mL, 2.4 mmol) was added at 0 °C and the reaction was maintained at 0 °C for another 15 min. The mixture was then stirred at room temperature for 14 h. The reaction was quenched with saturated aq. NH_4Cl (10 mL). The aqueous layer was extracted with *tert*-butyl methyl ether (3 × 15 mL) and the combined organic layers were washed with brine (15 mL), dried over Na_2SO_4, and concentrated

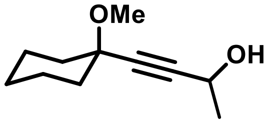

under reduced pressure. The residue was purified by flash chromatography (SiO_2, hexanes/*tert*-butyl methyl ether, 2:1) to give the desired product as a pale yellow oil (0.28 g, 75%). **^{1}H NMR** (400 MHz, $CDCl_3$) δ 4.59 (q, *J* = 6.6 Hz, 1H), 3.35 (s, 3H), 2.02 – 1.76 (m, 3H), 1.71 – 1.44 (m, 6H), 1.47 (d, *J* = 6.5 Hz, 4H), 1.39 – 1.22 (m, 1H). **^{13}C NMR** (101 MHz, $CDCl_3$) δ 88.1, 85.1, 73.9, 58.6, 50.8, 36.8, 25.6, 24.8, 22.9. **IR** (Microscope, cm^{-1}): 3396, 2980, 2935, 2858, 2825, 1449, 1369, 1328, 1292, 1258, 1184, 1170, 1146, 1122, 1093, 1080, 1030, 984, 924. **HRMS** (ESI) for $C_{11}H_{18}O_2Na$ [M+Na]+: calcd. 205.1199, found 205.1199.

3-(1-(Methoxymethoxy)cyclohexyl)prop-2-yn-1-yl acetate (15c). Triethylamine (0.21 mL, 1.5 mmol), acetic anhydride (71 µL, 0.75 mmol) and 4-dimethylaminopyridine (6.1 mg, 0.050 mmol) were added to a solution of alcohol **S26** (99 mg, 0.50 mmol) in CH_2Cl_2 (1.0 mL). The mixture was stirred at room temperature for 2 h before the reaction was quenched with saturated aq. NH_4Cl (2 mL) and the mixture diluted with *tert*-butyl methyl ether (3 mL). The aqueous phase was extracted with *tert*-butyl methyl ether (3 × 3 mL). The combined organic fractions were washed with brine, dried over Na_2SO_4, filtered, and concentrated. The residue was purified by flash chromatography (SiO_2, hexanes/*tert*-butyl methyl ether, 5:1) to give the desired product as a colorless oil (0.11 g, 88%). **^{1}H NMR** (400 MHz, $CDCl_3$) δ 4.91 (s, 2H), 4.73 (s, 2H), 3.40 (s, 3H), 2.09 (s, 3H), 2.01 – 1.89 (m, 2H), 1.74 – 1.61 (m, 4H), 1.59 – 1.46 (m, 3H), 1.27 (dt, *J* = 10.8, 3.8 Hz, 1H). **^{13}C NMR** (101 MHz, $CDCl_3$) δ 170.4, 93.0, 87.7, 80.7, 74.9, 56.0, 52.5, 38.5, 25.4, 23.1, 20.9. **IR** (Microscope, cm^{-1}): 2936, 2859, 1750, 1448, 1378, 1360, 1295, 1222, 1177, 1150, 1097, 1025, 967, 929. **HRMS** (ESI) for $C_{13}H_{20}O_4Na$ [M+Na]+: calcd. 263.1253, found 263.1254.

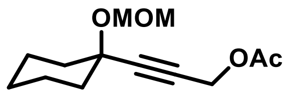

3-(1-Methoxycyclohexyl)prop-2-yn-1-yl acetate (**15a**). Prepared analogously from from alcohol **S6** (365 mg, 1.48 mmol) as a colorless oil (221 mg, 71%). **^{1}H NMR** (400 MHz, $CDCl_3$) δ 4.73 (s, 2H), 3.34 (s, 3H), 2.09 (s, 3H), 1.93 – 1.82 (m, 2H), 1.68 – 1.43 (m, 7H), 1.36 – 1.24 (m, 1H). **^{13}C NMR** (101 MHz, $CDCl_3$) δ 170.4, 87.9, 79.9, 73.9, 52.5, 50.8, 36.6, 25.5, 22.7, 20.9. **IR** (Microscope, cm^{-1}): 2935, 2858, 1746, 1447, 1377, 1360, 1218, 1169, 1090, 1028, 966, 926. **HRMS** (ESI) for $C_{12}H_{18}O_3Na$: calcd. 233.114814, found 233.114740.

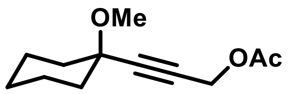

3-(1-((*tert*-Butyldimethylsilyl)oxy)cyclohexyl)prop-2-yn-1-yl acetate (15b). Prepared analogously from alcohol **S27** (0.13 g, 0.50 mmol) as a colorless oil (0.14 g, 91%). **^{1}H NMR** (400 MHz, $CDCl_3$) δ 4.71 (s, 2H), 2.09 (s, 3H), 1.76 (ddd, *J* = 10.9, 7.7, 3.8 Hz, 2H), 1.63 (dddd, *J* = 15.6, 12.2, 7.8, 3.6 Hz, 5H), 1.52 – 1.36 (m, 2H), 1.31 (tdd, *J* = 12.9, 8.9, 4.2 Hz, 1H), 0.88 (s, 9H), 0.15 (s, 6H). **^{13}C NMR** (101 MHz, $CDCl_3$) δ

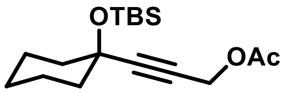

170.4, 91.5, 78.5, 69.2, 52.6, 41.0, 26.0, 25.4, 22.8, 20.9, 18.3, −2.8. **IR** (Microscope, cm^{-1}): 2934, 2896, 2857, 1753, 1472, 1463, 1446, 1377, 1360, 1253, 1221, 1178, 1138, 1101, 1055, 1024, 1002, 875, 837, 813, 776, 672. **HRMS** (ESI) for $C_{17}H_{30}O_3SiNa$ [M+Na]+: calcd. 333.1856, found 333.1856.

3-(1-(Benzyloxy)cyclohexyl)prop-2-yn-1-yl acetate (15d). Prepared analogously from alcohol

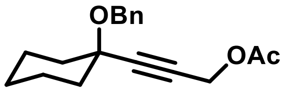

S7 (0.12 g, 0.50 mmol) as a colorless oil (0.14 g, 94%). **^{1}H NMR** (400 MHz, $CDCl_3$) δ 7.40 – 7.30 (m, 4H), 7.29 – 7.23 (m, 1H), 4.75 (s, 2H), 4.62 (s, 2H), 2.10 (s, 3H), 1.95 (dt, *J* = 12.2, 5.9 Hz, 2H), 1.71 (ddd, *J* = 14.2, 7.9, 3.5 Hz, 4H), 1.62 – 1.45 (m, 3H), 1.34 (qt, *J* = 9.2, 5.2 Hz, 1H). **^{13}C NMR** (101 MHz, $CDCl_3$) δ 170.4, 139.3, 128.4, 127.8, 127.4, 88.4, 80.1, 74.1, 65.7, 52.6, 37.2, 25.6, 22.8, 21.0. **IR** (Microscope, cm^{-1}): 2935, 2858, 1748, 1497, 1451, 1377, 1360, 1292, 1221, 1175, 1137, 1086, 1069, 1028, 966, 940, 905, 736, 698. **HRMS** (ESI) for $C_{18}H_{22}O_3Na$ [M+Na]+: calcd. 309.1461, found 309.1460.

4-(1-(Methoxymethoxy)cyclohexyl)but-3-yn-2-yl acetate (S29). Prepared analogously from

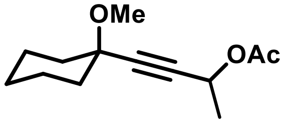

alcohol **S28** (87 mg, 0.48 mmol) as a colorless oil (96 mg, 90%). **^{1}H NMR** (400 MHz, $CDCl_3$) δ 5.50 (q, *J* = 6.7 Hz, 1H), 3.34 (s, 3H), 2.07 (s, 3H), 1.94 – 1.83 (m, 2H), 1.70 – 1.60 (m, 2H), 1.60 – 1.42 (m, 5H), 1.49 (d, *J* = 6.7 Hz, 3H), 1.29 (dq, *J* = 13.8, 5.4, 4.0 Hz, 1H). **^{13}C NMR** (101 MHz, $CDCl_3$) δ 170.0, 85.8, 84.7, 73.9, 60.6, 50.8, 36.7, 25.6, 22.9, 21.8, 21.3. **IR** (Microscope, cm^{-1}): 2987, 2936, 2859, 2825, 1745, 1448, 1371, 1339, 1292, 1232, 1185, 1174, 1134, 1093, 1048, 1031, 981, 927, 846. **HRMS** (ESI) for $C_{13}H_{20}O_3Na$ [M+Na]+: calcd. 247.1305, found 247.1302.

4-Methyl-4-((triethylsilyl)oxy)pent-2-yn-1-yl acetate (S30). Prepared analogously from alcohol

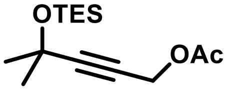

S10 (150 mg, 0.65 mmol) as a colorless oil (160 mg, 90%). **^{1}H NMR** (400 MHz, $CDCl_3$) δ 4.69 (s, 2H), 2.09 (s, 3H), 1.47 (s, 6H), 0.96 (t, *J* = 7.9 Hz, 9H), 0.66 (q, *J* = 7.9 Hz, 6H). **^{13}C NMR** (101 MHz, $CDCl_3$) δ 170.3, 92.1, 76.3, 66.2, 52.4, 32.9, 20.8, 7.0, 6.1. **IR** (Microscope, cm^{-1}): 2955, 1751, 1377, 1217, 1161, 1032, 965, 726. **HRMS** (ESI) for $C_{14}H_{26}O_3Si$: calcd. 270.44, found 270.44.

3-(1-Methoxycyclohexyl)prop-2-yn-1-yl pivalate (S31). Triethylamine (91 µL, 0.65 mmol) and

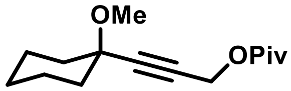

4-dimethylaminopyridine (8.0 mg, 0.06 mmol) were added to a solution of alcohol **S6** (110 mg, 0.65 mmol) in methylene chloride (5 mL) at ambient temperature. The solution was stirred for 15 min before pivaloyl chloride (88 µL, 0.71 mmol) was introduced. After 2 h of stirring, the solution was diluted with water (5 mL) and the aqueous layer was extracted with *tert*-butyl methyl ether (3 x 5 mL). The combined organic phases were washed with brine (5 mL), dried over Na_2SO_4, and concentrated *in vacuo*.

The crude material was purified by flash chromatography (silica, pentane/*tert*-butyl methyl ether, 20:1) to provide the title compound (132 mg, 80%) as a colorless oil. **^{1}H NMR** (400 MHz, $CDCl_3$) δ 4.72 (s, 2H), 3.34 (s, 3H), 1.92 – 1.84 (m, 2H), 1.69 – 1.43 (m, 8H), 1.21 (s, 9H). **^{13}C NMR** (101 MHz, $CDCl_3$) δ 177.8, 87.4, 80.4, 74.0, 52.4, 50.8, 38.8, 36.6, 27.2, 25.5, 22.8. **IR** (Microscope, cm^{-1}): 2935, 1736, 1480, 1279, 1134, 1093, 1031, 927. **HRMS** (EI) for $C_{15}H_{24}O_3$: calcd. 252.17199, found 252.17200.

3-(1-Methoxycyclohexyl)prop-2-yn-1-yl methyl carbonate (S32). Pyridine (0.42 mL, 5.34 mmol) and methyl chloroformiate were added to a stirred solution of alcohol **S6** (300 mg, 1.78 mmol) in methylene chloride (10 mL) at 0°C. The mixture was allowed to reach room temperature over the course of 15 h before sat. aq. NH_4Cl was added. The aqueous phase was extracted with diethyl ether (3 x 10 mL) and the combined organic layers were washed with brine (10 mL), dried over Na_2SO_4 and concentrated *in vacuo*. The crude material was purified by flash chromatography (silica, pentane/*tert*-butyl methyl ether, 10:1) to provide the title compound as a colorless oil (366 mg, 91%). **^{1}H NMR** (400 MHz, $CDCl_3$) δ 4.80 (s, 2H), 3.81 (s, 3H), 3.34 (s, 3H), 1.88 (ddd, J = 13.3, 6.3, 3.4 Hz, 2H), 1.72 – 1.43 (m, 7H), 1.37 – 1.26 (m, 1H). **^{13}C NMR** (101 MHz, $CDCl_3$) δ 155.3, 88.8, 79.2, 73.9, 55.9, 55.2, 50.9, 36.5, 25.5, 22.7. **IR** (Microscope, cm^{-1}): 2936, 1751, 1444, 1374, 1251, 1168, 1091, 950, 790. **HRMS** (EI) for $C_{12}H_{18}O_4$ [M]+: calcd. 226.11996, found 226.12014.

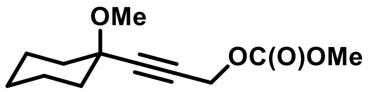

***tert*-Butyl (3-(1-methoxycyclohexyl)prop-2-yn-1-yl) carbonate (S33).** Hünig base (0.16 mL, 0.92 mmol), 4-dimethylaminopyridine (5.1 mg, 0.04 mmol) and di-*tert*-butyl dicarbonate (108 mg, 0.49 mmol) were added to a solution of alcohol **S6** (70 mg, 0.41 mmol) in methylene chloride (3 mL) at ambient temperature. The solution was stirred for 2 h before it was diluted with water (5 mL). The aqueous layer was extracted with *tert*-butyl methyl ether (3 x 5 mL). The combined organic phases were washed with brine (5 mL), dried over Na_2SO_4, and concentrated *in vacuo*. The crude material was purified by flash chromatography (silica, pentane/*tert*-butyl methyl ether, 20:1) to provide the title compound (82 mg, 74%) as a colorless oil. **^{1}H NMR** (400 MHz, $CDCl_3$) δ 4.72 (s, 2H), 3.34 (s, 3H), 1.93 – 1.84 (m, 2H), 1.68 – 1.51 (m, 7H), 1.49 (s, 9H), 1.28 (tt, J = 14.3, 9.8 Hz, 1H). **^{13}C NMR** (101 MHz, $CDCl_3$) δ 152.9, 88.3, 82.9, 79.8, 73.9, 55.0, 50.9, 36.6, 27.8, 25.5, 22.7. **IR** (Microscope, cm^{-1}): 2935, 1743, 1452, 1369, 1251, 1156, 1091, 928, 855, 791. **HRMS** (ESI) for $C_{15}H_{24}O_4Na$ [M+Na]+: calcd. 291.15668, found 291.15631.

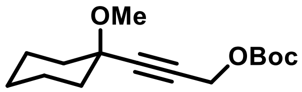

3-(1-Methoxycyclohexyl)prop-2-yn-1-yl diisopropylcarbamate (S34). Diisopropyl carbamoyl

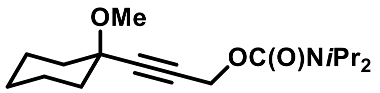

chloride (437 mg, 2.67 mmol), triethylamine (0.49 mL, 3.56 mmol) and 4-dimethylaminopyridine (10.9 mg, 0.08 mmol) were added to a solution of alcohol **S6** (300 mg, 1.78 mmol) in toluene (5 mL). The solution was stirred at reflux temperature for 15 h before it was cooled to room temperature and diluted with *tert*-butyl methyl ether (10 mL). The suspension was filtered through a short plug of Celite which was carefully rinsed with *tert*-butyl methyl ether (20 mL). The combined filtrates were evaporated *in vacuo* and the residue was purified by flash chromatography (silica, pentane/*tert*-butyl methyl ether, 10:1) to provide the title compound as a colorless oil (298 mg, 57%). **^{1}H NMR** (400 MHz, $CDCl_3$) δ 4.76 (s, 2H), 4.18 – 3.70 (bm, 2H), 3.35 (s, 3H), 1.95 – 1.85 (m, 2H), 1.70 – 1.45 (m, 8H), 1.22 (d, J = 6.8 Hz, 12H). **^{13}C NMR** (101 MHz, $CDCl_3$) δ 154.9, 87.0, 81.3, 74.0, 52.5, 50.8, 46.4 (b), 36.7, 25.5, 22.8, 20.7 (b). **IR** (Microscope, cm^{-1}): 2935, 1695, 1439, 1367, 1286, 1217, 1133, 1047, 927, 768. **HRMS** (ESI) for $C_{17}H_{29}O_3Na$ [M+Na]+: calcd. 318.20396, found 318.20416.

3-(1-Methoxycyclohexyl)prop-2-yn-1-yl methanesulfonate (**S35**). Triethylamine (0.75 mL, 5.35

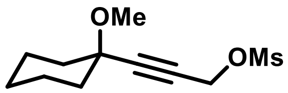

mmol) and methanesulfonyl chloride (0.17 mL, 2.14 mmol) were added to a stirred solution of alcohol **S6** (300 mg, 1.78 mmol) in diethyl ether (10 mL) at 0 °C. The mixture was allowed to reach room temperature within 15 h before sat. aq. NH_4Cl was introduced. The aqueous phase was extracted with diethyl ether (3 x 10 mL) and the combined organic layers were washed with brine (10 mL), dried over Na_2SO_4 and concentrated *in vacuo*. The crude material was purified by flash chromatography (silica, pentane/*tert*-butyl methyl ether, 3:1) to provide the title compound as a white solid (388 mg, 88%). **^{1}H NMR** (400 MHz, $CDCl_3$) δ 4.93 (s, 2H), 3.35 (s, 3H), 3.12 (s, 3H), 1.92 – 1.82 (m, 2H), 1.74 – 1.42 (m, 7H), 1.39 – 1.28 (m, 1H). **^{13}C NMR** (101 MHz, $CDCl_3$) δ 91.4, 77.8, 73.8, 58.0, 51.0, 39.1, 36.4, 25.3, 22.6. **IR** (Microscope, cm^{-1}): 2937, 1652, 1336, 1169, 1123, 1078, 978, 950, 915, 877, 846, 800, 761, 740. **HRMS** (ESI) for $C_{11}H_{18}O_4NaS$ [M+Na]+: calcd. 269.08180, found 269.08148.

1-(3-Bromoprop-1-yn-1-yl)-1-methoxycyclohexane (S36). Methanesulfonyl chloride (0.17 mL,

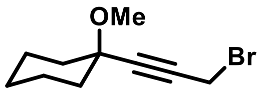

2.2 mmol) and triethylamine (0.33 mL, 2.4 mmol) were added to alcohol **S6** (0.33 g, 2.0 mmol) in THF (30 mL). The resulting suspension was stirred for 30 min. In a separate flask, lithium bromide (1.1 g, 13 mmol) was dissolved in THF (6 mL). The mesylate solution was transferred via a fritted cannula to the flask containing the lithium bromide solution (rinsing the flask with THF (2 mL)). The resulting yellow suspension was stirred under argon at room temperature for 20 h before the mixture was diluted with water (30 mL) and extracted with pentane (3 × 30 mL). The combined organic layers were washed with

brine (50 mL), dried over Na_2SO_4, and concentrated *in vacuo*. The residue was and purified by bulb-to-bulb distillation to provide the desired product as a colorless oil (0.43 g, 93%). **^{1}H NMR** (400 MHz, $CDCl_3$) δ 3.98 (s, 2H), 3.35 (s, 3H), 1.96 – 1.86 (m, 2H), 1.73 – 1.44 (m, 6H), 1.36 – 1.22 (m, 2H). **^{13}C NMR** (101 MHz, $CDCl_3$) δ 88.42, 81.21, 74.07, 50.96, 36.63, 25.52, 22.81, 14.72. **IR** (Microscope, cm^{-1}): 2935, 2857, 2824, 1447, 1341, 1293, 1258, 1209, 1185, 1171, 1146, 1092, 1027, 927, 903, 845, 819, 613, 521. **HRMS** (ESI) for $C_{10}H_{15}ONaBr$ [M+Na]+: calcd. 253.0199, found 253.0200.

Hydrogenative Rearrangement and Hydrogenative Cycloisomerization Reactions

Representative Procedure. A flame-dried Schlenk tube was charged with [CpRu(MeCN)$_3$]PF$_6$ (**C3**, 8.6 mg, 0.020 mmol, 10 mol%) or [Cp^{COOMe}Ru(MeCN)$_3$]PF$_6$ (**C5**, 9.8 mg, 0.020 mmol, 10 mol%) under argon. CH_2Cl_2 (2.0 mL) and the substrate (0.20 mmol) were added sequentially and the Schlenk tube was sealed with a septum. The headspace of the reaction vessel was purged with hydrogen for 2 min using a hydrogen-filled balloon and an outlet cannula. The outlet cannula was removed and the reaction mixture was vigorously stirred (1200 rpm) under a hydrogen atmosphere. After 16 h, the flask was vented, the solution was diluted with pentane (4.0 mL) and filtered through a plug of silica, which was rinsed with pentane/*tert*-butyl methyl ether (5:1, 12 mL). The combined filtrates were evaporated and the residue was subjected to flash chromatography or preparative HPLC to afford the desired product.

In all hydrogenative cycloisomerization reactions, [CpRu(MeCN)$_3$]PF$_6$ (**C3**, 10 mol%) was used as the catalyst.

***tert*-Butyldimethyl((2-methyl-1-(2-methylcyclopent-1-en-1-yl)propan-2-yl)oxy)silane (4a).**

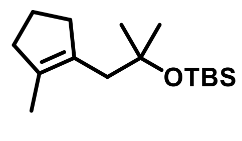

Prepared analogously from substrate **1a**; colorless oil (50 mg, 93%). **^{1}H NMR** (400 MHz, $CDCl_3$) δ 2.41 – 2.34 (m, 2H), 2.29 – 2.23 (m, 2H), 2.21 (s, 2H), 1.72 (tt, *J* = 8.1, 6.8 Hz, 2H), 1.64 – 1.57 (m, 3H), 1.18 (s, 6H), 0.86 (s, 9H), 0.08 (s, 6H). **^{13}C NMR** (101 MHz, $CDCl_3$) δ 134.1, 133.2, 75.2, 44.1, 38.5, 38.4, 30.3, 26.0, 22.3, 18.1, 14.7, 1.1. **IR** (Microscope, cm^{-1}): 2929, 1247, 1150, 839, 776. **MS** (EI) for $C_{16}H_{32}OSi$: calcd. 268.51, found 268.51.

Triethyl((2-methyl-1-(2-methylcyclopent-1-en-1-yl)propan-2-yl)oxy)silane (4b). Prepared analogously from substrate **1b**; colorless liquid (46 mg, 86%). **^{1}H NMR** (400 MHz, $CDCl_3$) δ 2.43 – 2.35 (m, 2H), 2.26 (ddtt, *J* = 7.6, 5.3, 2.2, 1.1 Hz, 2H), 2.20 (s, 2H), 1.72 (tt, *J* = 8.1, 6.8 Hz, 2H), 1.61 (dt, *J* = 2.2, 1.2 Hz, 3H), 1.19 (s, 6H), 0.94 (t, *J* = 7.9 Hz, 9H), 0.65 – 0.52 (m, 6H). **^{13}C NMR** (101 MHz, $CDCl_3$) δ 136.1, 135.2, 77.1, 46.0, 40.5, 40.5, 32.4, 24.2, 16.7, 9.2, 8.9. **IR** (Microscope, cm^{-1}): 2954, 2911, 2876, 1174, 1152, 1127, 1034, 1006, 719, 670. **HRMS** (ESI) for $C_{16}H_{32}OSiNa$ [M+Na]: calcd. 291.21146, found 291.21154.

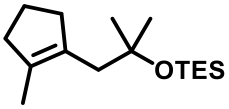

Triisopropyl((2-methyl-1-(2-methylcyclopent-1-en-1-yl)propan-2-yl)oxy)silane (4c). Prepared analogously from substrate **1c**; colorless oil (54 mg, 87%). **^{1}H NMR** (400 MHz, $CDCl_3$) δ 2.43 – 2.34 (m, 2H), 2.33 – 2.22 (m, 4H), 1.73 (tt, *J* = 8.2, 6.8 Hz, 2H), 1.67 – 1.59 (m, 3H), 1.22 (s, 6H), 1.10 – 1.02 (m, 21H). **^{13}C NMR** (101 MHz, $CDCl_3$) δ 134.4, 133.0, 75.1, 44.4, 38.5, 38.5, 30.6, 22.3, 18.5, 14.8, 13.6. **IR** (Microscope, cm^{-1}): 2938, 2864, 1458, 1378, 1248, 1160, 1044, 881, 678. **MS** (EI) for $C_{19}H_{38}OSi$: calcd. 310.59, found 310.59.

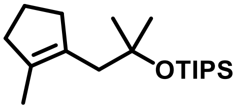

***tert*-Butyldimethyl((4-((2-methylcyclopent-1-en-1-yl)methyl)tetrahydro-2H-pyran-4-yl)oxy)-silane (5).** Prepared analogously from substrate **S19**; colorless oil (45 mg, 73%). **^{1}H NMR** (400 MHz, $CDCl_3$) δ 3.79 (td, *J* = 11.2, 2.3 Hz, 2H), 3.72 – 3.62 (m, 2H), 2.40 – 2.31 (m, 4H), 2.26 (t, *J* = 7.5 Hz, 2H), 1.80 – 1.68 (m, 4H), 1.61 (s, 3H), 1.39 (dq, *J* = 14.1, 2.8 Hz, 2H), 0.91 (s, 9H), 0.13 (s, 6H). **^{13}C NMR** (101 MHz, $CDCl_3$) δ 135.8, 131.4, 74.5, 64.2, 42.8, 38.9, 38.5, 38.0, 26.2, 22.2, 18.6, 15.1, –1.40. **IR** (Microscope, cm^{-1}): 2952, 2927, 2856, 1253, 1150, 1105, 1046, 1008, 871, 833, 771. **HRMS** (ESI) for $C_{18}H_{34}O_2SiNa$ [M+Na]: calcd. 333.22203, found 333.22207.

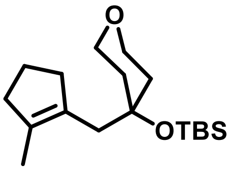

***tert*-Butyldimethyl(((1r,4r)-1-((2-methylcyclopent-1-en-1-yl)methyl)-4 (trifluoromethyl)-cyclohexyl)oxy)silane (6).** Prepared analogously from substrate **S20**; colorless oil (59 mg, 78%). **^{1}H NMR** (400 MHz, $CDCl_3$) δ 2.48 – 2.36 (m, 2H), 2.31 (s, 2H), 2.28 – 2.18 (m, 2H), 2.07 – 1.98 (m, 1H), 1.92 – 1.78 (m, 4H), 1.73 (tt, *J* = 8.2, 6.8 Hz, 2H), 1.66 – 1.60 (m, 3H), 1.54 – 1.39 (m, 4H), 0.87 (s, 9H), 0.10 (s, 6H).**^{13}C NMR** (101 MHz, $CDCl_3$) δ 134.4, 131.9, 129.4 (q, *J* = 278.5 Hz), 75.7, 40.6 (q, *J* = 26.5 Hz), 38.4, 38.1, 37.5, 36.8, 26.1, 22.5, 22.2, 18.3, 14.7, –1.3. **IR** (Microscope, cm^{-1}): 2952, 1338, 1279, 1252, 1167, 1085, 830, 771, 690. **HRMS** (ESI) for $C_{20}H_{35}F_3OSiNa$ [M+Na]: calcd. 399.23015, found 399.23036.

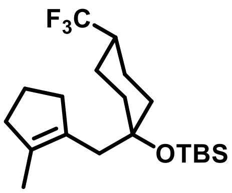

***tert*-Butyldimethyl((1-((2,4,4-trimethylcyclopent-1-en-1-yl)methyl)cyclohexyl)oxy)silane (7).**

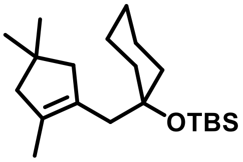

Prepared analogously from substrate **S22**; colorless oil (49 mg, 73%). **^{1}H NMR** (400 MHz, $CDCl_3$) δ 2.23 (s, 2H), 2.22 – 2.17 (m, 2H), 2.08 (d, *J* = 2.4 Hz, 2H), 1.71 – 1.62 (m, 2H), 1.61 – 1.57 (m, 3H), 1.50 – 1.10 (m, 8H), 1.04 (s, 6H), 0.90 (s, 9H), 0.11 (s, 6H). **^{13}C NMR** (101 MHz, $CDCl_3$) δ 133.3, 131.0, 77.2, 54.0, 53.5, 42.8, 38.2, 36.5, 29.9, 26.3, 25.9, 22.5, 18.6, 15.2, −1.4. **IR** (Microscope, cm^{-1}): 2927, 2856, 1462, 1361, 1251, 1145, 1056, 1026, 1005, 833, 770, 686. **HRMS** (ESI) $C_{21}H_{40}OSiNa$ [M+Na]: calcd. 359.27406, found 359.27424.

Dimethyl 3-((1-((*tert*-butyldimethylsilyl)oxy)cyclohexyl)methyl)-4-methylcyclopent-3-ene-1,1-dicarboxylate (8).

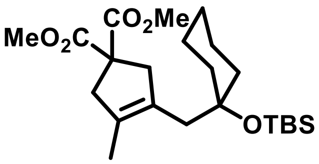

Prepared analogously from substrate **S23**; colorless oil (78 mg, 91%). **^{1}H NMR** (400 MHz, $CDCl_3$) δ 3.71 (s, 6H), 3.05 (q, *J* = 2.0 Hz, 2H), 2.93 (dt, *J* = 2.7, 1.3 Hz, 2H), 2.25 (s, 2H), 1.70 – 1.56 (m, 5H), 1.49 – 1.32 (m, 8H), 0.88 (s, 9H), 0.10 (s, 6H). **^{13}C NMR** (101 MHz, $CDCl_3$) δ 173.0, 131.6, 129.7, 76.8, 57.5, 52.8, 46.1, 45.7, 41.4, 38.2, 26.2, 25.7, 22.8, 18.5, 14.4, −1.4. **IR** (Microscope, cm^{-1}): 2929, 1737, 1434, 1250, 1145, 1057, 834, 771. **HRMS** (ESI) for $C_{23}H_{40}O_5SiNa$ [M+Na]: calcd. 447.25372, found 447.25344.

***tert*-Butyl((2-(4-fluorophenyl)-1-(2-methylcyclopent-1-en-1-yl)propan-2-yl)oxy)dimethylsilane (9).**

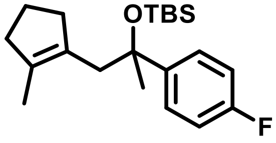

Prepared analogously from substrate **S24**; colorless oil (52 mg, 75%). **^{1}H NMR** (400 MHz, $CDCl_3$) δ 7.48 – 7.30 (m, 2H), 6.95 (t, *J* = 8.8 Hz, 2H), 2.53 (d, *J* = 13.5 Hz, 2H), 2.40 (d, *J* = 13.5 Hz, 2H), 2.24 – 2.04 (m, 3H), 1.71 – 1.51 (m, 6H), 1.48 – 1.31 (m, 3H), 0.92 (s, 9H), 0.06 (s, 3H), -0.16 (s, 3H). **^{13}C NMR** (101 MHz, $CDCl_3$) δ 161.7 (d, *J* = 244.0 Hz), 144.7 (d, *J* = 3.1 Hz), 135.2, 132.0, 127.3 (d, *J* = 8.0 Hz), 114.2 (d, *J* = 20.8 Hz), 77.7, 46.2, 38.3, 37.8, 28.2, 26.2, 22.3, 18.5, 14.3, −1.6, −2.3. **^{19}F NMR** (282 MHz, $CDCl_3$) δ -117.39. **IR** (Microscope, cm^{-1}): 2929, 1508, 1254, 1225, 1159, 1091, 996, 833, 773, 582. **HRMS** (ESI) for $C_{21}H_{34}FOSi$ [M+H]: calcd. 349.23575, found 349.23541.

2,2,3,3,5,8,8,9,9-Nonamethyl-5-((2-methylcyclopent-1-en-1-yl)methyl)-4,7-dioxa-3,8-disiladecane (10).

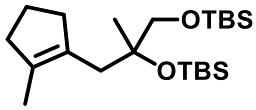

Prepared analogously from substrate **S25**; colorless oil (50 mg, 63%). **^{1}H NMR** (400 MHz, $CDCl_3$) δ 3.44 – 3.29 (m, 2H), 2.52 – 2.13 (m, 6H), 1.72 (p, *J* = 7.5 Hz, 2H), 1.64 – 1.58 (m, 3H), 1.12 (s, 3H), 0.90 (s, 9H), 0.85 (s, 9H), 0.07 (s, 6H), 0.04 (s, 6H). **^{13}C NMR** (101 MHz, $CDCl_3$) δ 134.1, 132.7, 77.6, 70.8, 38.9, 38.4, 26.1, 26.1, 24.8, 22.3, 18.5, 18.3, 14.6, −1.6, −1.7, −5.2, −5.3. **IR** (Microscope,

cm^{-1}): 2953, 2929, 2857, 1253, 1098, 1026, 832, 773. **HRMS** (ESI) for $C_{22}H_{46}O_2Si_2$ [M+Na]: calcd. 421.29286, found 421.29275.

3-(1-Methoxycyclohexyl)prop-1-en-2-yl acetate (17a). Prepared analogously from substrate

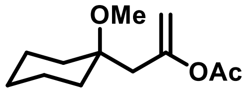

15a (1.63 g, 7.75 mmol) using $[Cp^{COOMe}Ru(MeCN)_3]PF_6$ (381 mg, 0.77 mmol, 10 mol%); colorless oil (1.47 g, 89%). **^{1}H NMR** (400 MHz, $CDCl_3$) δ 4.85 (d, *J* = 1.3 Hz, 1H), 4.78 (dd, *J* = 1.3, 0.6 Hz, 1H), 3.17 (s, 3H), 2.38 (s, 2H), 2.13 (s, 3H), 1.81 – 1.72 (m, 2H), 1.63 – 1.49 (m, 3H), 1.47 – 1.39 (m, 2H), 1.38 – 1.27 (m, 2H), 1.28 – 1.20 (m, 1H). **^{13}C NMR** (101 MHz, $CDCl_3$) δ 169.4, 152.9, 105.1, 74.9, 48.5, 39.9, 34.1, 25.8, 21.9, 21.3. **IR** (Microscope, cm^{-1}): 2932, 2856, 1752, 1662, 1456, 1368, 1190, 1073, 1020, 953, 871. **HRMS** (ESI+) for $C_{12}H_{20}O_3Na$: calcd. 235.13046, found 235.13035.

3-(1-((*tert*-Butyldimethylsilyl)oxy)cyclohexyl)prop-1-en-2-yl acetate (17b). Prepared

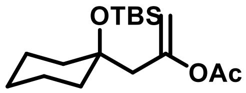

analogously from substrate **15b** using $[Cp^{COOMe}Ru(MeCN)_3]PF_6$ (9.8 mg, 0.020 mmol); colorless oil (45 mg, 72%). **^{1}H NMR** (400 MHz, $CDCl_3$) δ 4.83 (d, *J* = 1.2 Hz, 1H), 4.79 (d, *J* = 1.2 Hz, 1H), 2.44 (s, 2H), 2.12 (s, 3H), 1.70 – 1.59 (m, 4H), 1.54 – 1.44 (m, 2H), 1.43 – 1.29 (m, 4H), 0.88 (s, 9H), 0.09 (s, 6H). **^{13}C NMR** (101 MHz, $CDCl_3$) δ 169.3, 153.4, 105.5, 75.0, 45.8, 38.0, 26.1, 25.7, 22.9, 21.4, 18.5, -1.5. **IR** (Microscope, cm^{-1}): 2829, 2856, 1757, 1369, 1252, 1196, 1057, 1020, 832, 770. **HRMS** (ESI) for $C_{17}H_{32}O_3SiNa$ [M+Na]+: calcd. 335.20129, found 335.20112.

3-(1-(Methoxymethoxy)cyclohexyl)prop-1-en-2-yl acetate (17c). Prepared analogously from

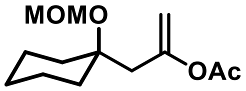

substrate **15c** (48 mg, 0.20 mmol) using $[Cp^{COOMe}Ru(MeCN)_3]PF_6$ (9.8 mg, 0.020 mmol), colorless oil (36 mg, 75%). **^{1}H NMR** (400 MHz, $CDCl_3$) δ 4.85 (d, *J* = 1.3 Hz, 1H), 4.79 (d, *J* = 1.3 Hz, 1H), 4.71 (s, 2H), 3.40 (s, 3H), 2.47 (s, 2H), 2.12 (s, 3H), 1.85 – 1.72 (m, 2H), 1.71 – 1.57 (m, 2H), 1.55 – 1.37 (m, 5H), 1.28 (dtt, *J* = 12.8, 9.3, 3.3 Hz, 1H). **^{13}C NMR** (101 MHz, $CDCl_3$) δ 169.3, 152.9, 105.6, 90.8, 77.0, 55.8, 42.3, 35.1, 25.7, 22.3, 21.4. **IR** (Microscope, cm^{-1}): 2932, 2863, 2821, 1755, 1663, 1450, 1370, 1199, 1160, 1141, 1086, 1030, 955, 921. **HRMS** (ESI) for $C_{13}H_{22}O_4Na$ [M+Na]+: calcd. 265.1410, found 265.1409.

3-(1-(Benzyloxy)cyclohexyl)prop-1-en-2-yl acetate (17d). Prepared analogously from

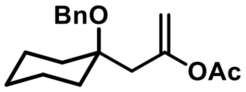

substrate **15d** (57 mg, 0.20 mmol) using $[Cp^{COOMe}Ru(MeCN)_3]PF_6$ (20 mg, 0.040 mmol, 20 mol%); colorless oil (25 mg, 43%). Purified by preparative HPLC (column: YMC-Triart C18, S-5 µm, 150 mm length, 20.0 mm ID; gradient: 15.0 mL/min MeOH/H_2O 80:20; t_R = 12.37 min). Analytical HPLC: column: Eclipse Plus C18, 1.8 µm, 50 mm length, 4.6 mm ID; gradient: 1.0 mL/min, MeOH/H_2O 75:25; t_R = 3.66 min. **^{1}H NMR** (400 MHz, $CDCl_3$) δ 7.43 – 7.29 (m, 4H), 7.27 – 7.21 (m, 1H), 4.85 (d, J = 1.4 Hz, 1H),

4.84 – 4.81 (m, 1H), 4.42 (s, 2H), 2.52 (s, 2H), 1.96 (s, 3H), 1.95 – 1.88 (m, 2H), 1.72 – 1.56 (m, 3H), 1.54 – 1.37 (m, 4H), 1.33 – 1.20 (m, 1H). **^{13}C NMR** (101 MHz, $CDCl_3$) δ 169.4, 153.0, 139.6, 128.4, 127.2, 127.2, 105.2, 75.3, 62.5, 41.0, 34.6, 25.8, 22.1, 21.3. **IR** (Microscope, cm^{-1}): 2932, 2858, 1753, 1663, 1450, 1369, 1224, 1201, 1132, 1090, 1064, 1026, 959, 874, 735, 697. **HRMS** (ESI) for $C_{18}H_{24}O_3Na$ [M+Na]+: calcd. 311.1617, found 311.1618.

3-(1-Methoxycyclohexyl)prop-1-en-2-yl pivalate (18). Prepared analogously from substrate

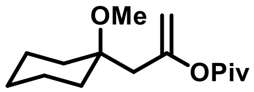

S31 using [$Cp^{COOMe}Ru(MeCN)_3]PF_6$ (9.8 mg, 0.020 mmol); colorless oil (40 mg, 78%). **^{1}H NMR** (400 MHz, $CDCl_3$) δ 4.86 – 4.75 (m, 2H), 3.17 (s, 3H), 2.37 (s, 2H), 1.75 (dd, *J* = 10.9, 7.2 Hz, 2H), 1.62 – 1.31 (m, 8H), 1.25 (s, 9H). **^{13}C NMR** (101 MHz, $CDCl_3$) δ 177.0, 153.3, 104.7, 74.9, 48.5, 40.4, 39.0, 34.0, 27.2, 25.7, 21.9. **IR** (Microscope, cm^{-1}): 2933, 2859, 1741, 1662, 1480, 1458, 1281, 1123, 1075, 868. **HRMS** (ESI) for $C_{15}H_{26}O_3Na$ [M+Na]+: calcd. 277.17741, found 277.17771.

4-Methyl-4-((triethylsilyl)oxy)pent-1-en-2-yl acetate (19). Prepared analogously from substrate

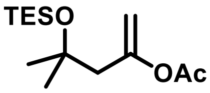

S30 using [$Cp^{COOMe}Ru(MeCN)_3]PF_6$ (9.8 mg, 0.020 mmol); colorless oil (51 mg, 94%). **^{1}H NMR** (400 MHz, $CDCl_3$) δ 4.81 (d, *J* = 1.2 Hz, 1H), 4.77 (d, *J* = 1.1 Hz, 1H), 2.37 (s, 2H), 2.11 (s, 3H), 1.26 (s, 6H), 0.94 (t, *J* = 7.9 Hz, 9H), 0.57 (q, *J* = 7.8 Hz, 6H). **^{13}C NMR** (101 MHz, $CDCl_3$) δ 169.3, 153.8, 105.0, 72.8, 48.8, 29.9, 21.4, 7.2, 6.8. **IR** (Microscope, cm^{-1}): 2956, 1757, 1367, 1189, 1155, 1041, 872, 722. **HRMS** (ESI) for $C_{14}H_{28}O_3SiNa$ [M+Na]+: calcd. 295.16999, found 295.16994.

3-(1-Methoxycyclohexyl)prop-1-en-2-yl methyl carbonate (20a). Prepared analogously from

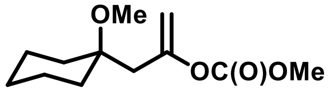

substrate **S32** using [$Cp^{COOMe}Ru(MeCN)_3]PF_6$ (9.8 mg, 0.020 mmol); colorless oil (35 mg, 77%). **^{1}H NMR** (400 MHz, $CDCl_3$) δ 4.97 (d, *J* = 1.6 Hz, 1H), 4.84 – 4.74 (m, 1H), 3.82 (s, 3H), 3.17 (s, 3H), 2.41 (s, 2H), 1.82 – 1.73 (m, 2H), 1.60 – 1.49 (m, 3H), 1.48 – 1.39 (m, 2H), 1.38 – 1.29 (m, 2H), 1.28 – 1.16 (m, 1H). **^{13}C NMR** (101 MHz, $CDCl_3$) δ 153.8, 153.1, 104.7, 74.8, 55.1, 48.5, 39.7, 34.0, 25.7, 21.9. **IR** (Microscope, cm^{-1}): 2933, 1755, 1441, 1272, 1214, 1072, 947, 869, 784. **HRMS** (EI) for $C_{12}H_{20}O_4$: calcd. 228.13561, found 228.13560.

***tert*-Butyl (3-(1-methoxycyclohexyl)prop-1-en-2-yl) carbonate (20b).** Prepared analogously

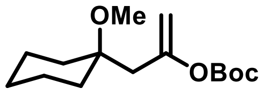

from substrate **S33** using [$Cp^{COOMe}Ru(MeCN)_3]PF_6$ (9.8 mg, 0.020 mmol); colorless oil (34 mg, 63%). **^{1}H NMR** (400 MHz, $CDCl_3$) δ 4.91 (d, *J* = 1.4 Hz, 1H), 4.76 (d, *J* = 1.4 Hz, 1H), 3.17 (s, 3H), 2.41 (s, 2H), 1.82 – 1.71 (m, 2H), 1.60 – 1.50 (m, 3H), 1.46 – 1.39 (m, 9H), 1.35 (ddd, *J* = 14.5, 11.2, 3.9 Hz, 5H), 1.23 (dddd, *J* = 11.0, 7.4, 4.4, 1.7 Hz, 1H). **^{13}C NMR** (101 MHz, $CDCl_3$) δ 153.0, 151.5, 104.6, 82.7, 74.8, 48.4,

39.8, 34.0, 27.8, 25.7, 21.9. **IR** (Microscope, cm^{-1}): 2933, 1747, 1369, 1283, 1226, 1143, 1073, 835, 784, 751. **HRMS** (ESI) for $C_{15}H_{26}O_4Na$ [M+Na]+: calcd. 293.17233, found 293.17204.

3-(1-Methoxycyclohexyl)prop-1-en-2-yl diisopropyl carbamate (21). Prepared analogously from substrate **S34** using $[Cp^{COOMe}Ru(MeCN)_3]PF_6$ (9.8 mg, 0.020 mmol); colorless oil (37 mg, 62%). **^{1}H NMR** (400 MHz, $CDCl_3$) δ 4.80 (d, J = 1.0 Hz, 1H), 4.72 (d, J = 1.0 Hz, 1H), 3.92 (bd, J = 26.5 Hz, 2H), 3.18 (s, 3H), 2.47 (s, 2H), 1.79 – 1.71 (m, 2H), 1.60 – 1.50 (m, 3H), 1.48 – 1.34 (m, 3H), 1.31 – 1.18 (m, 14H). **^{13}C NMR** (101 MHz, $CDCl_3$) δ 153.5, 104.1, 74.9, 48.4, 46.3 (b), 40.3, 34.1, 25.8, 21.9, 21.3 (b), 20.7 (b). **IR** (Microscope, cm^{-1}): 2932, 1698, 1431, 1368, 1310, 1216, 1149, 1075, 1045, 919, 731. **HRMS** (ESI) for $C_{17}H_{31}NO_3Na$ [M+Na]+: calcd. 320.21961, found 320.21982.

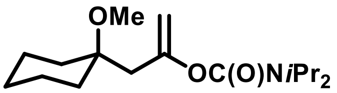

3-(1-Methoxycyclohexyl)prop-1-en-2-yl methanesulfonate (22). Prepared analogously from substrate **S35** using $[Cp^{COOMe}Ru(MeCN)_3]PF_6$ (9.8 mg, 0.020 mmol); white, waxy solid (38 mg, 77%). **^{1}H NMR** (400 MHz, $CDCl_3$) δ 5.16 (d, J = 2.4 Hz, 1H), 4.96 – 4.83 (m, 1H), 3.20 (s, 3H), 3.12 (s, 3H), 2.44 (s, 2H), 1.88 – 1.73 (m, 2H), 1.63 – 1.51 (m, 3H), 1.51 – 1.39 (m, 2H), 1.34 (ddd, J = 13.3, 11.1, 4.1 Hz, 2H), 1.23 (dtd, J = 8.8, 6.1, 4.5 Hz, 1H). **^{13}C NMR** (101 MHz, $CDCl_3$) δ 152.5, 105.5, 74.8, 48.6, 41.0, 37.8, 34.1, 25.7, 21.8. **IR** (Microscope, cm^{-1}): 2933, 1657, 1334, 1160, 1123, 1071, 968, 948, 914, 877, 845, 810, 689. **HRMS** (ESI) for $C_{11}H_{20}O_4NaS$: calcd. 271.09745, found 271.09755.

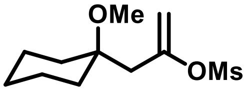

1-(2-Bromoallyl)-1-methoxycyclohexane (23). Prepared analogously from substrate **S36** using $[Cp^{COOMe}Ru(MeCN)_3]PF_6$ (9.8 mg, 0.020 mmol); The copolar side product **S37** could not be removed with flash chromatography on silica gel. Therefore a second flash chromatography with cyano silica gel was performed (hexanes:ethyl acetate = 70:1); slightly volatile colorless liquid (27 mg, 58%; the yield refers to the isolated yield after the second purification). **^{1}H NMR** (400 MHz, $CDCl_3$) δ 5.65 (dt, J = 1.7, 0.9 Hz, 1H), 5.59 (d, J = 1.5 Hz, 1H), 3.20 (s, 3H), 2.63 (d, J = 1.0 Hz, 2H), 1.83 – 1.75 (m, 2H), 1.63 – 1.53 (m, 3H), 1.51 – 1.42 (m, 2H), 1.41 – 1.31 (m, 2H), 1.24 (dddd, J = 11.5, 8.9, 5.8, 4.0 Hz, 1H). **^{13}C NMR** (101 MHz, $CDCl_3$) δ 128.3, 120.6, 75.5, 48.5, 47.0, 34.1, 25.8, 21.8. **IR** (Microscope, cm^{-1}): 2932, 2848, 1501, 1346, 1291, 1258, 1209, 1171, 1144, 1096, 1027, 927, 903, 845, 574. **HRMS** (Ci) for $C_{10}H_{18}BrO$ [M]: calcd. 233.05357, found 233.05373.

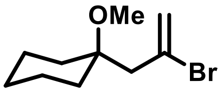

1-Bromo-1-cyclohexylidenepropan-2-one (S37). This compound was the side product separated from alkenyl bromide (**S36**) by flash chromatography on cyano silica gel; colorless oil (8 mg, 18%). **^{1}H NMR** (600 MHz, $CDCl_3$) δ 2.47 (s, 3H), 2.45 – 2.42 (m, 4H), 1.63 (d, *J* = 5.9 Hz, 2H), 1.57 (dt, *J* = 6.0, 2.9 Hz, 4H). **^{13}C NMR** (151 MHz, $CDCl_3$) δ 198.1, 149.1, 114.4, 35.4, 32.5, 30.0, 28.0, 27.3, 26.0. **IR** (Microscope, cm^{-1}): 2930, 1623, 1454, 1148, 1074, 886, 816, 574, 502. **MS** (ESI) for $C_9H_{13}BrO$: calcd. 216.01444, found 216.01462.

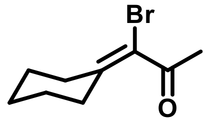

(*E*)-1-(1-Methoxycyclohexyl)but-2-en-2-yl acetate (24). Prepared analogously from substrate **S29** (44 mg, 0.20 mmol) using [Cp^{COOMe}Ru(MeCN)$_3$]PF_6 (9.8 mg, 0.020 mmol), colorless oil (23 mg, 51%). ^{1}H NMR of the crude reaction mixture before isolation of the pure product shows an *E*/*Z* ratio of 7:3. NOESY indicates that the isolated major product is the *E*-alkene. **^{1}H NMR** (400 MHz, $CDCl_3$) δ 5.29 (q, *J* = 7.1 Hz, 1H), 3.16 (s, 3H), 2.40 (s, 2H), 2.10 (s, 3H), 1.85 – 1.70 (m, 2H), 1.65 (d, *J* = 7.0 Hz, 3H), 1.60 – 1.39 (m, 5H), 1.36 – 1.25 (m, 2H), 1.24 – 1.09 (m, 1H). **^{13}C NMR** (101 MHz, $CDCl_3$) δ 170.0, 146.4, 115.6, 76.0, 48.6, 35.8, 34.2, 25.7, 21.8, 21.3, 12.6. **IR** (Microscope, cm^{-1}): 2931, 2857, 2826, 1745, 1687, 1455, 1444, 1367, 1226, 1203, 1179, 1165, 1149, 1131, 1076, 1004, 949, 906, 851, 839, 818, 729, 604, 540. **HRMS** (ESI) for $C_{13}H_{22}O_3Na$ [M+Na]+: calcd. 249.1461, found 249.1462.

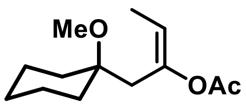

Methyl 2-methoxy-5-((1-methoxycyclohexyl)methyl)furan-3-carboxylate (25). Prepared analogously from enyne **S7** (28 mg, 0.10 mmol) using [CpRu(MeCN)$_3$]PF_6 (4.3 mg, 0.010 mmol); colorless oil (22 mg, 78%). **^{1}H NMR** (400 MHz, $CDCl_3$) δ 6.26 (t, *J* = 0.8 Hz, 1H), 4.07 (s, 3H), 3.77 (s, 3H), 3.24 (s, 3H), 2.64 (d, *J* = 0.8 Hz, 2H), 1.78 – 1.64 (m, 2H), 1.61 – 1.38 (m, 5H), 1.35 – 1.14 (m, 3H). **^{13}C NMR** (101 MHz, $CDCl_3$) δ 163.8, 161.3, 142.2, 109.2, 91.7, 75.1, 58.0, 51.3, 48.6, 35.1, 34.0, 25.8, 21.9. The spectral data is consistent with those previously reported.[9]

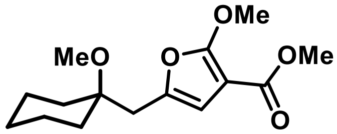

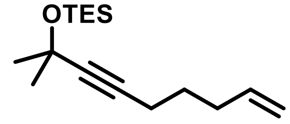
OTES

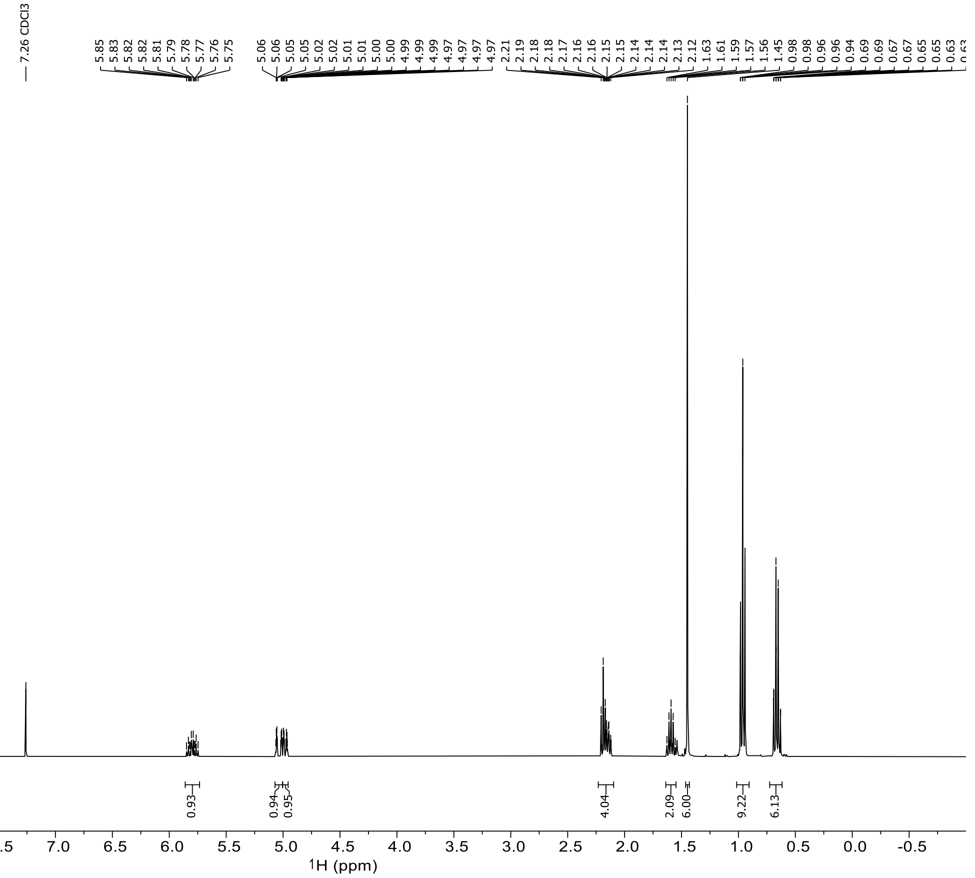
7.26 CDCl3
5.85
5.83
5.82
5.82
5.81
5.79
5.78
5.77
5.76
5.75
5.06
5.06
5.05
5.05
5.02
5.02
5.01
5.00
5.00
4.99
4.99
4.99
4.97
4.97
4.97
4.97
2.21
2.19
2.18
2.18
2.17
2.16
2.16
2.15
2.15
2.14
2.14
2.14
2.13
2.12
1.63
1.61
1.59
1.57
1.56
1.45
0.98
0.98
0.96
0.96
0.94
0.69
0.69
0.67
0.67
0.65
0.65
0.63
0.93
0.94
0.95
4.04
2.09
6.00
9.22
6.13
1H (ppm)

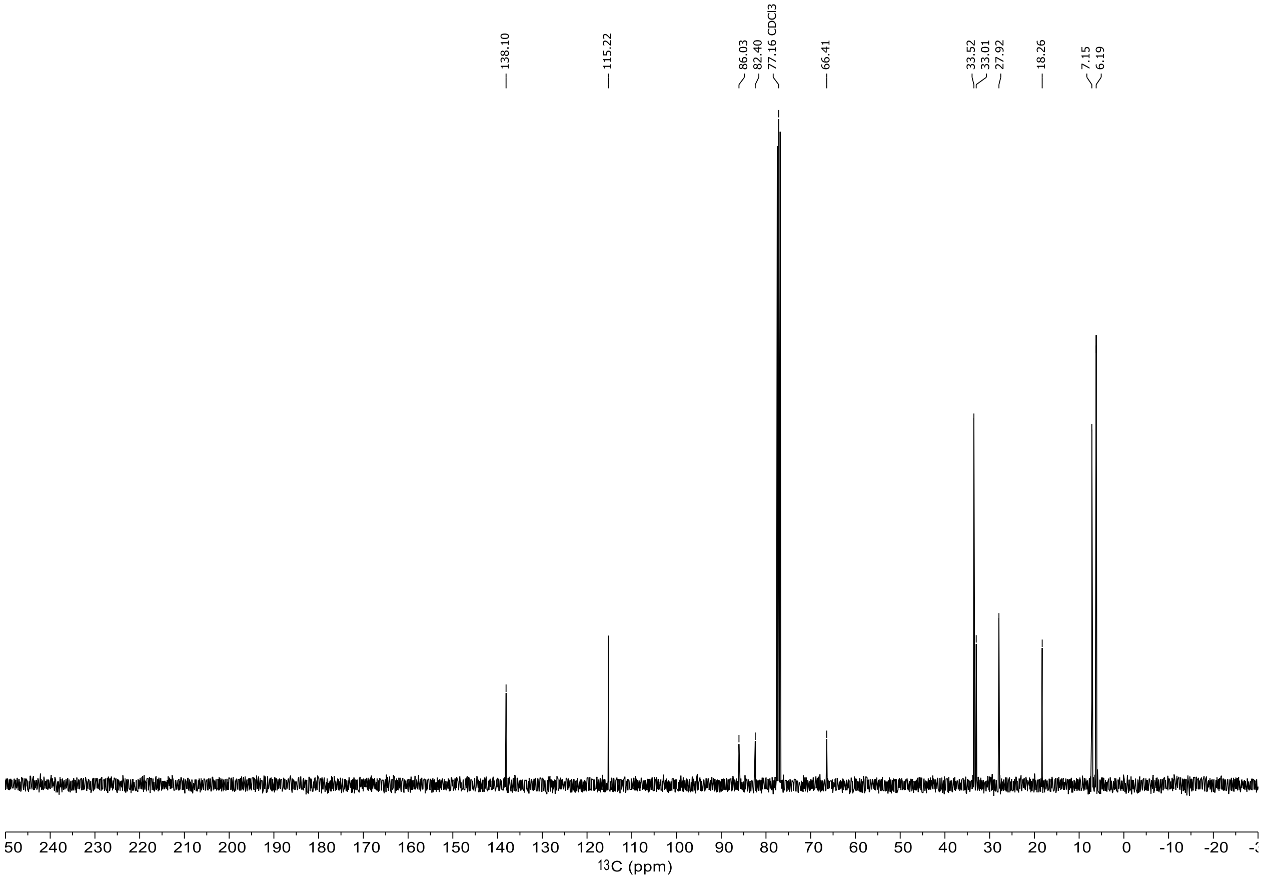
138.10
115.22
86.03
82.40
77.16 CDCl3
66.41
33.52
33.01
27.92
18.26
7.15
6.19
13C (ppm)

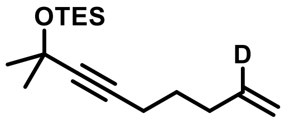
OTES
D

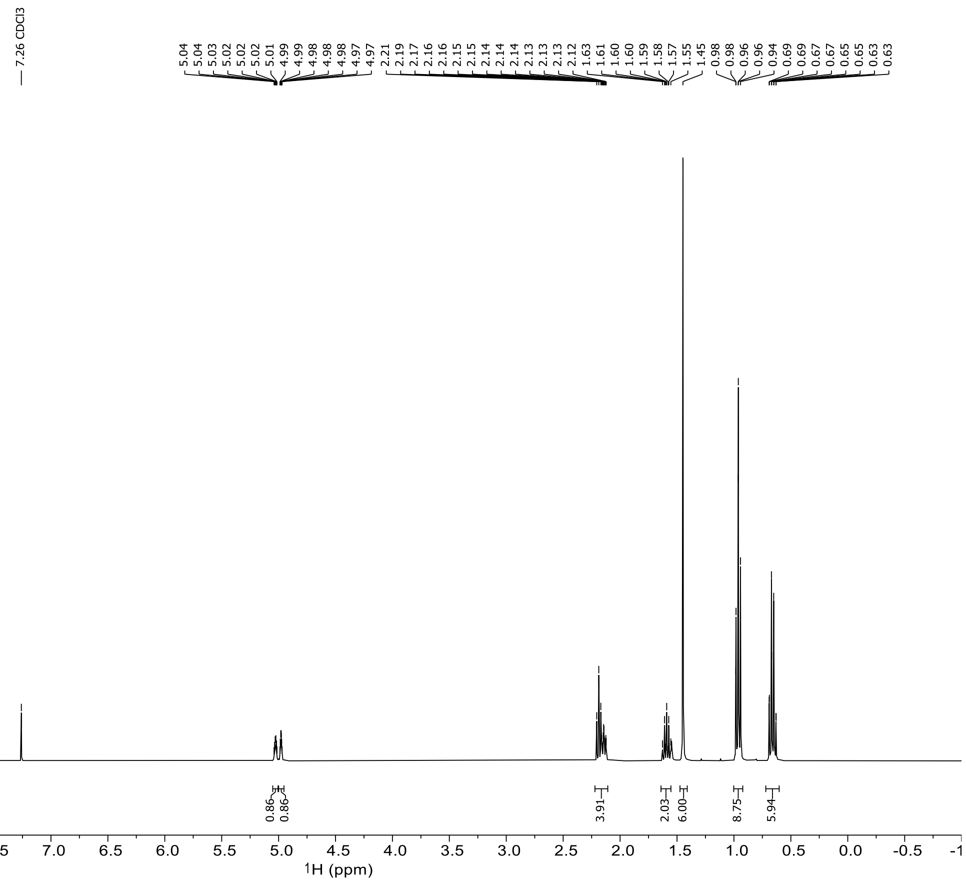
7.26 CDCl3
5.04 5.04 5.03 5.02 5.02 5.02 5.01 4.99 4.99 4.98 4.98 4.98 4.97 4.97 2.21 2.19 2.17 2.16 2.16 2.15 2.15 2.14 2.14 2.14 2.13 2.13 2.13 2.12 1.63 1.61 1.60 1.59 1.58 1.57 1.55 1.45 0.98 0.98 0.96 0.96 0.94 0.69 0.69 0.67 0.67 0.65 0.65 0.63 0.63
0.86 0.86 3.91 2.03 6.00 8.75 5.94
1H (ppm)

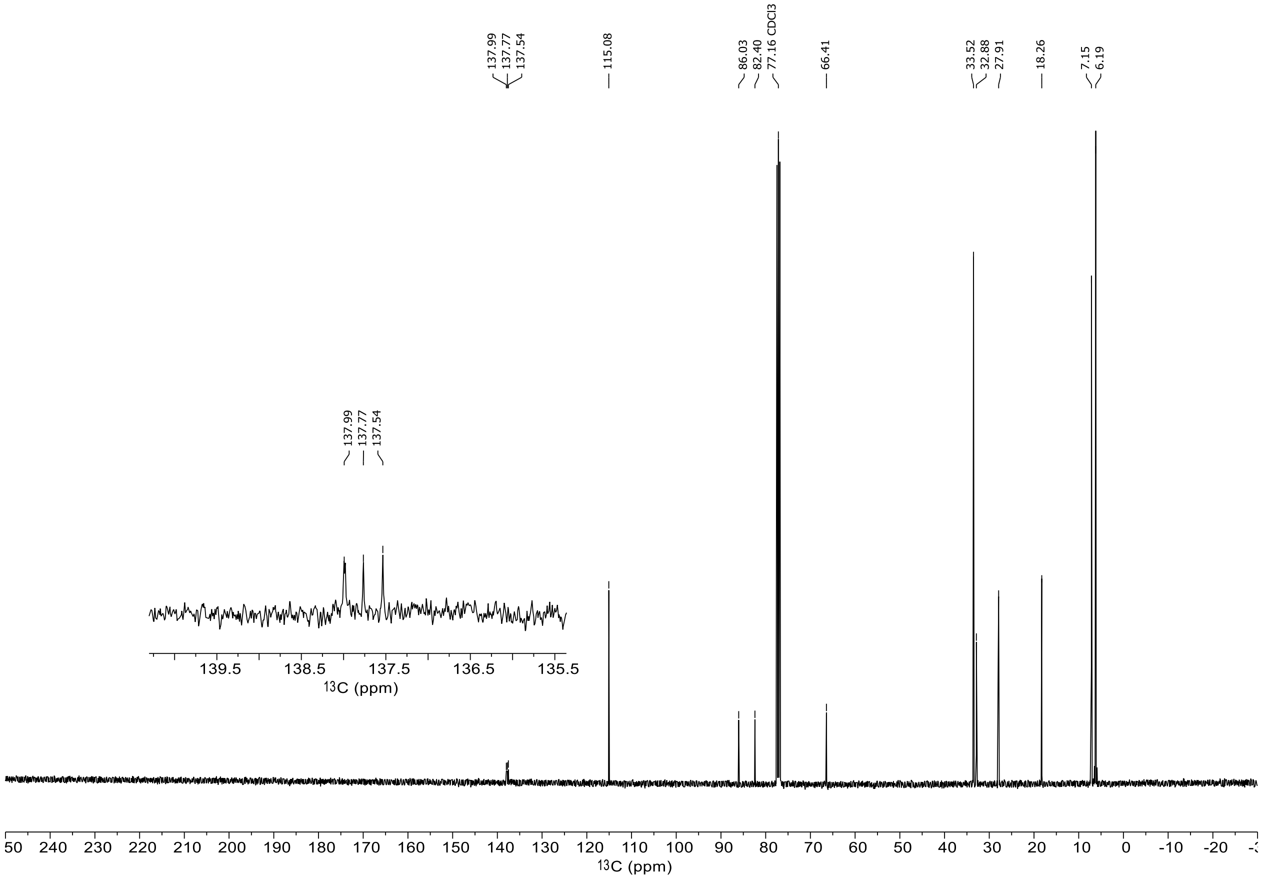
137.99 137.77 137.54
115.08
86.03 82.40 77.16 CDCl3
66.41
33.52 32.88 27.91
18.26
7.15 6.19
139.5 138.5 137.5 136.5 135.5
13C (ppm)

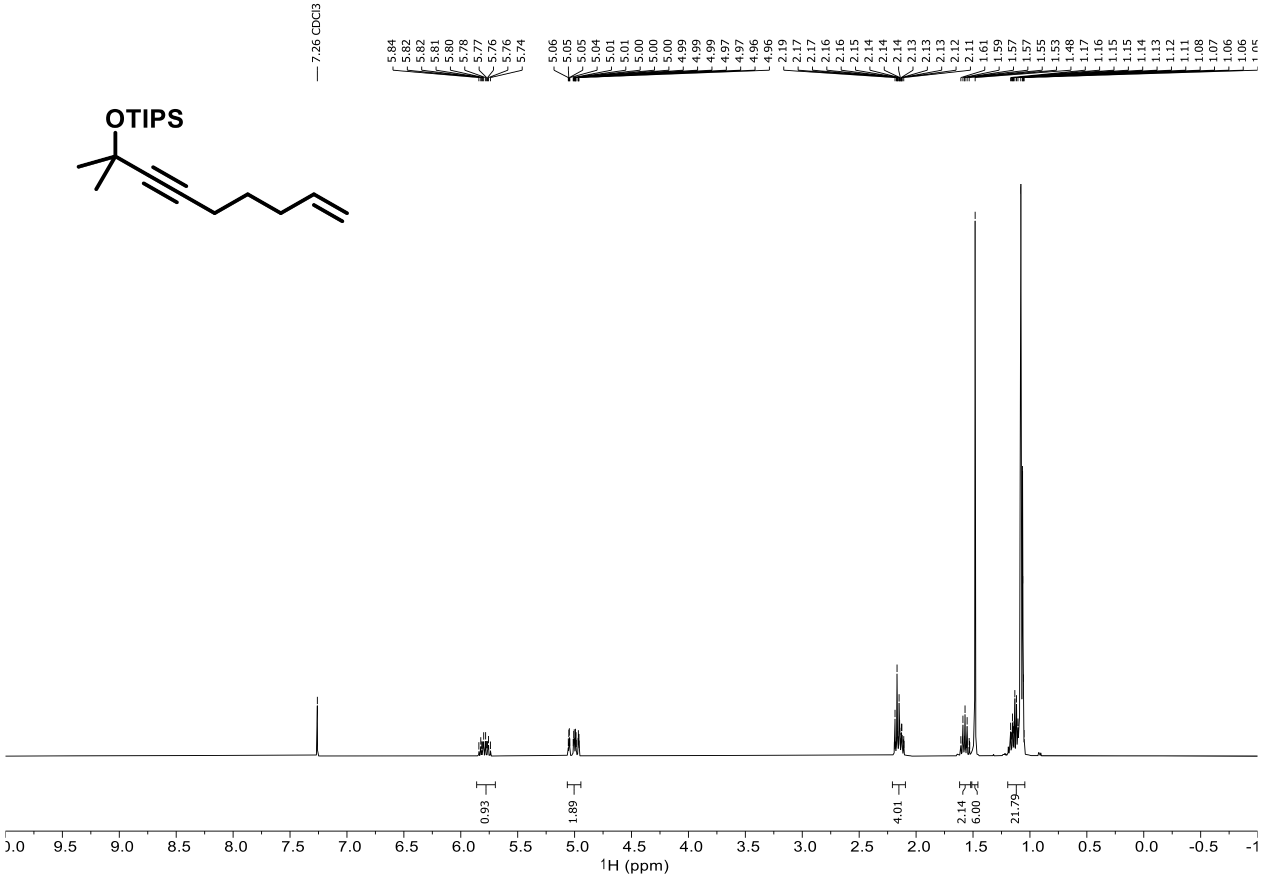
7.26 CDCl3
5.84 5.82 5.82 5.81 5.80 5.78 5.77 5.76 5.76 5.74
5.06 5.05 5.05 5.04 5.01 5.01 5.00 5.00 4.99 4.99 4.99 4.97 4.97 4.96 4.96
2.19 2.17 2.17 2.16 2.16 2.15 2.14 2.14 2.14 2.13 2.13 2.13 2.12 2.11
1.61 1.59 1.57 1.57 1.57 1.55 1.53 1.48 1.17 1.16 1.15 1.15 1.14 1.13 1.12 1.11 1.08 1.07 1.06 1.06
OTIPS
0.93
1.89
4.01
2.14
6.00
21.79
9.5 9.0 8.5 8.0 7.5 7.0 6.5 6.0 5.5 5.0 4.5 4.0 3.5 3.0 2.5 2.0 1.5 1.0 0.5 0.0 -0.5
1H (ppm)

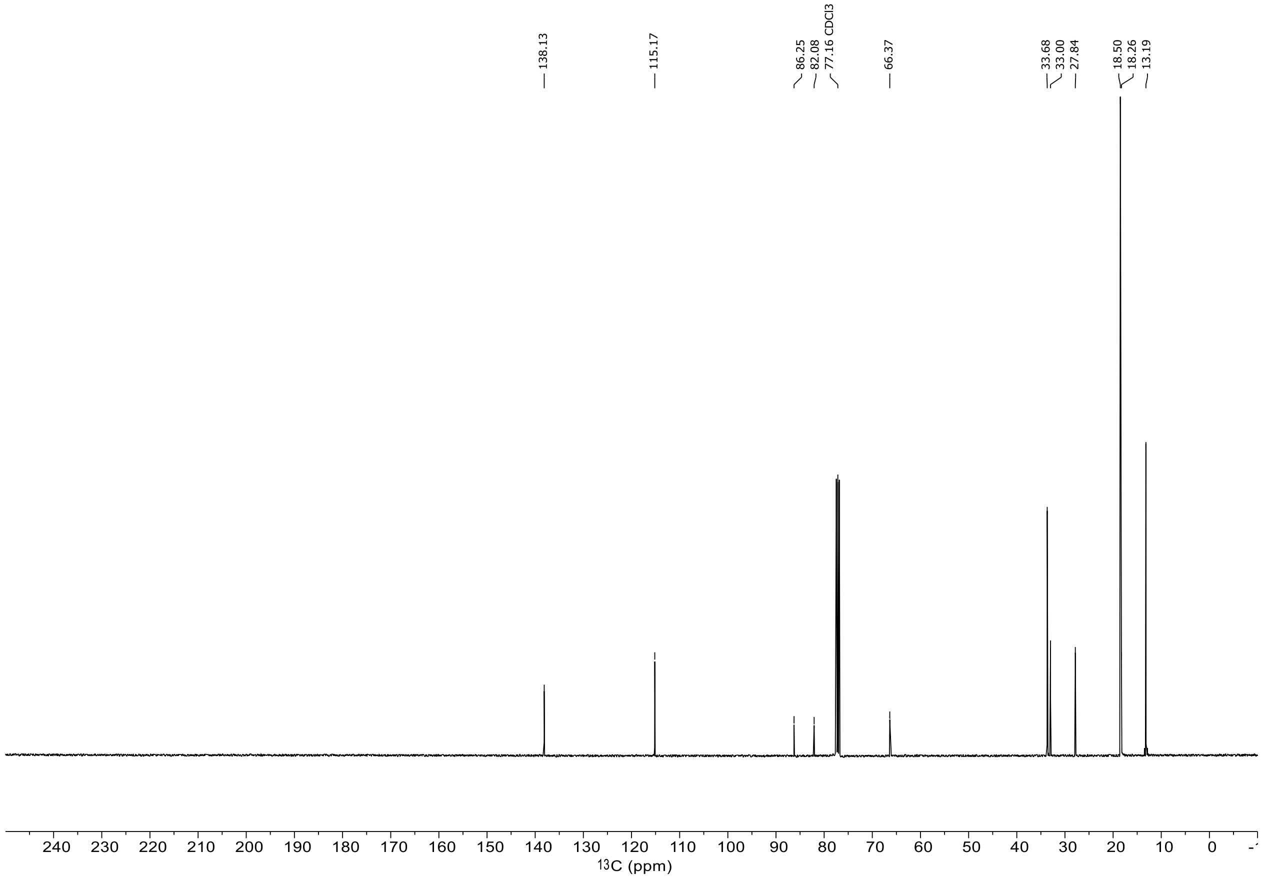
138.13
115.17
86.25
82.08
77.16 CDCl3
66.37
33.68
33.00
27.84
18.50
18.26
13.19
240 230 220 210 200 190 180 170 160 150 140 130 120 110 100 90 80 70 60 50 40 30 20 10 0
13C (ppm)

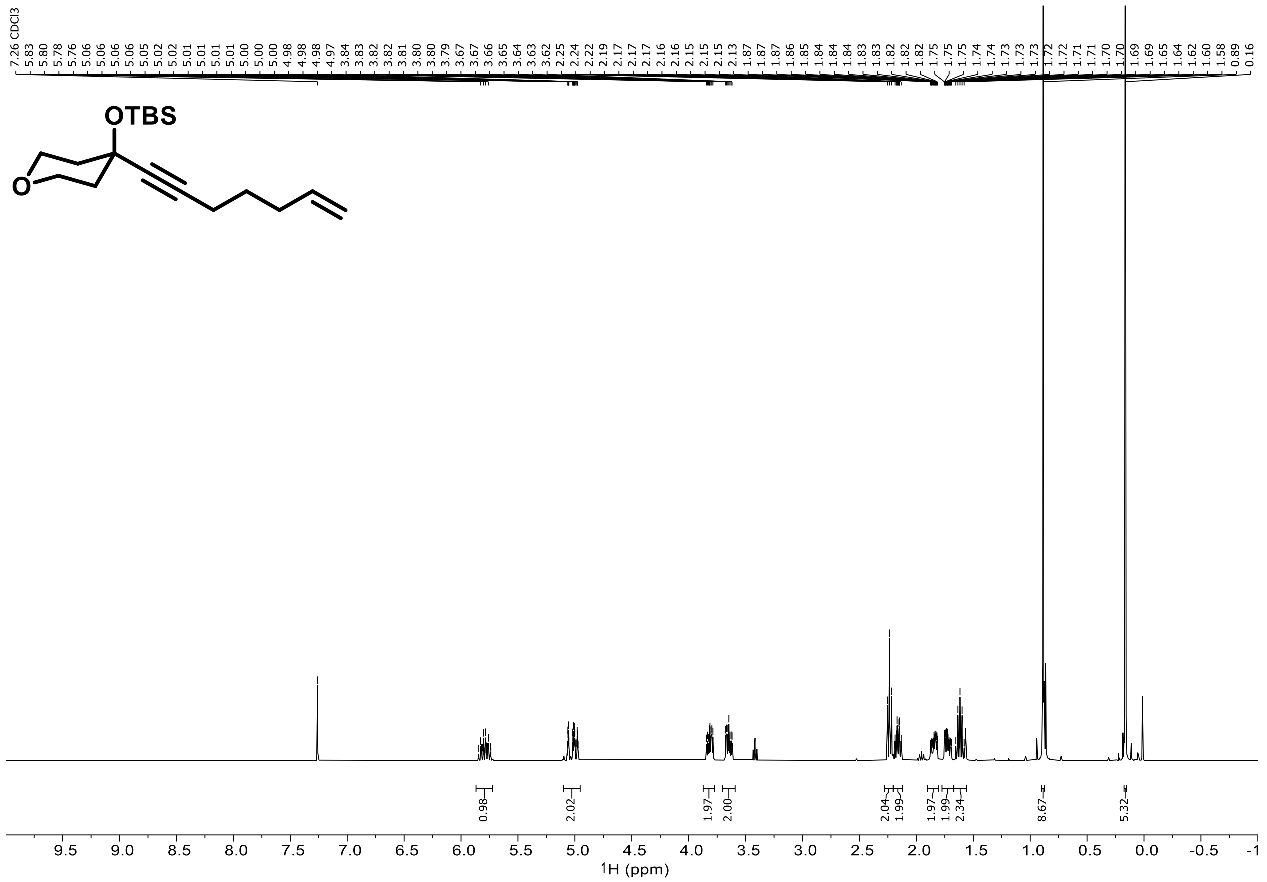
OTBS
O
0.98
2.02
1.97
2.00
2.04
1.99
1.97
1.99
2.34
8.67
5.32
1H (ppm)

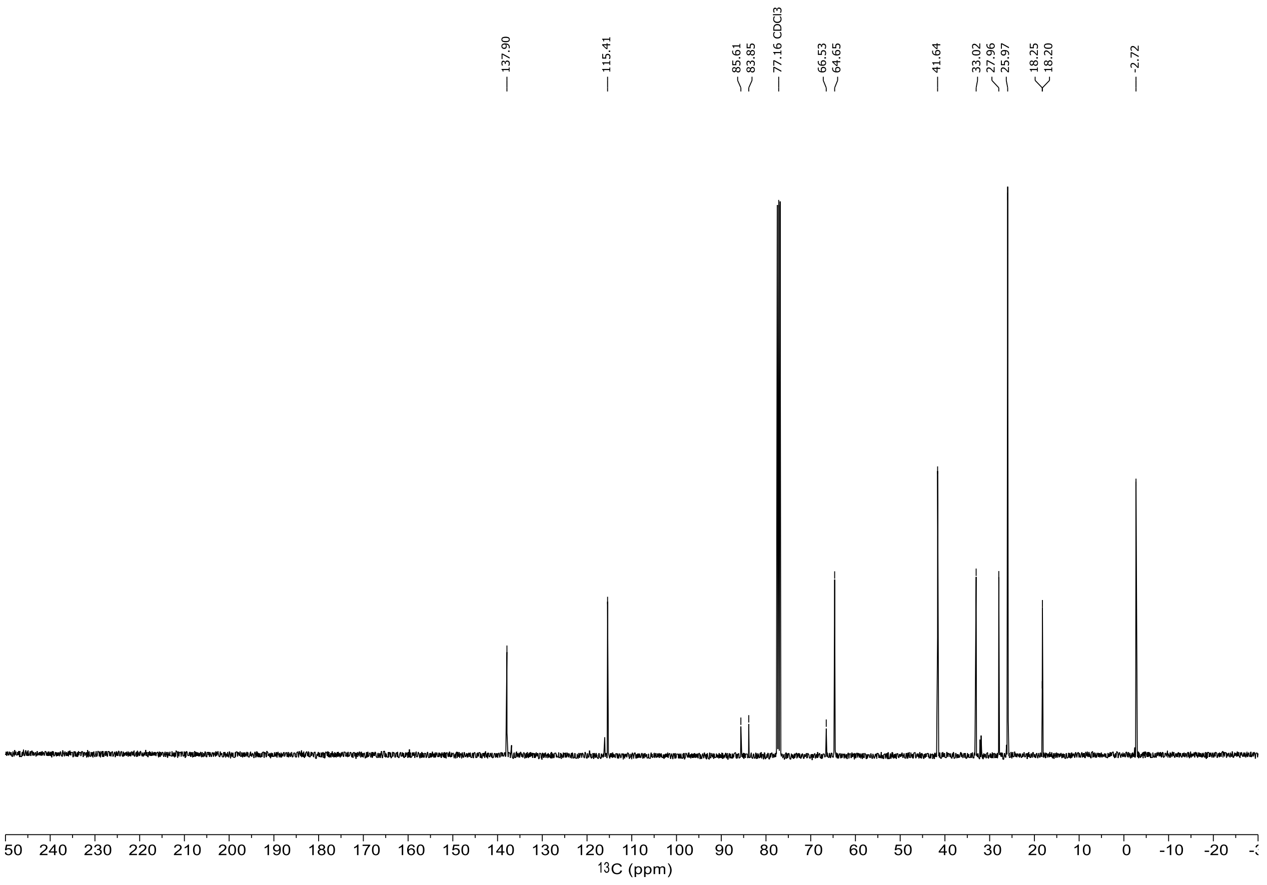
137.90
115.41
85.61
83.85
77.16 CDCl3
66.53
64.65
41.64
33.02
27.96
25.97
18.25
18.20
-2.72
13C (ppm)

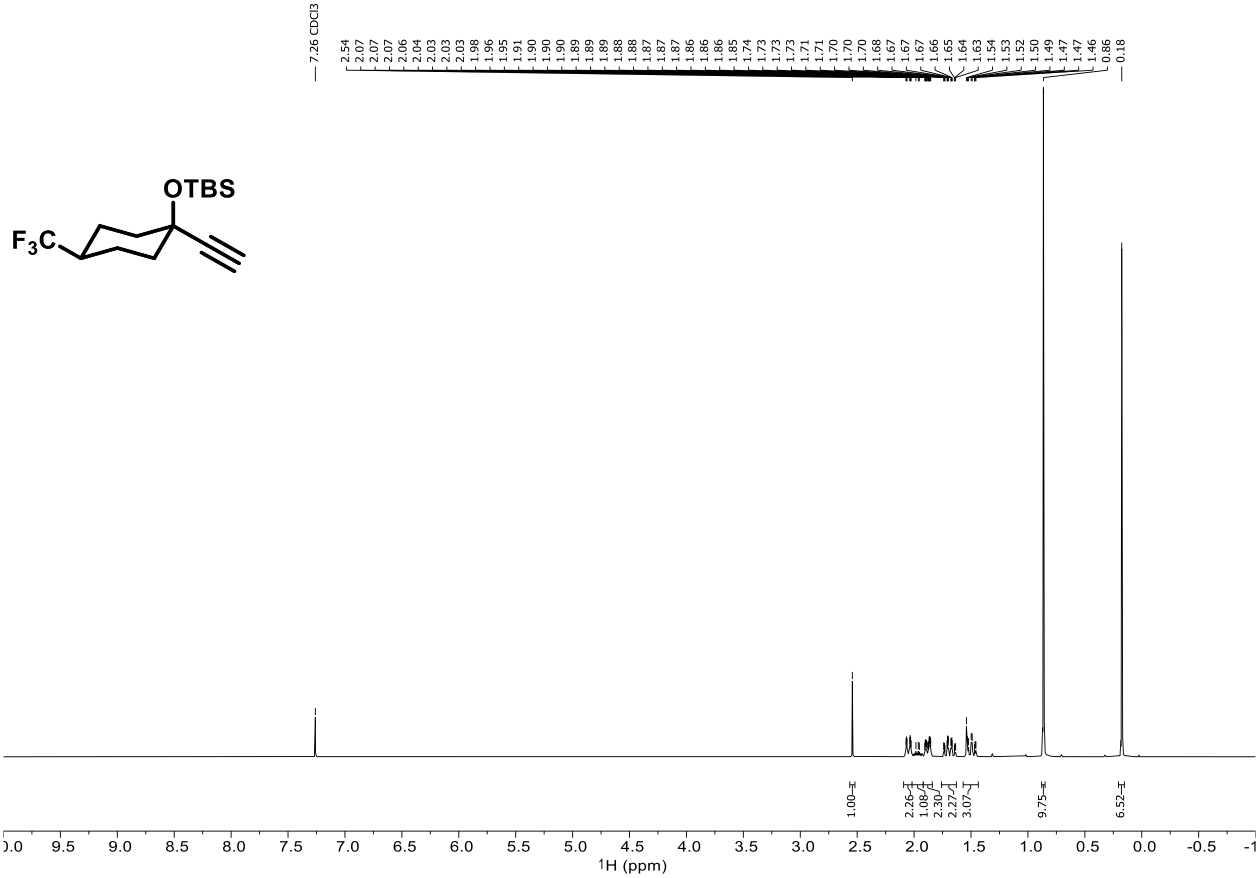
OTBS
F_3C
7.26 CDCl3
2.54 2.07 2.07 2.07 2.06 2.04 2.03 2.03 2.03 1.98 1.96 1.95 1.91 1.90 1.90 1.90 1.89 1.89 1.89 1.88 1.88 1.87 1.87 1.86 1.86 1.86 1.85 1.74 1.73 1.73 1.73 1.71 1.71 1.70 1.70 1.70 1.68 1.67 1.67 1.67 1.66 1.65 1.64 1.63 1.54 1.53 1.52 1.50 1.49 1.47 1.47 1.46 0.86
0.18
1.00
2.26 1.08 2.30 2.27 3.07
9.75
6.52
1H (ppm)

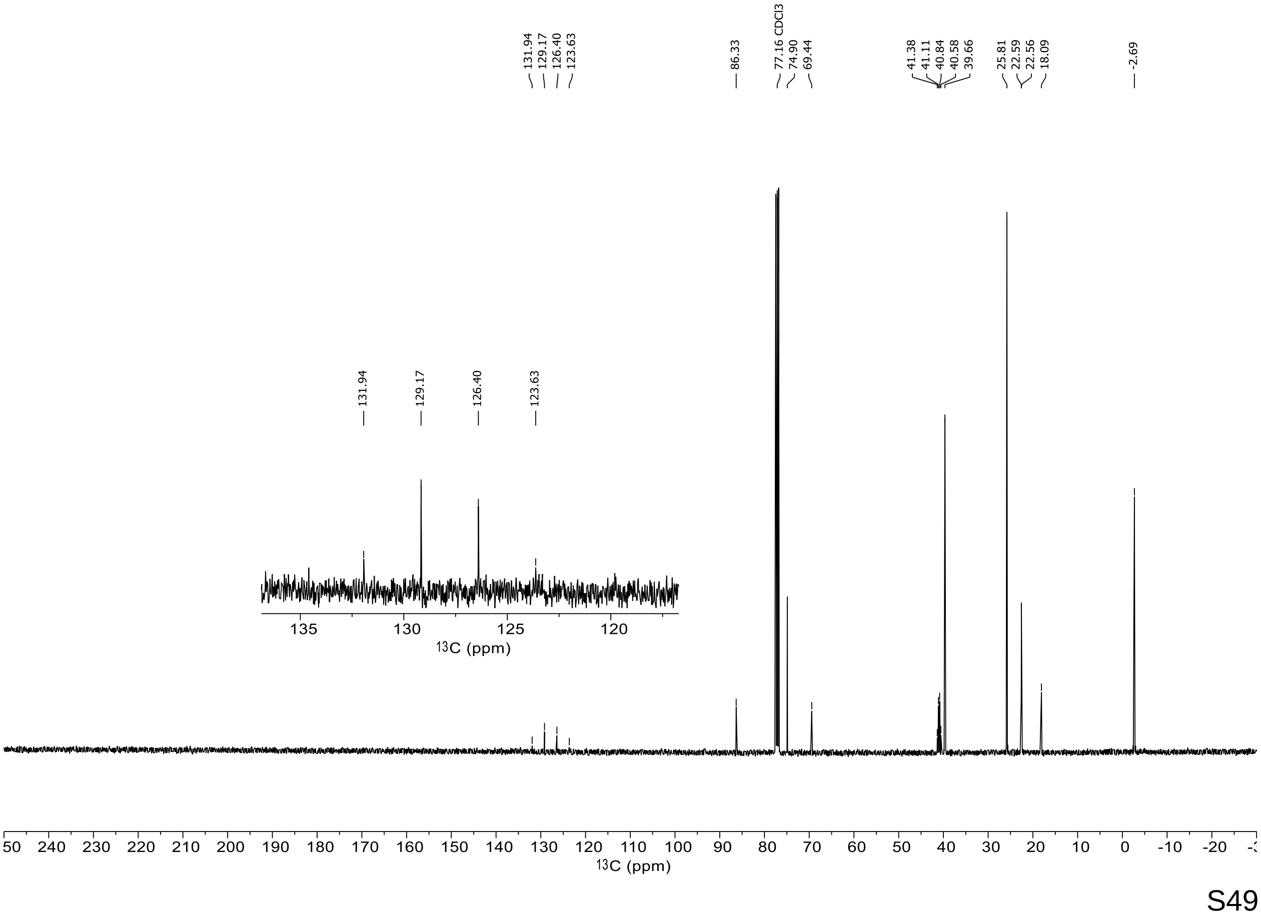
131.94 129.17 126.40 123.63
86.33
77.16 CDCl3 74.90 69.44
41.38 41.11 40.84 40.58 39.66
25.81 22.59 22.56 18.09
-2.69
13C (ppm)

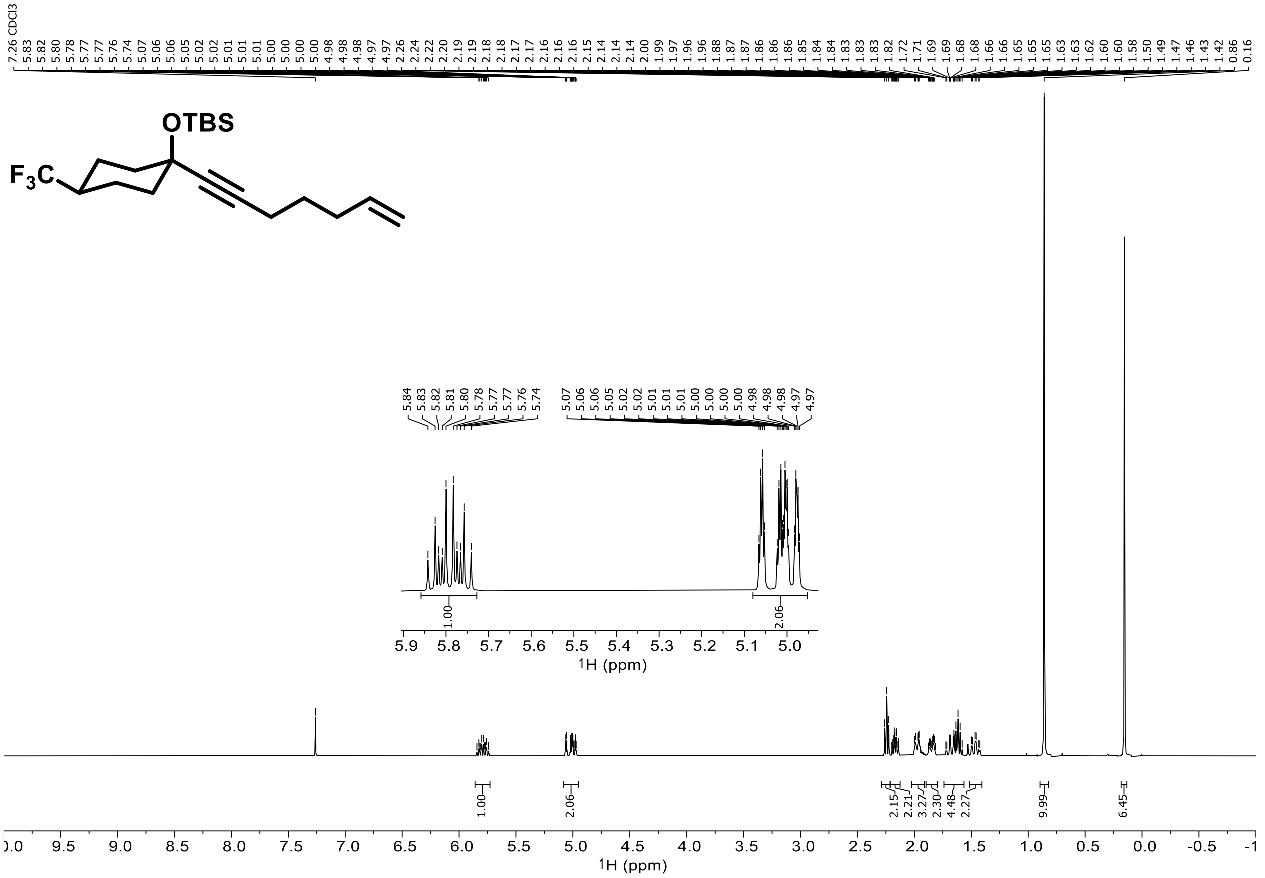

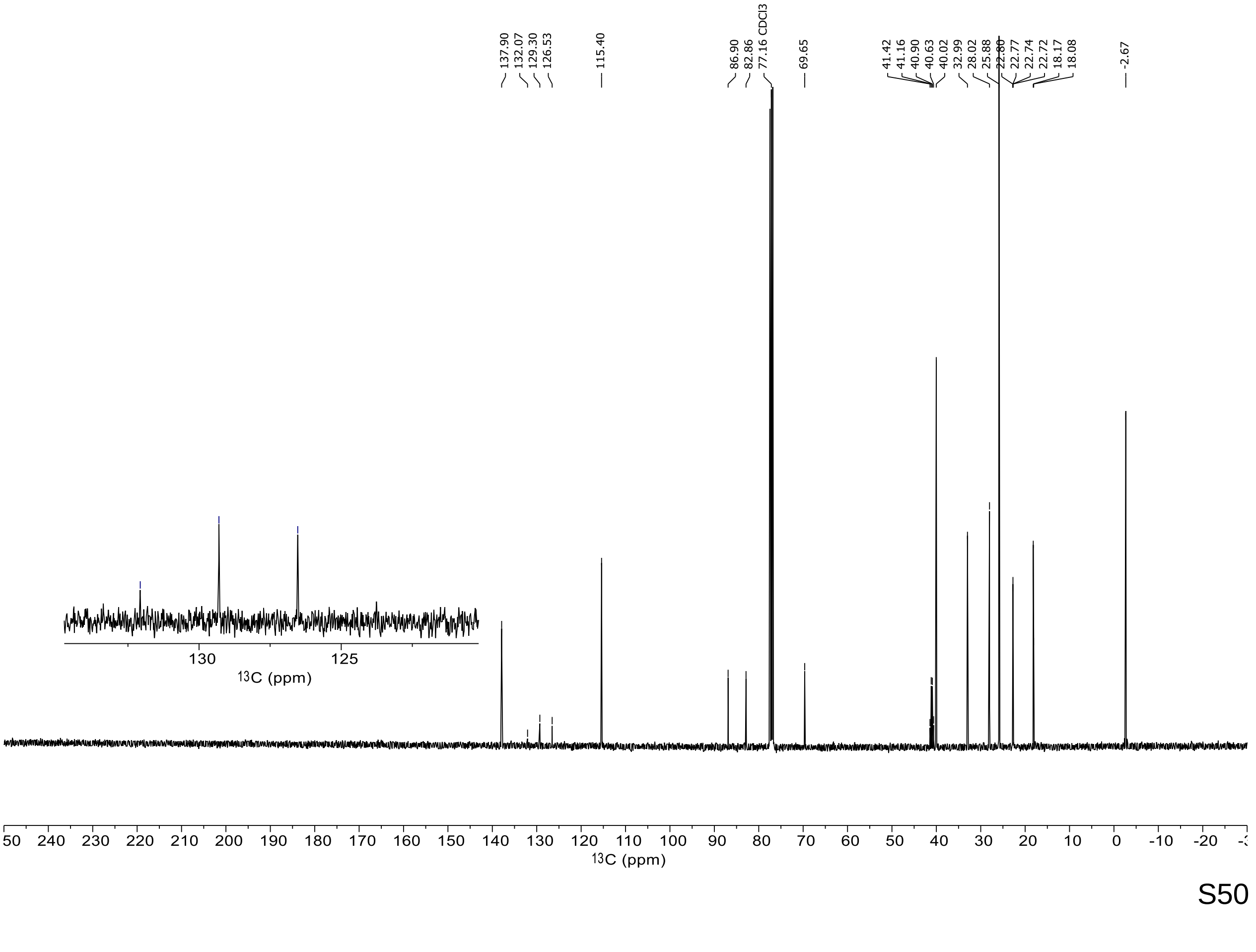

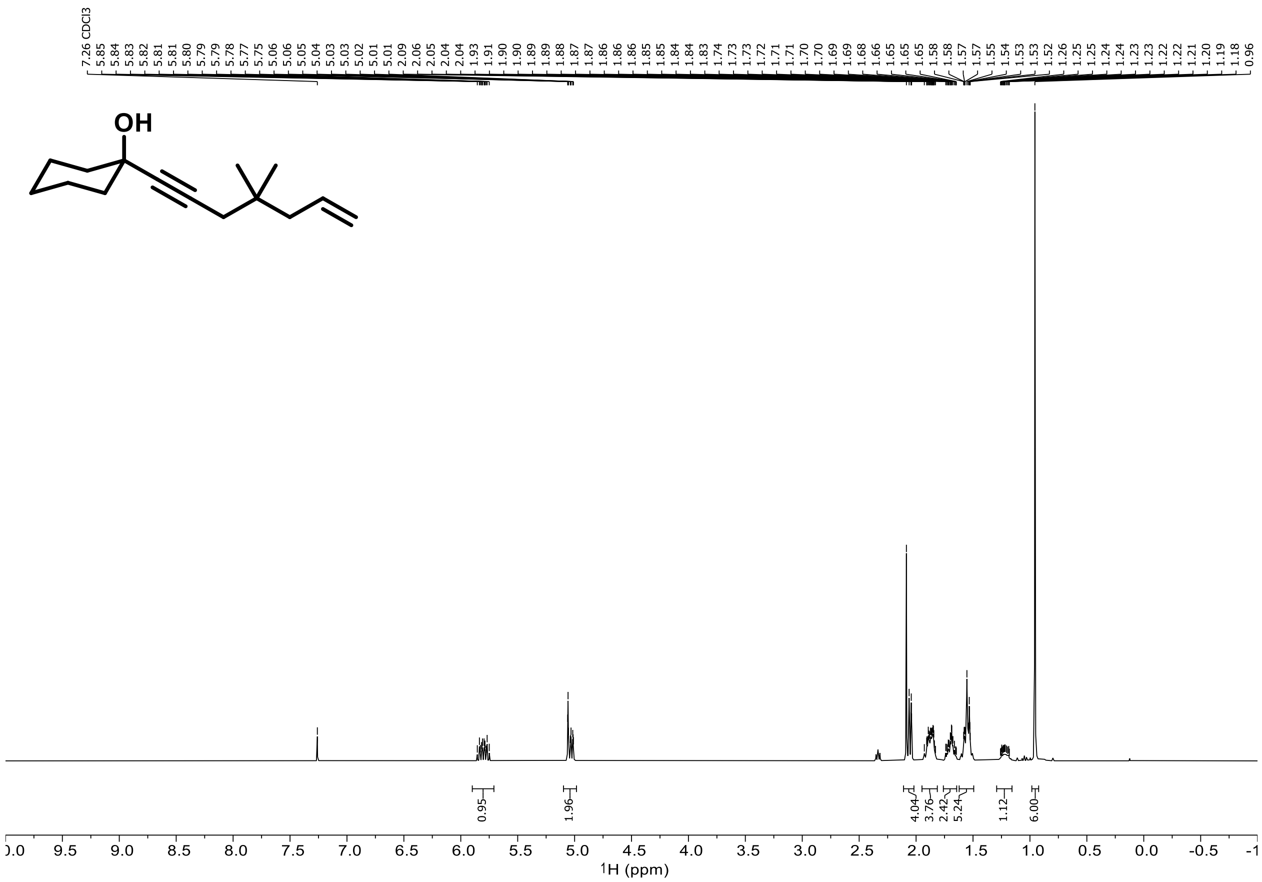
OH
0.95
1.96
4.04
3.76
2.42
5.24
1.12
6.00
1H (ppm)

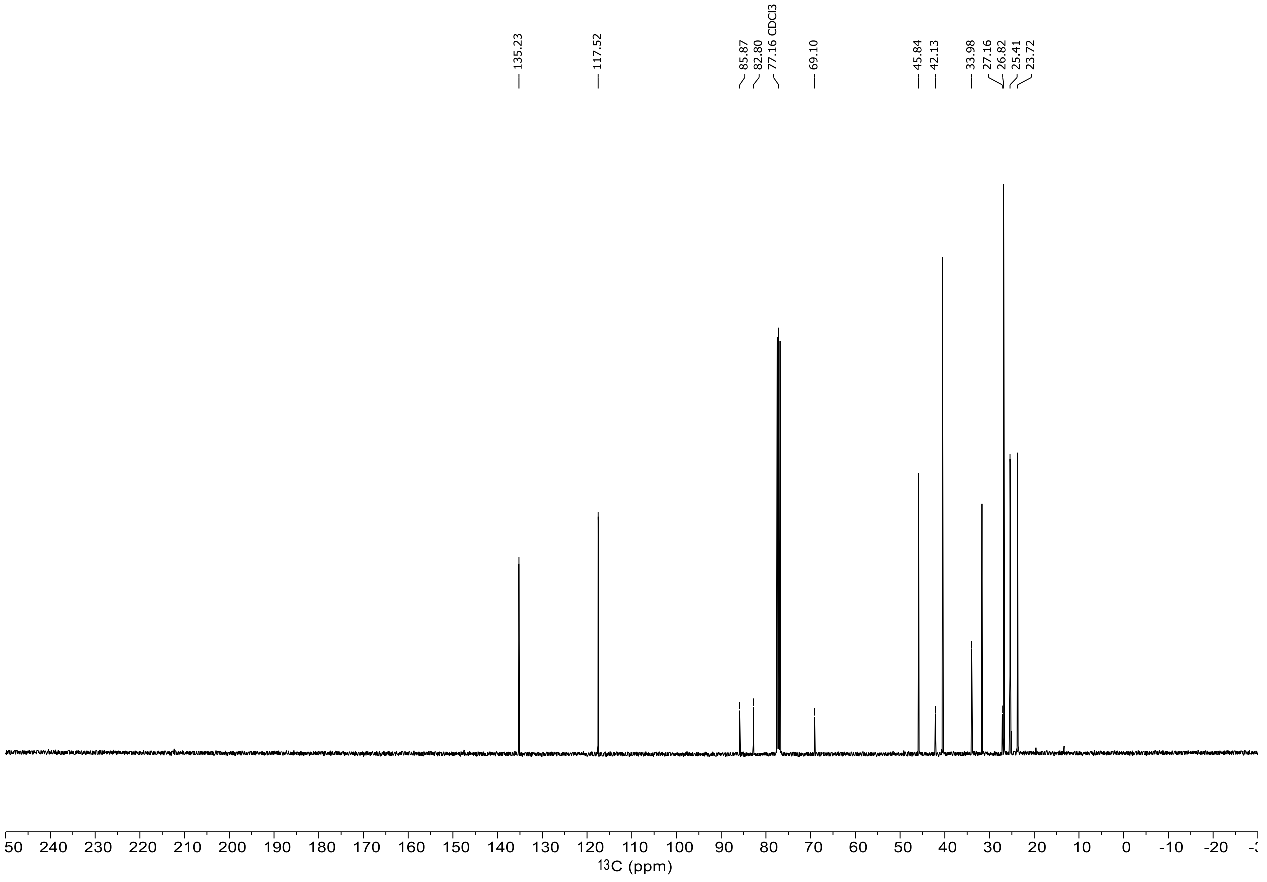
135.23
117.52
85.87
82.80
77.16 CDCl3
69.10
45.84
42.13
33.98
27.16
26.82
25.41
23.72
13C (ppm)

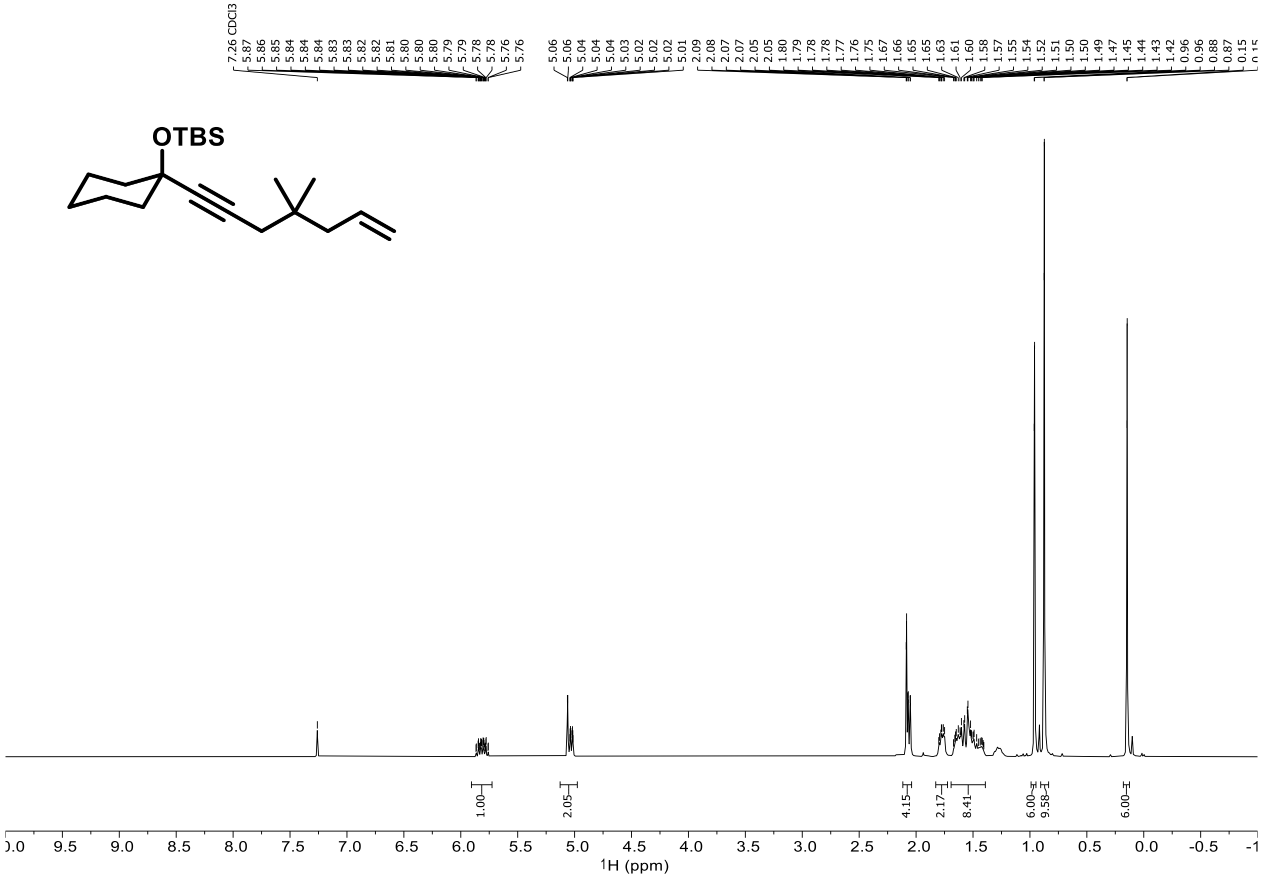
OTBS
7.26 CDCl3
1.00
2.05
4.15
2.17
8.41
6.00
9.58
6.00
1H (ppm)

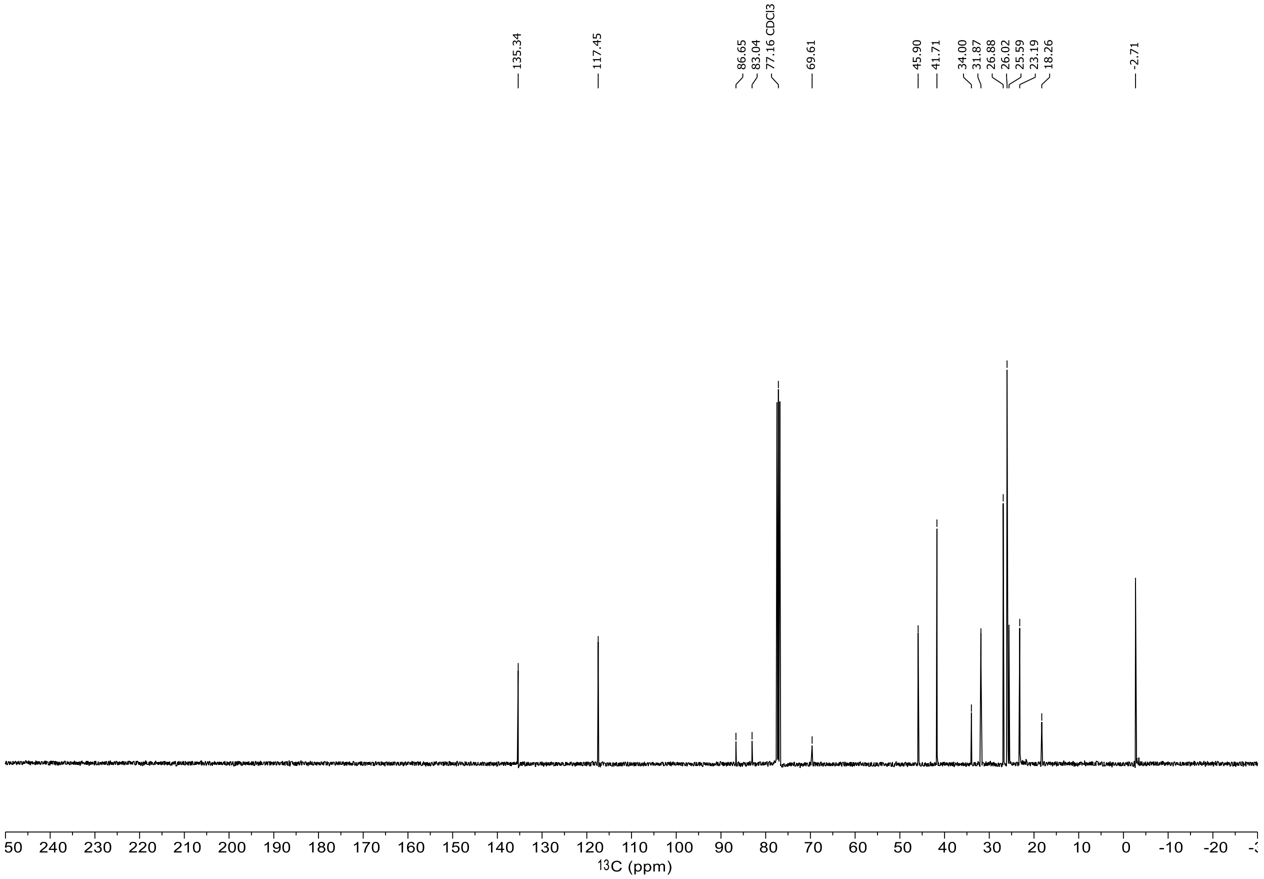
135.34
117.45
86.65
83.04
77.16 CDCl3
69.61
45.90
41.71
34.00
31.87
26.88
26.02
25.59
23.19
18.26
-2.71
13C (ppm)

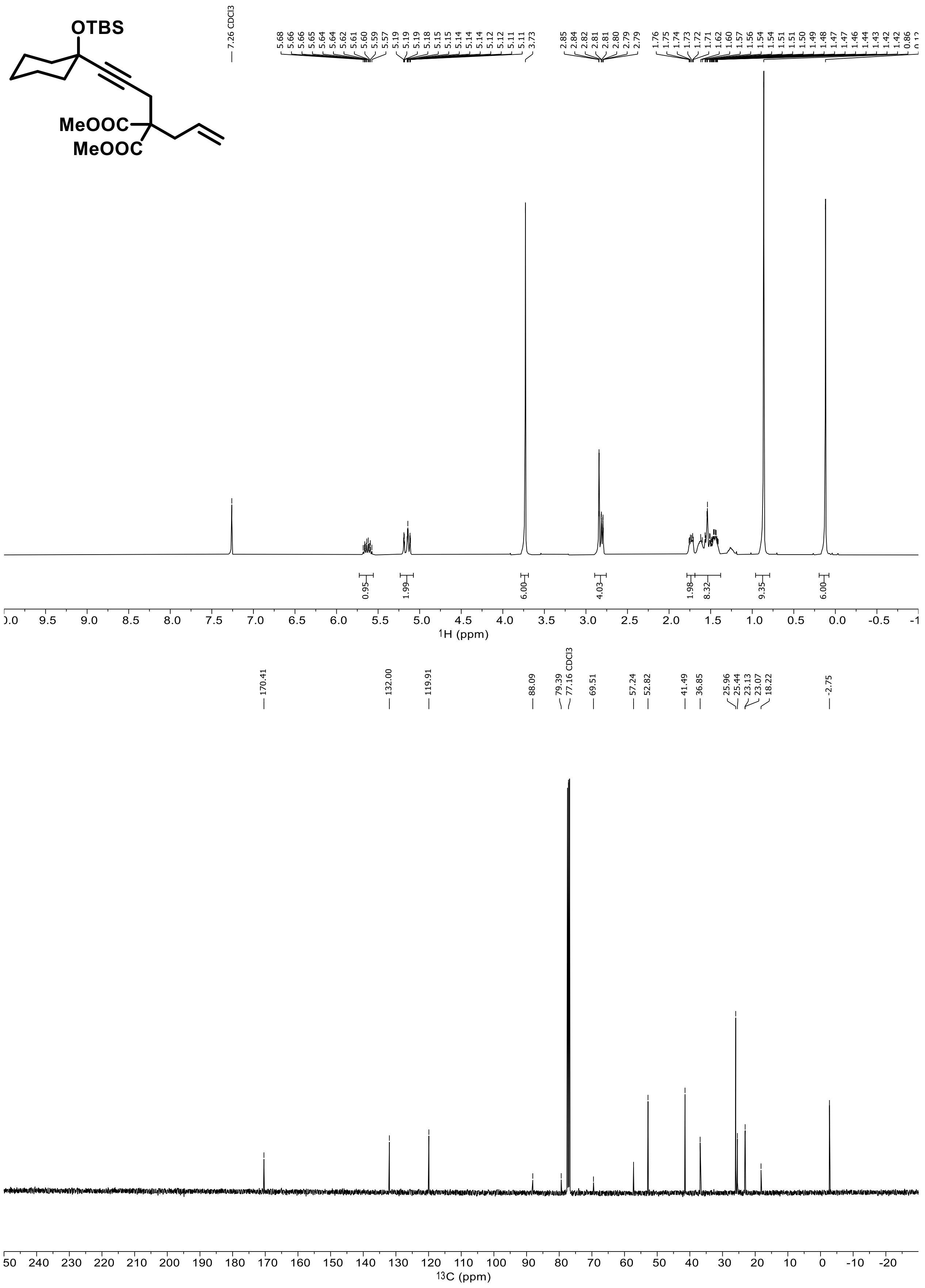
OTBS
MeOOC
MeOOC
7.26 CDCl3
^{1}H (ppm)
170.41
132.00
119.91
88.09
79.39
77.16 CDCl3
69.51
57.24
52.82
41.49
36.85
25.96
25.44
23.13
23.07
18.22
-2.75
^{13}C (ppm)

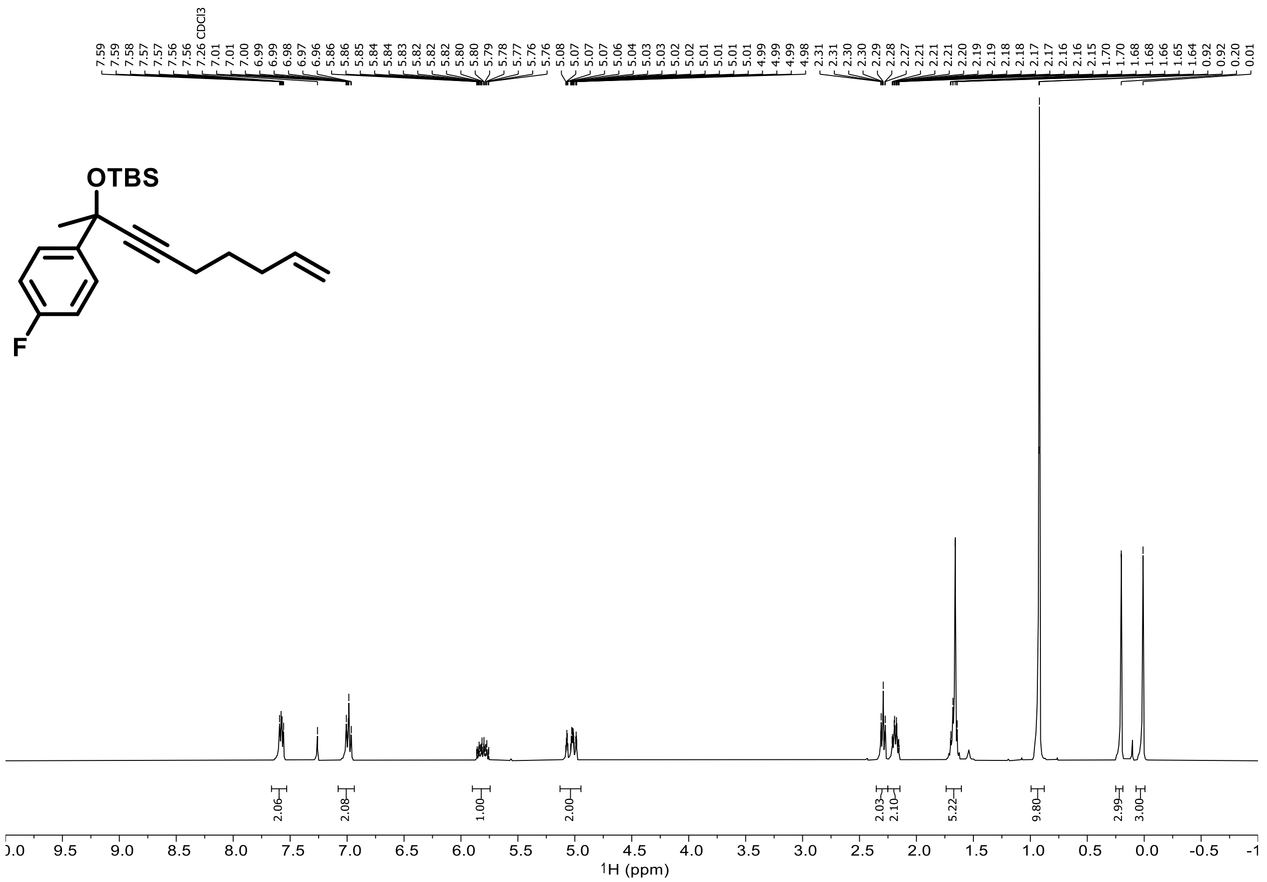

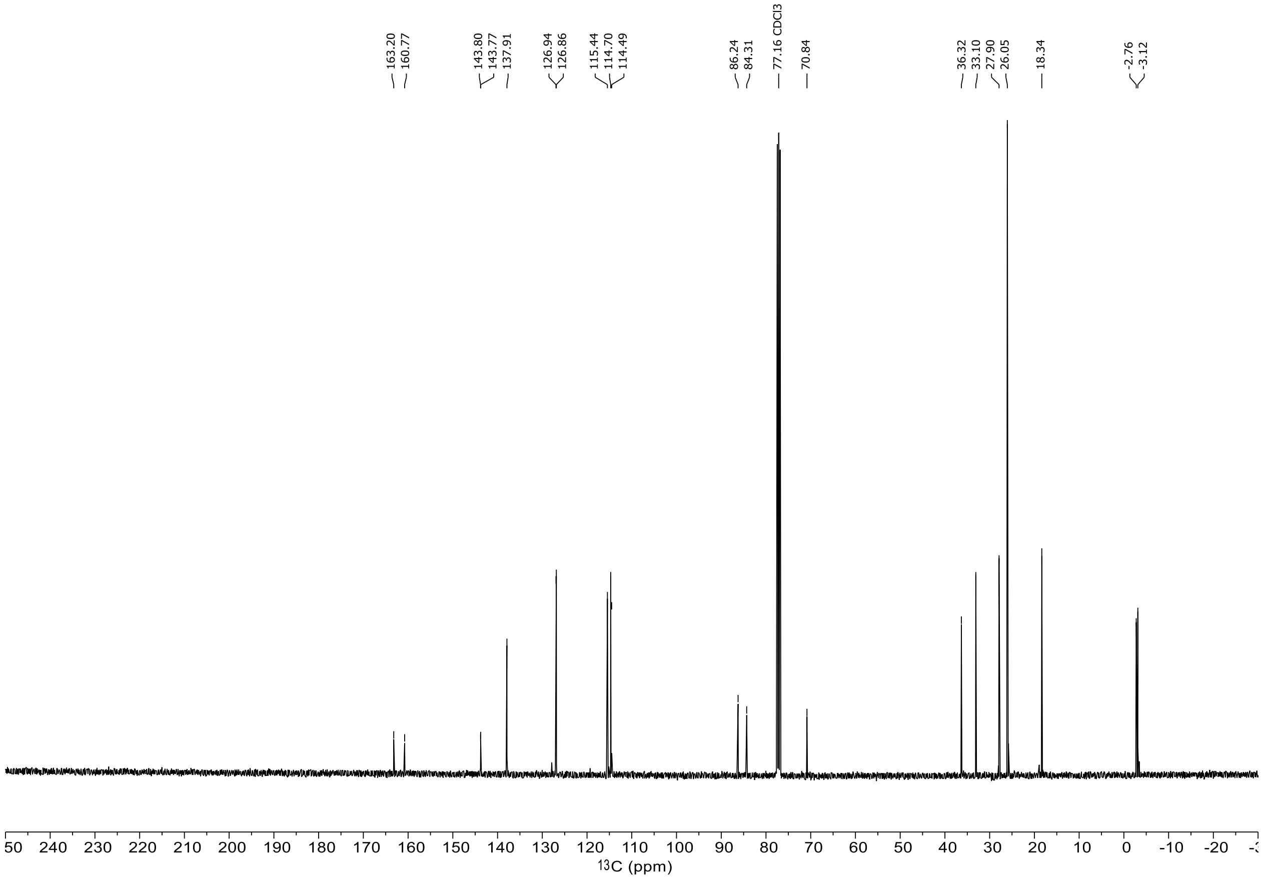

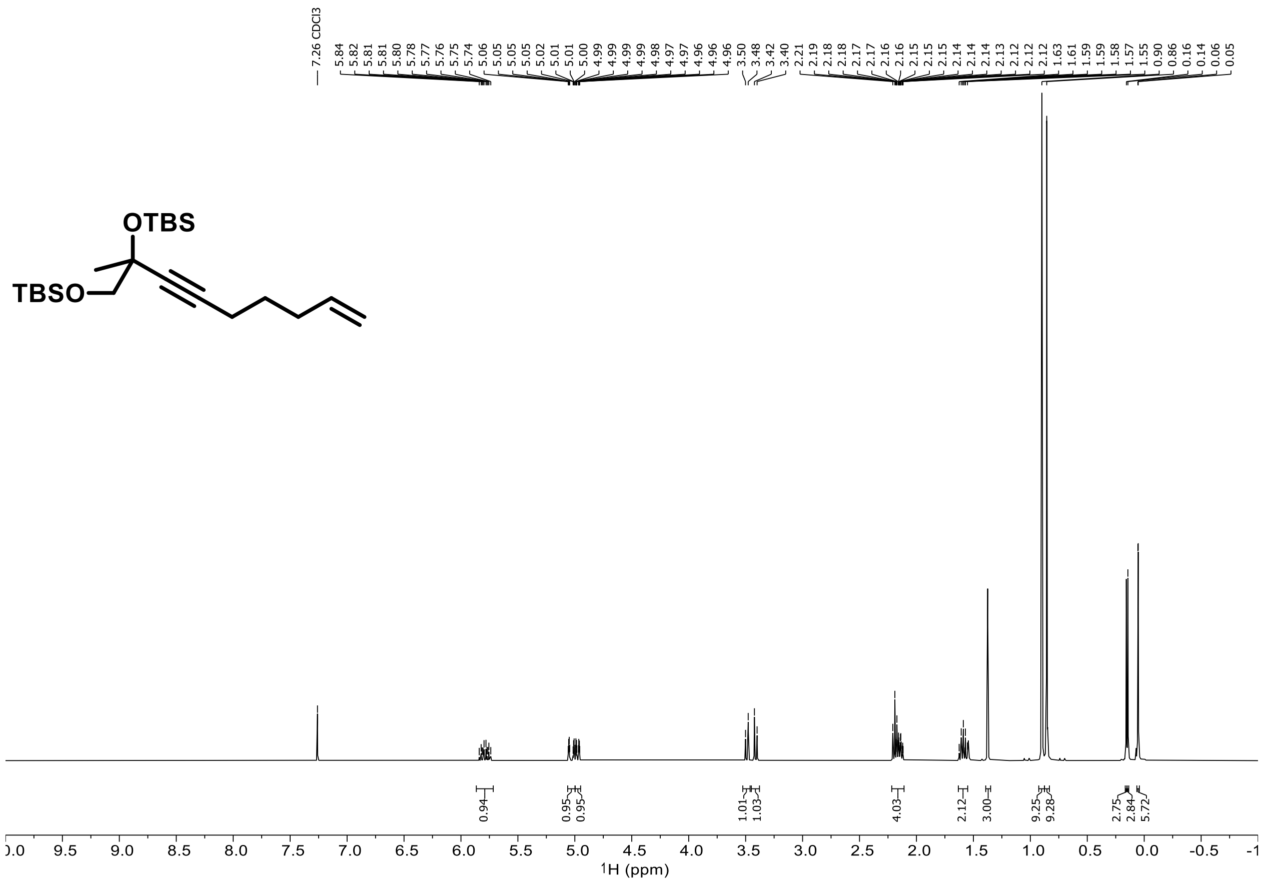
OTBS
TBSO
7.26 CDCl3
0.94
0.95
0.95
1.01
1.03
4.03
2.12
3.00
9.25
9.28
2.75
2.84
5.72
1H (ppm)

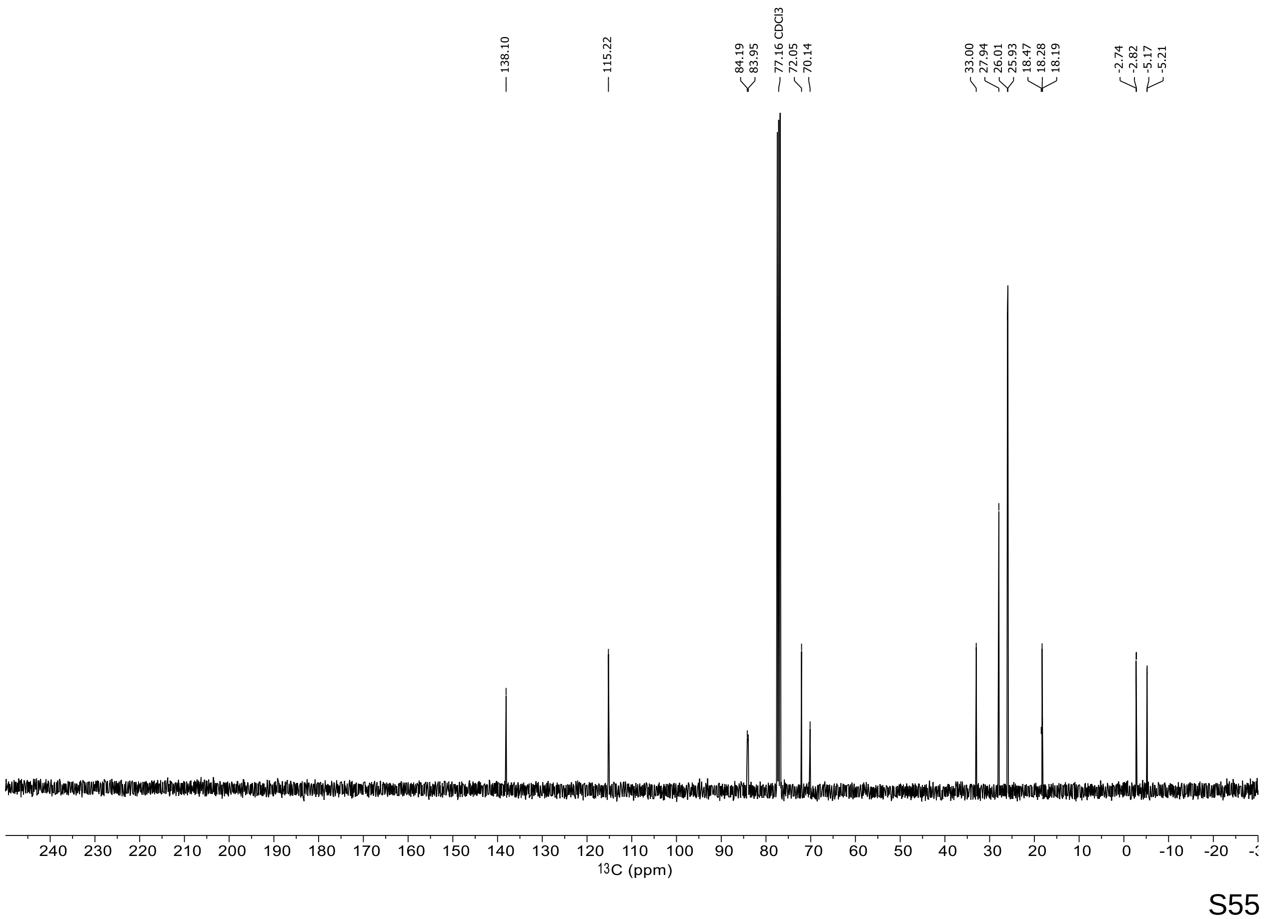
138.10
115.22
84.19
83.95
77.16 CDCl3
72.05
70.14
33.00
27.94
26.01
25.93
18.47
18.28
18.19
-2.74
-2.82
-5.17
-5.21
13C (ppm)

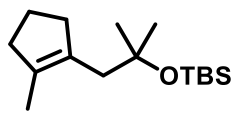
OTBS

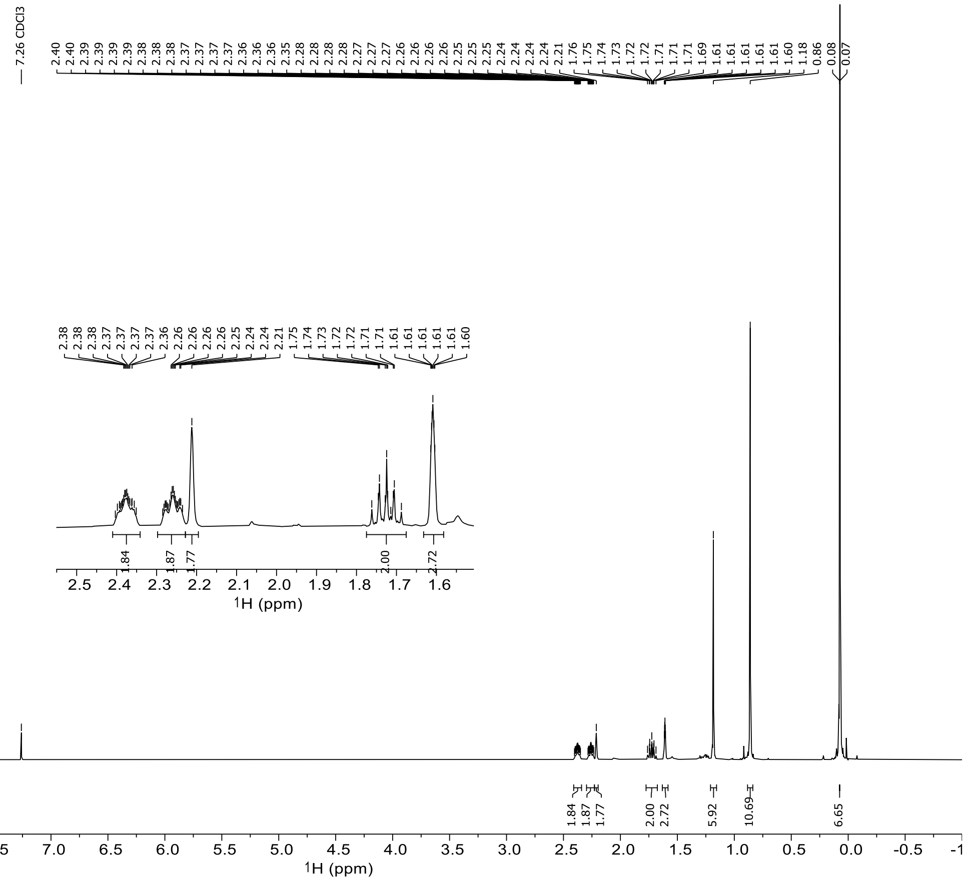
7.26 CDCl3
1.84
1.87
1.77
2.00
2.72
5.92
10.69
6.65
1H (ppm)

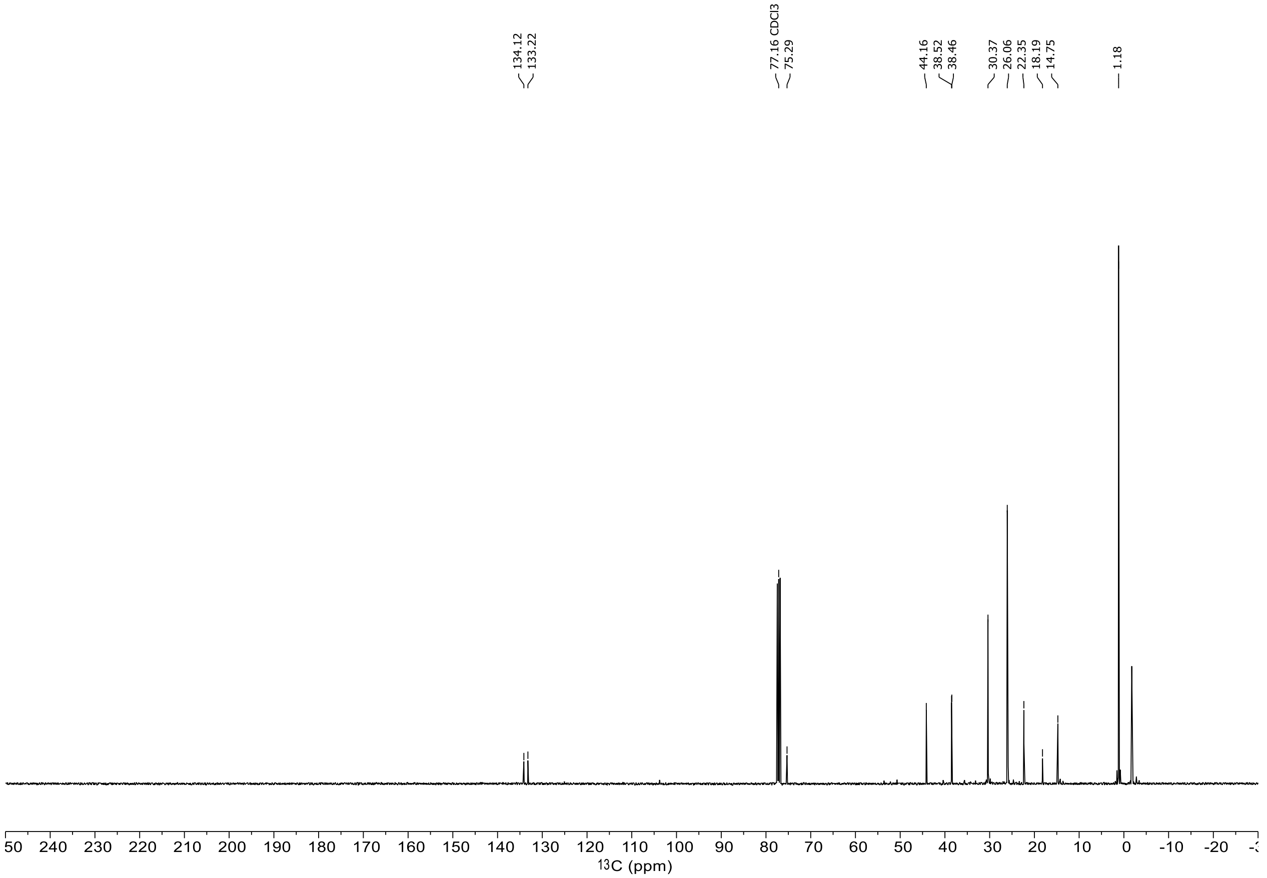
134.12
133.22
77.16 CDCl3
75.29
44.16
38.52
38.46
30.37
26.06
22.35
18.19
14.75
1.18
13C (ppm)

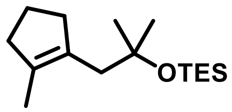
OTES

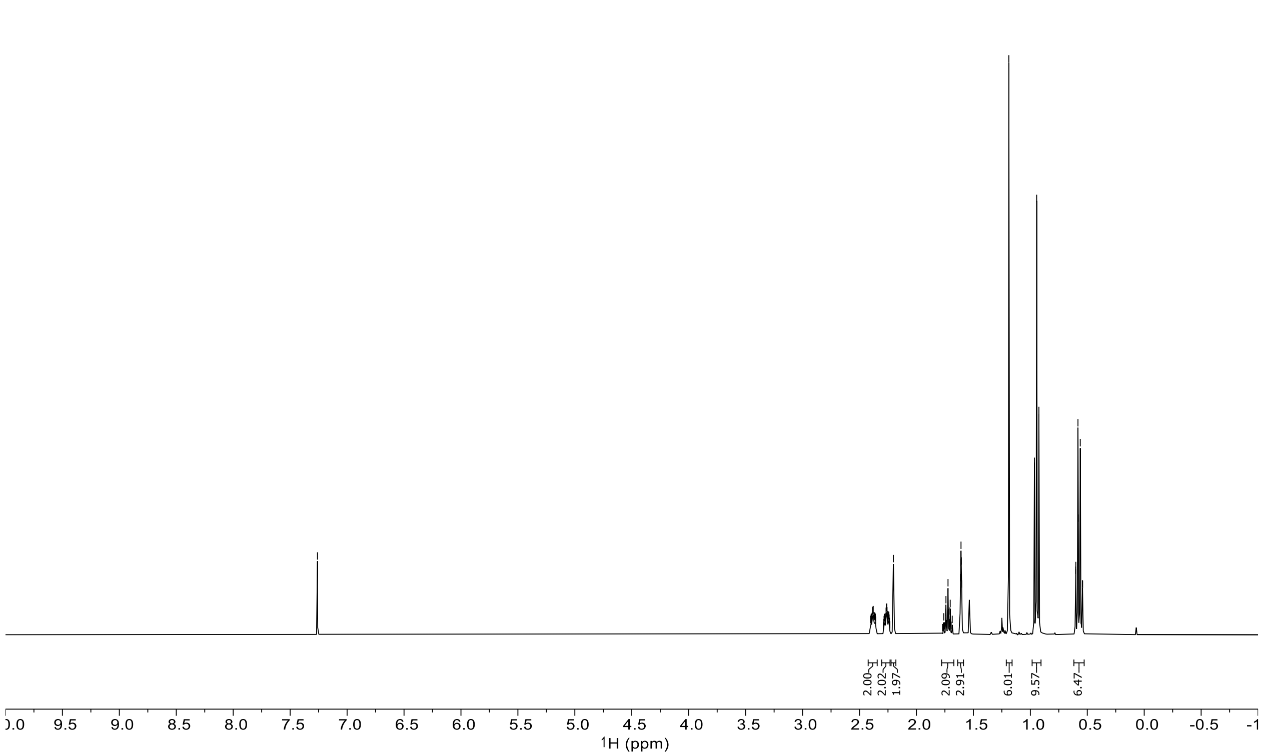
7.26 CDCl3
2.40
2.40
2.40
2.40
2.39
2.39
2.39
2.38
2.38
2.37
2.37
2.37
2.36
2.36
2.29
2.29
2.28
2.28
2.28
2.27
2.27
2.27
2.26
2.26
2.26
2.26
2.25
2.25
2.25
2.24
2.24
2.24
2.24
2.23
2.20
1.74
1.74
1.72
1.72
1.71
1.70
1.61
1.61
1.61
1.61
1.60
1.19
0.96
0.94
0.92
0.60
0.60
0.58
0.58
0.56
0.54
2.00
2.02
1.97
2.09
2.91
6.01
9.57
6.47
9.5
9.0
8.5
8.0
7.5
7.0
6.5
6.0
5.5
5.0
4.5
4.0
3.5
3.0
2.5
2.0
1.5
1.0
0.5
0.0
-0.5
1H (ppm)

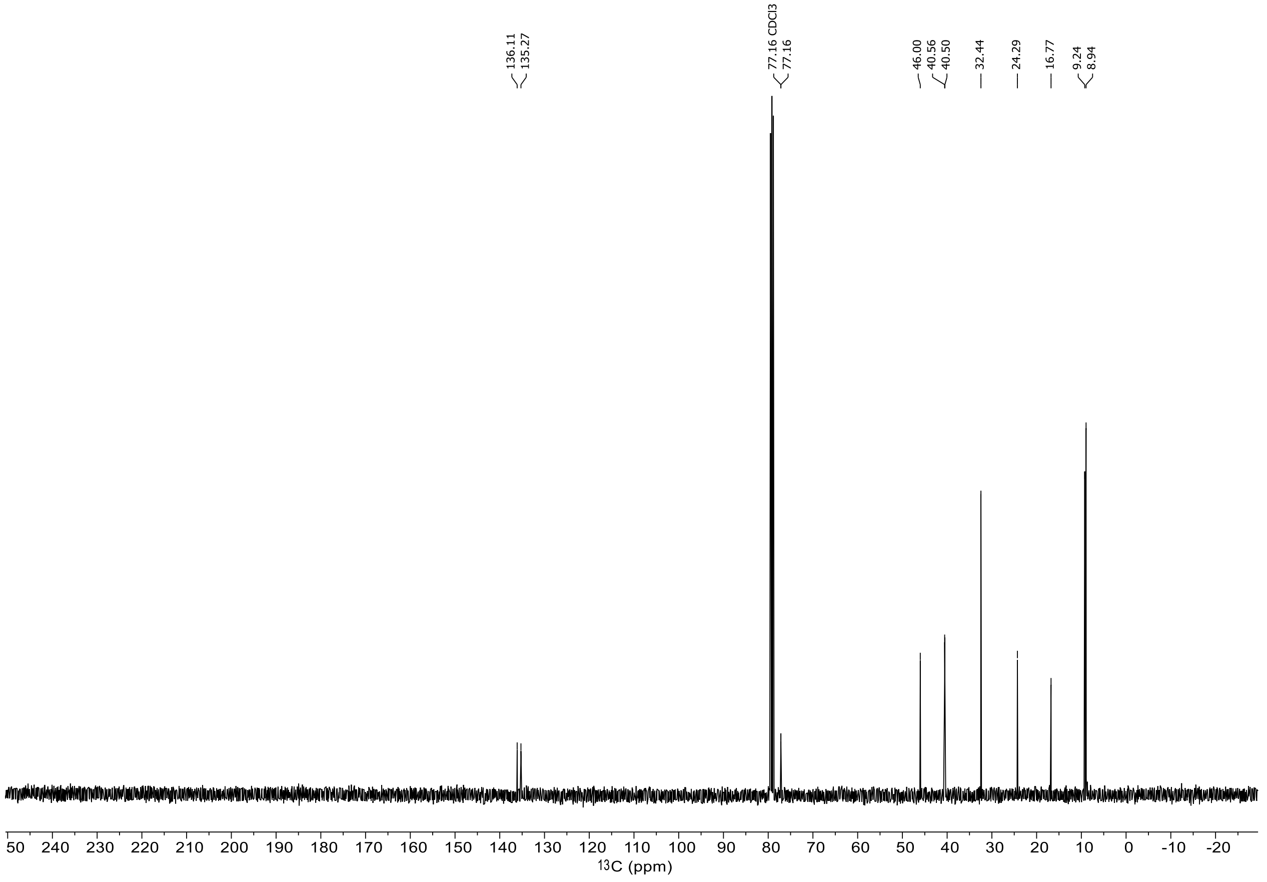
136.11
135.27
77.16 CDCl3
77.16
46.00
40.56
40.50
32.44
24.29
16.77
9.24
8.94
240
230
220
210
200
190
180
170
160
150
140
130
120
110
100
90
80
70
60
50
40
30
20
10
0
-10
-20
13C (ppm)

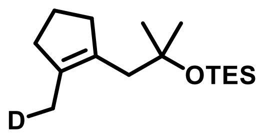
OTES
D

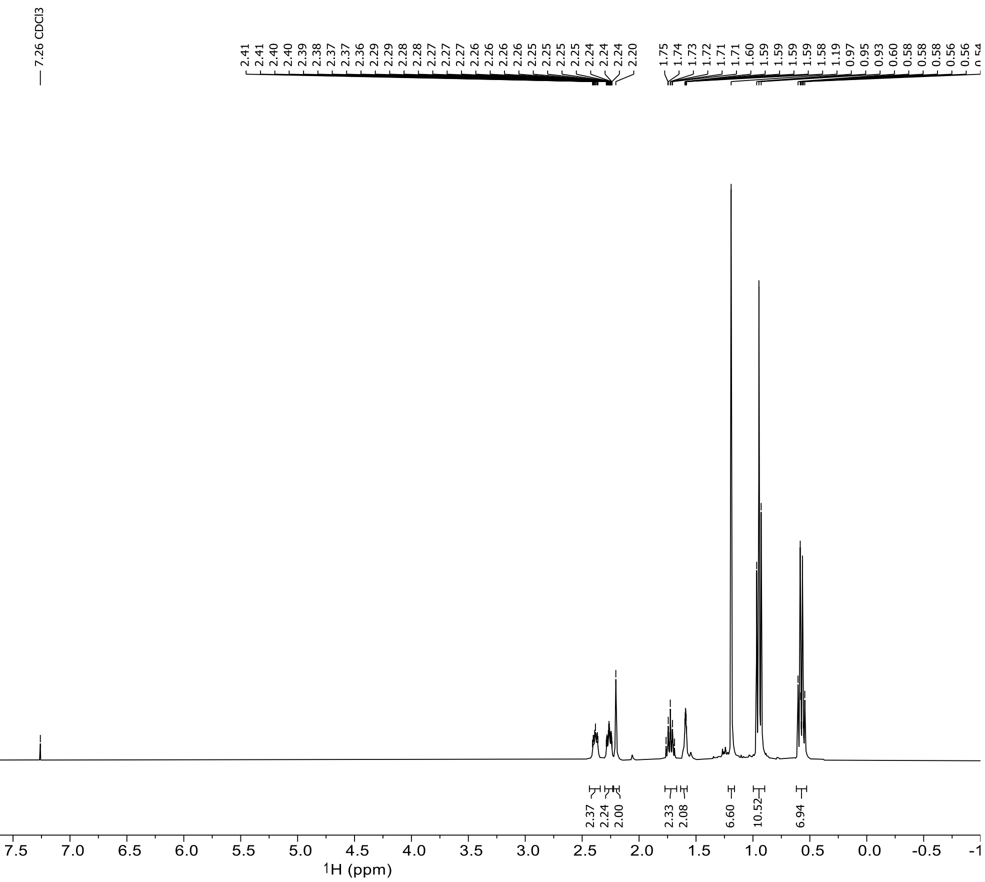
7.26 CDCl3
2.41 2.41 2.40 2.40 2.39 2.38 2.37 2.37 2.36 2.29 2.29 2.28 2.28 2.27 2.27 2.27 2.26 2.26 2.26 2.26 2.25 2.25 2.25 2.25 2.24 2.24 2.24 2.20
1.75 1.74 1.73 1.72 1.71 1.71 1.60 1.59 1.59 1.59 1.59 1.58 1.19 0.97 0.95 0.93 0.60 0.58 0.58 0.58 0.56 0.56
2.37 2.24 2.00 2.33 2.08 6.60 10.52 6.94
1H (ppm)

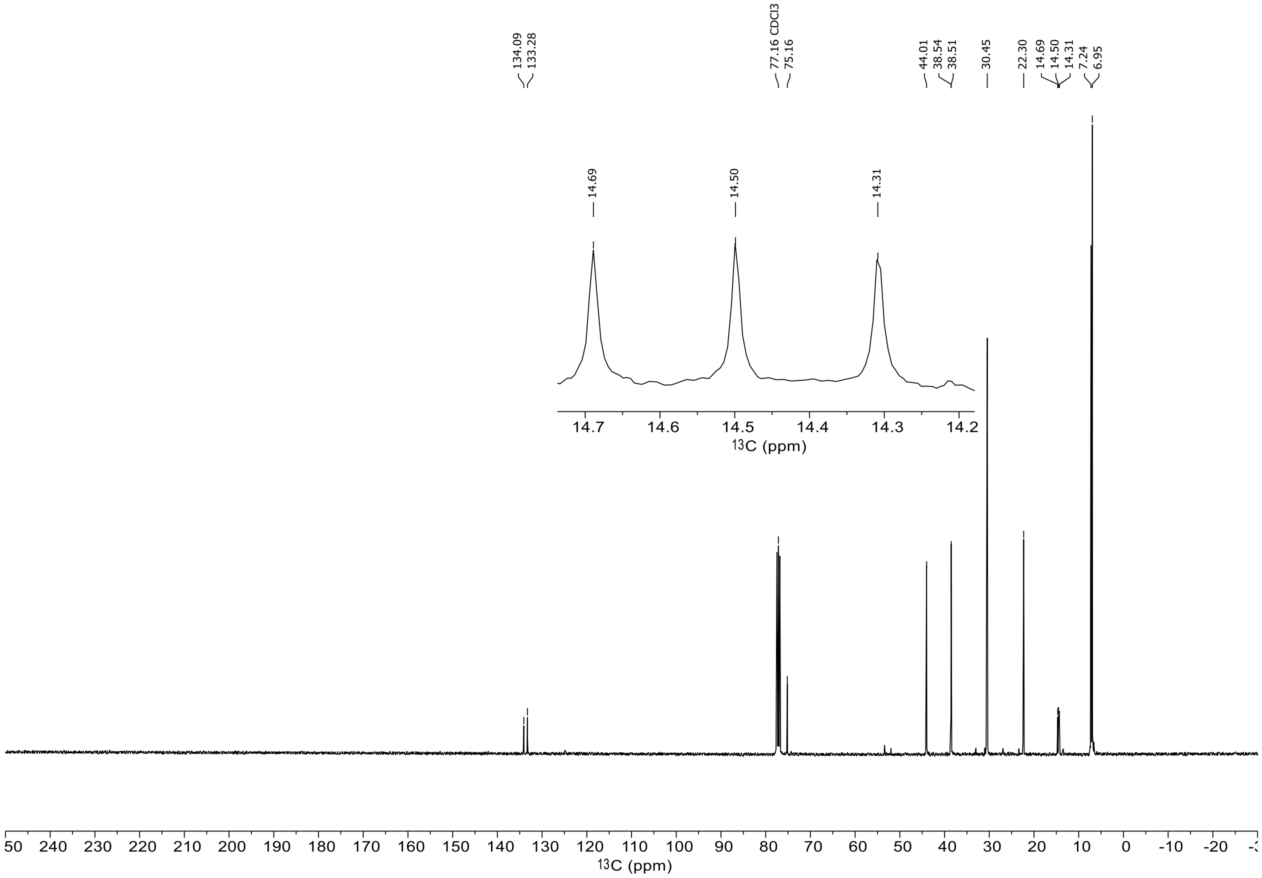
134.09 133.28
77.16 CDCl3 75.16
44.01 38.54 38.51 30.45 22.30 14.69 14.50 14.31 7.24 6.95
14.69 14.50 14.31
14.7 14.6 14.5 14.4 14.3 14.2
13C (ppm)
13C (ppm)

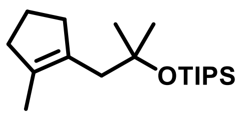
OTIPS

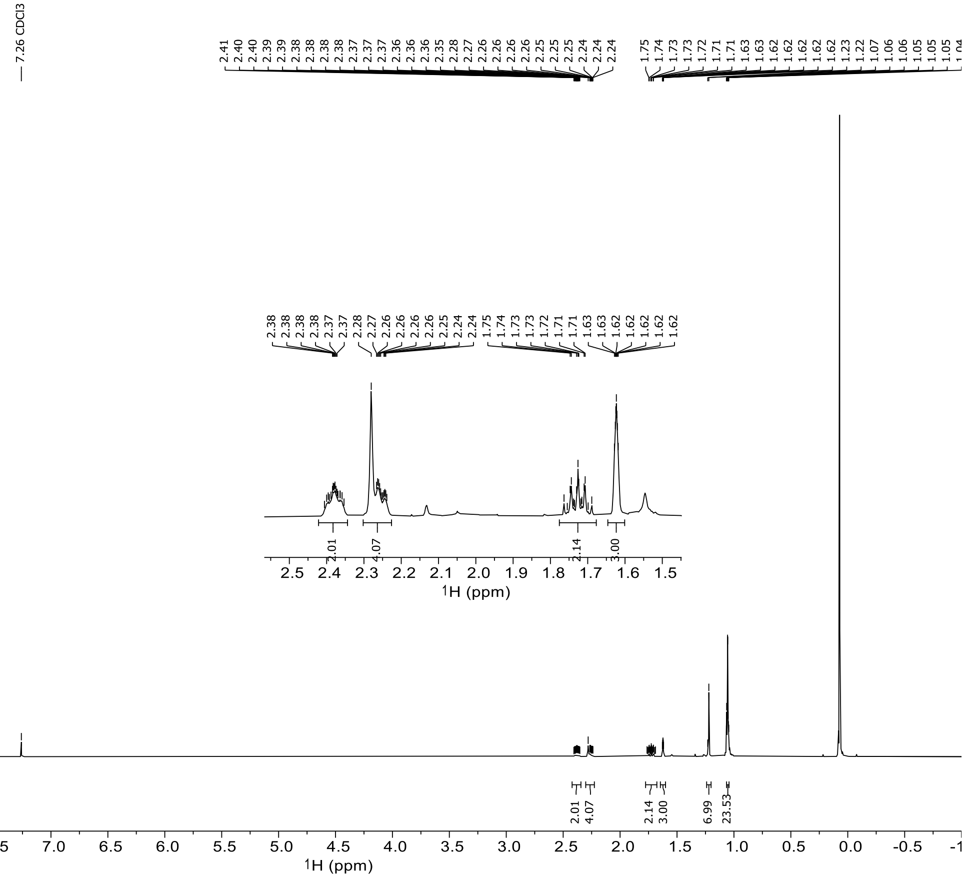
7.26 CDCl3
2.01
4.07
2.14
3.00
6.99
23.53
1H (ppm)

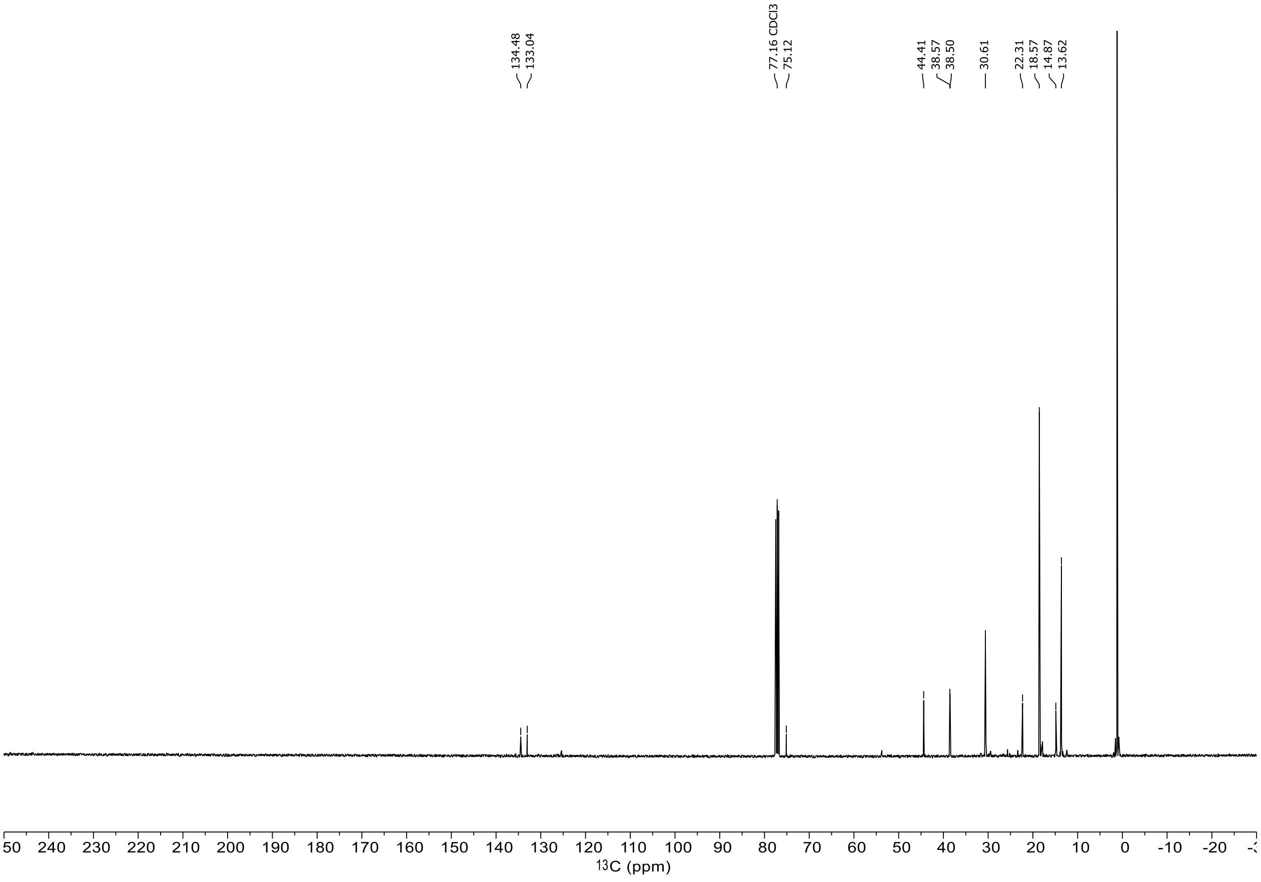
134.48
133.04
77.16 CDCl3
75.12
44.41
38.57
38.50
30.61
22.31
18.57
14.87
13.62
13C (ppm)

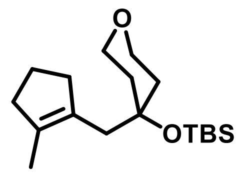
O
OTBS

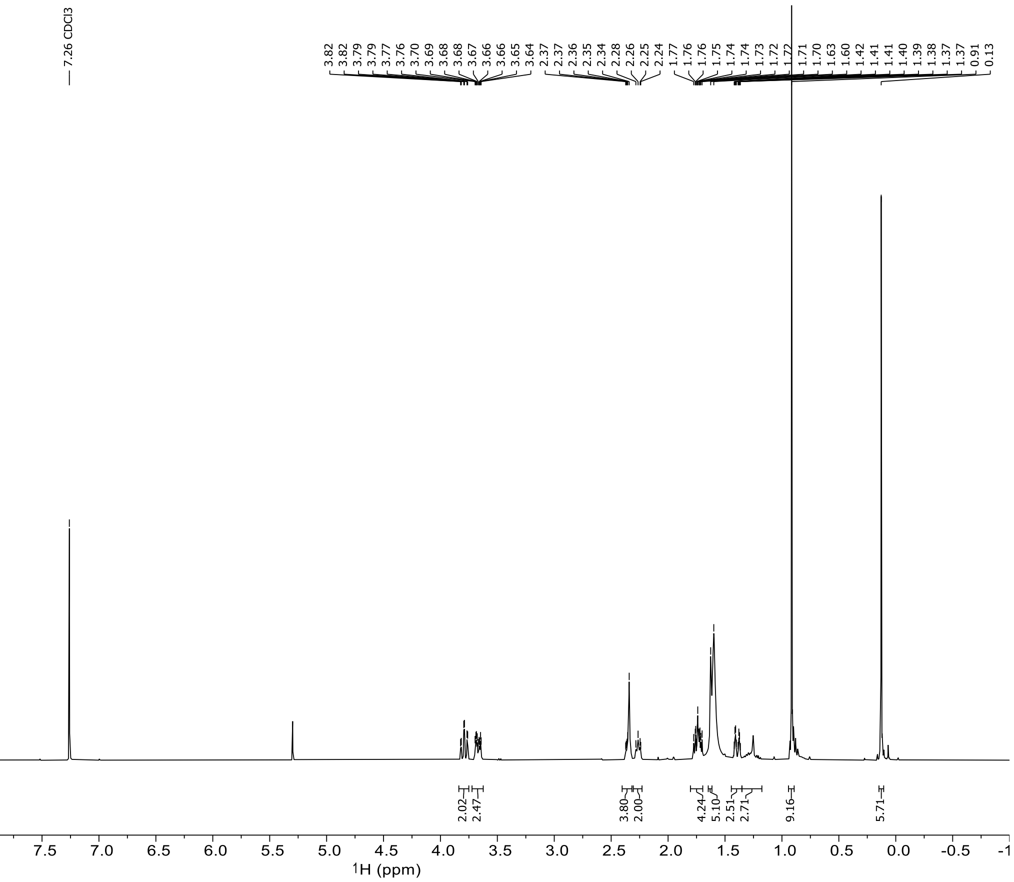
7.26 CDCl3
3.82 3.82 3.79 3.79 3.77 3.76 3.70 3.69 3.68 3.67 3.66 3.66 3.65 3.64 2.37 2.37 2.36 2.35 2.34 2.28 2.26 2.25 2.24 1.77 1.76 1.76 1.75 1.74 1.74 1.73 1.72 1.72 1.71 1.70 1.63 1.60 1.42 1.41 1.41 1.40 1.39 1.38 1.37 1.37 0.91 0.13
2.02 2.47 3.80 2.00 4.24 5.10 2.51 2.71 9.16 5.71
1H (ppm)

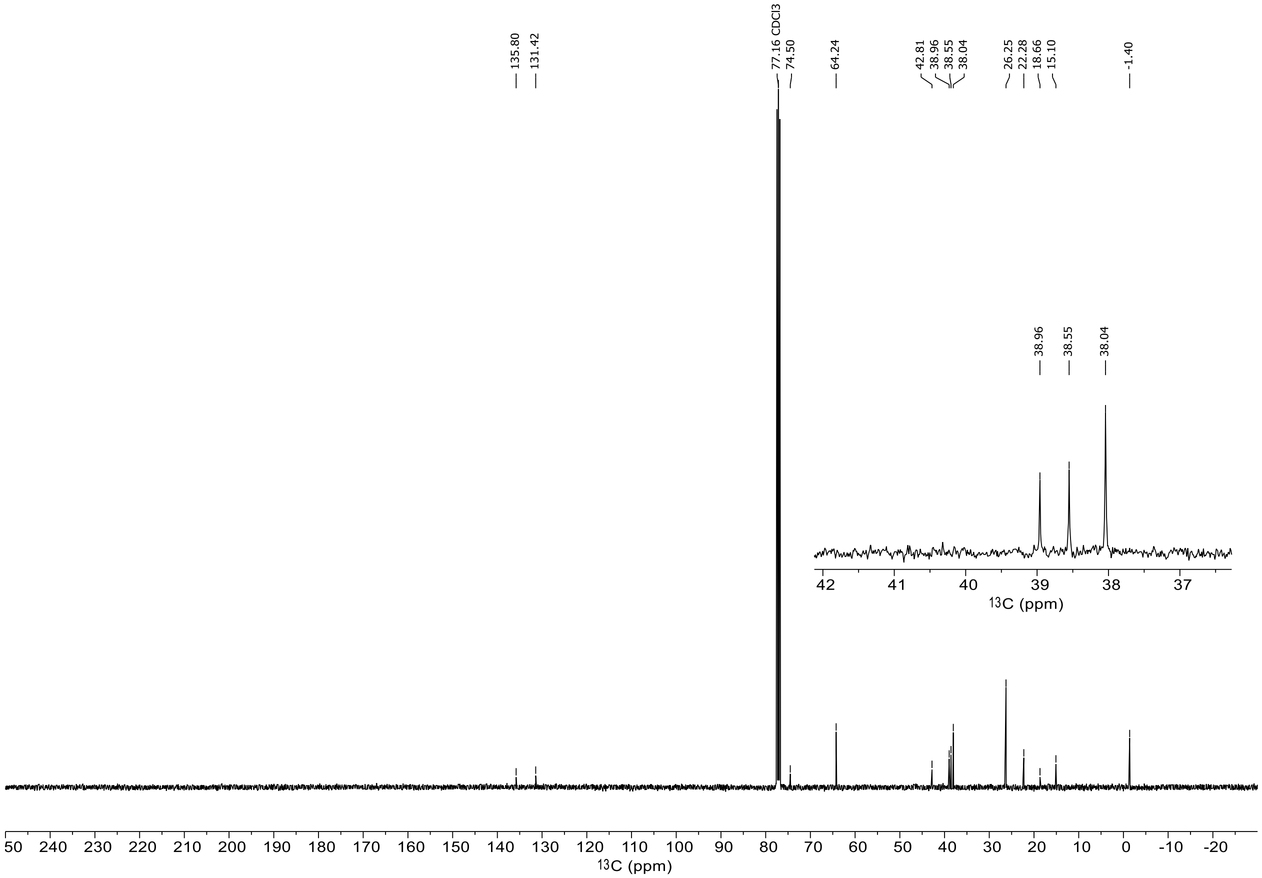
135.80 131.42 77.16 CDCl3 74.50 64.24 42.81 38.96 38.55 38.04 26.25 22.28 18.66 15.10 -1.40
38.96 38.55 38.04
13C (ppm)
13C (ppm)

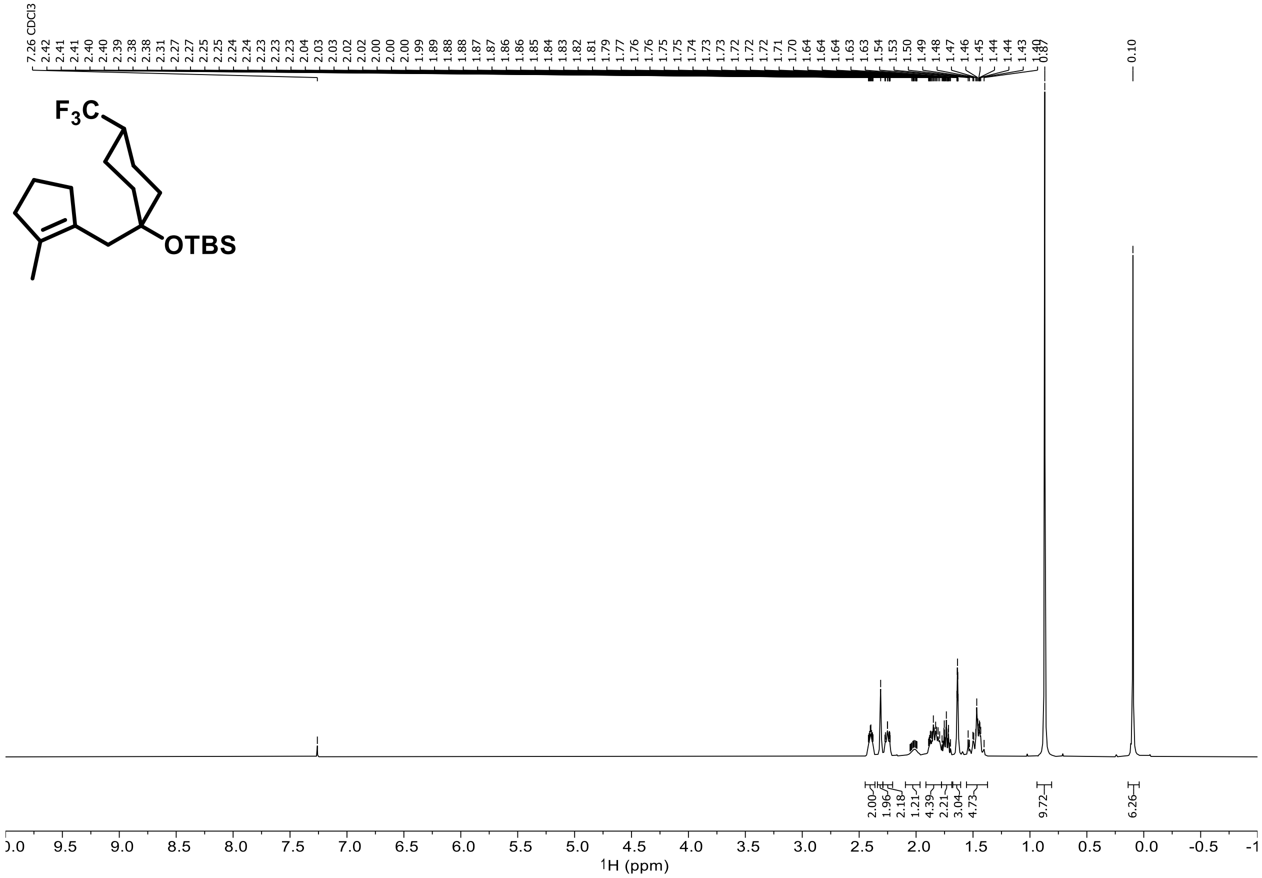
7.26 CDCl3
0.10
F3C
OTBS
1H (ppm)

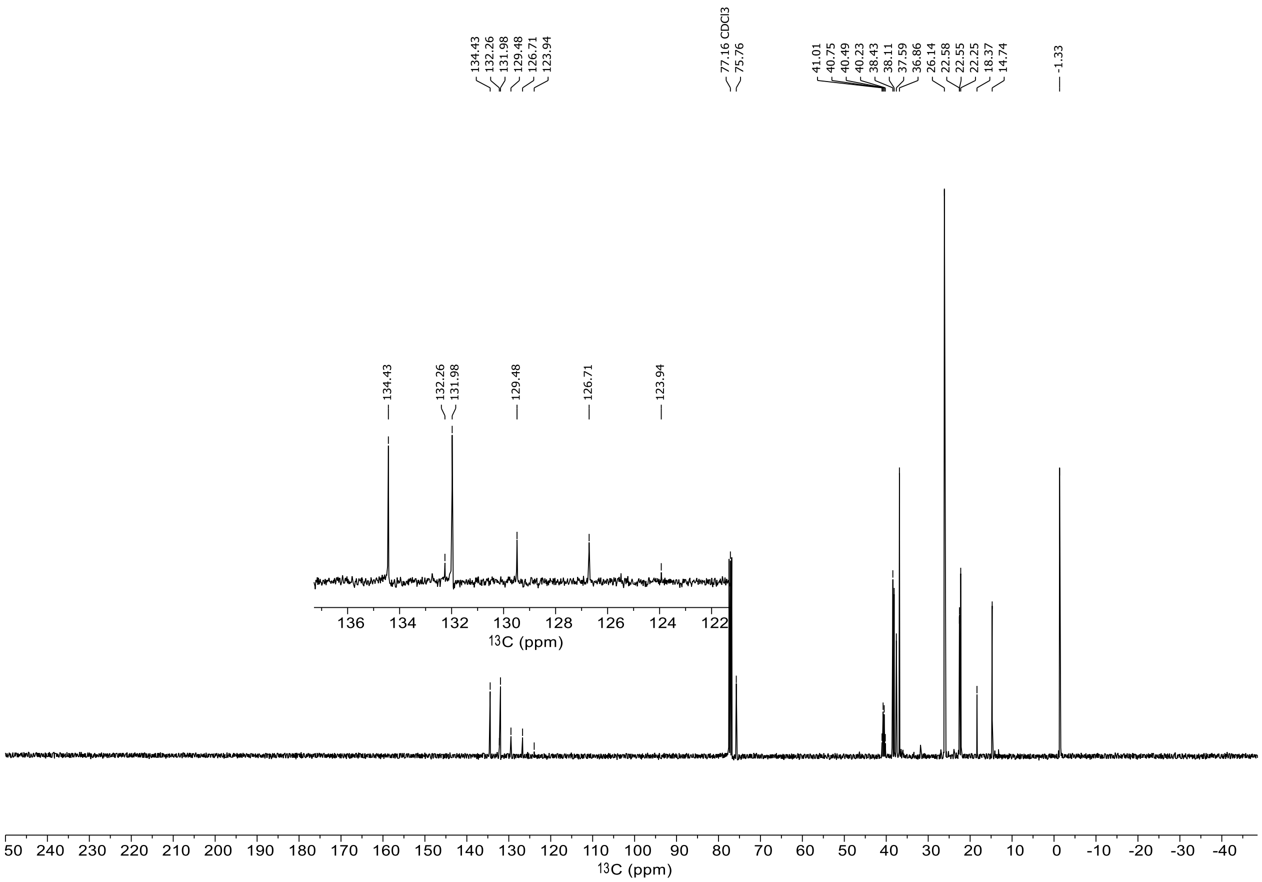
134.43
132.26
131.98
129.48
126.71
123.94
77.16 CDCl3
75.76
41.01
40.75
40.49
40.23
38.43
38.11
37.59
36.86
26.14
22.58
22.55
22.25
18.37
14.74
-1.33
13C (ppm)

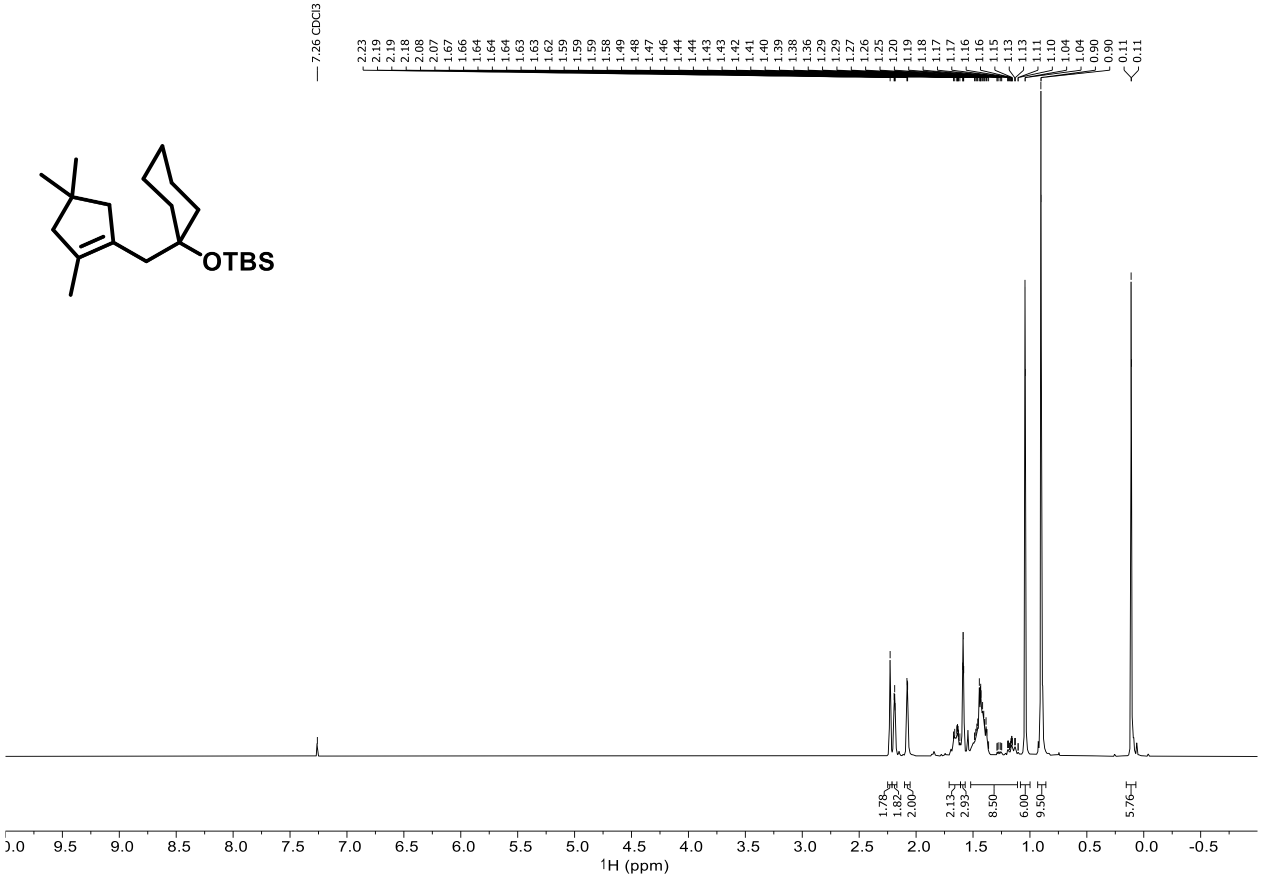

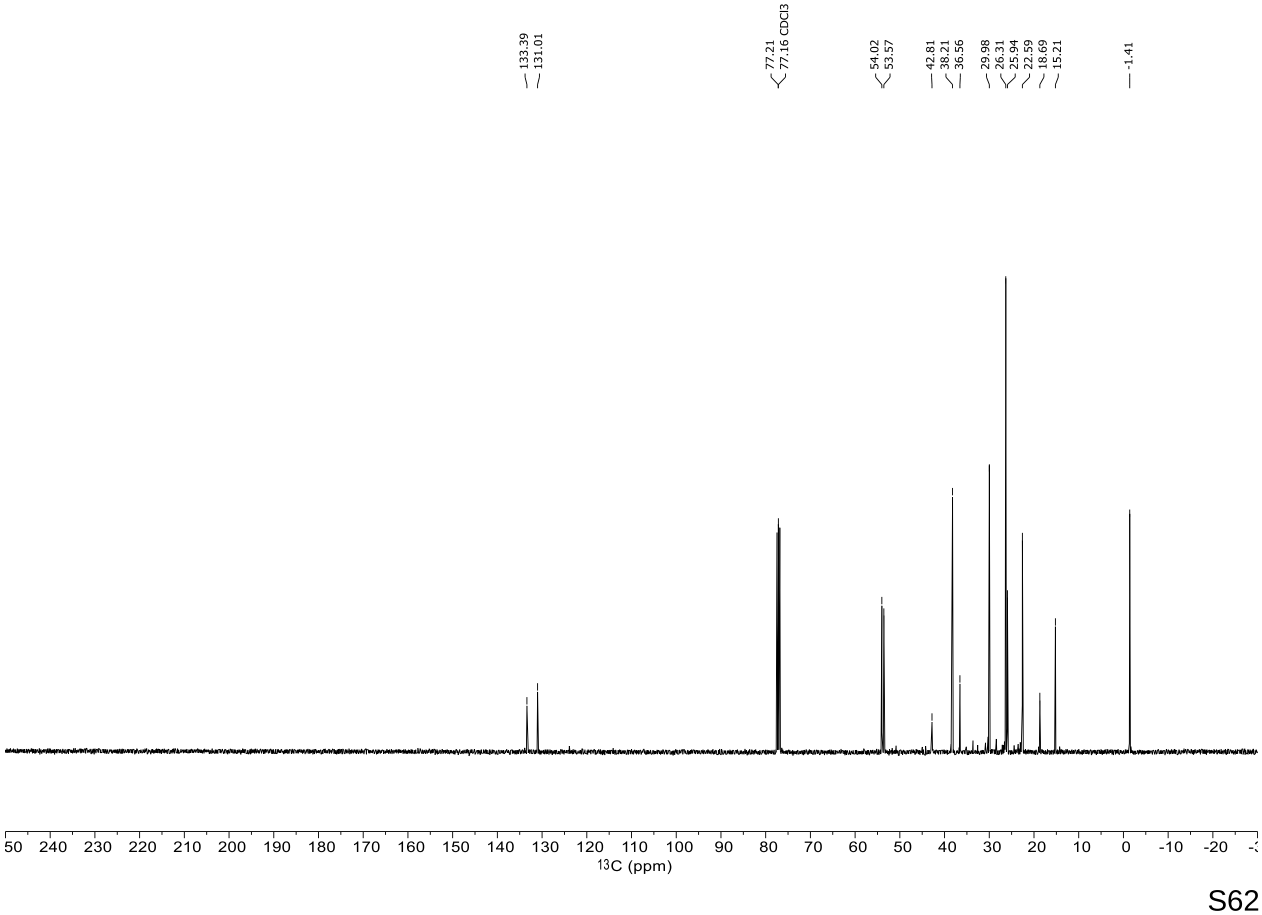

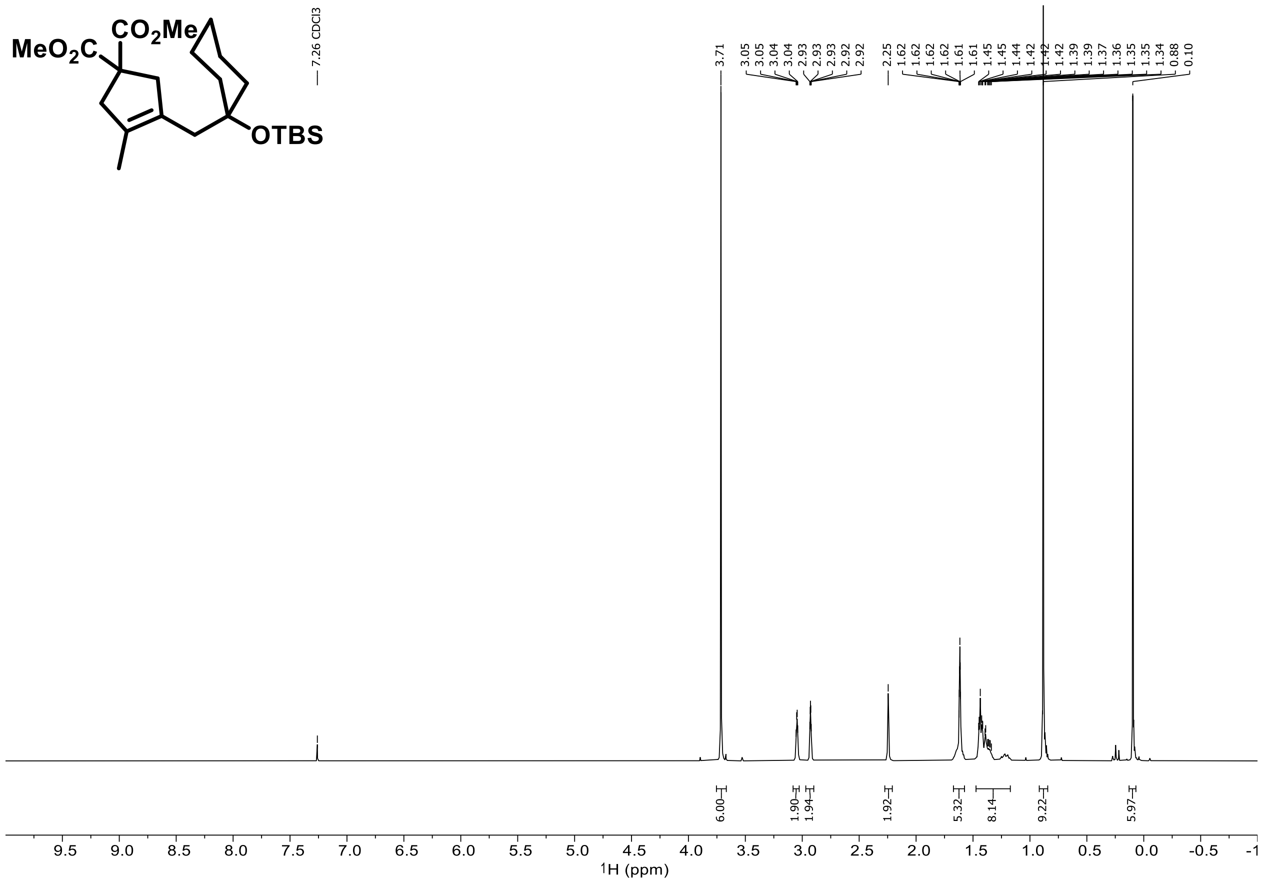
MeO2C
CO2Me
OTBS
7.26 CDCl3
3.71
3.05
3.05
3.04
3.04
2.93
2.93
2.93
2.92
2.92
2.25
1.62
1.62
1.62
1.62
1.61
1.61
1.45
1.45
1.44
1.42
1.42
1.42
1.39
1.39
1.37
1.36
1.35
1.35
1.34
0.88
0.10
6.00
1.90
1.94
1.92
5.32
8.14
9.22
5.97
9.5 9.0 8.5 8.0 7.5 7.0 6.5 6.0 5.5 5.0 4.5 4.0 3.5 3.0 2.5 2.0 1.5 1.0 0.5 0.0 -0.5
1H (ppm)

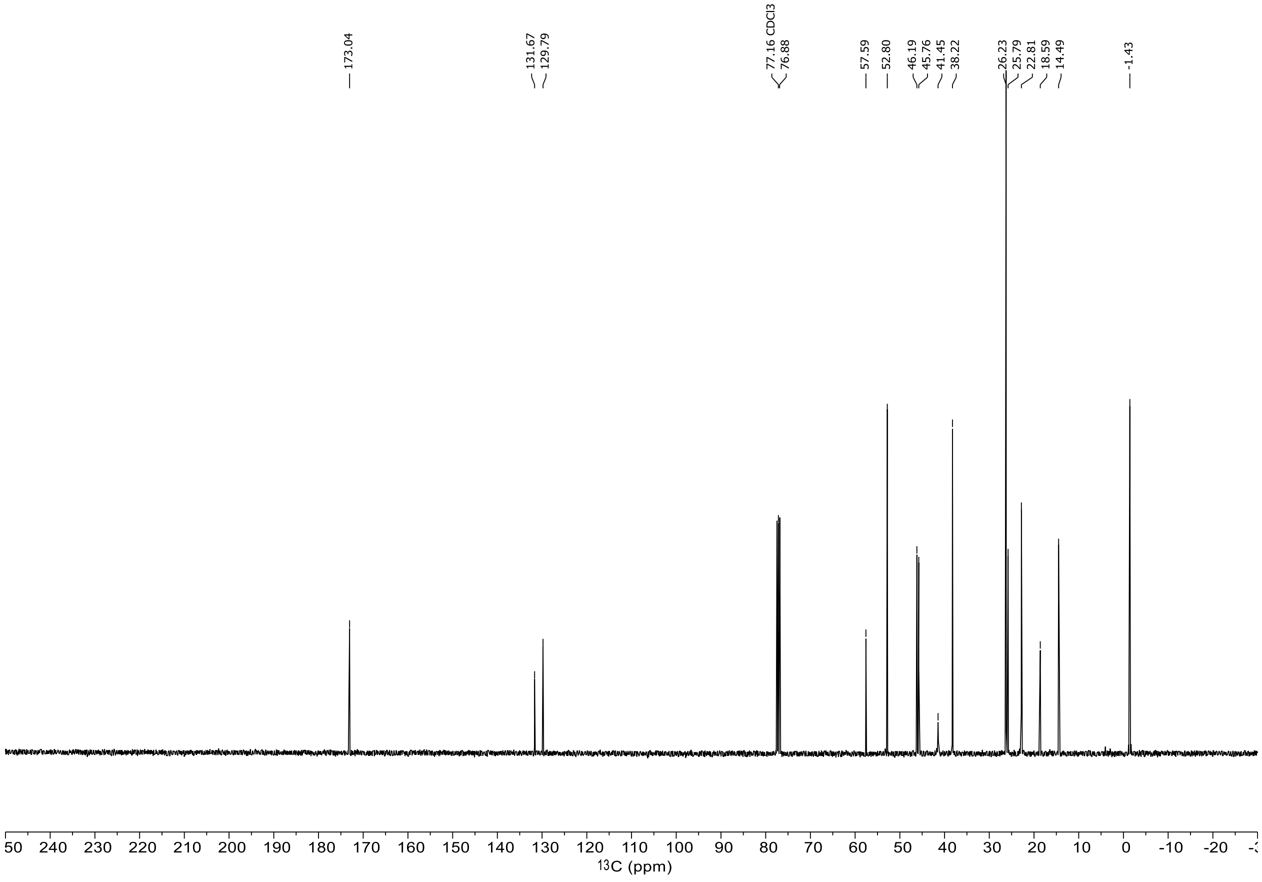
173.04
131.67
129.79
77.16 CDCl3
76.88
57.59
52.80
46.19
45.76
41.45
38.22
26.23
25.79
22.81
18.59
14.49
-1.43
240 230 220 210 200 190 180 170 160 150 140 130 120 110 100 90 80 70 60 50 40 30 20 10 0 -10 -20
13C (ppm)

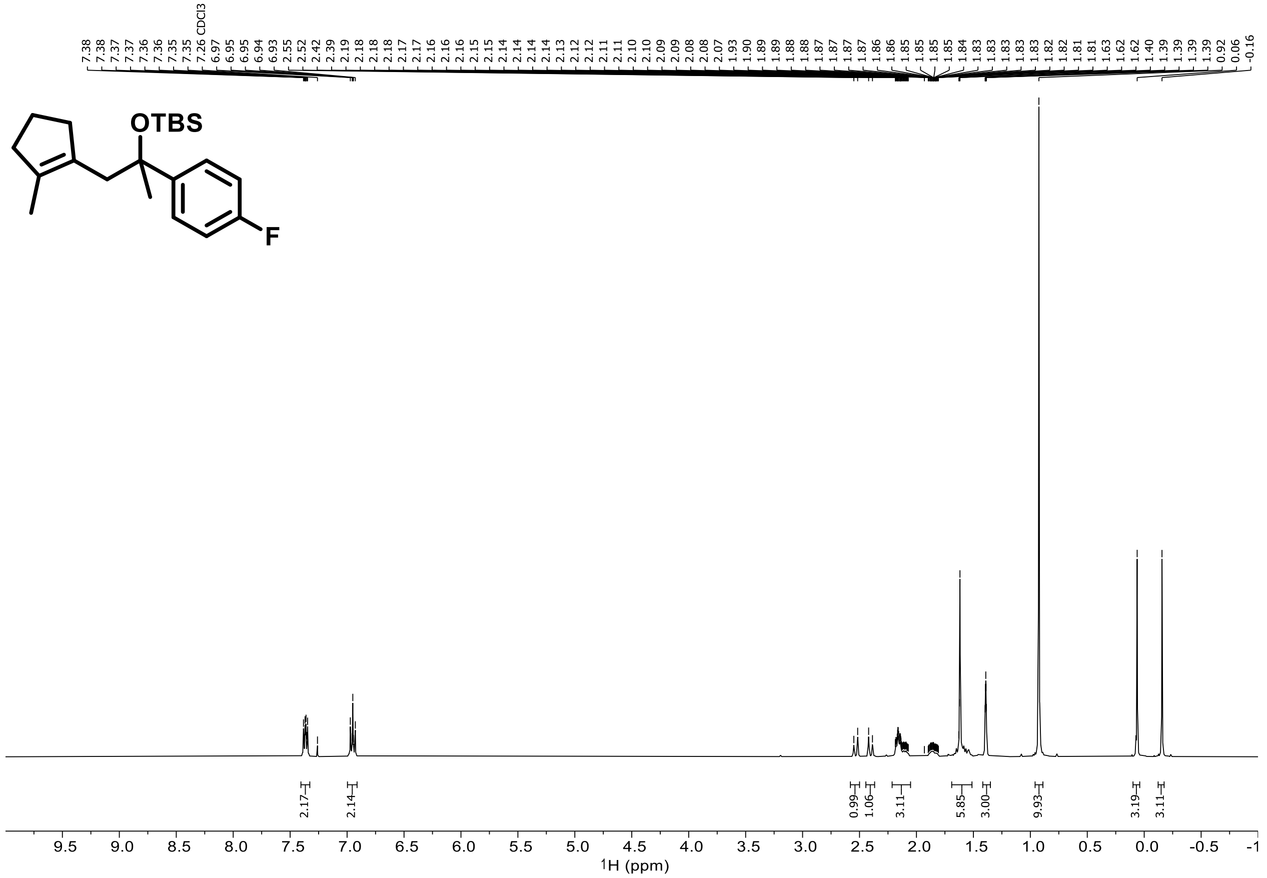
7.38 7.38 7.37 7.37 7.36 7.36 7.35 7.35 7.26 CDCl3 6.97 6.95 6.95 6.94 6.93 2.55 2.52 2.42 2.39 2.19 2.18 2.18 2.18 2.17 2.17 2.16 2.16 2.16 2.15 2.15 2.14 2.14 2.14 2.14 2.13 2.12 2.12 2.11 2.11 2.10 2.10 2.09 2.09 2.08 2.08 2.07 1.93 1.90 1.89 1.89 1.88 1.88 1.87 1.87 1.87 1.87 1.86 1.86 1.85 1.85 1.85 1.85 1.84 1.83 1.83 1.83 1.83 1.83 1.82 1.82 1.81 1.81 1.63 1.62 1.62 1.40 1.39 1.39 1.39 1.39 0.92 0.06 -0.16
OTBS
F
2.17
2.14
0.99
1.06
3.11
5.85
3.00
9.93
3.19
3.11
9.5 9.0 8.5 8.0 7.5 7.0 6.5 6.0 5.5 5.0 4.5 4.0 3.5 3.0 2.5 2.0 1.5 1.0 0.5 0.0 -0.5
1H (ppm)

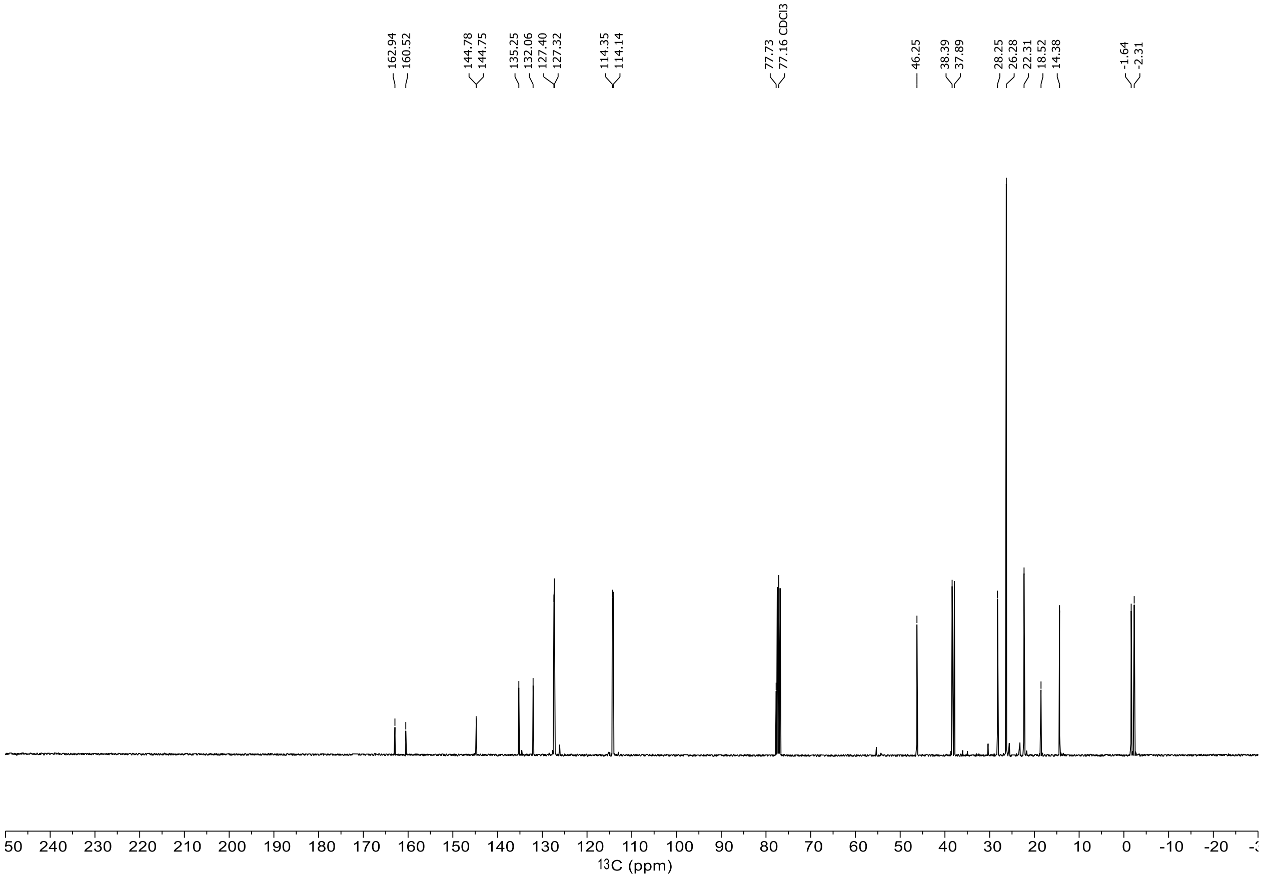
162.94 160.52
144.78 144.75
135.25 132.06 127.40 127.32
114.35 114.14
77.73 77.16 CDCl3
46.25
38.39 37.89
28.25 26.28 22.31 18.52 14.38
-1.64 -2.31
240 230 220 210 200 190 180 170 160 150 140 130 120 110 100 90 80 70 60 50 40 30 20 10 0 -10 -20
13C (ppm)

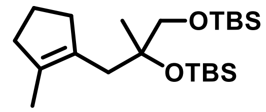
OTBS
OTBS

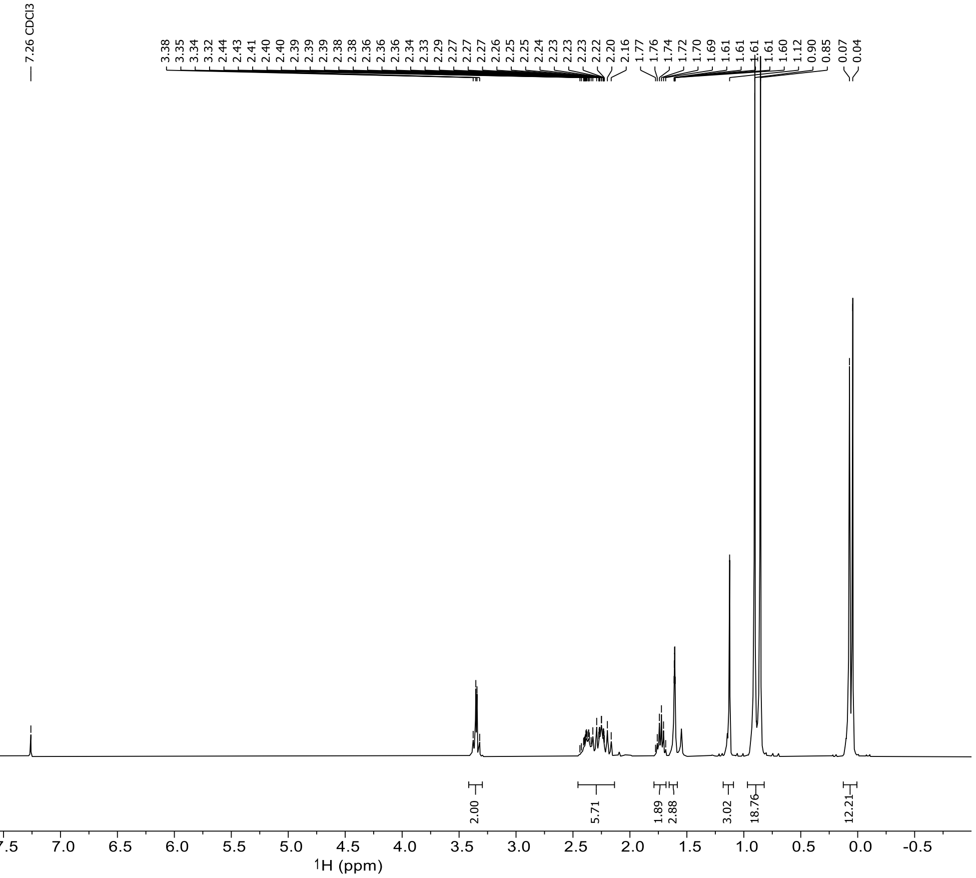
7.26 CDCl3
3.38
3.35
3.34
3.32
2.44
2.43
2.41
2.40
2.40
2.39
2.39
2.39
2.38
2.38
2.36
2.36
2.34
2.33
2.29
2.27
2.27
2.27
2.26
2.25
2.25
2.24
2.23
2.23
2.23
2.22
2.20
2.16
1.77
1.76
1.74
1.72
1.70
1.69
1.61
1.61
1.61
1.61
1.60
1.12
0.90
0.85
0.07
0.04
2.00
5.71
1.89
2.88
3.02
18.76
12.21
).0 9.5 9.0 8.5 8.0 7.5 7.0 6.5 6.0 5.5 5.0 4.5 4.0 3.5 3.0 2.5 2.0 1.5 1.0 0.5 0.0 -0.5
1H (ppm)

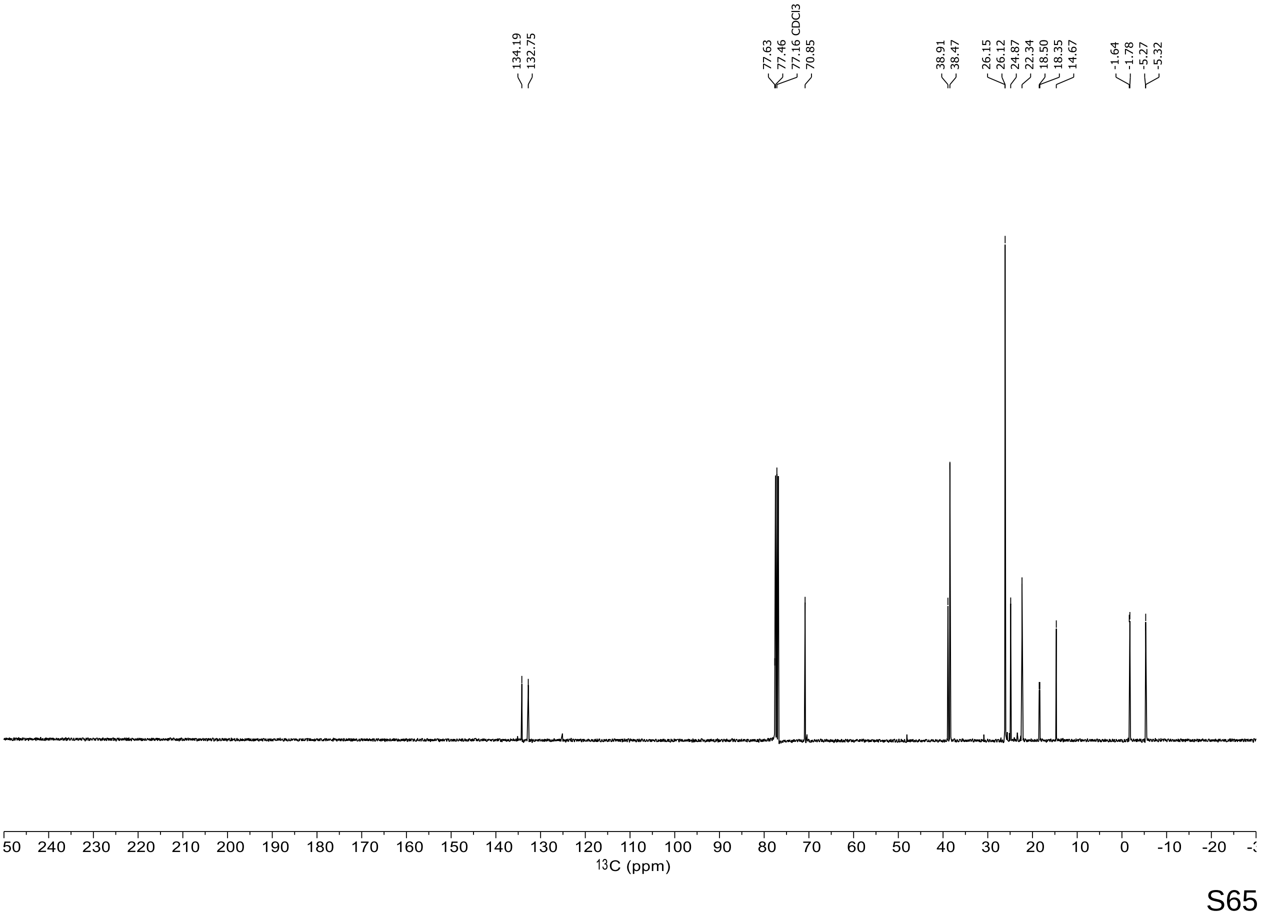
134.19
132.75
77.63
77.46
77.16 CDCl3
70.85
38.91
38.47
26.15
26.12
24.87
22.34
18.50
18.35
14.67
-1.64
-1.78
-5.27
-5.32
50 240 230 220 210 200 190 180 170 160 150 140 130 120 110 100 90 80 70 60 50 40 30 20 10 0 -10 -20
13C (ppm)

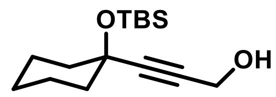
OTBS
OH

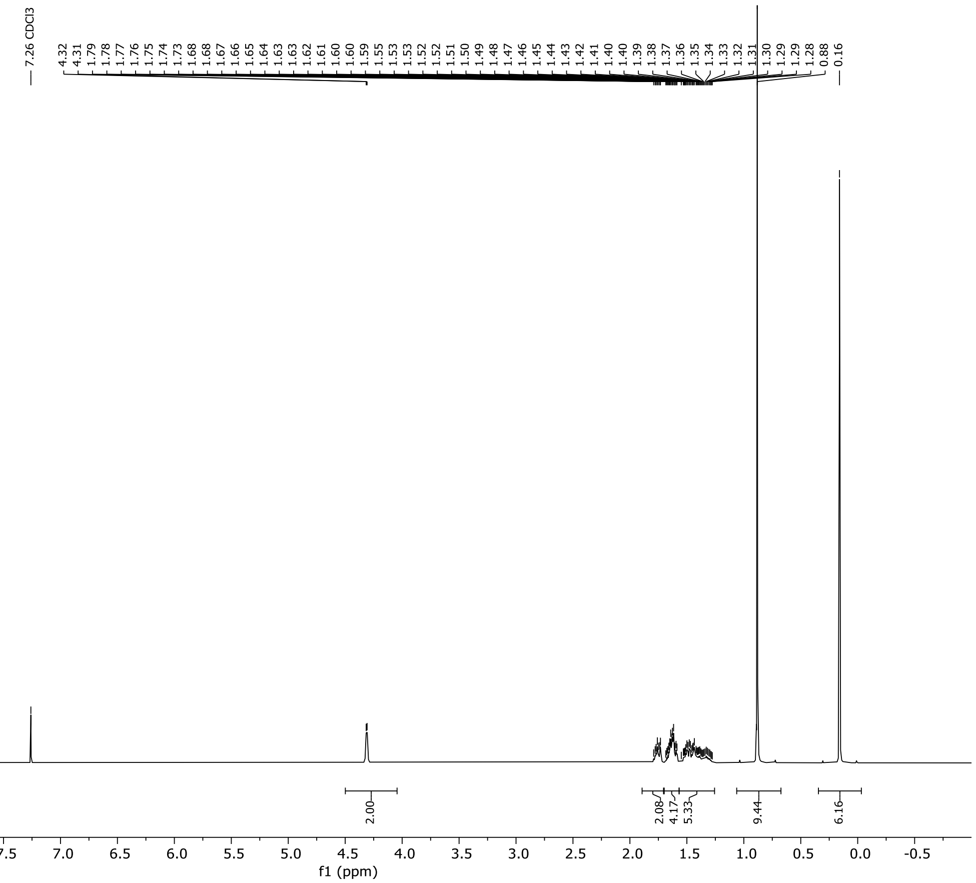
7.26 CDCl3
4.32
4.31
1.79
1.78
1.77
1.76
1.75
1.74
1.73
1.68
1.68
1.67
1.66
1.65
1.64
1.63
1.63
1.62
1.61
1.60
1.60
1.59
1.55
1.53
1.53
1.52
1.52
1.51
1.50
1.49
1.48
1.47
1.46
1.45
1.44
1.43
1.42
1.41
1.40
1.40
1.39
1.38
1.37
1.36
1.35
1.34
1.33
1.32
1.31
1.30
1.29
1.29
1.28
0.88
0.16
2.00
2.08
4.17
5.33
9.44
6.16
f1 (ppm)

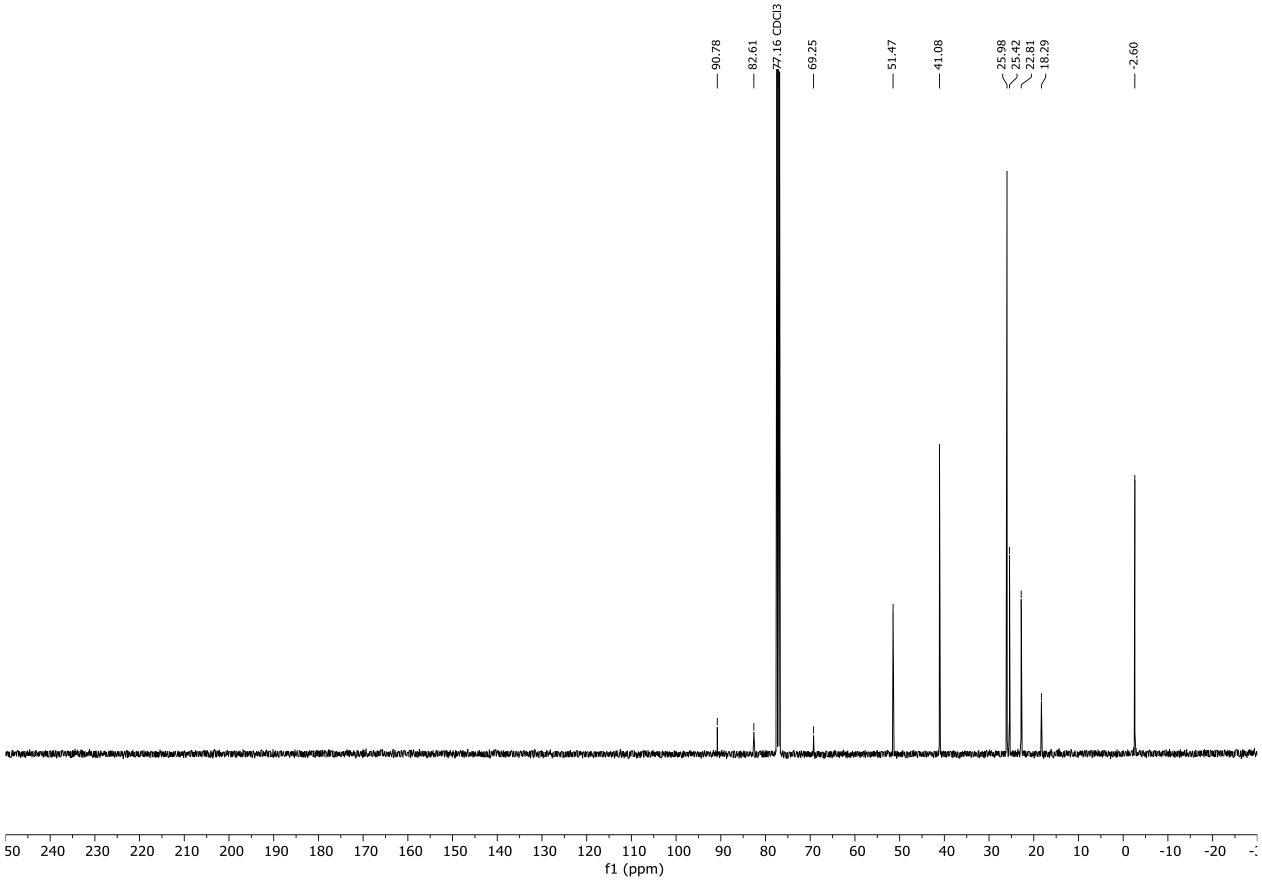
90.78
82.61
77.16 CDCl3
69.25
51.47
41.08
25.98
25.42
22.81
18.29
-2.60
f1 (ppm)

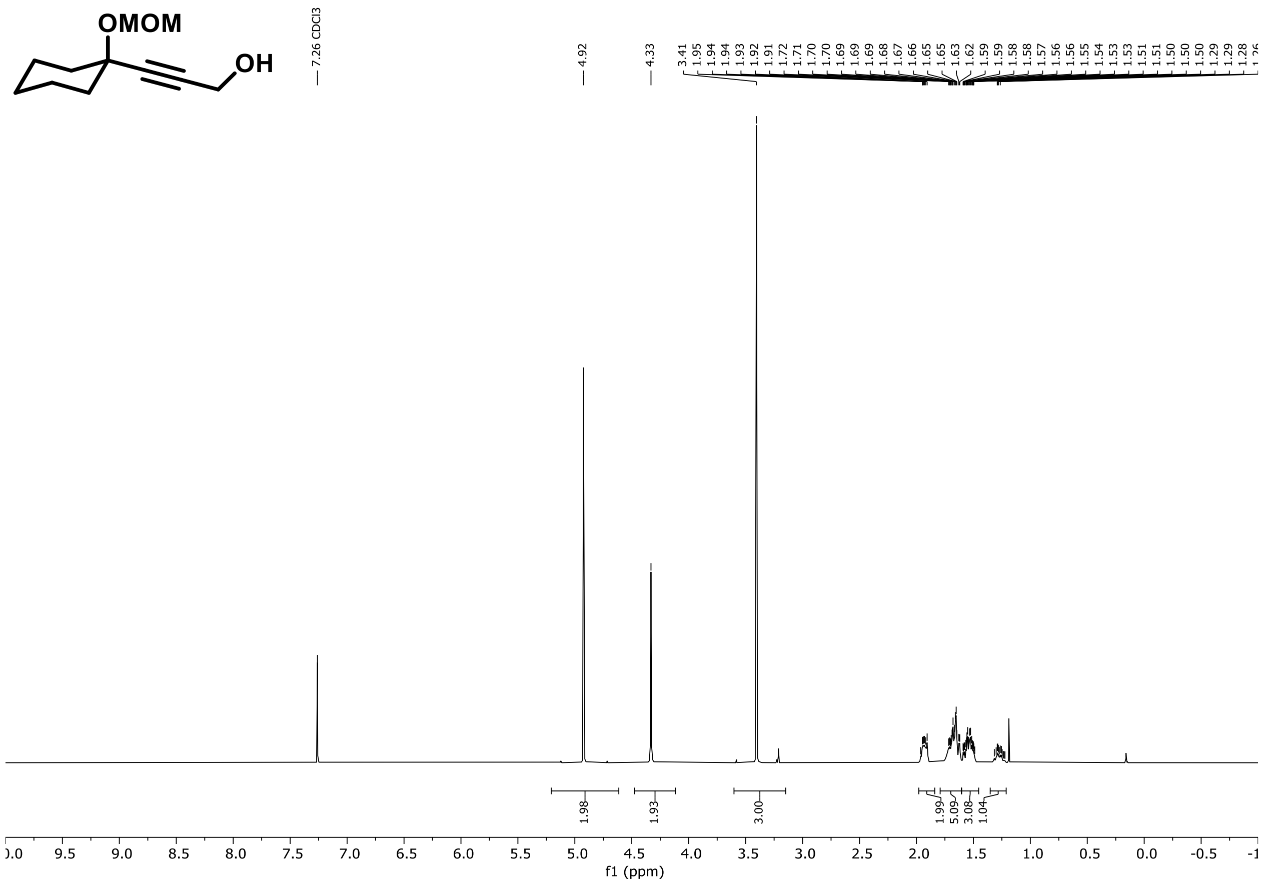
OMOM
OH
7.26 CDCl3
4.92
4.33
3.41
1.95
1.94
1.94
1.93
1.92
1.91
1.72
1.71
1.70
1.70
1.69
1.69
1.69
1.68
1.67
1.66
1.65
1.65
1.63
1.62
1.59
1.59
1.58
1.58
1.57
1.56
1.56
1.55
1.54
1.53
1.53
1.51
1.51
1.50
1.50
1.50
1.29
1.28
1.98
1.93
3.00
1.99
5.09
3.08
1.04
9.5 9.0 8.5 8.0 7.5 7.0 6.5 6.0 5.5 5.0 4.5 4.0 3.5 3.0 2.5 2.0 1.5 1.0 0.5 0.0 -0.5
f1 (ppm)

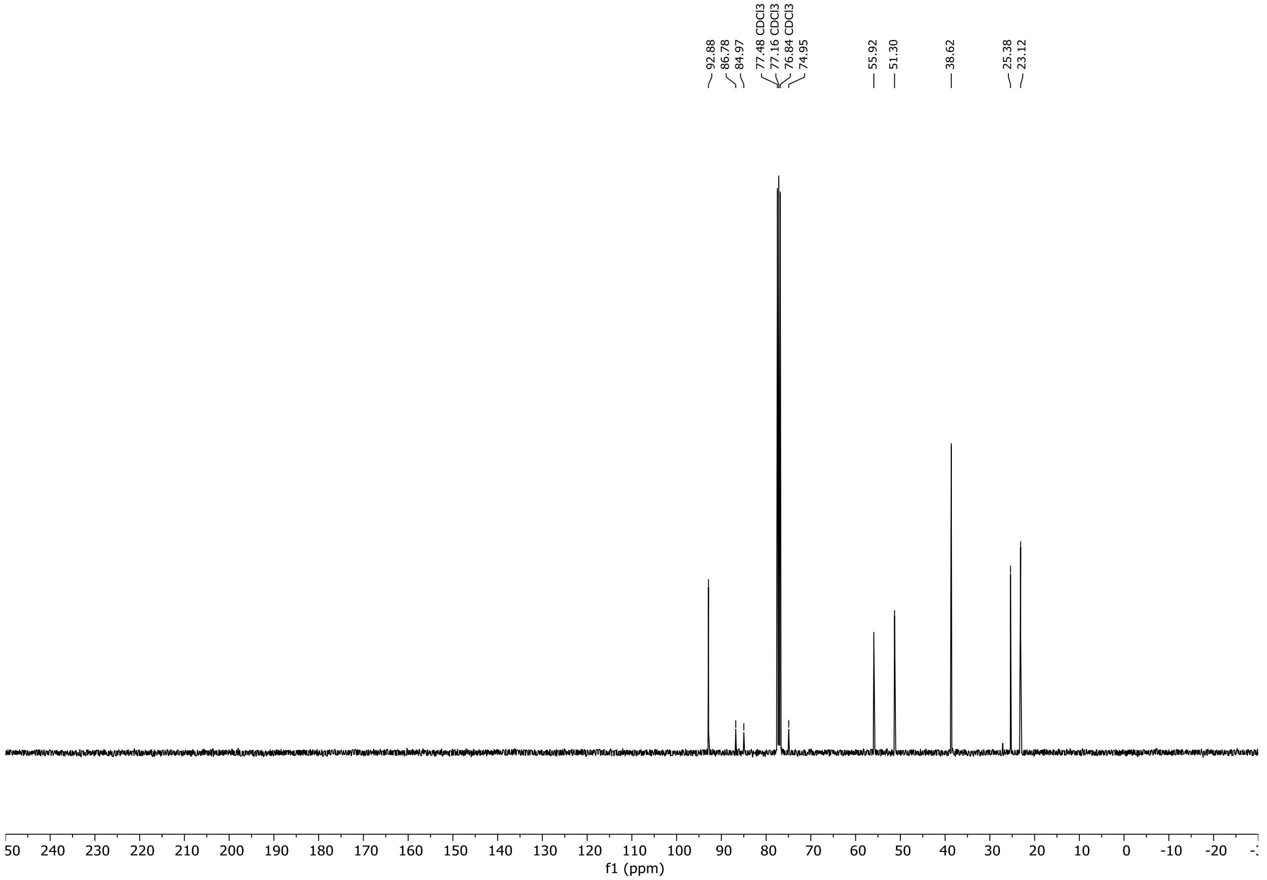
92.88
86.78
84.97
77.48 CDCl3
77.16 CDCl3
76.84 CDCl3
74.95
55.92
51.30
38.62
25.38
23.12
240 230 220 210 200 190 180 170 160 150 140 130 120 110 100 90 80 70 60 50 40 30 20 10 0 -10 -20
f1 (ppm)

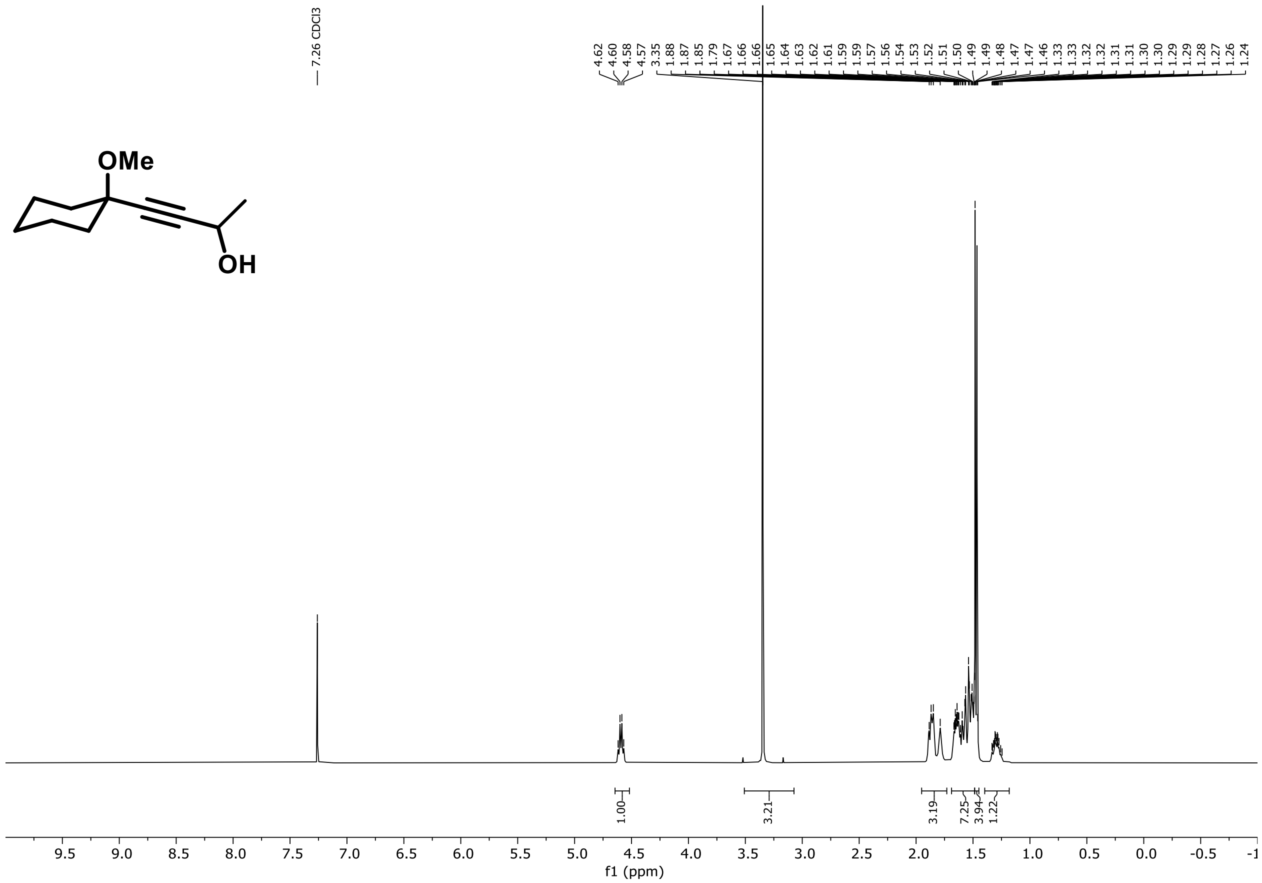
OMe
OH
7.26 CDCl3
4.62
4.60
4.58
4.57
3.35
1.88
1.87
1.85
1.79
1.67
1.66
1.66
1.65
1.64
1.63
1.62
1.61
1.59
1.59
1.57
1.56
1.54
1.53
1.52
1.51
1.50
1.49
1.49
1.48
1.47
1.46
1.33
1.33
1.32
1.32
1.31
1.31
1.30
1.30
1.29
1.29
1.28
1.27
1.26
1.24
1.00
3.21
3.19
7.25
3.94
1.22
f1 (ppm)

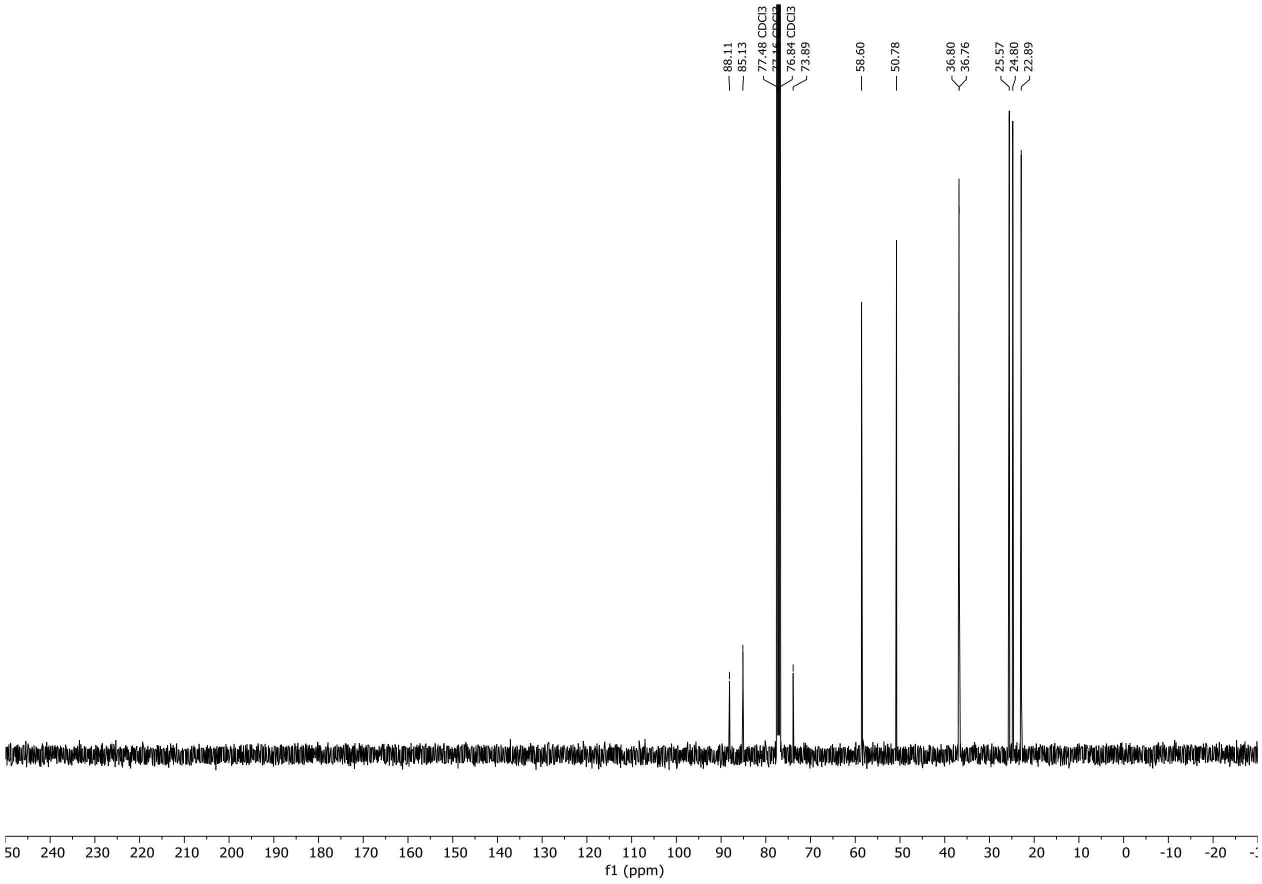
88.11
85.13
77.48 CDCl3
76.84 CDCl3
73.89
58.60
50.78
36.80
36.76
25.57
24.80
22.89
f1 (ppm)

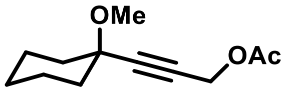
OMe
OAc

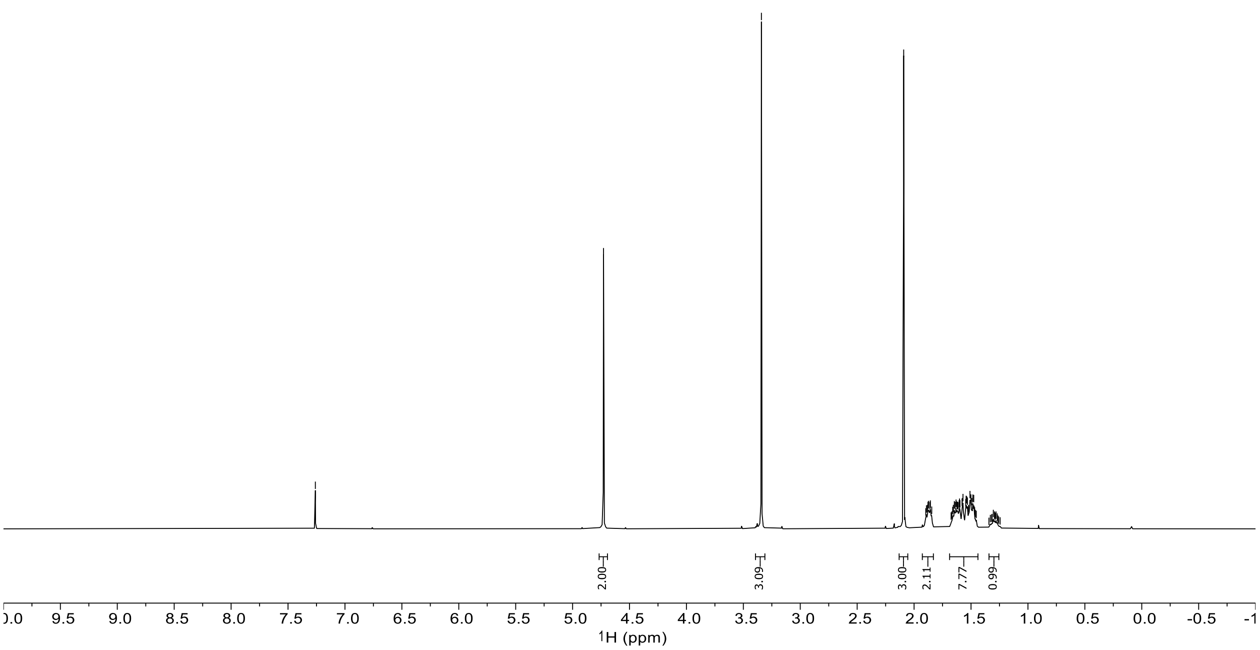
7.26 CDCl3
4.73
3.34 2.09 1.90 1.89 1.88 1.88 1.87 1.86 1.86 1.84 1.67 1.67 1.66 1.65 1.65 1.64 1.63 1.62 1.62 1.61 1.60 1.60 1.60 1.58 1.58 1.57 1.55 1.54 1.54 1.53 1.52 1.51 1.50 1.49 1.48 1.48 1.47 1.46 1.45 1.30 1.29 1.29 1.28
2.00
3.09
3.00
2.11
7.77
0.99
9.5 9.0 8.5 8.0 7.5 7.0 6.5 6.0 5.5 5.0 4.5 4.0 3.5 3.0 2.5 2.0 1.5 1.0 0.5 0.0 -0.5
1H (ppm)

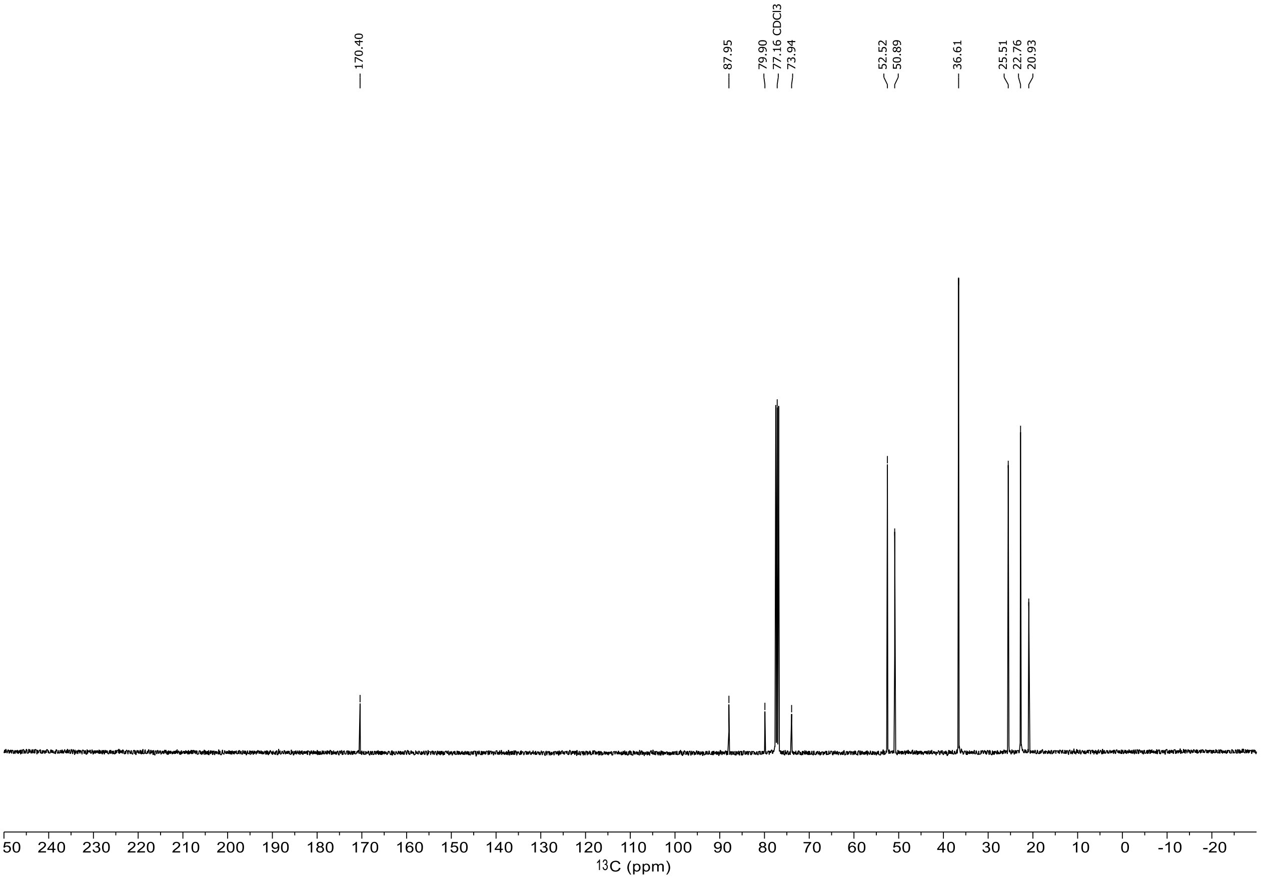
170.40
87.95
79.90
77.16 CDCl3
73.94
52.52
50.89
36.61
25.51
22.76
20.93
240 230 220 210 200 190 180 170 160 150 140 130 120 110 100 90 80 70 60 50 40 30 20 10 0 -10 -20
13C (ppm)

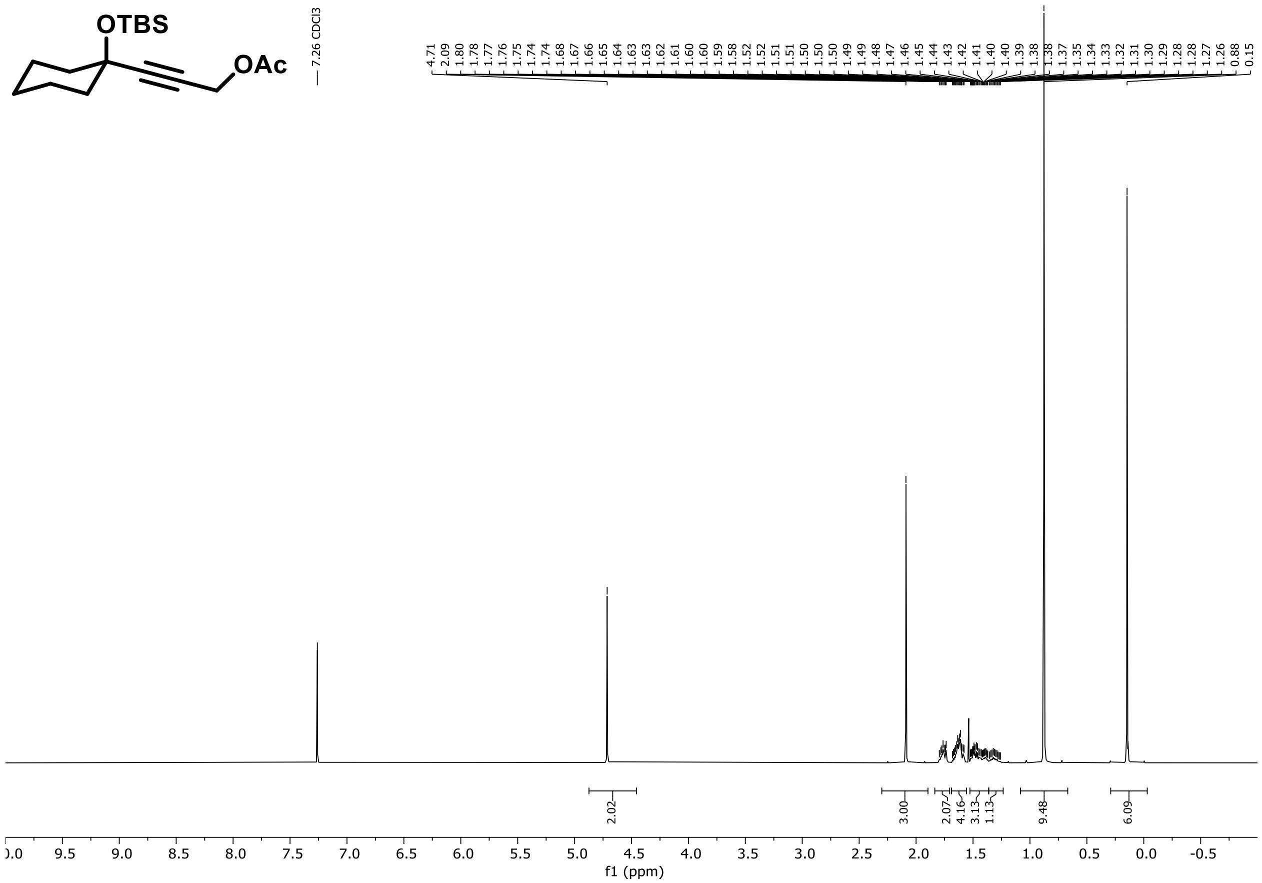
OTBS
OAc
7.26 CDCl3
4.71 2.09 1.80 1.78 1.77 1.76 1.75 1.74 1.74 1.68 1.67 1.66 1.65 1.64 1.63 1.63 1.62 1.61 1.60 1.60 1.59 1.58 1.52 1.52 1.51 1.51 1.50 1.50 1.50 1.49 1.49 1.48 1.47 1.46 1.45 1.44 1.43 1.42 1.41 1.40 1.40 1.39 1.38 1.38 1.37 1.35 1.34 1.33 1.32 1.31 1.30 1.29 1.28 1.28 1.27 1.26 0.88 0.15
2.02
3.00
2.07
4.16
3.13
1.13
9.48
6.09
f1 (ppm)

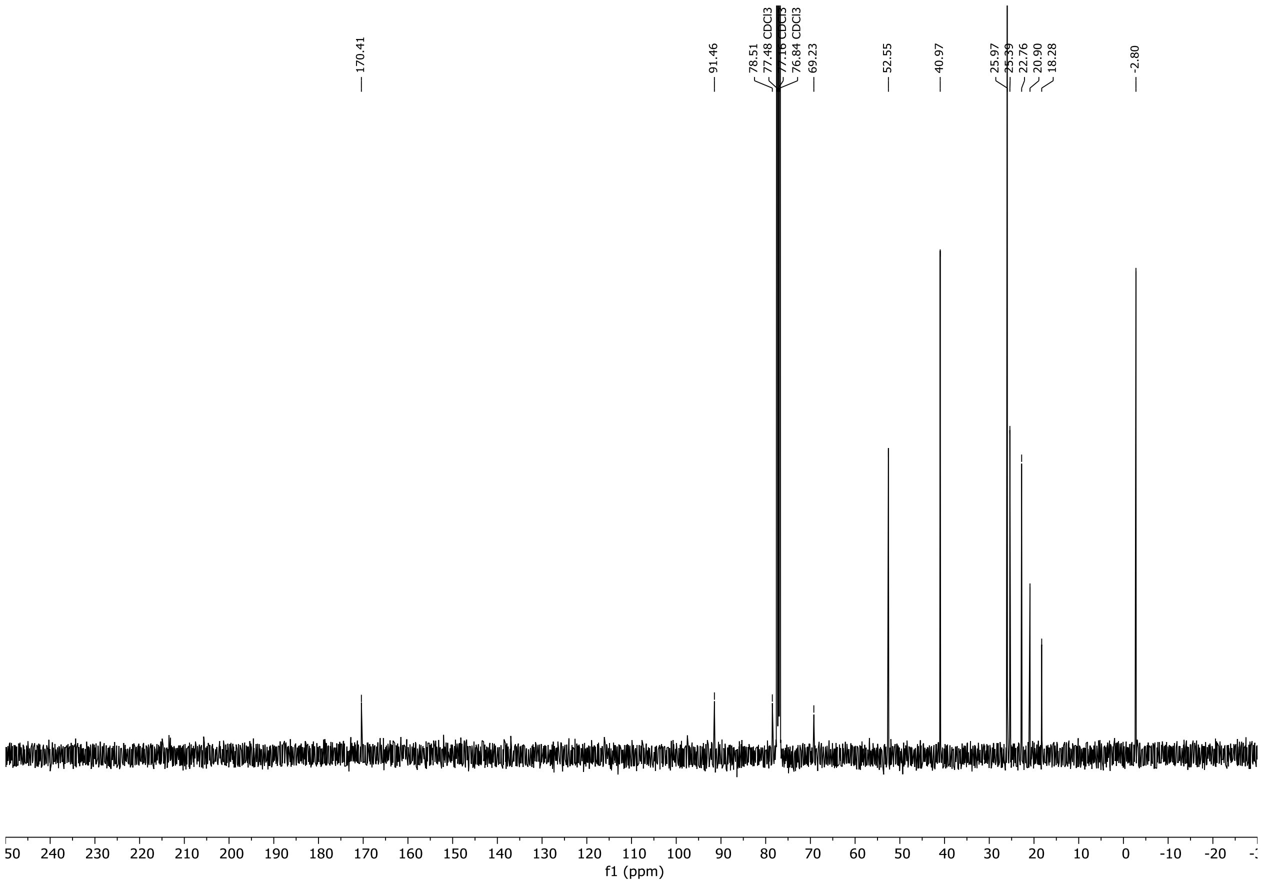
170.41
91.46
78.51
77.48 CDCl3
77.16 CDCl3
76.84 CDCl3
69.23
52.55
40.97
25.97
25.39
22.76
20.90
18.28
-2.80
f1 (ppm)

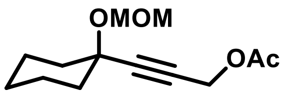
OMOM
OAc

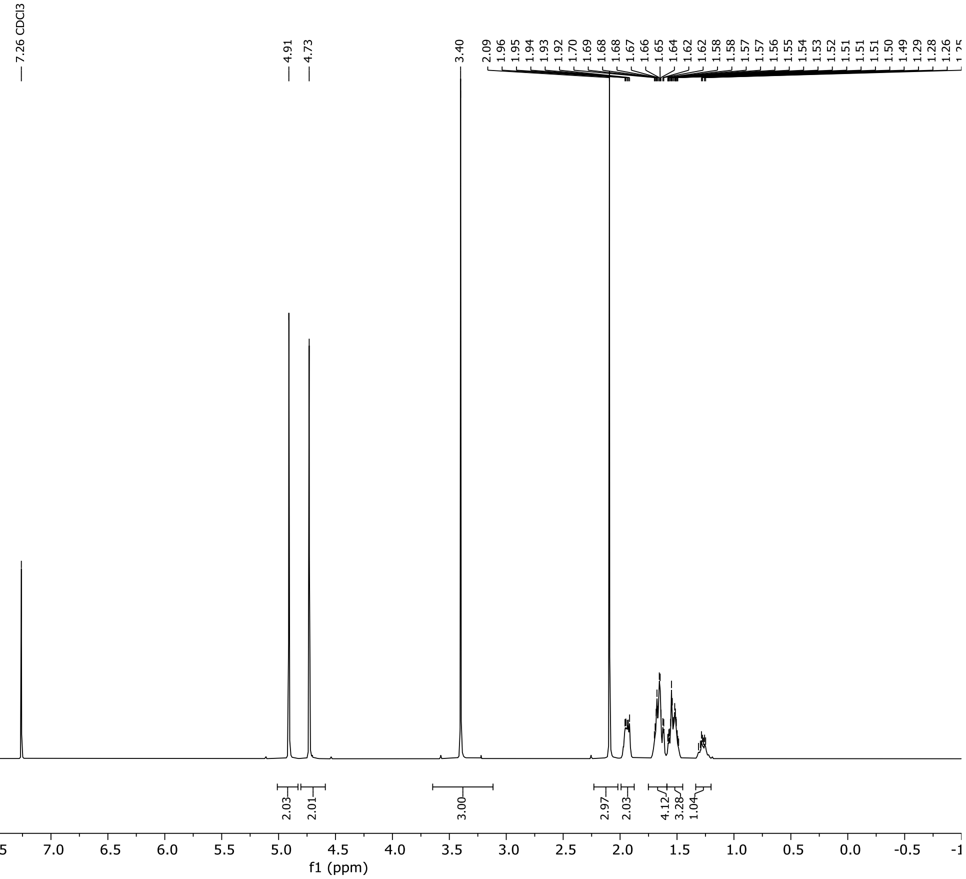

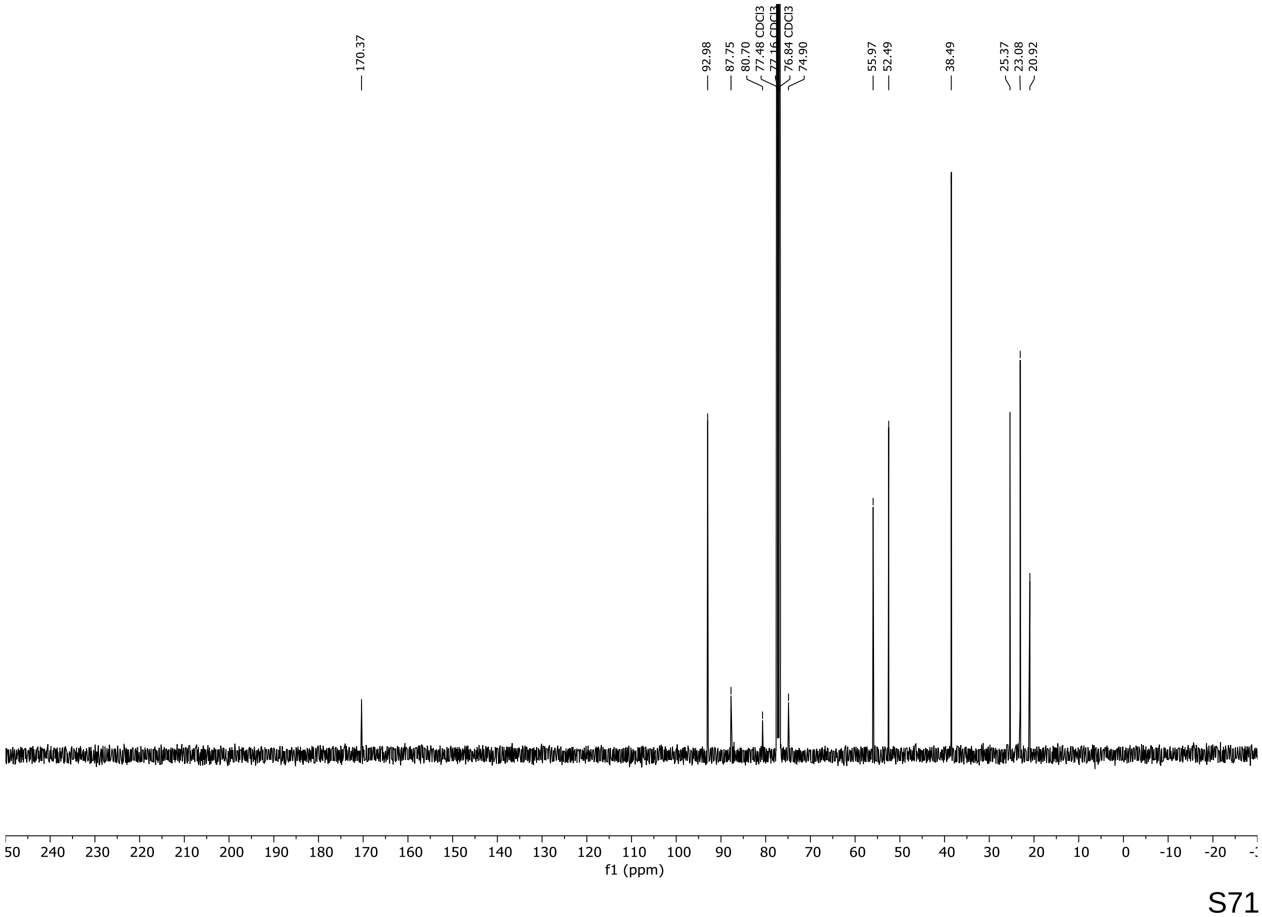

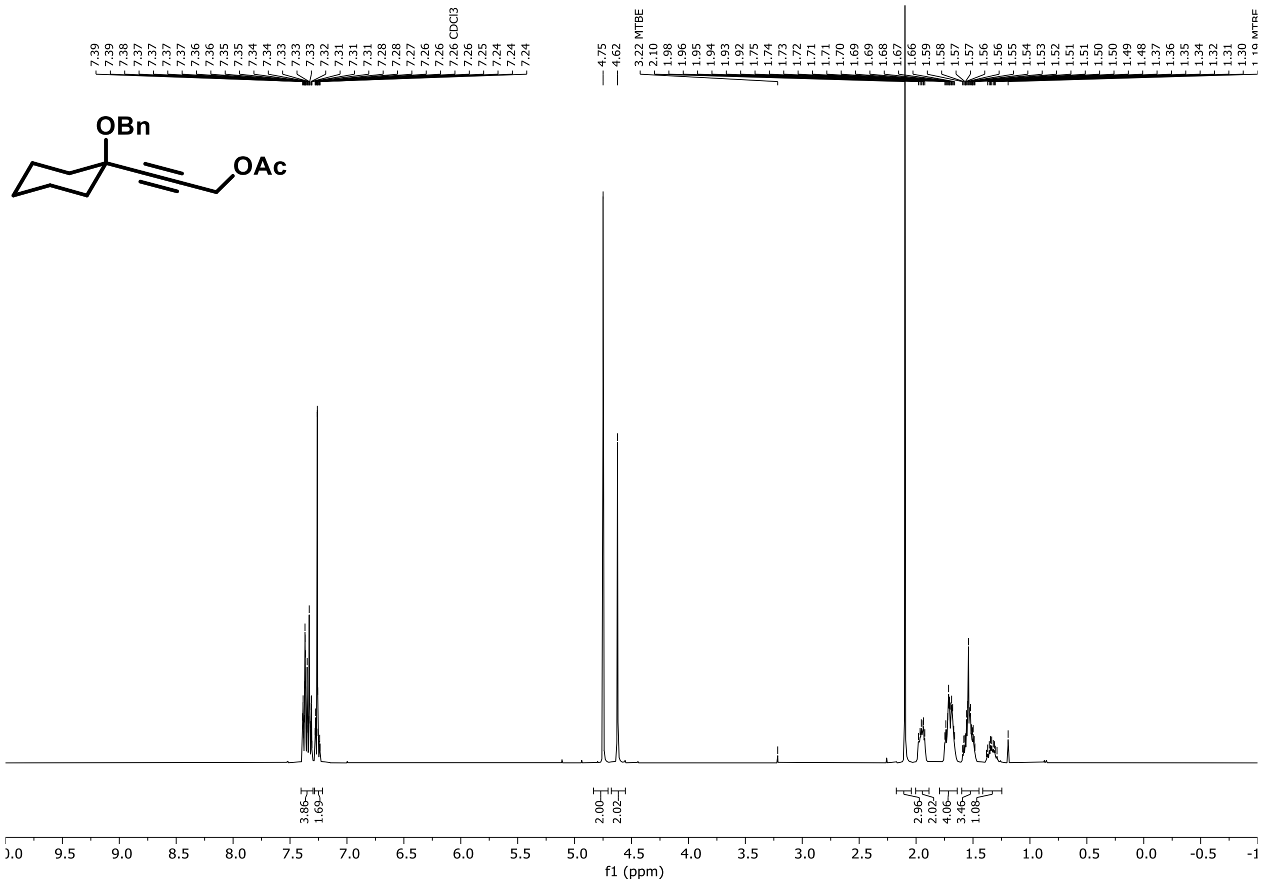

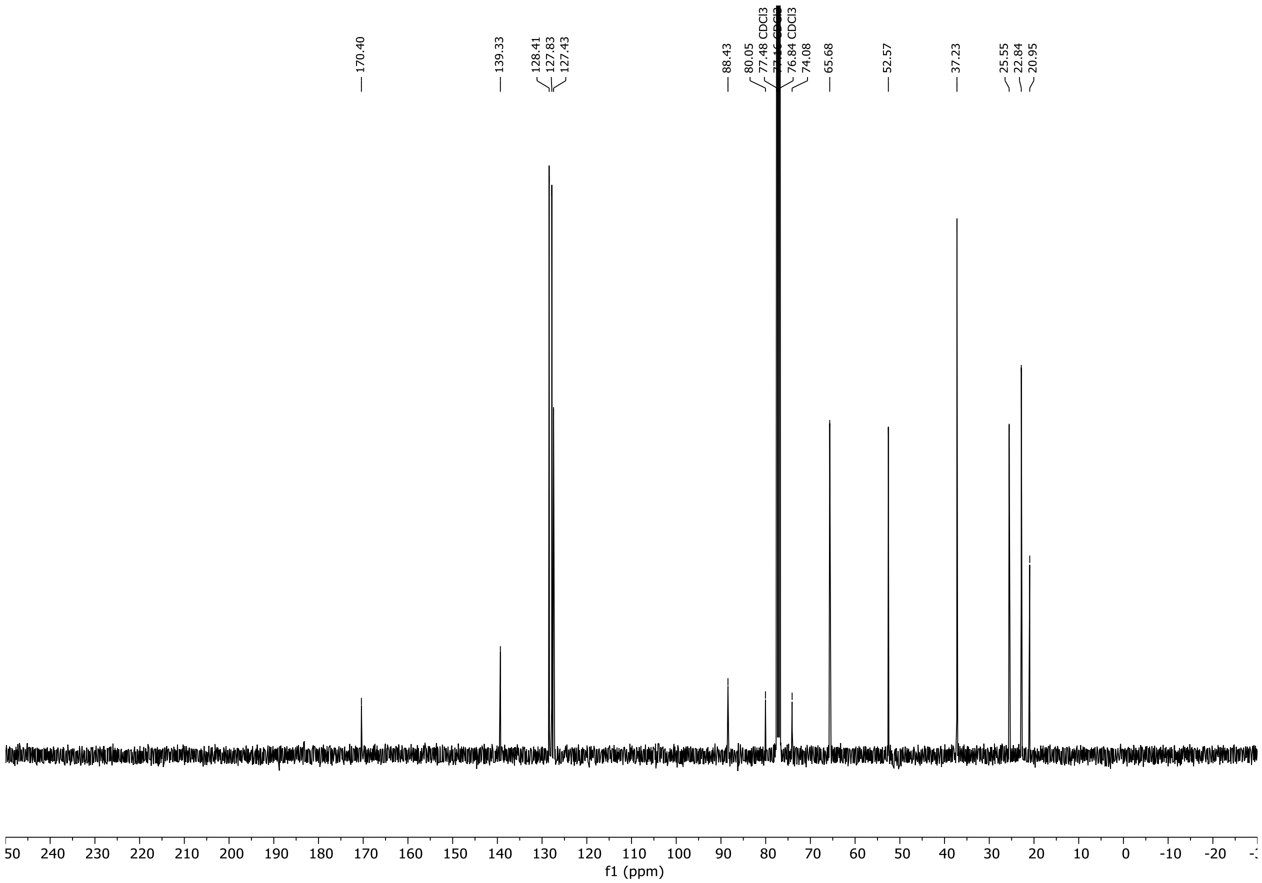

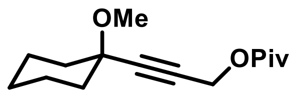
OMe
OPiv

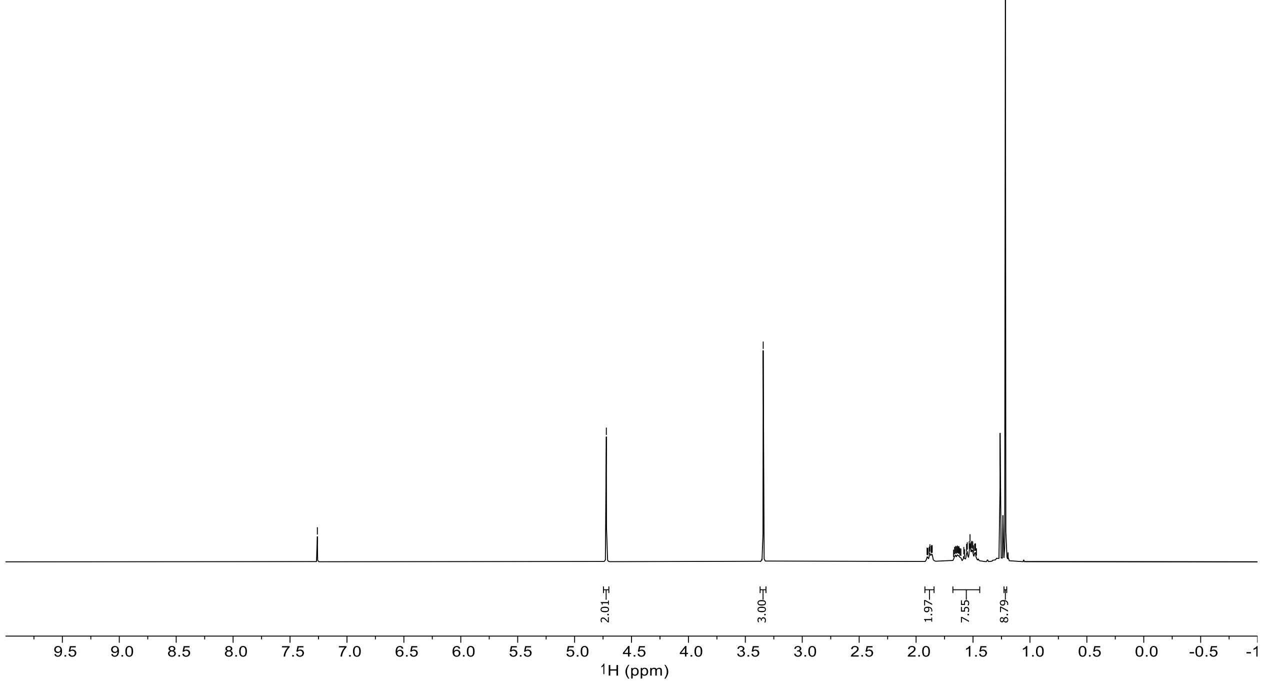
7.26 CDCl3
4.72
3.34
1.90
1.90
1.88
1.88
1.87
1.86
1.86
1.86
1.67
1.66
1.66
1.65
1.64
1.64
1.63
1.62
1.62
1.61
1.58
1.57
1.56
1.55
1.53
1.53
1.52
1.51
1.50
1.50
1.49
1.48
1.47
1.21
2.01
3.00
1.97
7.55
8.79
9.5
9.0
8.5
8.0
7.5
7.0
6.5
6.0
5.5
5.0
4.5
4.0
3.5
3.0
2.5
2.0
1.5
1.0
0.5
0.0
-0.5
1H (ppm)

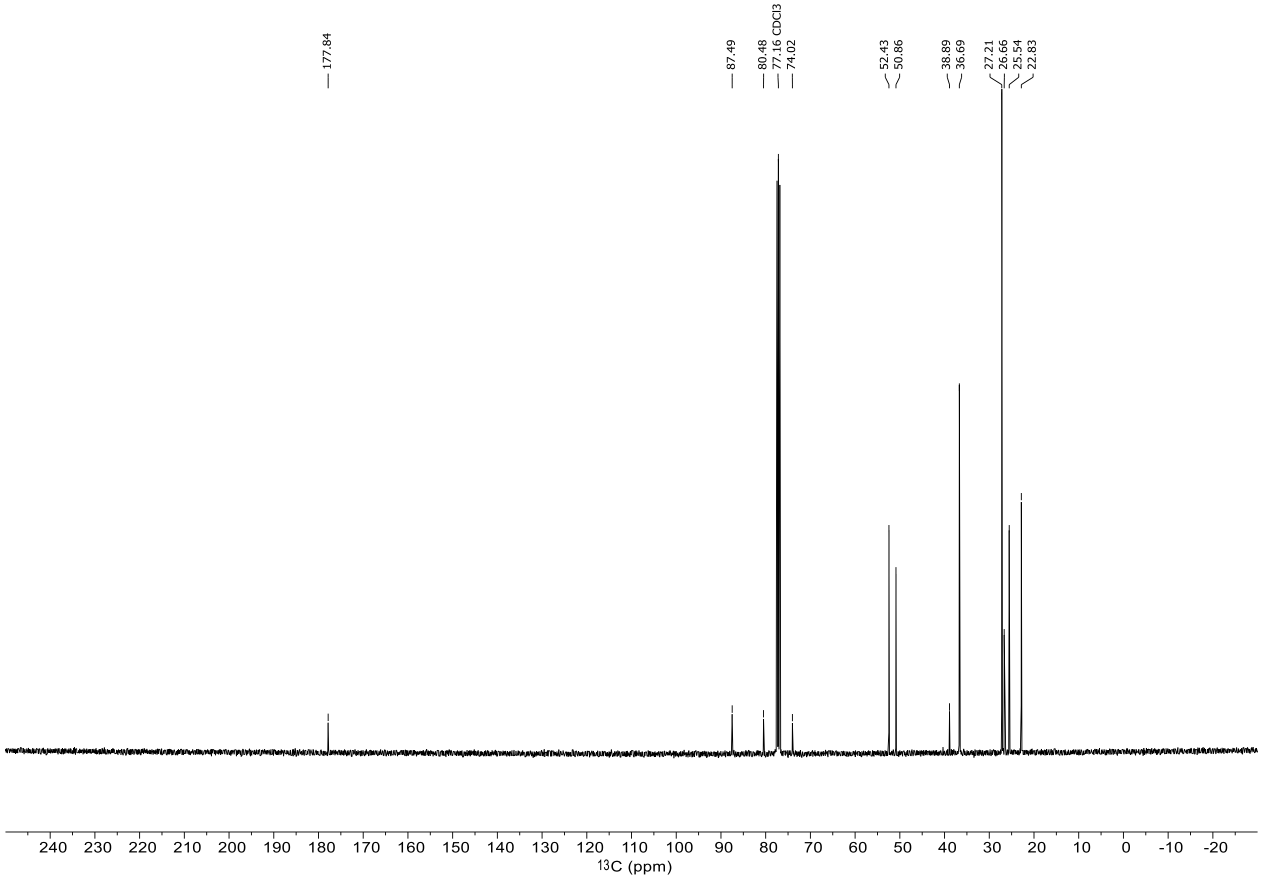
177.84
87.49
80.48
77.16 CDCl3
74.02
52.43
50.86
38.89
36.69
27.21
26.66
25.54
22.83
240
230
220
210
200
190
180
170
160
150
140
130
120
110
100
90
80
70
60
50
40
30
20
10
0
-10
-20
13C (ppm)

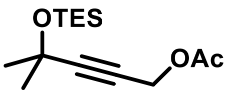
OTES
OAc

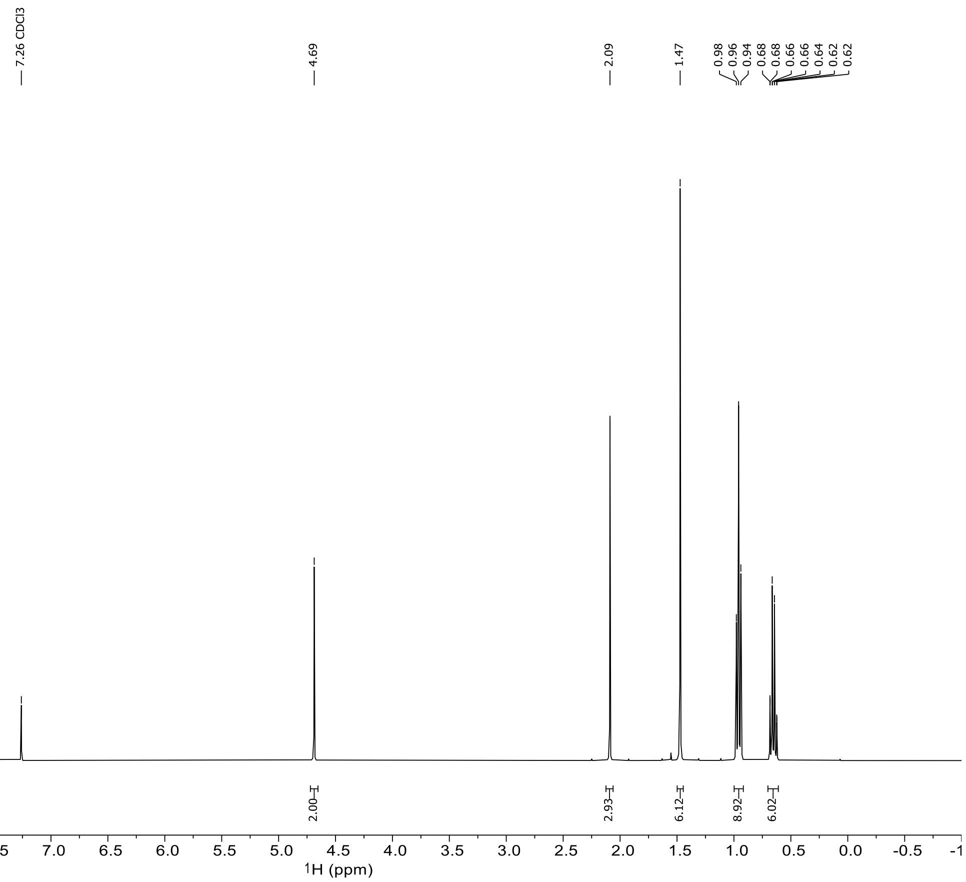
7.26 CDCl3
4.69
2.09
1.47
0.98
0.96
0.94
0.68
0.68
0.66
0.66
0.64
0.62
0.62
2.00
2.93
6.12
8.92
6.02
1H (ppm)

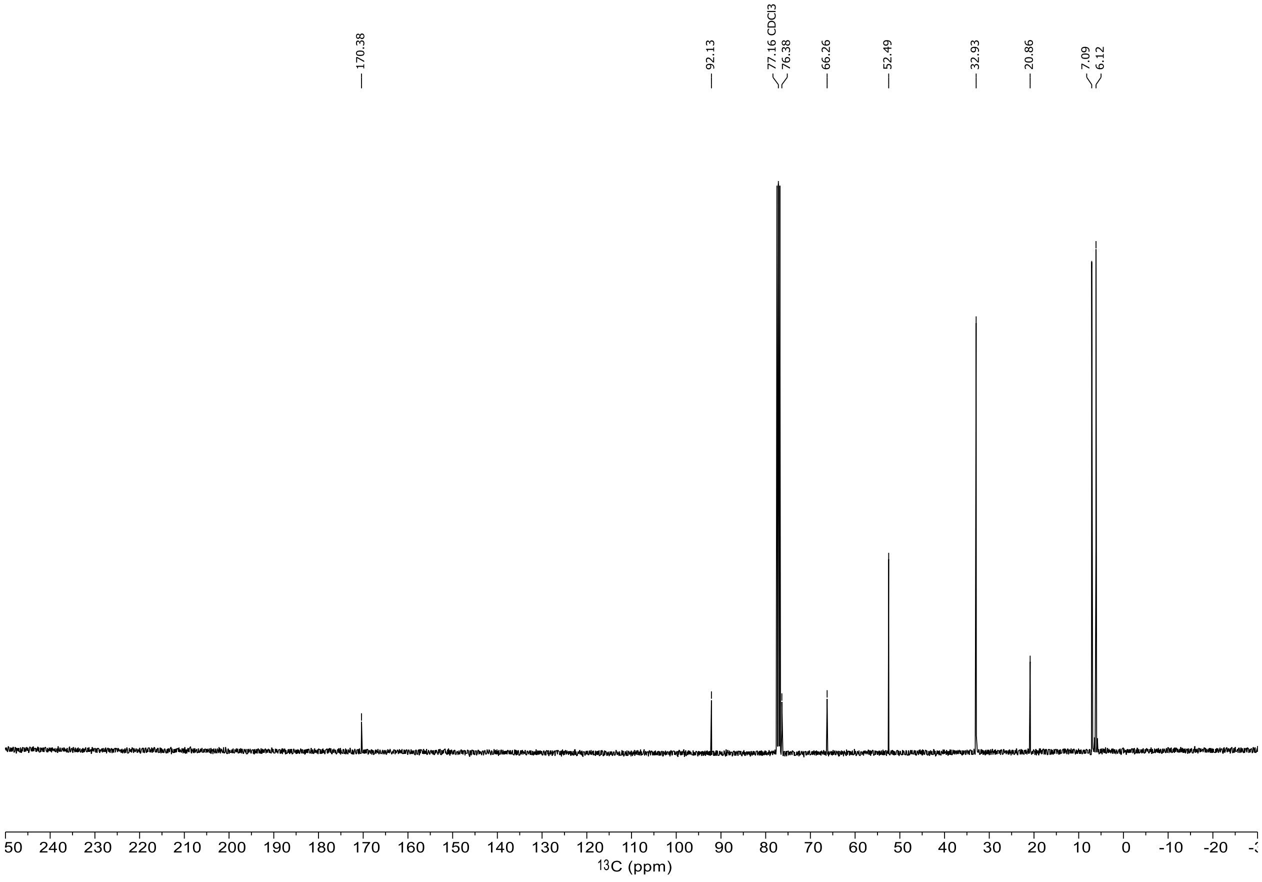
170.38
92.13
77.16 CDCl3
76.38
66.26
52.49
32.93
20.86
7.09
6.12
13C (ppm)

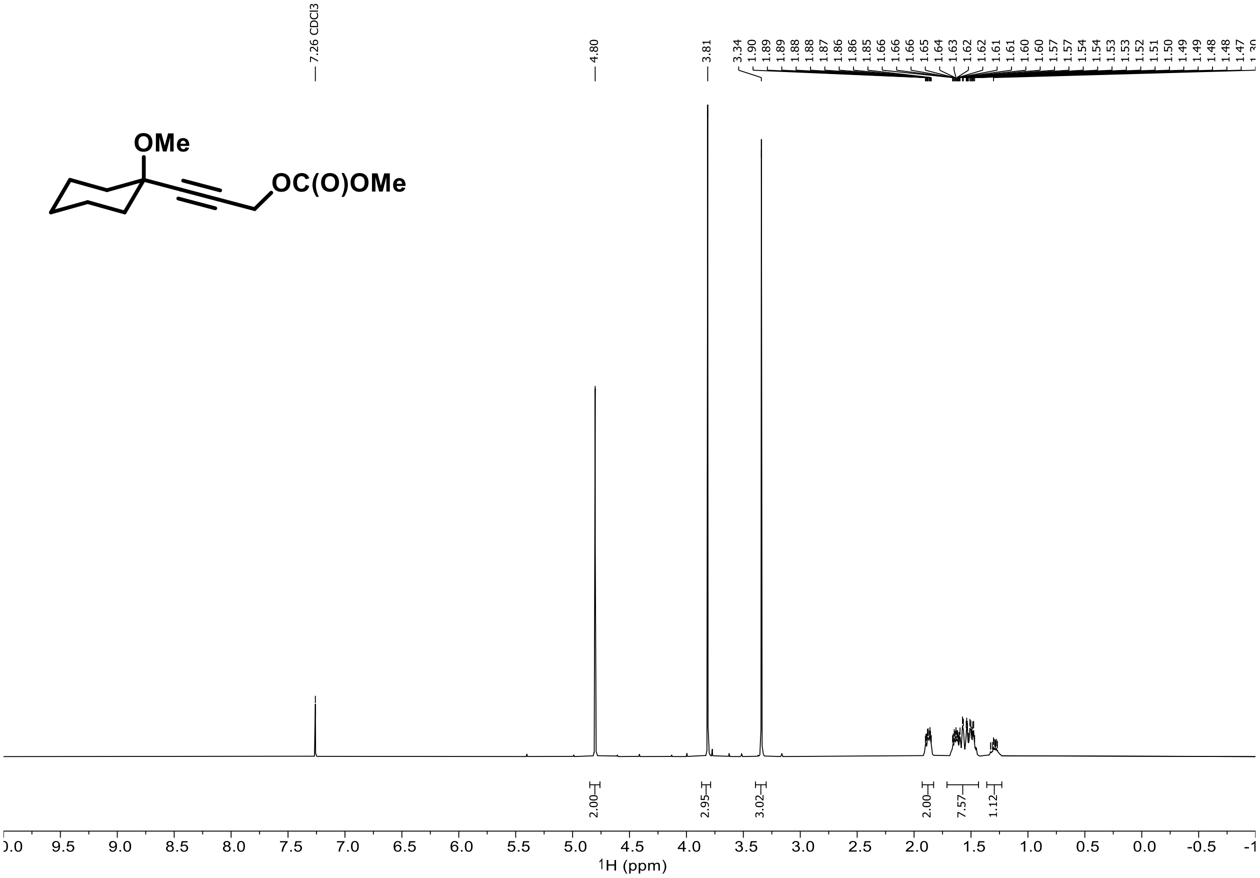
OMe
OC(O)OMe
7.26 CDCl3
4.80
3.81
3.34
1.90
1.89
1.89
1.88
1.88
1.87
1.86
1.86
1.85
1.66
1.66
1.66
1.65
1.64
1.63
1.62
1.62
1.61
1.61
1.60
1.60
1.57
1.57
1.54
1.54
1.53
1.53
1.52
1.51
1.50
1.49
1.49
1.48
1.48
1.47
2.00
2.95
3.02
2.00
7.57
1.12
1H (ppm)

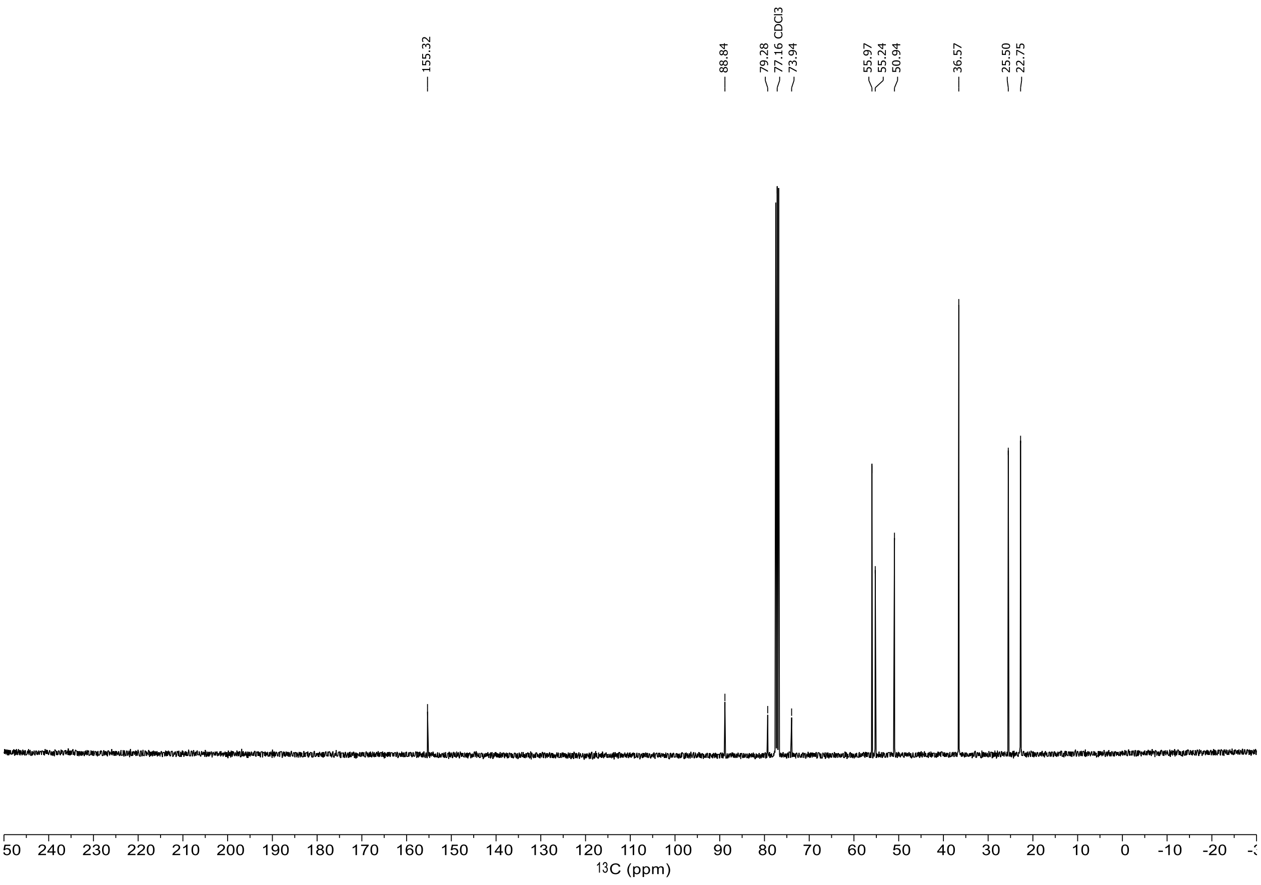
155.32
88.84
79.28
77.16 CDCl3
73.94
55.97
55.24
50.94
36.57
25.50
22.75
13C (ppm)

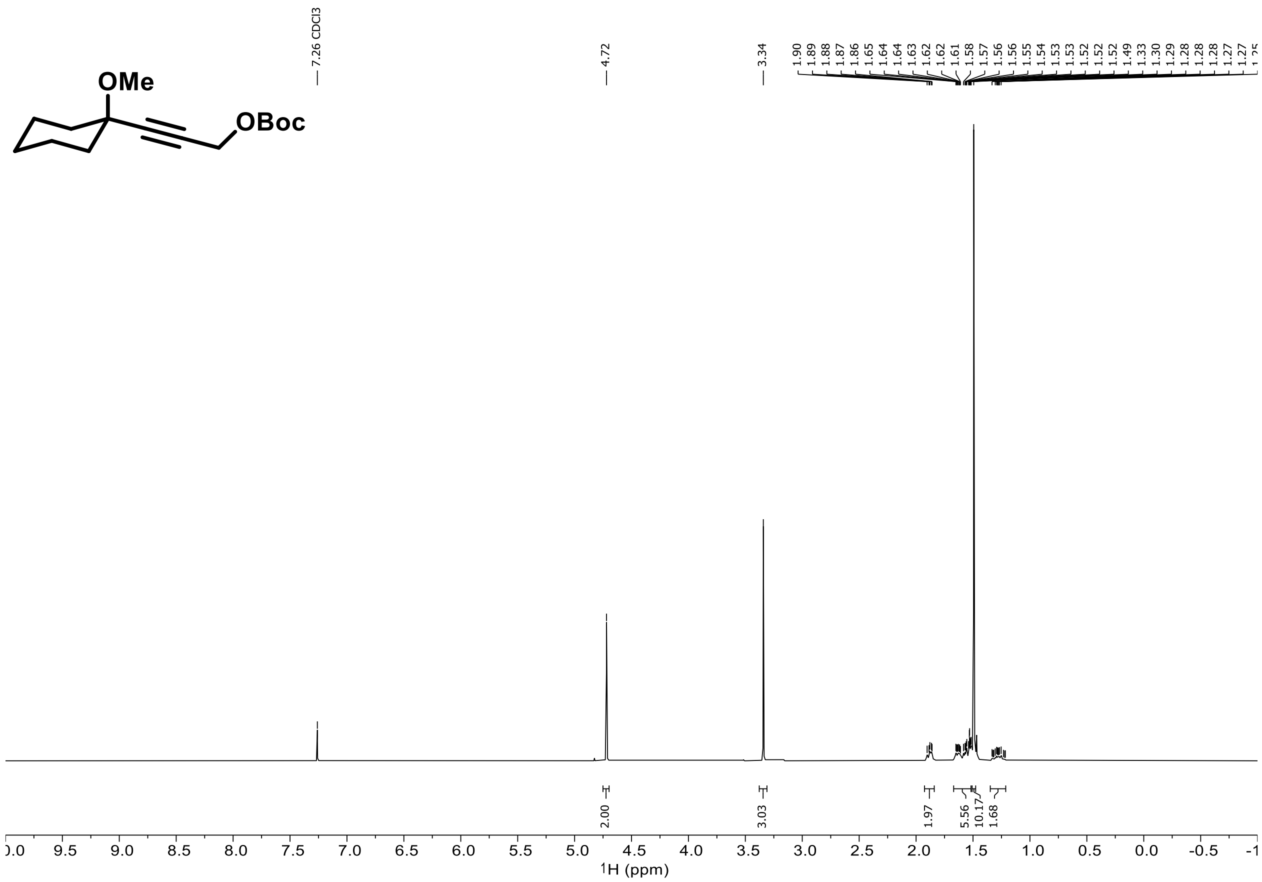
OMe
OBoc
7.26 CDCl3
4.72
3.34
1.90 1.89 1.88 1.87 1.86 1.65 1.64 1.64 1.63 1.62 1.62 1.61 1.58 1.57 1.56 1.56 1.55 1.54 1.53 1.53 1.52 1.52 1.52 1.49 1.33 1.30 1.29 1.28 1.28 1.28 1.27 1.27
2.00
3.03
1.97
5.56
10.17
1.68
1H (ppm)

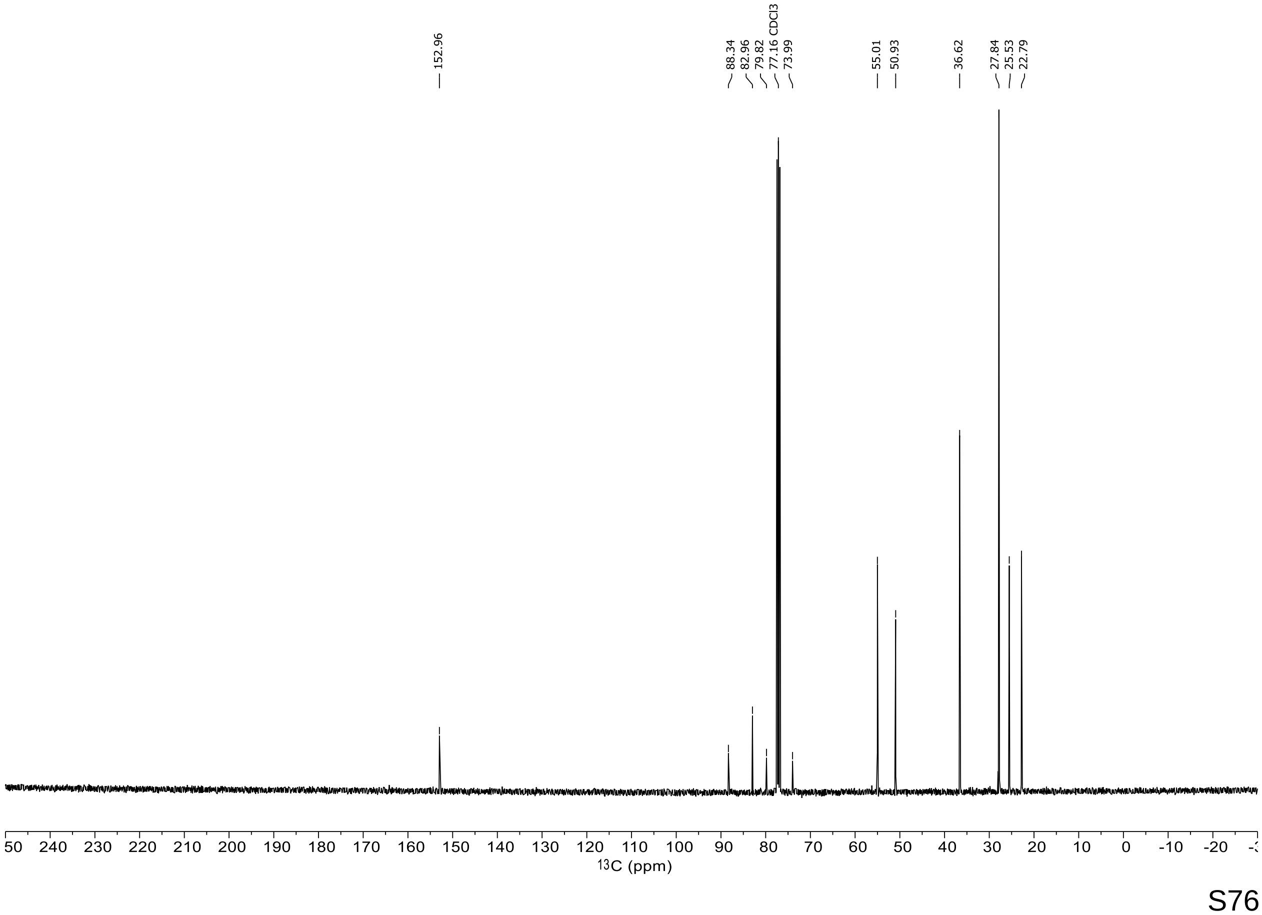
152.96
88.34
82.96
79.82
77.16 CDCl3
73.99
55.01
50.93
36.62
27.84
25.53
22.79
13C (ppm)

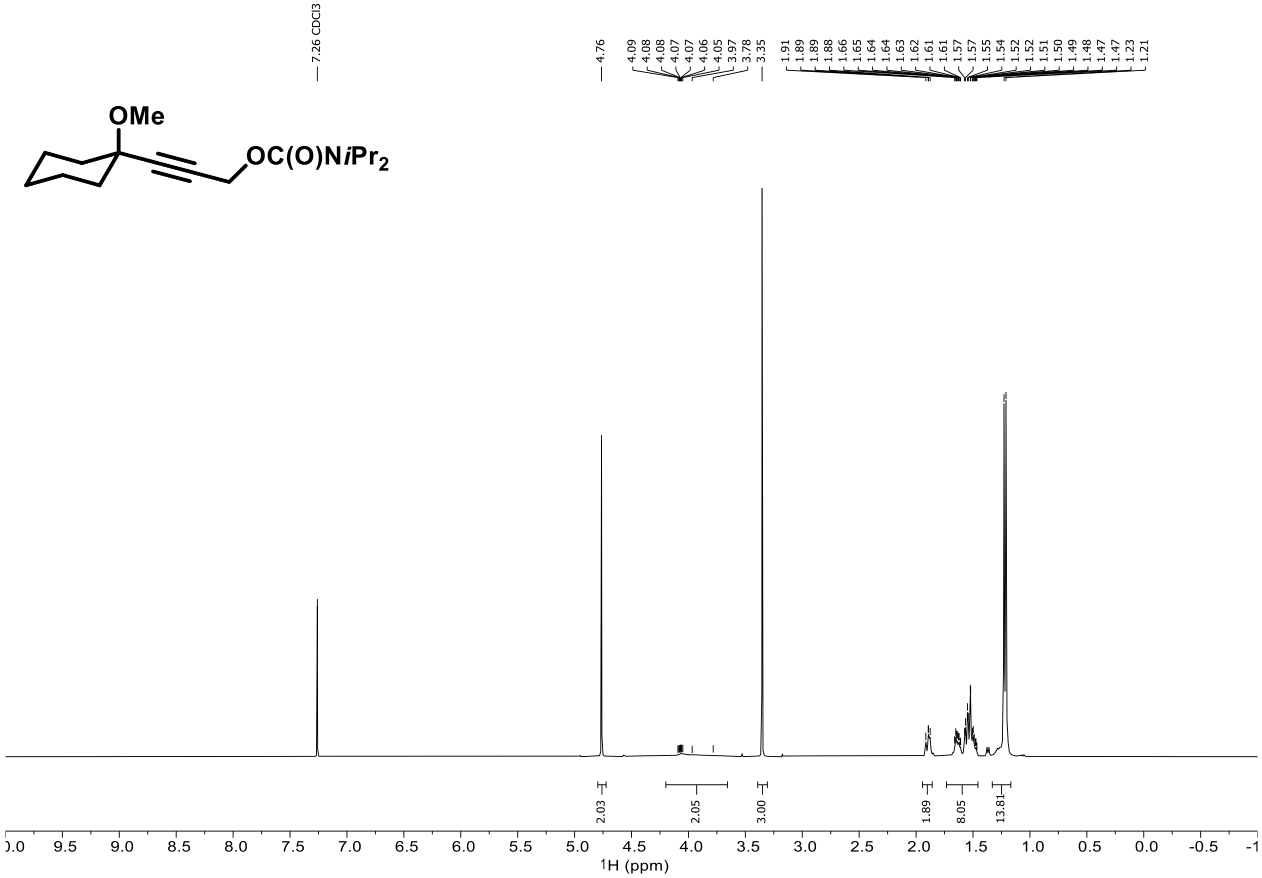
OMe
OC(O)NiPr2
7.26 CDCl3
4.76
4.09
4.08
4.08
4.07
4.07
4.06
4.05
3.97
3.78
3.35
1.91
1.89
1.89
1.88
1.66
1.65
1.64
1.64
1.63
1.62
1.61
1.61
1.57
1.57
1.57
1.55
1.54
1.52
1.52
1.51
1.50
1.49
1.48
1.47
1.47
1.23
1.21
2.03
2.05
3.00
1.89
8.05
13.81
1H (ppm)

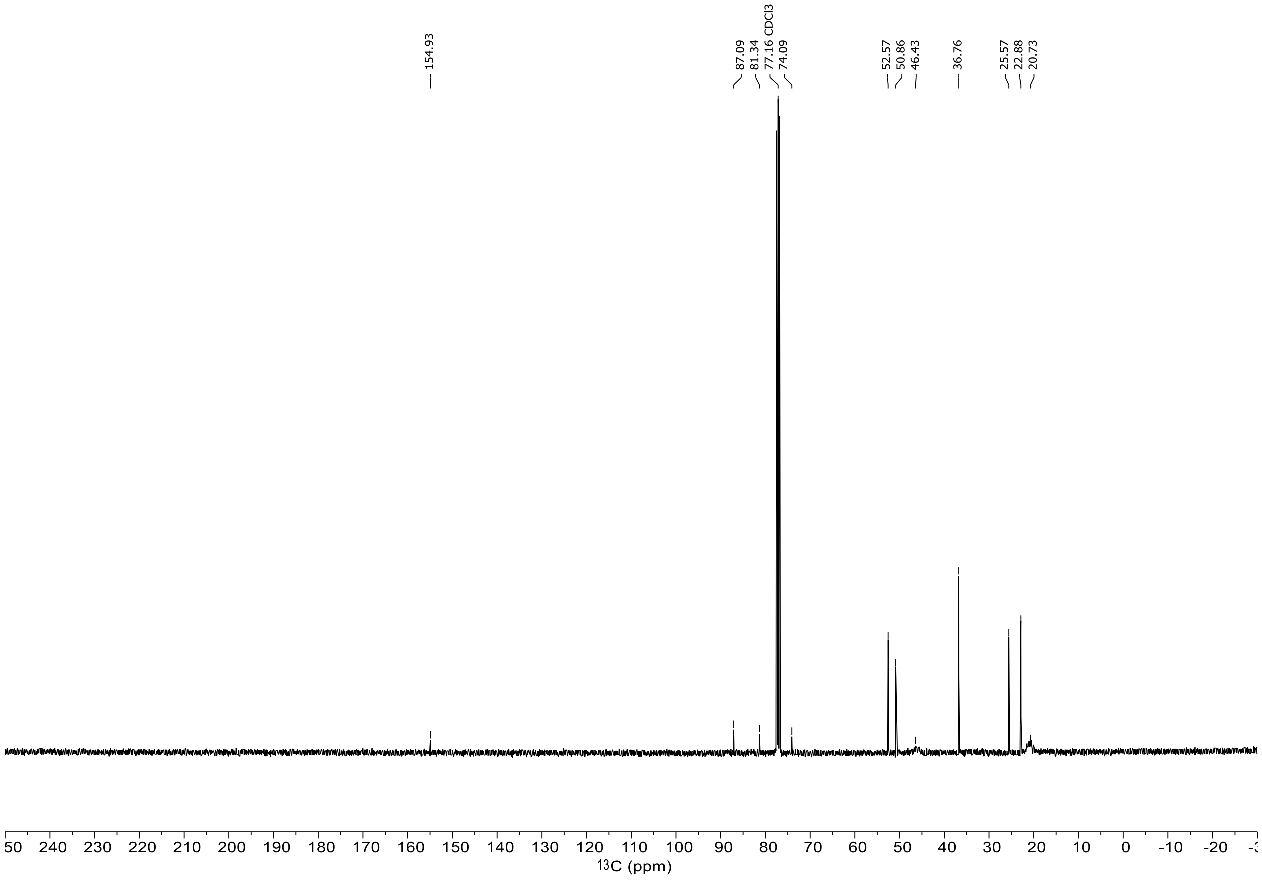
154.93
87.09
81.34
77.16 CDCl3
74.09
52.57
50.86
46.43
36.76
25.57
22.88
20.73
13C (ppm)

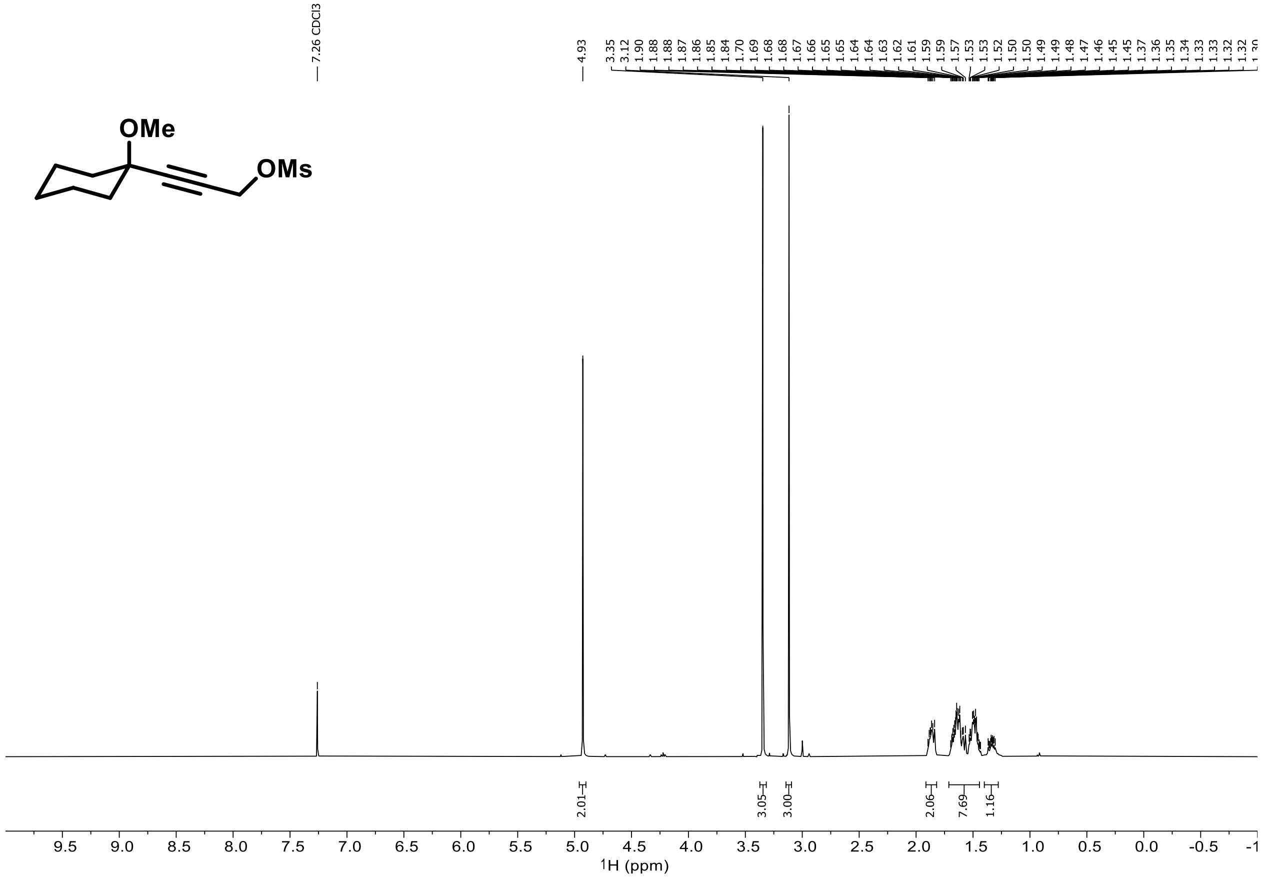
OMe
OMs
7.26 CDCl3
4.93
2.01
3.05
3.00
2.06
7.69
1.16
1H (ppm)

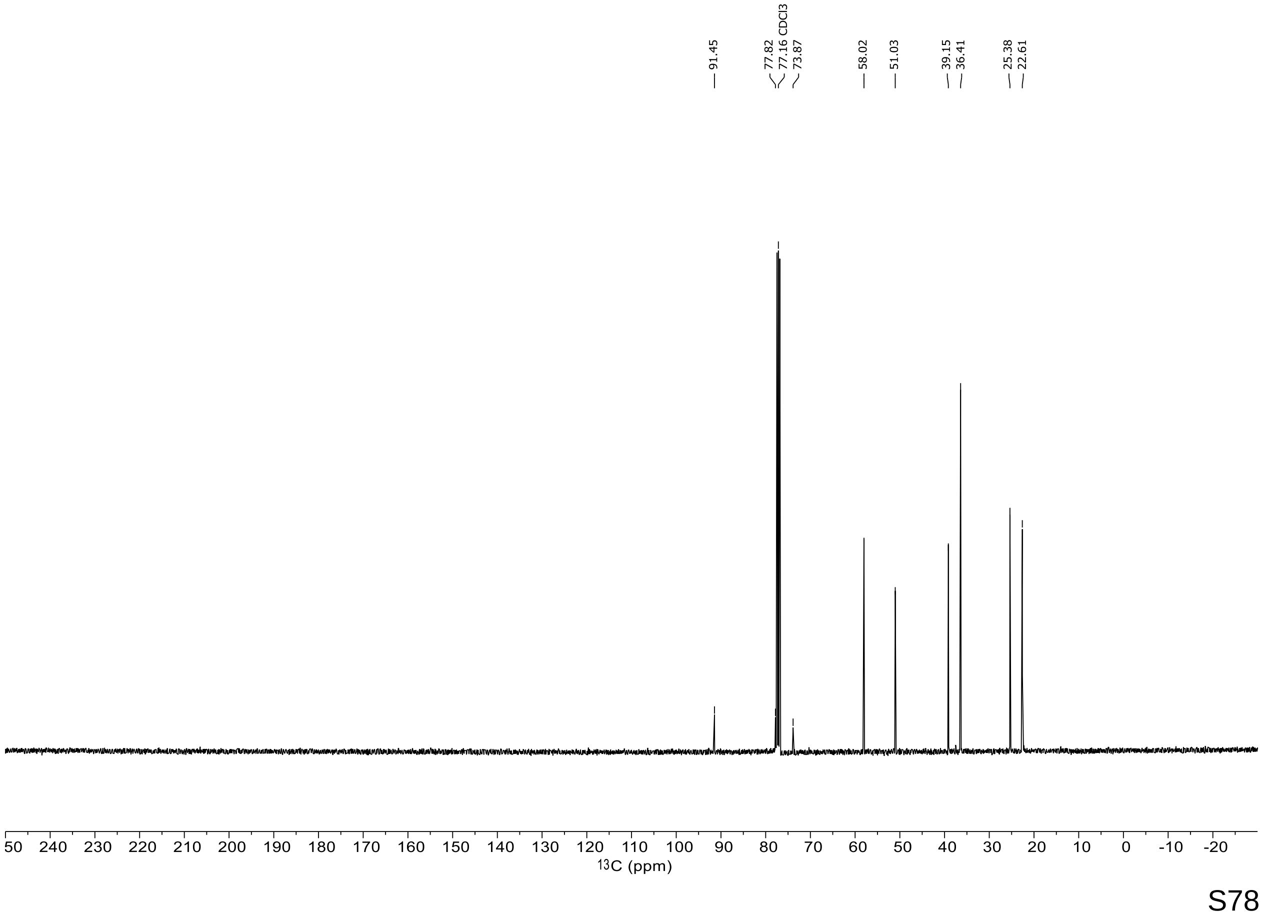
91.45
77.82
77.16 CDCl3
73.87
58.02
51.03
39.15
36.41
25.38
22.61
13C (ppm)

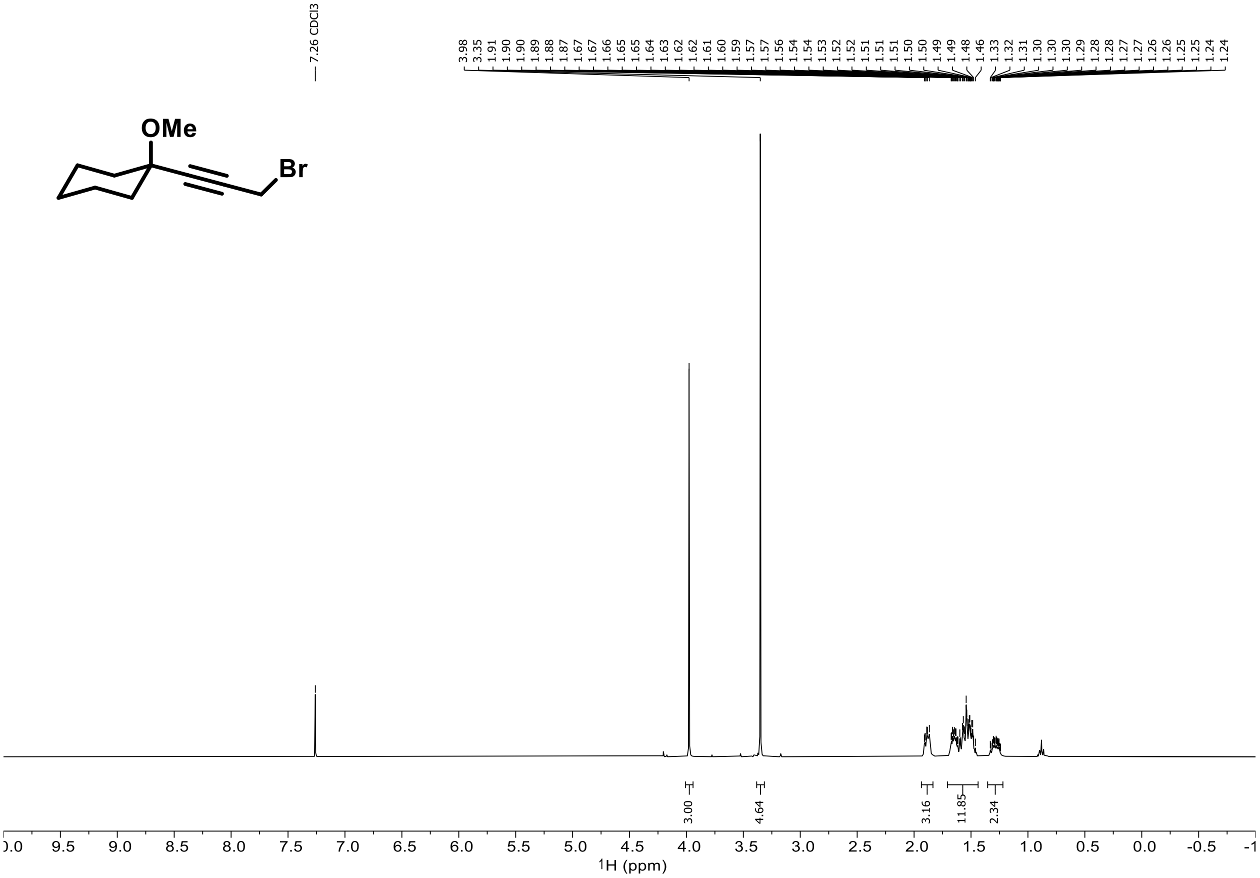
OMe
Br
7.26 CDCl3
3.98 3.35 1.91 1.90 1.90 1.89 1.88 1.87 1.67 1.67 1.66 1.65 1.65 1.64 1.63 1.62 1.62 1.61 1.60 1.59 1.57 1.57 1.56 1.54 1.54 1.53 1.52 1.52 1.51 1.51 1.51 1.50 1.49 1.49 1.48 1.46 1.33 1.32 1.31 1.30 1.30 1.30 1.29 1.28 1.28 1.27 1.27 1.26 1.26 1.25 1.25 1.24 1.24
3.00
4.64
3.16
11.85
2.34
1H (ppm)

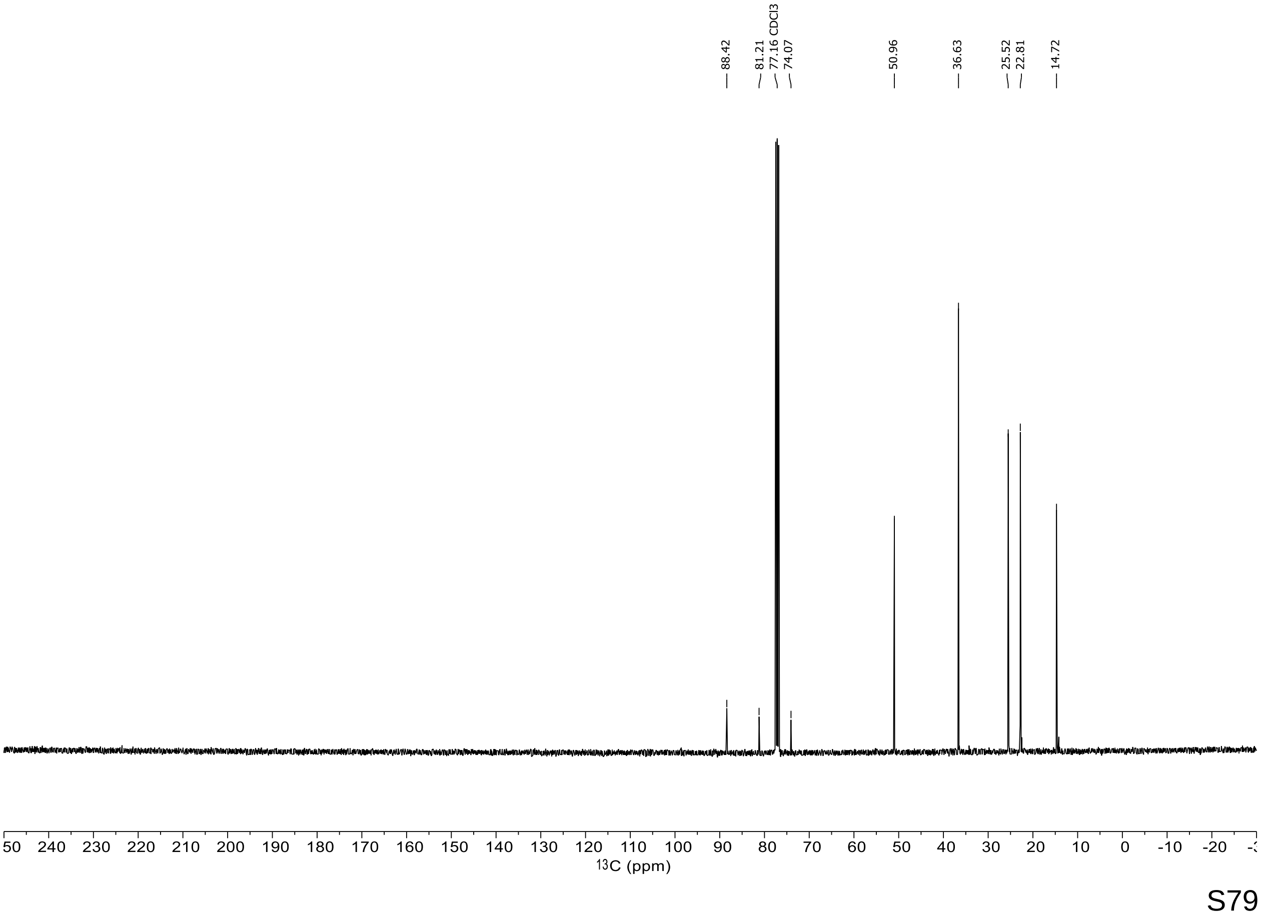
88.42
81.21
77.16 CDCl3
74.07
50.96
36.63
25.52
22.81
14.72
13C (ppm)

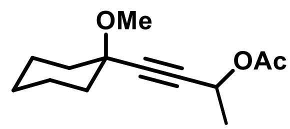
OMe
OAc

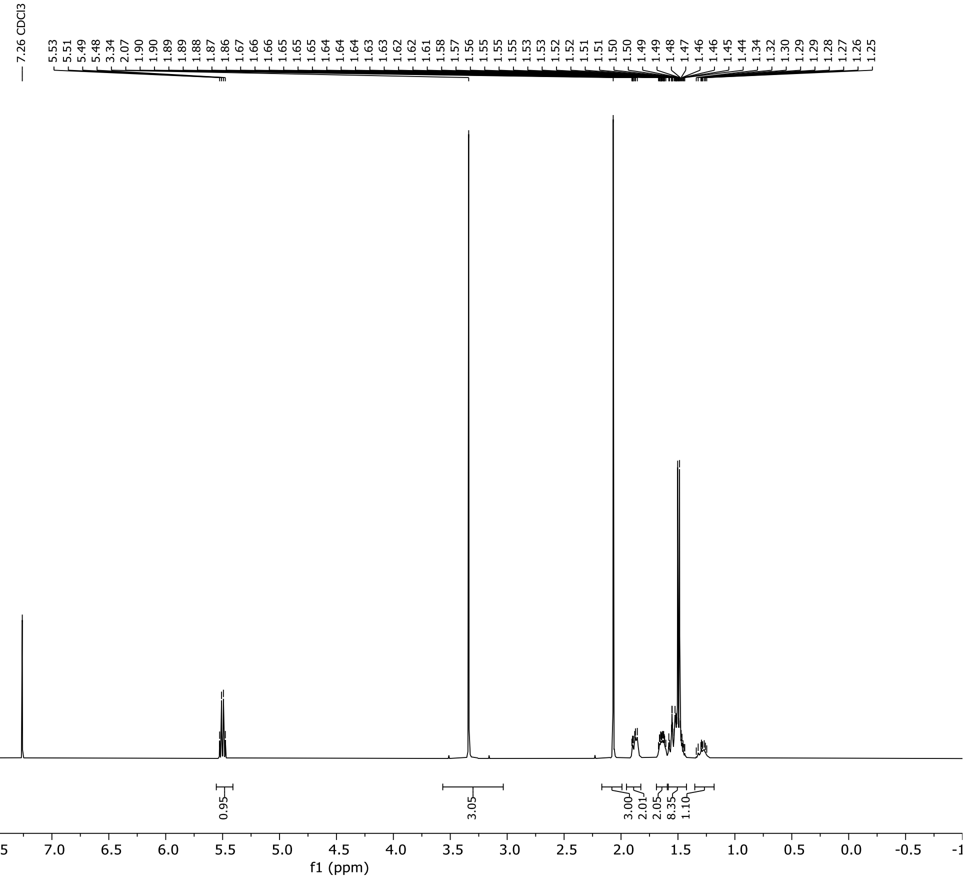
7.26 CDCl3
5.53
5.51
5.49
5.48
3.34
2.07
1.90
1.90
1.89
1.89
1.88
1.87
1.86
1.67
1.66
1.66
1.65
1.65
1.65
1.64
1.64
1.64
1.63
1.63
1.62
1.62
1.61
1.58
1.57
1.56
1.55
1.55
1.55
1.53
1.53
1.52
1.52
1.51
1.51
1.50
1.50
1.49
1.49
1.48
1.47
1.46
1.46
1.45
1.44
1.34
1.32
1.30
1.29
1.29
1.28
1.27
1.26
1.25
0.95
3.05
3.00
2.01
2.05
8.35
1.10
f1 (ppm)

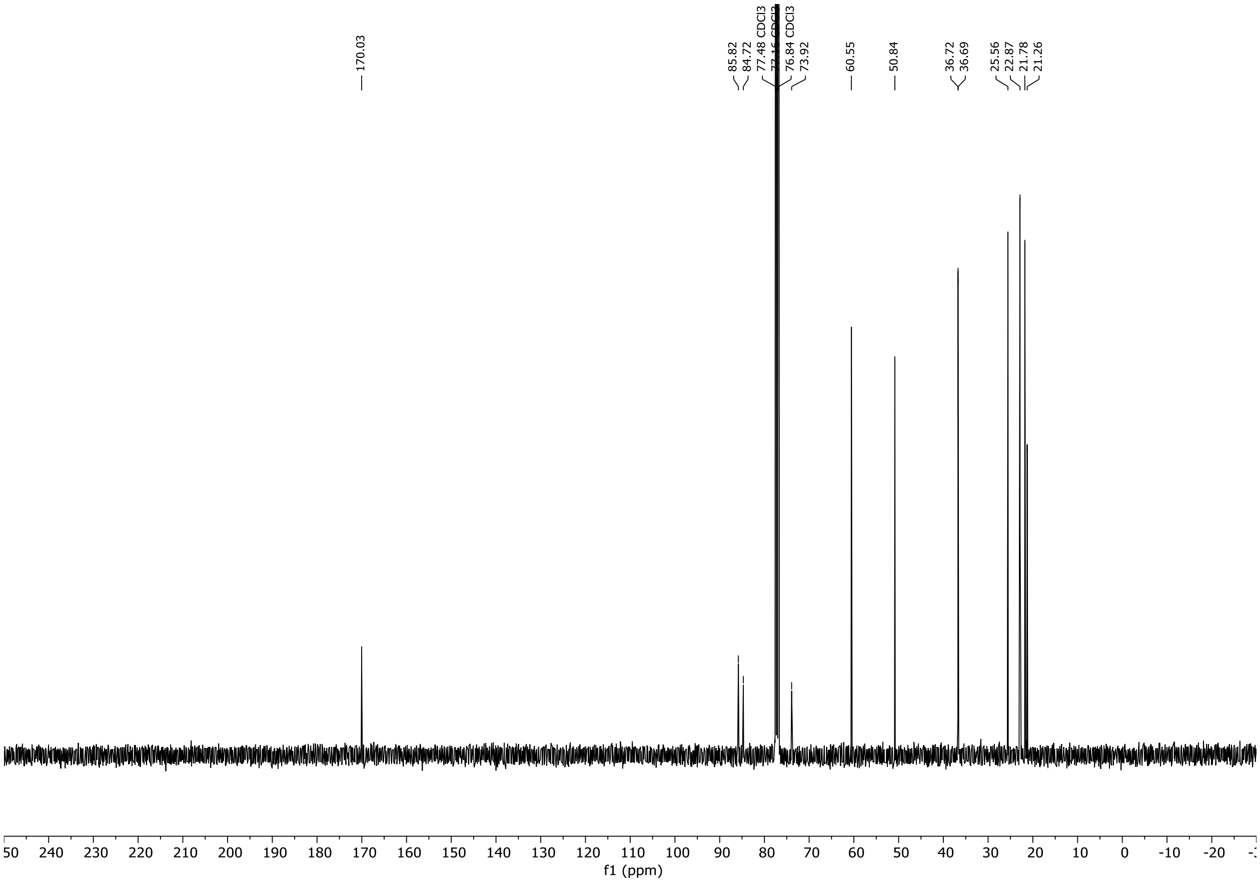
170.03
85.82
84.72
77.48 CDCl3
77.16 CDCl3
76.84 CDCl3
73.92
60.55
50.84
36.72
36.69
25.56
22.87
21.78
21.26
f1 (ppm)

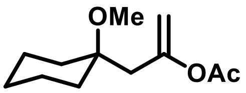
OMe
OAc

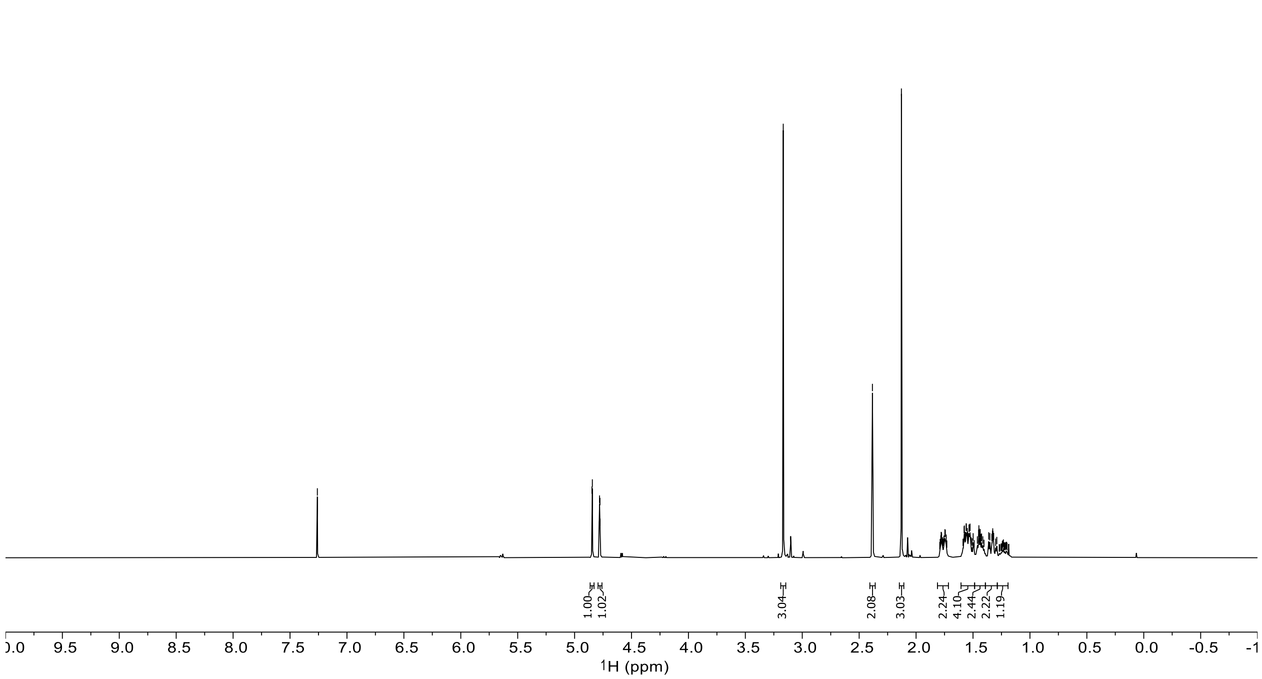
7.26 CDCl3
4.85
4.84
4.78
4.78
4.78
4.78
4.78
4.78
3.17
2.38
2.13
1.79
1.79
1.78
1.78
1.78
1.78
1.77
1.77
1.76
1.76
1.75
1.75
1.74
1.74
1.73
1.59
1.58
1.58
1.57
1.57
1.57
1.56
1.56
1.55
1.55
1.54
1.54
1.53
1.53
1.53
1.52
1.51
1.50
1.49
1.46
1.45
1.45
1.45
1.44
1.44
1.43
1.43
1.42
1.41
1.40
1.36
1.35
1.35
1.33
1.33
1.33
1.32
1.32
1.30
1.29
1.27
1.26
1.25
1.25
1.25
1.24
1.24
1.23
1.23
1.22
1.22
1.22
1.21
1.20
1.20
1.00
1.02
3.04
2.08
3.03
2.24
4.10
2.44
2.22
1.19
9.5
9.0
8.5
8.0
7.5
7.0
6.5
6.0
5.5
5.0
4.5
4.0
3.5
3.0
2.5
2.0
1.5
1.0
0.5
0.0
-0.5
1H (ppm)

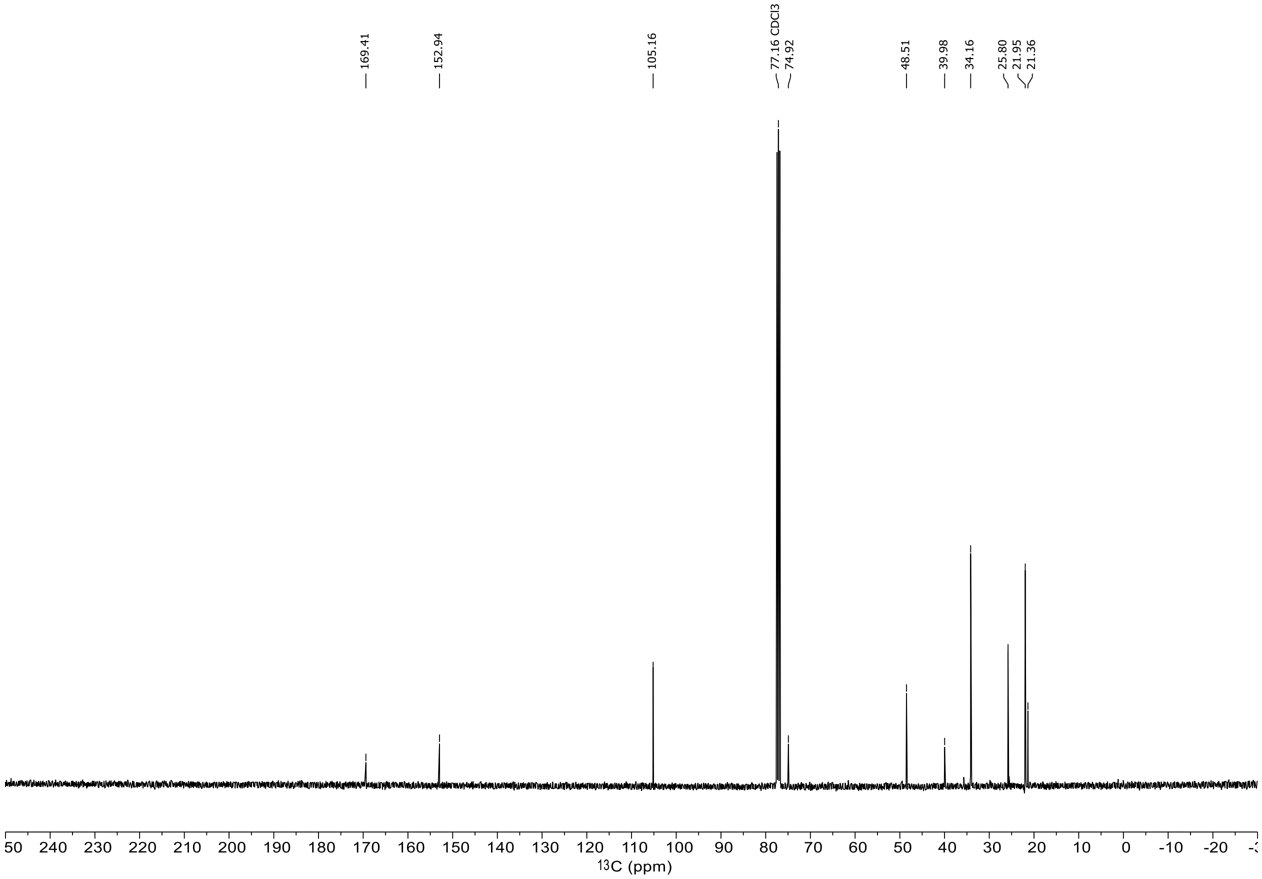
169.41
152.94
105.16
77.16 CDCl3
74.92
48.51
39.98
34.16
25.80
21.95
21.36
240
230
220
210
200
190
180
170
160
150
140
130
120
110
100
90
80
70
60
50
40
30
20
10
0
-10
-20
13C (ppm)

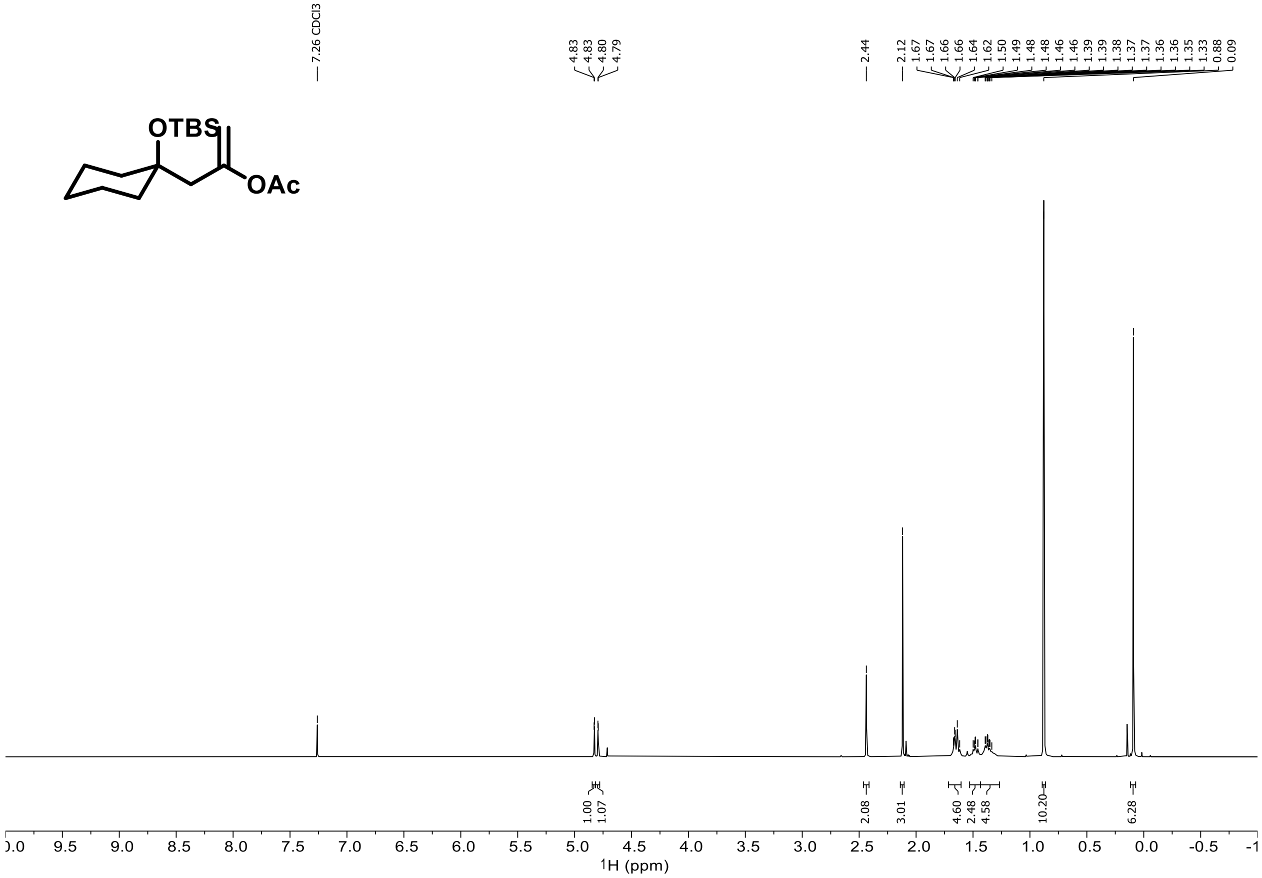
7.26 CDCl3
4.83
4.83
4.80
4.79
2.44
2.12
1.67
1.67
1.66
1.66
1.64
1.62
1.50
1.49
1.48
1.48
1.46
1.46
1.39
1.39
1.38
1.37
1.37
1.36
1.36
1.35
1.33
0.88
0.09
OTBS
OAc
1.00
1.07
2.08
3.01
4.60
2.48
4.58
10.20
6.28
1H (ppm)

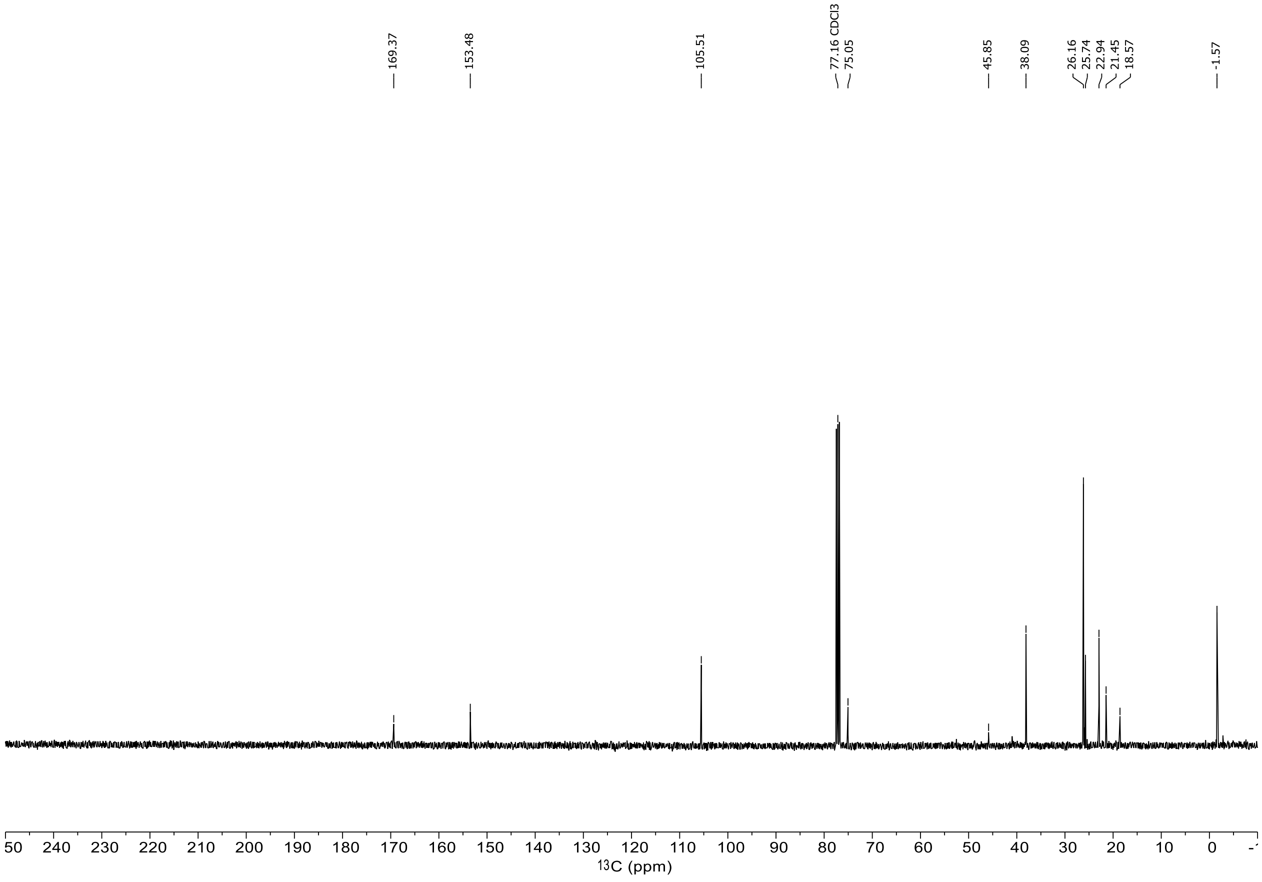
169.37
153.48
105.51
77.16 CDCl3
75.05
45.85
38.09
26.16
25.74
22.94
21.45
18.57
-1.57
13C (ppm)

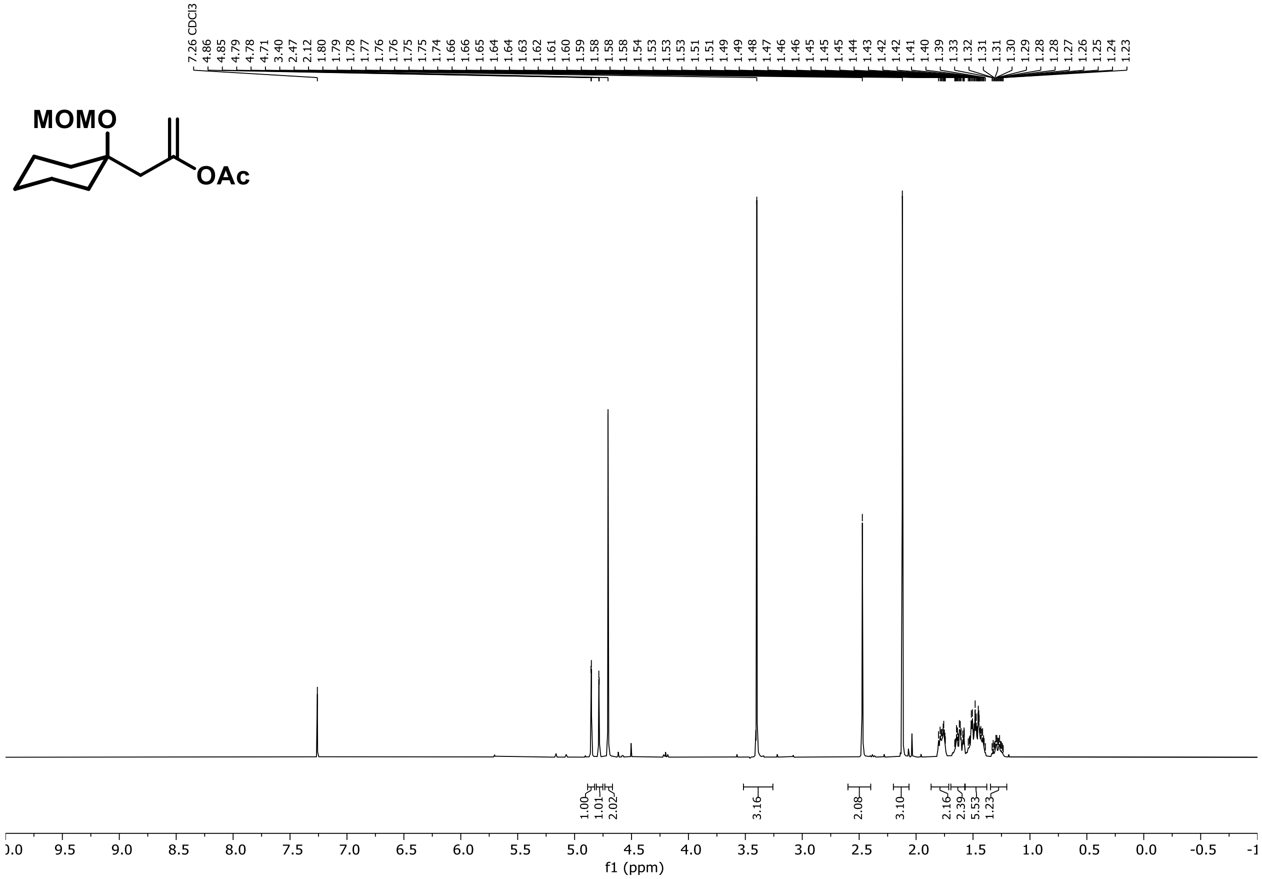
MOMO
OAc
7.26 CDCl3
4.86
4.85
4.79
4.78
4.71
3.40
2.47
2.12
1.00
1.01
2.02
3.16
2.08
3.10
2.16
2.39
5.53
1.23
f1 (ppm)

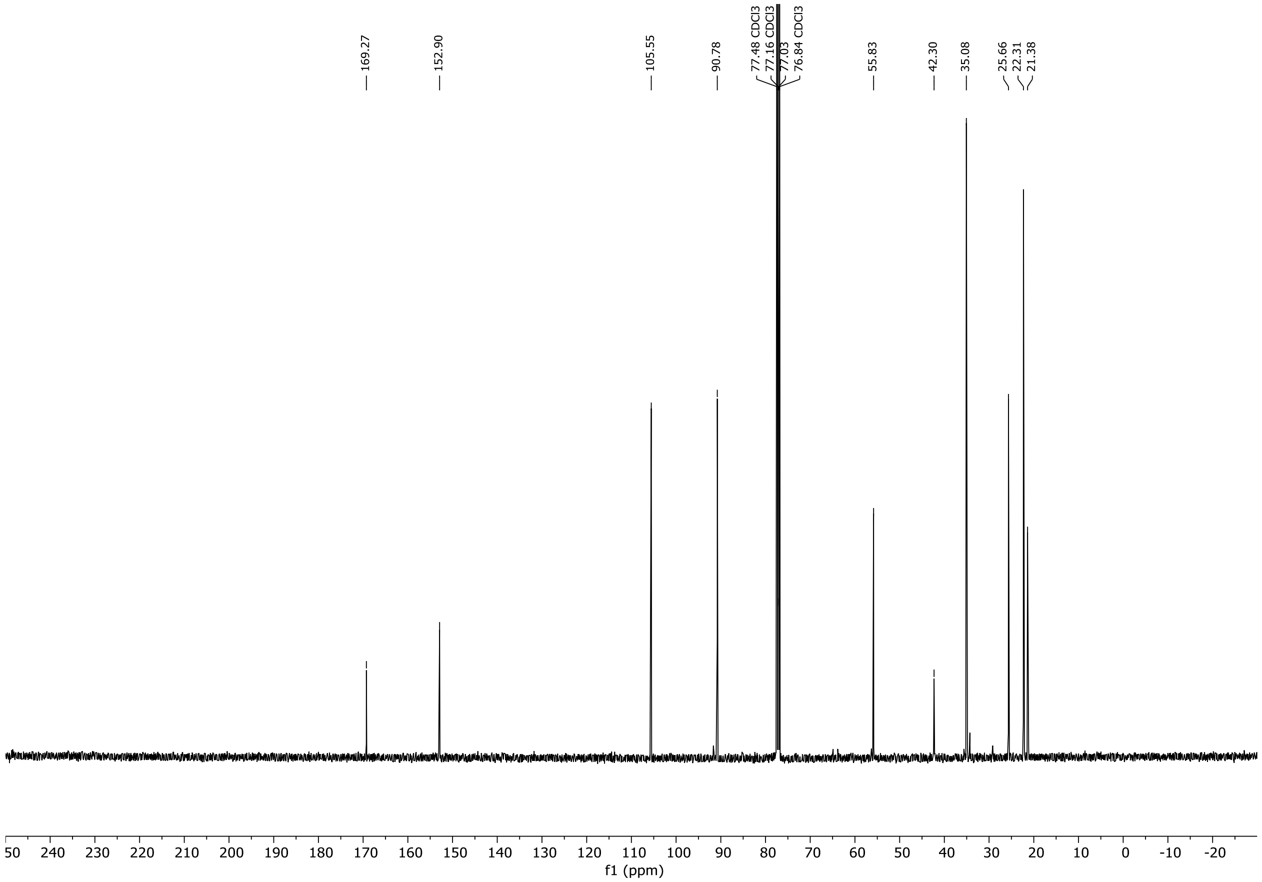
169.27
152.90
105.55
90.78
77.48 CDCl3
77.16 CDCl3
77.03
76.84 CDCl3
55.83
42.30
35.08
25.66
22.31
21.38
f1 (ppm)

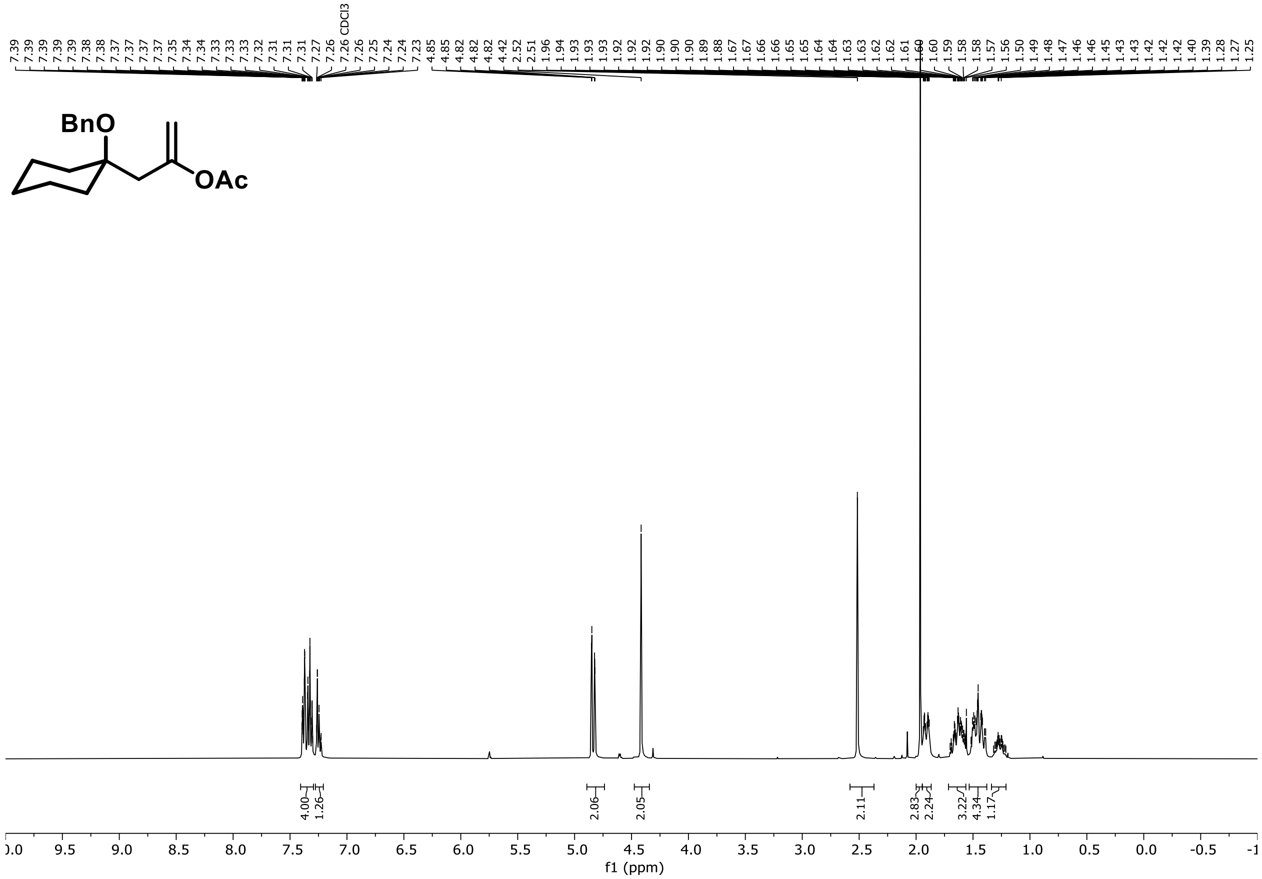
BnO
OAc
7.39 7.39 7.39 7.39 7.39 7.38 7.38 7.37 7.37 7.37 7.37 7.35 7.34 7.34 7.33 7.33 7.33 7.32 7.31 7.31 7.27 7.26 CDCl3 7.26 7.25 7.24 7.24 7.23 4.85 4.85 4.82 4.82 4.82 4.42 2.52 2.51 1.96 1.94 1.93 1.93 1.93 1.92 1.92 1.92 1.92 1.90 1.90 1.90 1.89 1.88 1.67 1.67 1.66 1.66 1.65 1.65 1.64 1.64 1.63 1.63 1.62 1.62 1.61 1.60 1.60 1.59 1.58 1.58 1.57 1.56 1.50 1.49 1.48 1.47 1.46 1.46 1.45 1.43 1.43 1.42 1.42 1.42 1.40 1.39 1.28 1.27 1.25
4.00
1.26
2.06
2.05
2.11
2.83
2.24
3.22
4.34
1.17
9.5 9.0 8.5 8.0 7.5 7.0 6.5 6.0 5.5 5.0 4.5 4.0 3.5 3.0 2.5 2.0 1.5 1.0 0.5 0.0 -0.5
f1 (ppm)

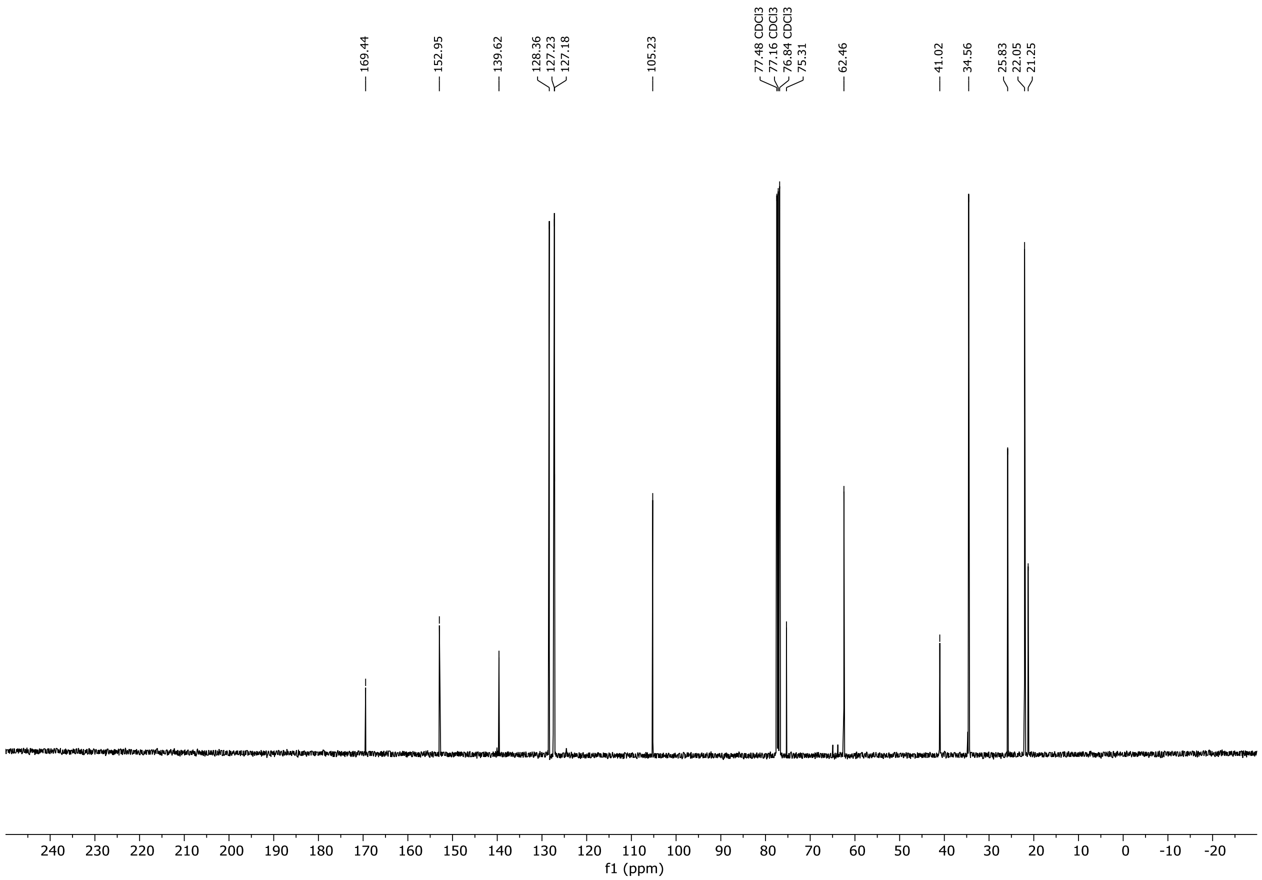
169.44
152.95
139.62
128.36
127.23
127.18
105.23
77.48 CDCl3
77.16 CDCl3
76.84 CDCl3
75.31
62.46
41.02
34.56
25.83
22.05
21.25
240 230 220 210 200 190 180 170 160 150 140 130 120 110 100 90 80 70 60 50 40 30 20 10 0 -10 -20
f1 (ppm)

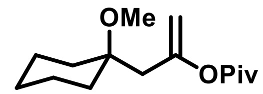

OMe
OPiv

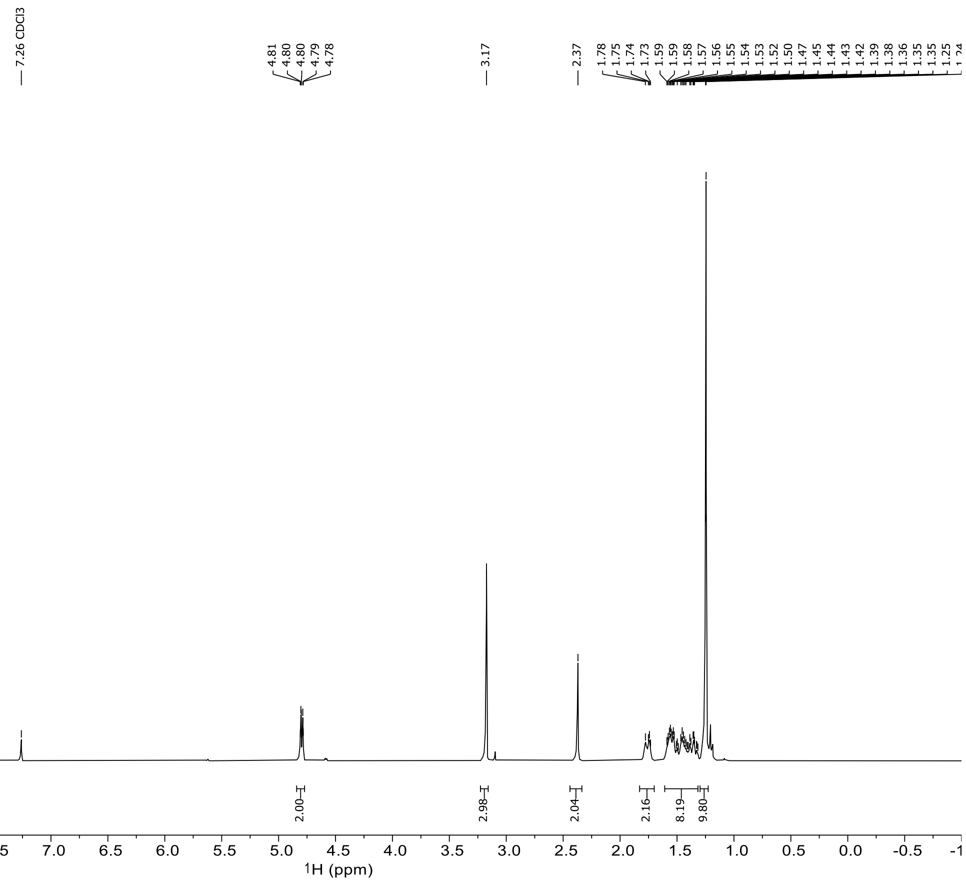

7.26 CDCl3
4.81
4.80
4.80
4.79
4.78
3.17
2.37
1.78
1.75
1.74
1.73
1.59
1.59
1.58
1.57
1.56
1.55
1.54
1.53
1.52
1.50
1.47
1.45
1.44
1.43
1.42
1.39
1.38
1.36
1.35
1.35
1.25
2.00
2.98
2.04
2.16
8.19
9.80
1H (ppm)

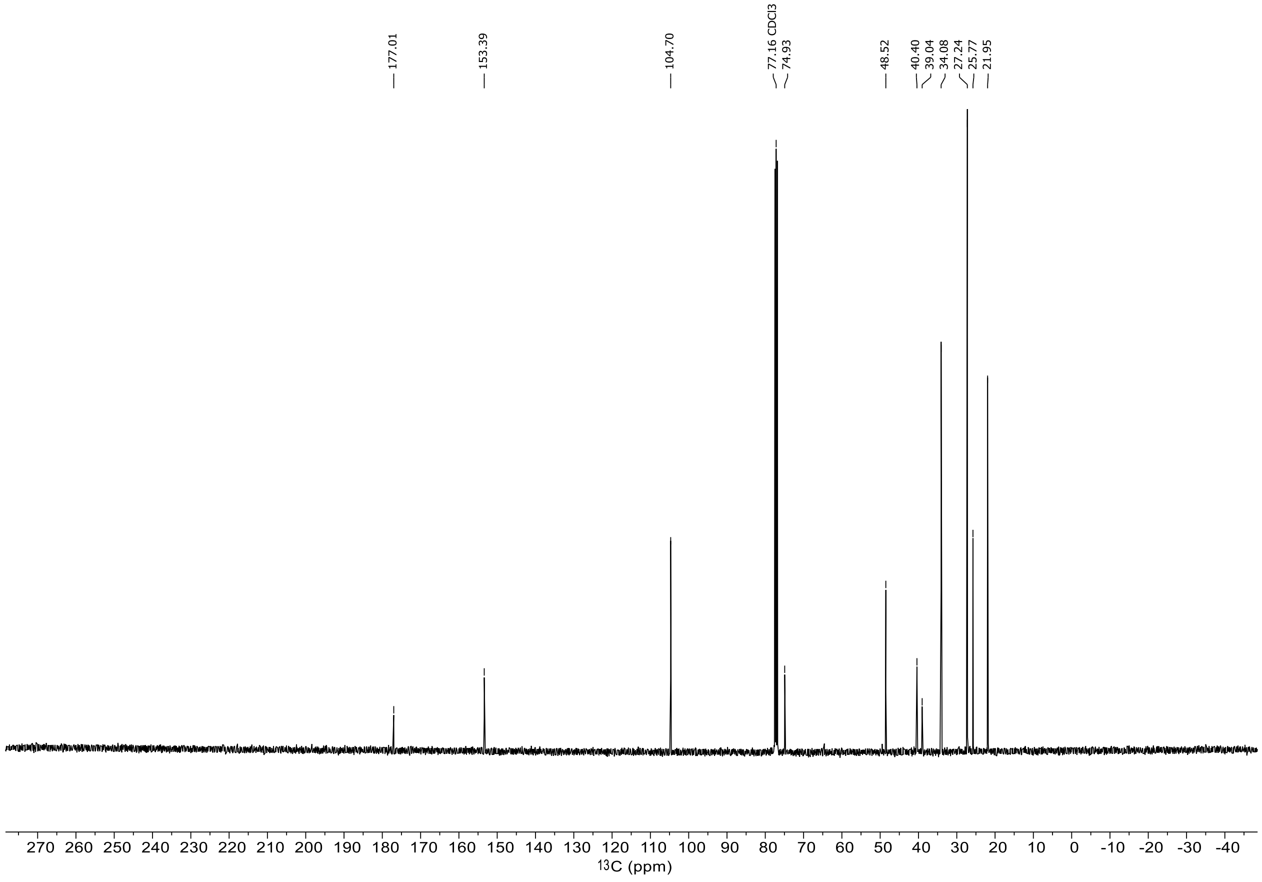

177.01
153.39
104.70
77.16 CDCl3
74.93
48.52
40.40
39.04
34.08
27.24
25.77
21.95
13C (ppm)

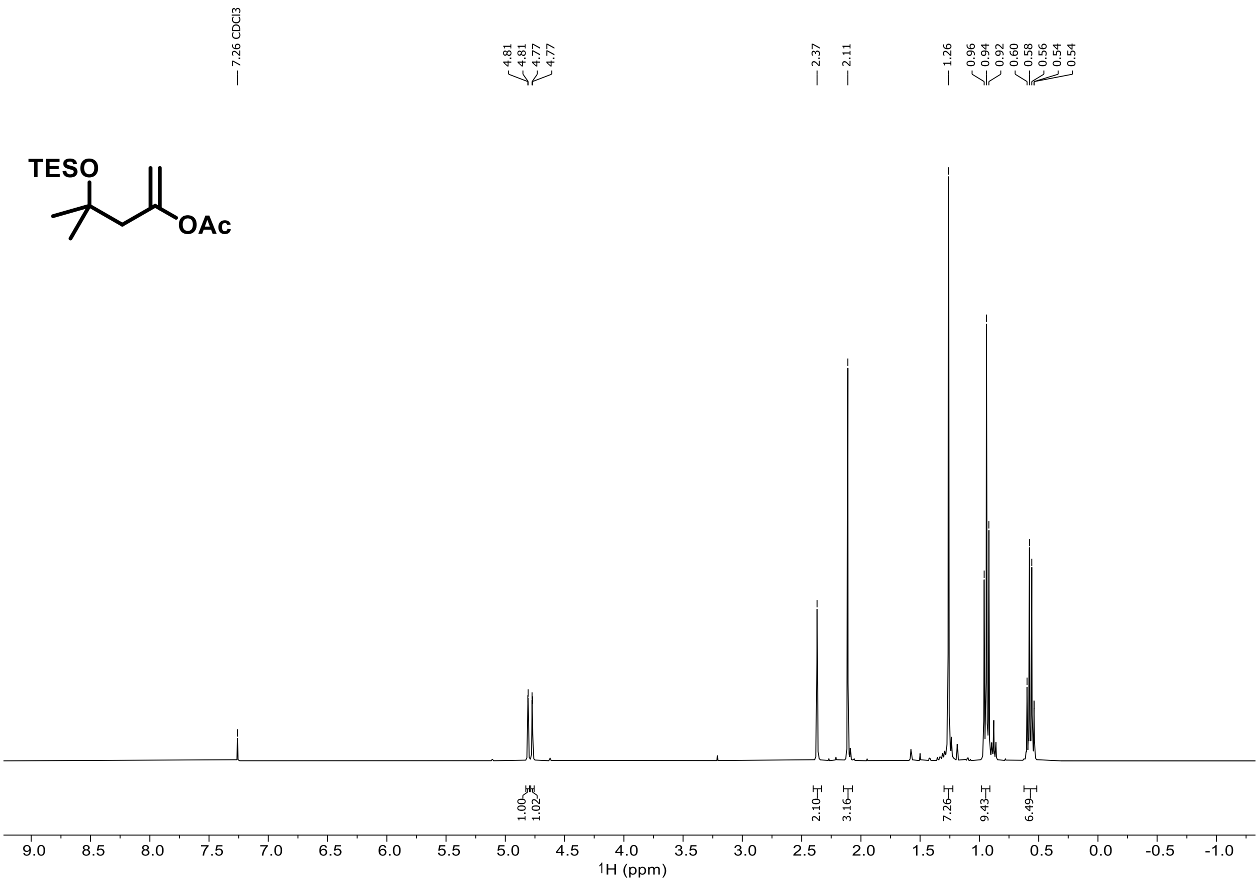
TESO
OAc
7.26 CDCl3
4.81
4.81
4.77
4.77
2.37
2.11
1.26
0.96
0.94
0.92
0.60
0.58
0.56
0.54
0.54
1.00
1.02
2.10
3.16
7.26
9.43
6.49
1H (ppm)

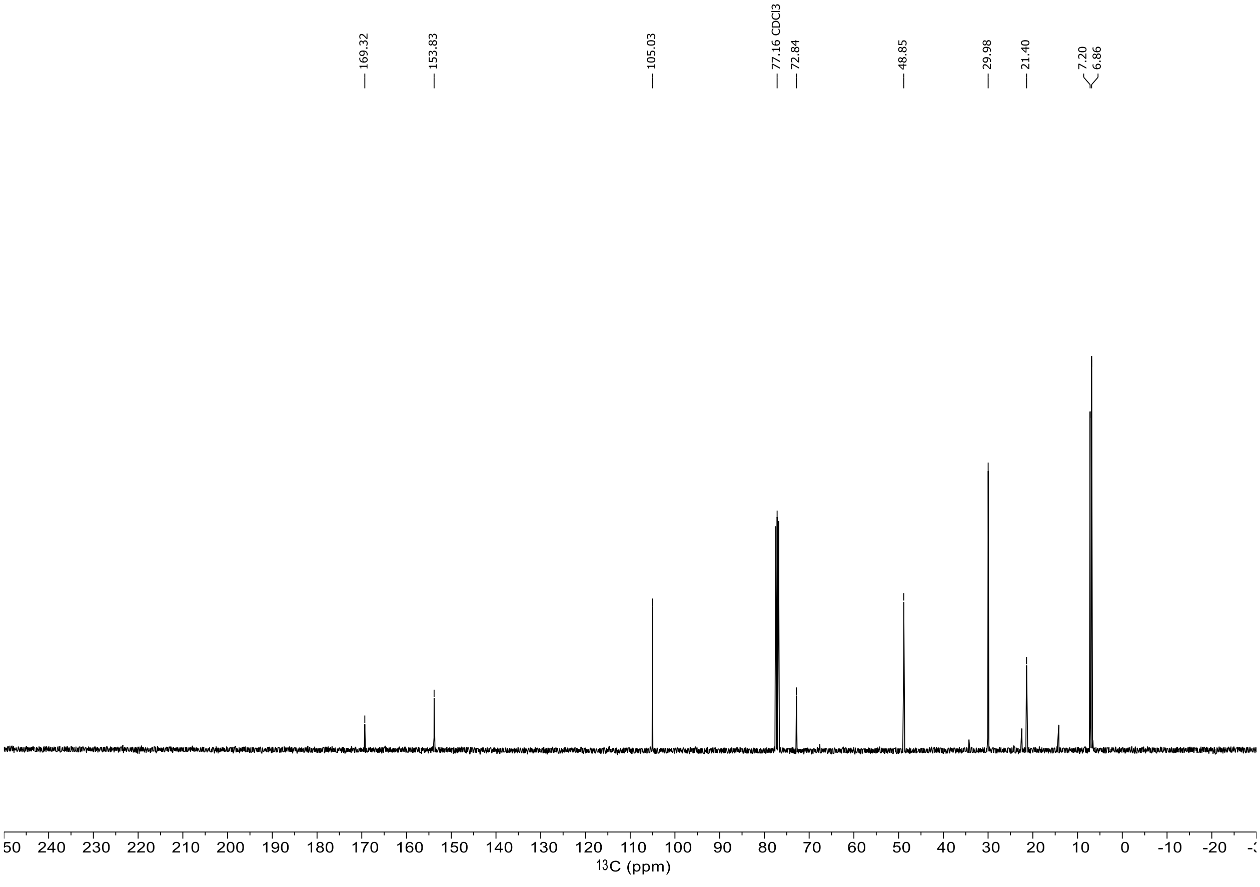
169.32
153.83
105.03
77.16 CDCl3
72.84
48.85
29.98
21.40
7.20
6.86
13C (ppm)

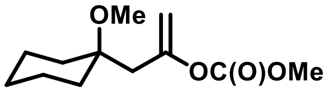

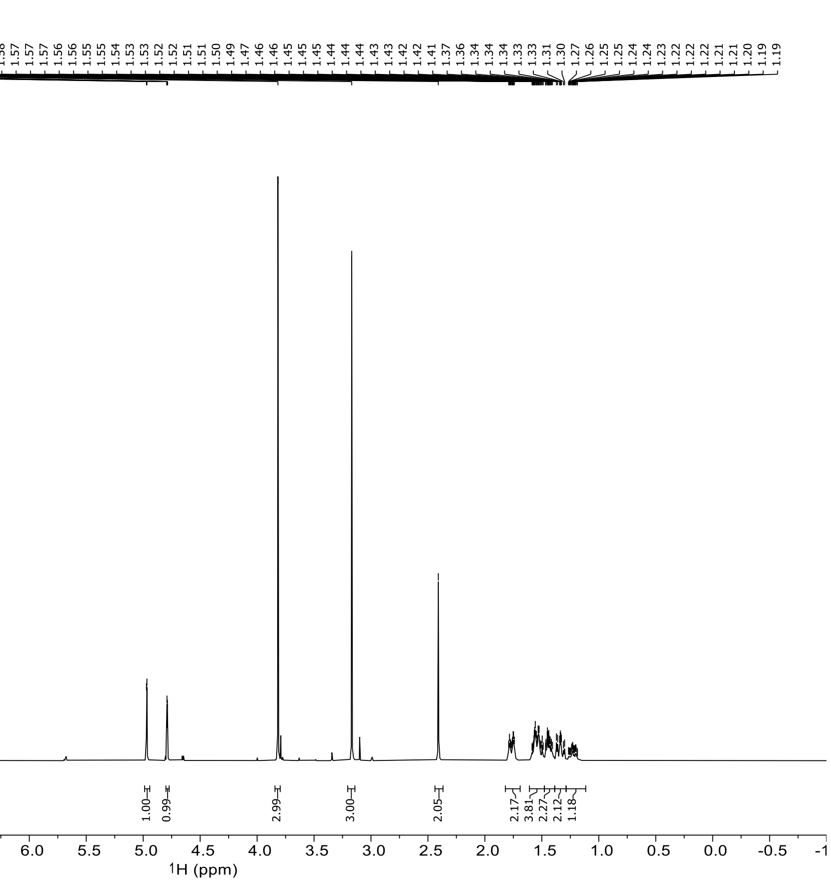
1H (ppm)

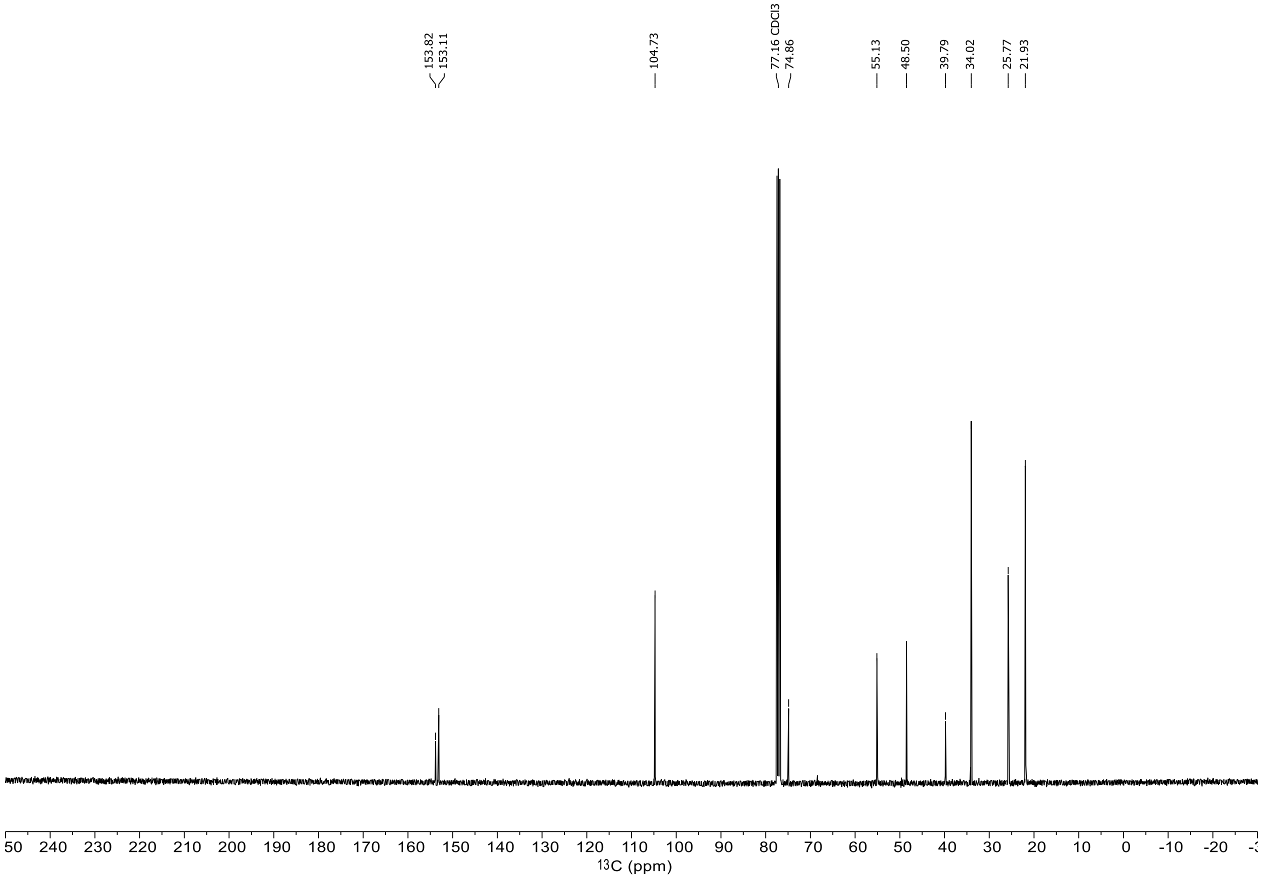
13C (ppm)

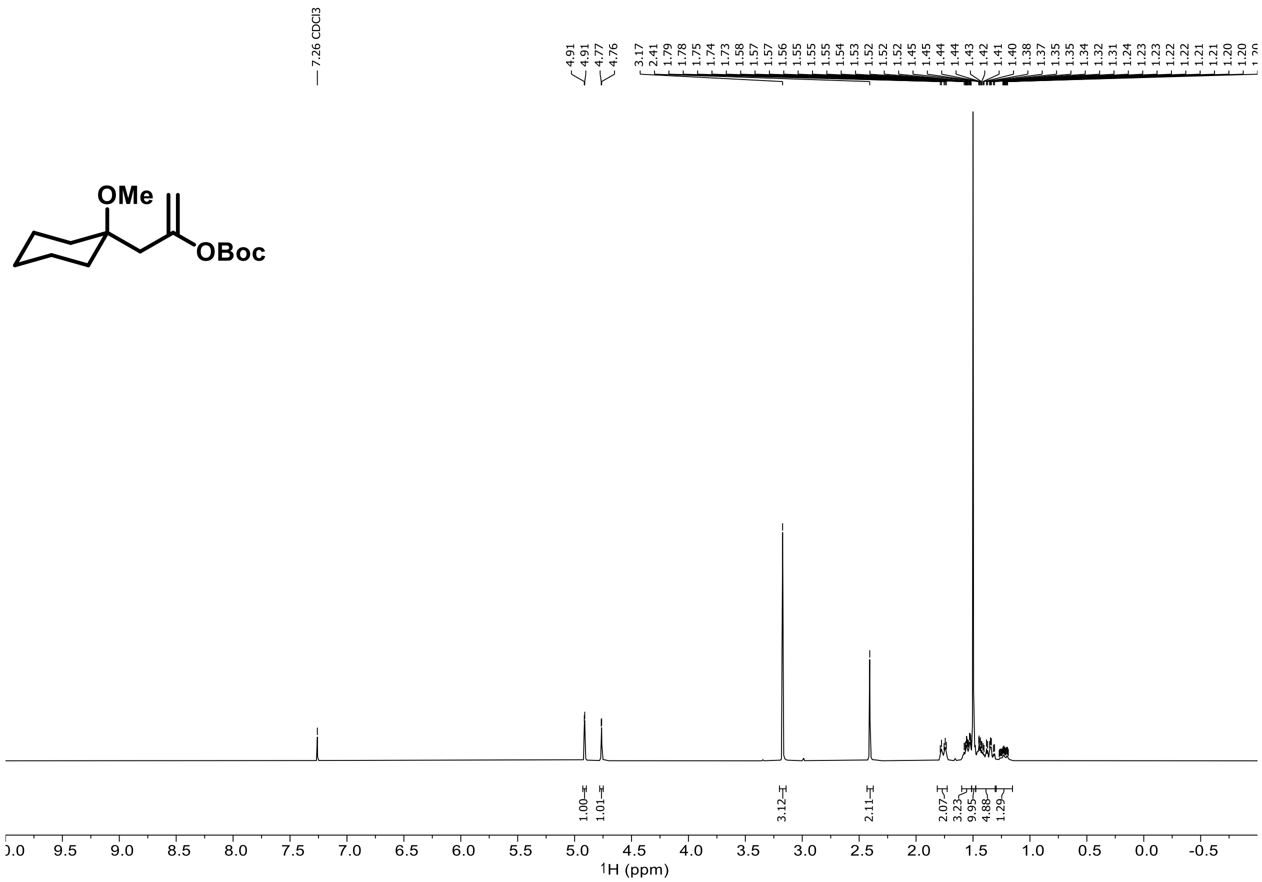
7.26 CDCl3
4.91
4.91
4.77
4.76
3.17
2.41
1.00
1.01
3.12
2.11
2.07
3.23
9.95
4.88
1.29
1H (ppm)

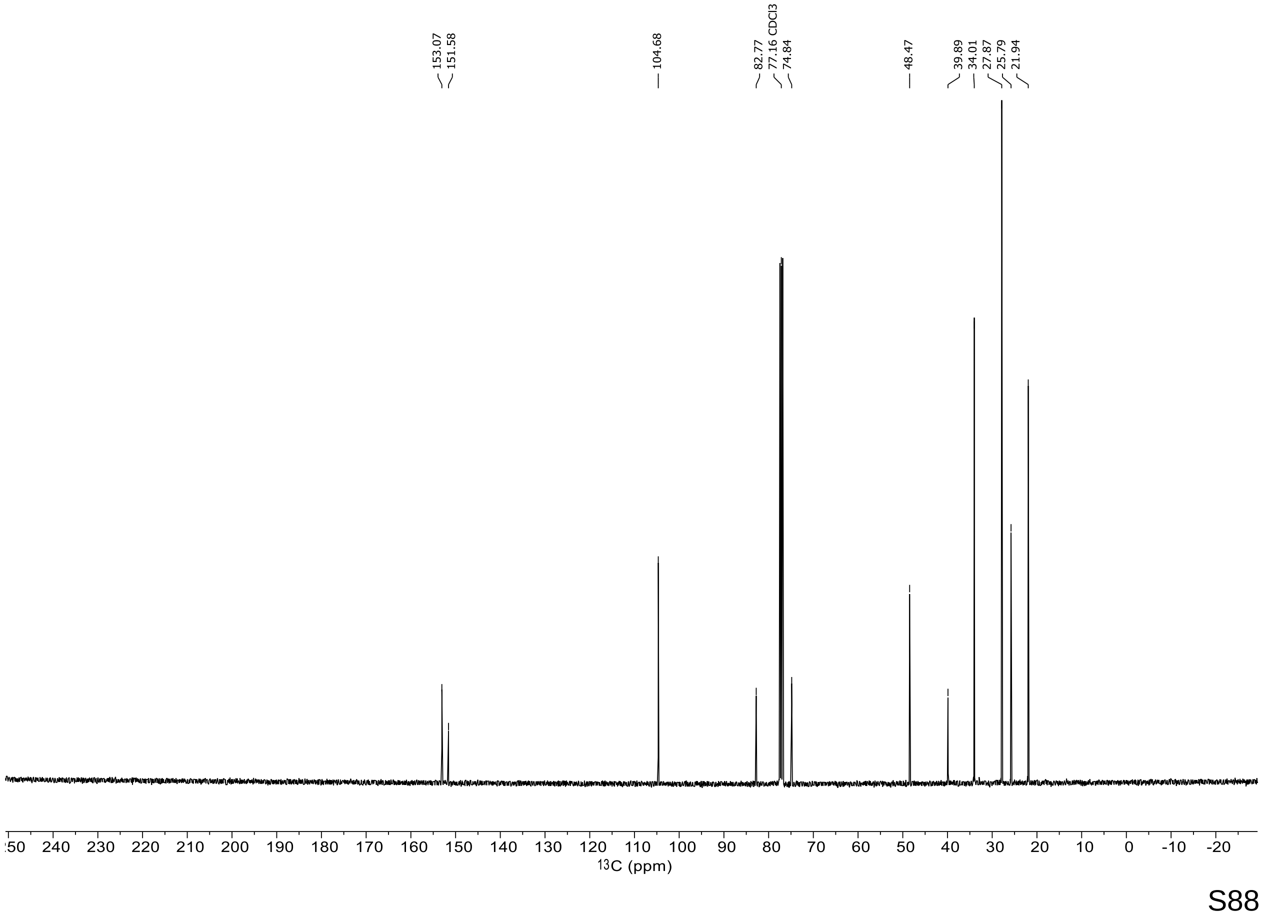
153.07
151.58
104.68
82.77
77.16 CDCl3
74.84
48.47
39.89
34.01
27.87
25.79
21.94
13C (ppm)

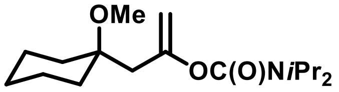

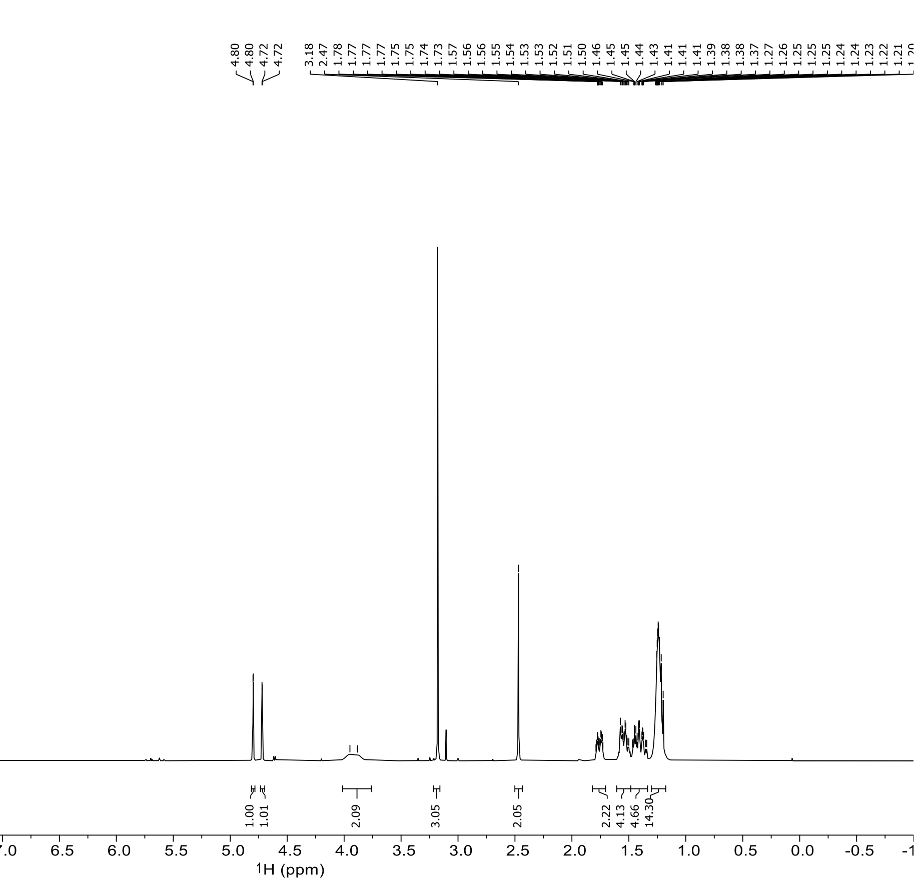

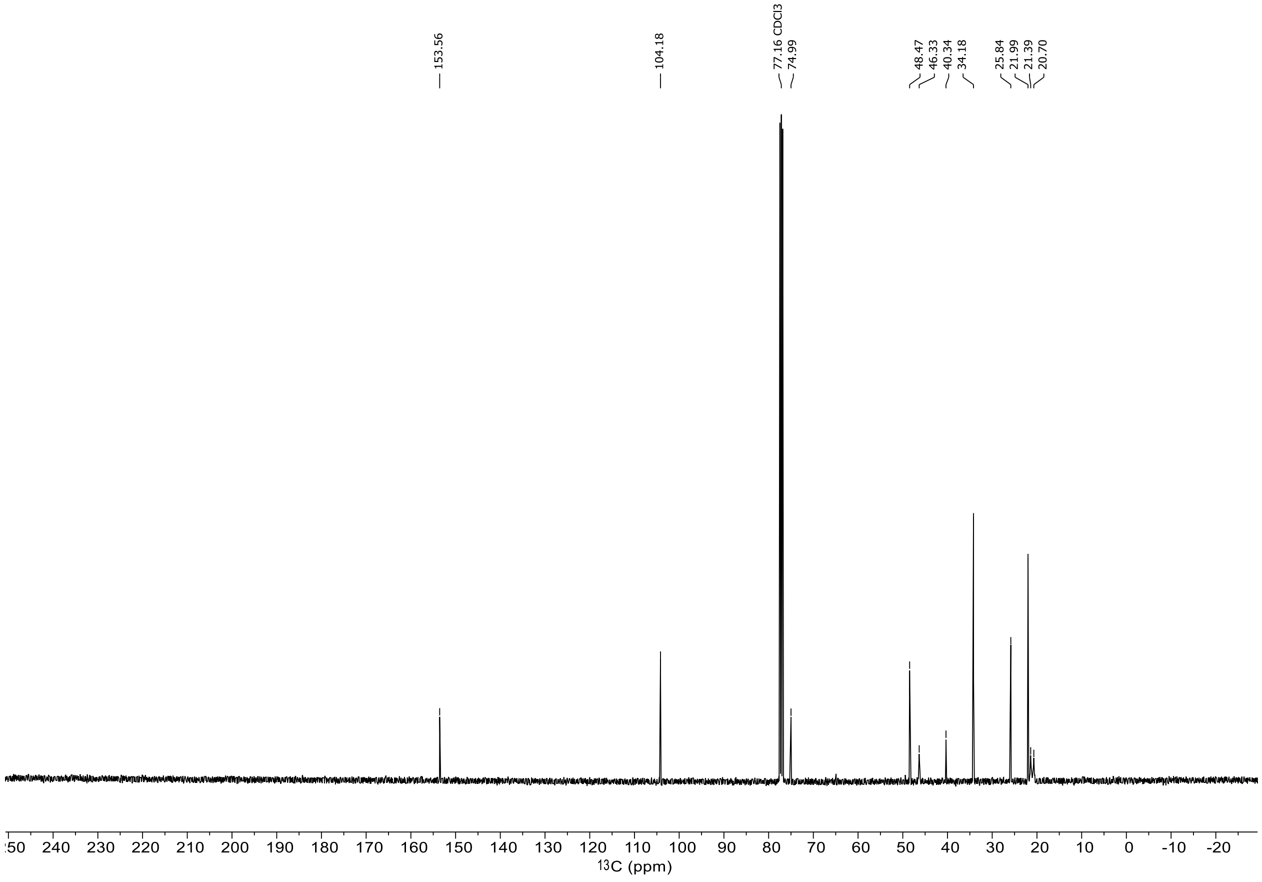

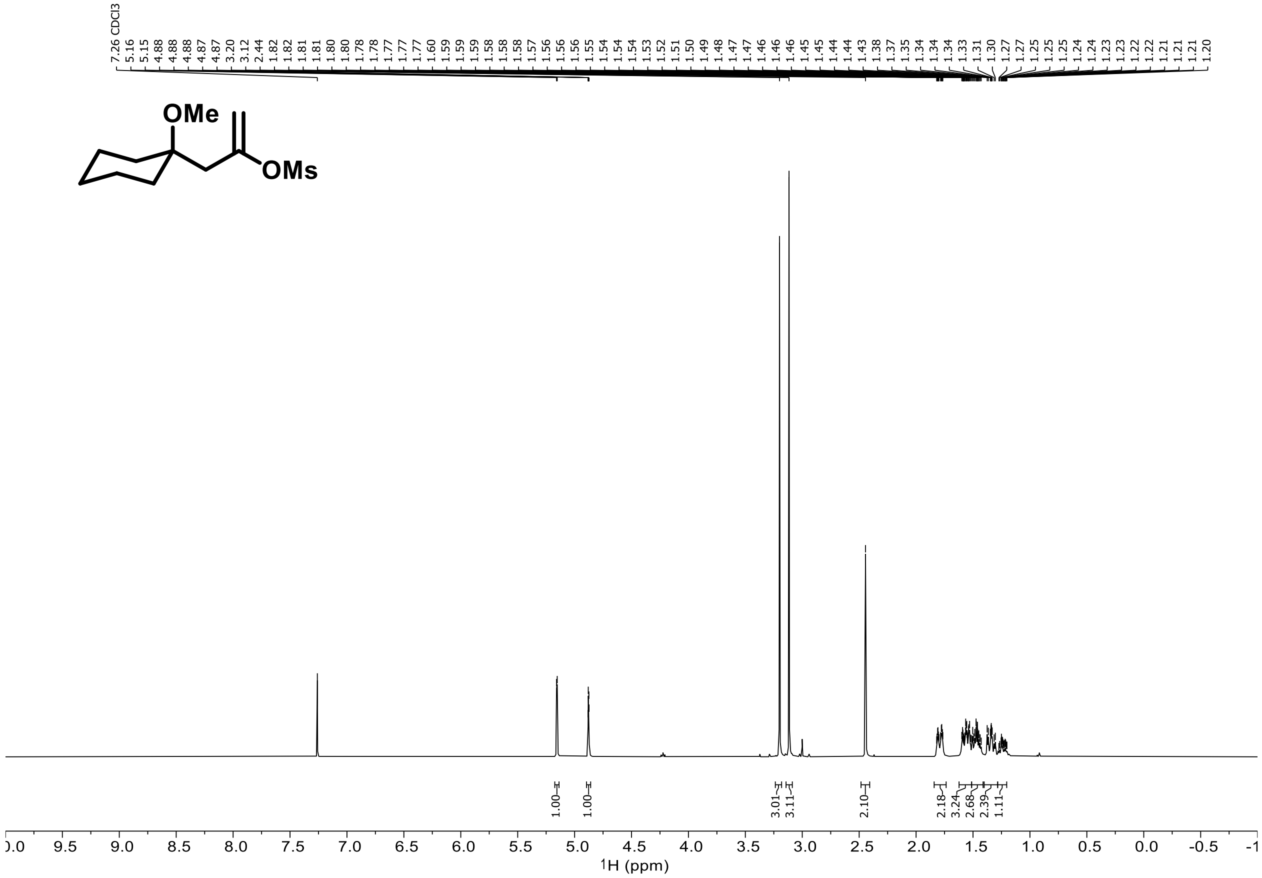
OMe
OMs
1H (ppm)

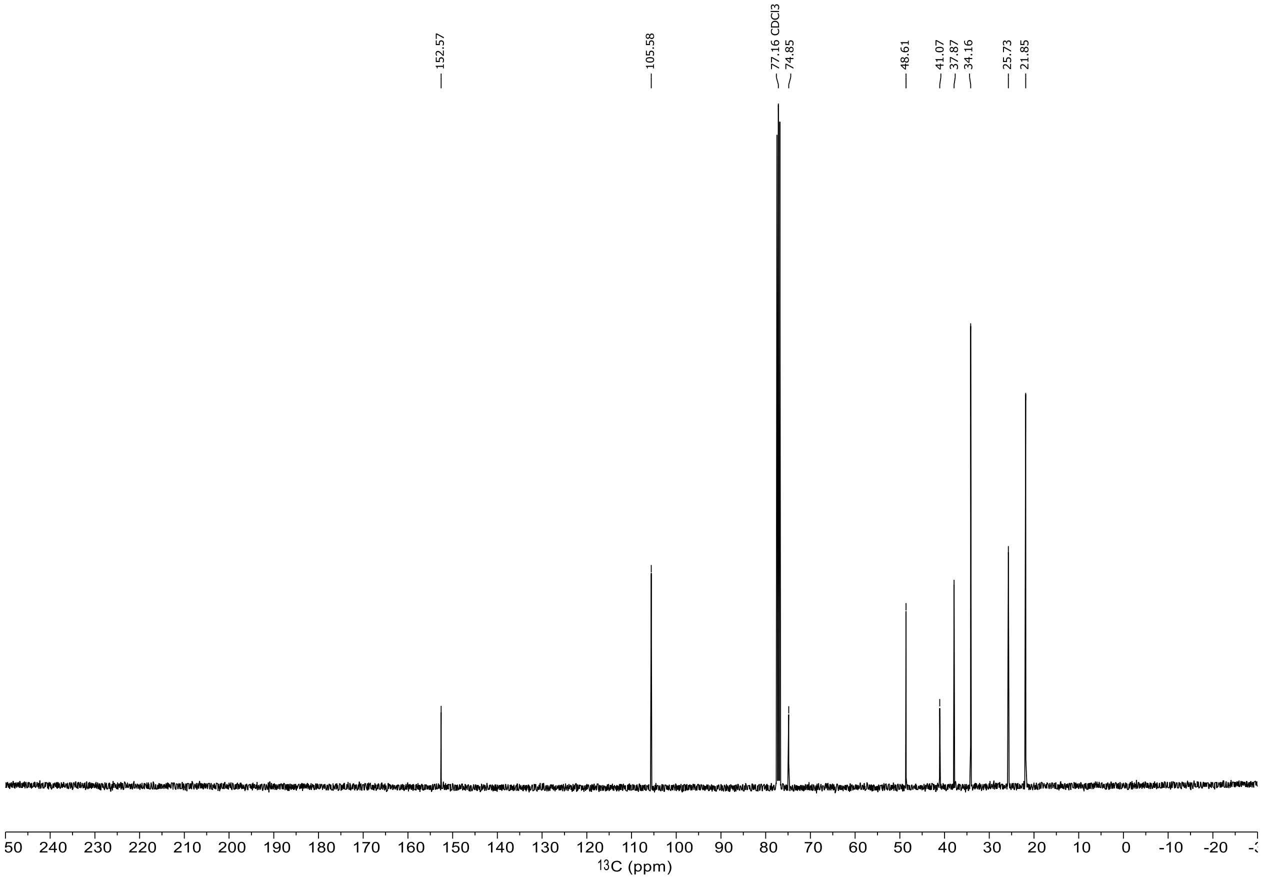
152.57
105.58
77.16 CDCl3
74.85
48.61
41.07
37.87
34.16
25.73
21.85
13C (ppm)

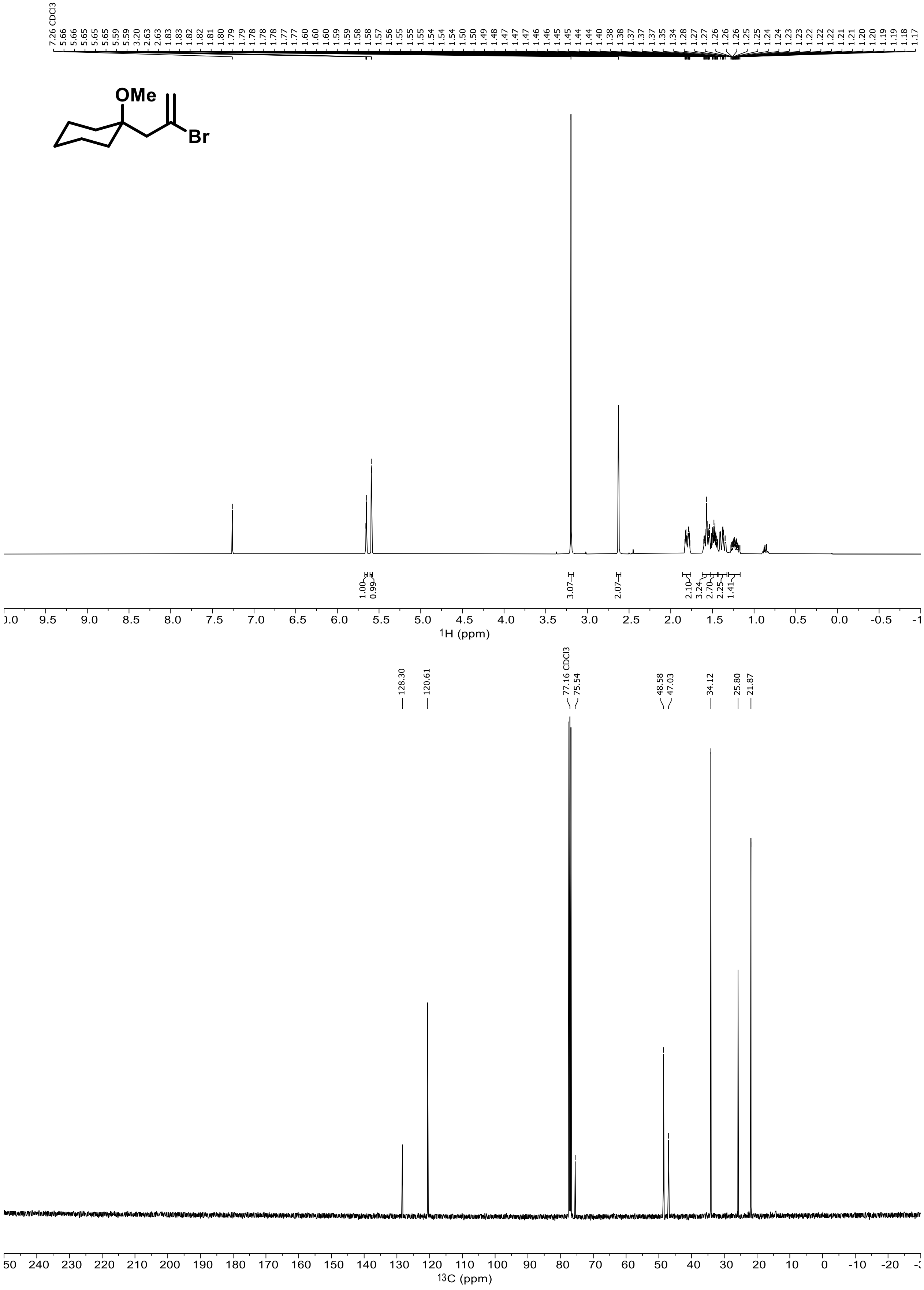
OMe
Br
1H (ppm)
13C (ppm)
7.26 CDCl3
128.30
120.61
77.16 CDCl3
75.54
48.58
47.03
34.12
25.80
21.87

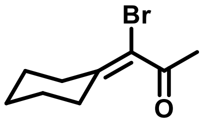

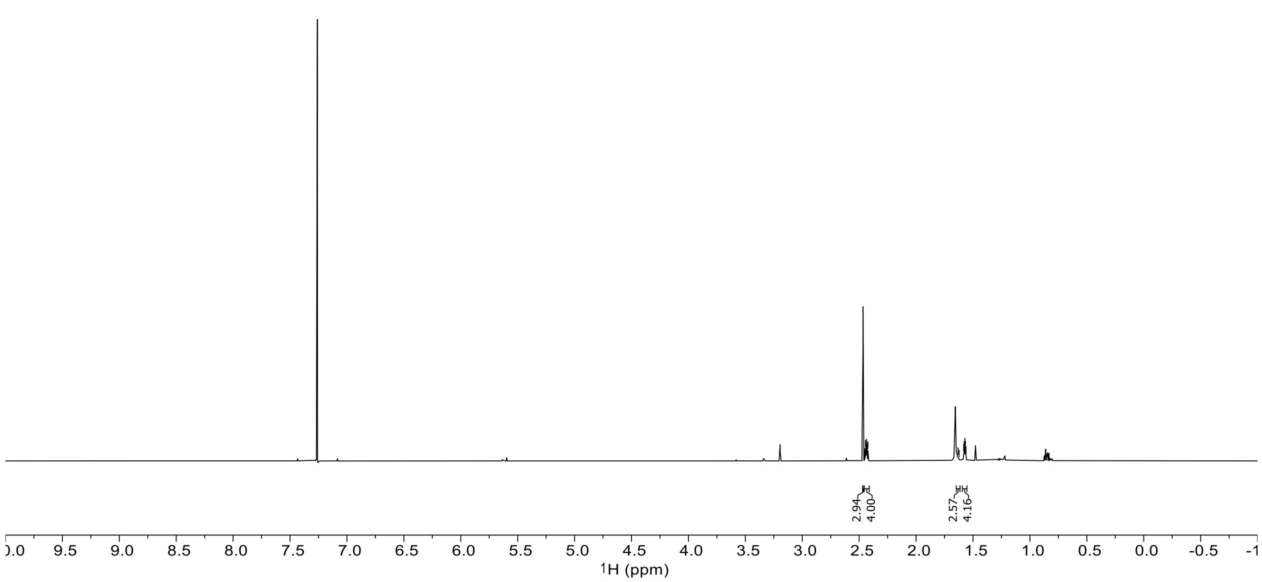

2.45 2.45 2.45 2.44 2.44 2.44 2.43 2.43 2.43 2.42 1.63 1.62 1.58 1.58 1.57 1.57 1.56
2.94 4.00 2.57 4.16
1H (ppm)

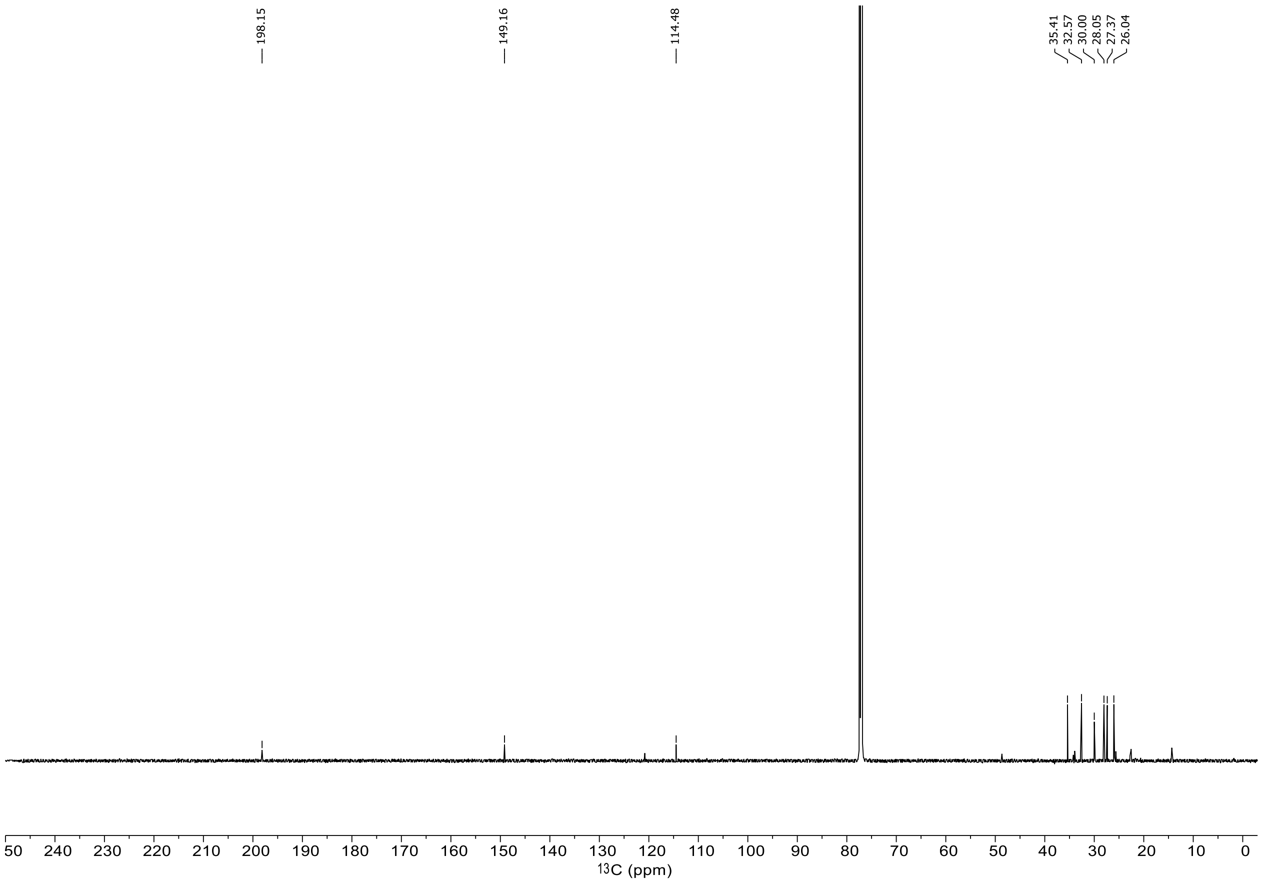

198.15
149.16
114.48
35.41 32.57 30.00 28.05 27.37 26.04
13C (ppm)

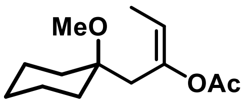
MeO
OAc

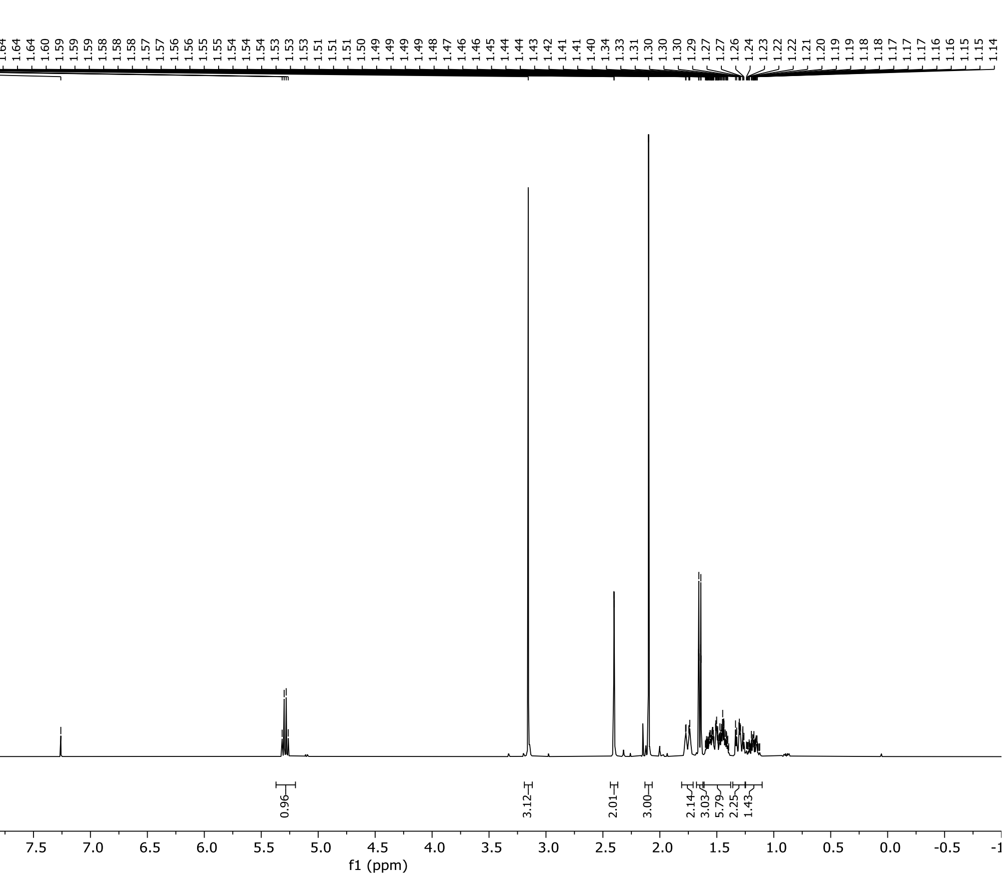
7.26 CDCl3
5.32
5.30
5.28
5.26
3.16
2.40
2.40
2.10
1.77
1.77
1.74
1.74
1.74
1.66
1.66
1.64
1.64
1.64
1.60
1.59
1.59
1.59
1.58
1.58
1.58
1.57
1.57
1.56
1.56
1.55
1.55
1.54
1.54
1.54
1.53
1.53
1.53
1.51
1.51
1.51
1.50
1.49
1.49
1.49
1.49
1.48
1.47
1.46
1.46
1.45
1.44
1.44
1.43
1.42
1.41
1.41
1.40
1.34
1.33
1.31
1.30
1.30
1.29
1.27
1.27
1.26
1.24
1.23
1.22
1.22
1.21
1.20
1.19
1.19
1.18
1.17
1.17
1.17
1.16
1.15
1.14
0.96
3.12
2.01
3.00
2.14
3.03
5.79
2.25
1.43
9.5 9.0 8.5 8.0 7.5 7.0 6.5 6.0 5.5 5.0 4.5 4.0 3.5 3.0 2.5 2.0 1.5 1.0 0.5 0.0 -0.5
f1 (ppm)

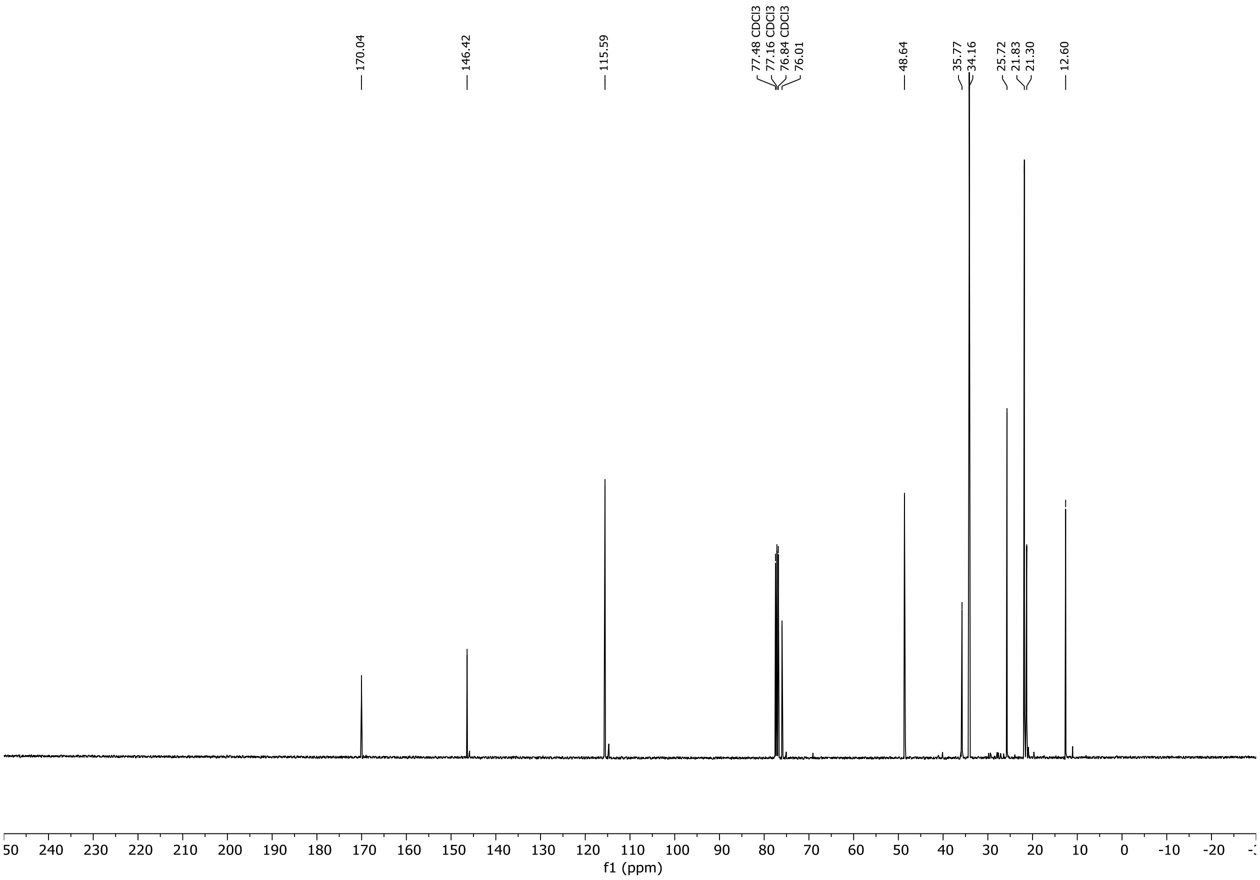
170.04
146.42
115.59
77.48 CDCl3
77.16 CDCl3
76.84 CDCl3
76.01
48.64
35.77
34.16
25.72
21.83
21.30
12.60
240 230 220 210 200 190 180 170 160 150 140 130 120 110 100 90 80 70 60 50 40 30 20 10 0 -10 -20
f1 (ppm)

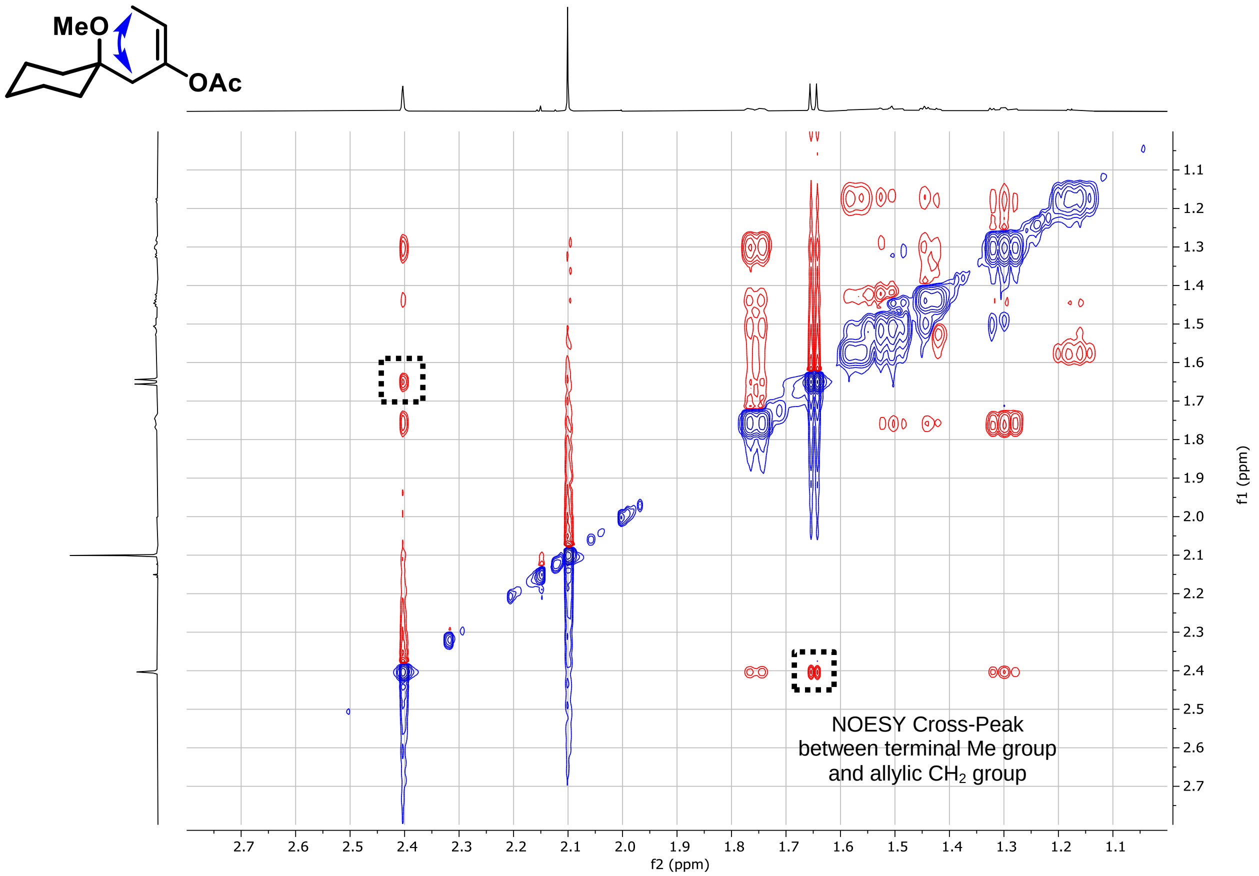
MeO
OAc
NOESY Cross-Peak
between terminal Me group
and allylic CH_2 group
f2 (ppm)
f1 (ppm)

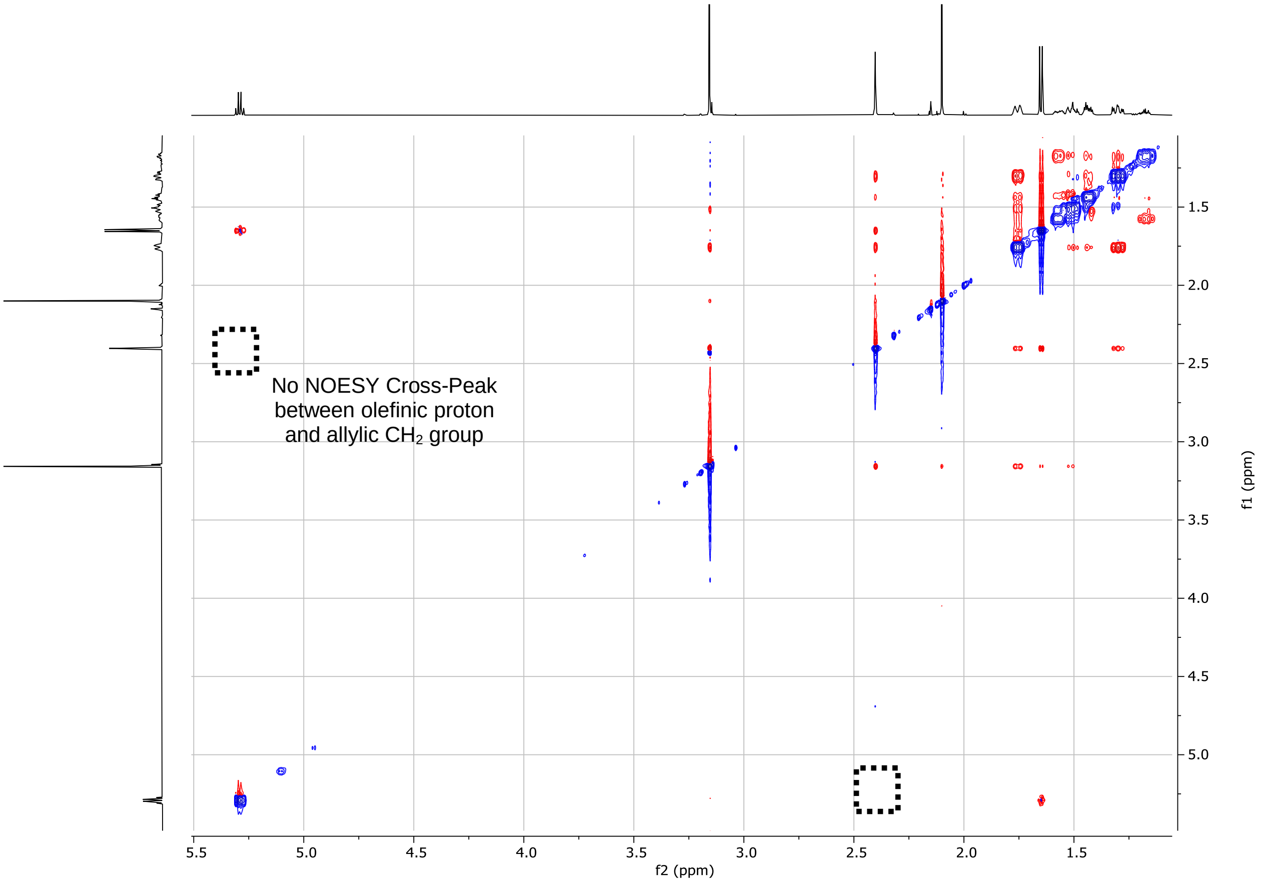
No NOESY Cross-Peak
between olefinic proton
and allylic CH_2 group
f2 (ppm)
f1 (ppm)

References

1. Aguilar, J. A.; Elliott, P. I. P.; López-Serrano, J.; Adams, R. W.; Duckett, S. B., Only para-hydrogen spectroscopy (OPSY), a technique for the selective observation of para-hydrogen enhanced NMR signals. *Chem. Commun.* **2007,** (11), 1183-1185.
2. Fagan, P. J.; Ward, M. D.; Calabrese, J. C., Molecular engineering of solid-state materials: organometallic building blocks. *J. Am. Chem. Soc.* **1989,** *111* (5), 1698-1719.
3. Fagan, P. J.; Mahoney, W. S.; Calabrese, J. C.; Williams, I. D., Structure and chemistry of the complex tetrakis(η^5-pentamethylcyclopentadienyl)tetrakis(μ^3-chloro)tetraruthenium(II): a useful precursor to (pentamethylcyclopentadienyl)ruthenium(0), -(II), and -(IV) complexes. *Organometallics* **1990,** *9* (6), 1843-1852.
4. Mbaye, M. D.; Demerseman, B.; Renaud, J.-L.; Toupet, L.; Bruneau, C., Ruthenium-Catalyzed O-Allylation of Phenols from Allylic Chlorides via Cationic [Cp*(η^3-allyl)(MeCN)RuX][PF_6] Complexes. *Adv. Syn. Cat.* **2004,** *346* (7), 835-841.
5. Kündig, E. P.; Monnier, F. R., Efficient Synthesis of Tris(acetonitrile)-(η^5-cyclopentadienyl)-ruthenium(II) Hexafluorophosphate via Ruthenocene. *Adv. Syn. Cat.* **2004,** *346* (8), 901-904.
6. Matsushima, Y.; Komatsuzaki, N.; Ajioka, Y.; Yamamoto, M.; Kikuchi, H.; Takata, Y.; Dodo, N.; Onitsuka, K.; Uno, M.; Takahashi, S., Synthesis and Properties of Planar-Chiral (η^6-Benzene)(η^5-cyclopentadienyl)ruthenium(II) Complexes in an Optically Pure Form. *Bull. Chem. Soc. Jpn.* **2001,** *74* (3), 527-537.
7. Biberger, T.; Gordon, C. P.; Leutzsch, M.; Peil, S.; Guthertz, A.; Copéret, C.; Fürstner, A., Alkyne *gem*-Hydrogenation: Formation of Pianostool Ruthenium Carbene Complexes and Analysis of Their Chemical Character. *Angew. Chem. Int. Ed.* **2019,** *58*, 8845-8850.
8. Biberger, T.; Zachmann, R. J.; Fürstner, A., Grubbs Metathesis Enabled by a Light-Driven *gem*-Hydrogenation of Internal Alkynes. *Angew. Chem. Int. Ed.* **2020,** *59* (42), 18423-18429.
9. Peil, S.; Fürstner, A., Mechanistic Divergence in the Hydrogenative Synthesis of Furans and Butenolides: Ruthenium Carbenes Formed by *gem*-Hydrogenation or through Carbophilic Activation of Alkynes. *Angew. Chem. Int. Ed.* **2019,** *58* (51), 18476-18481.
10. Wagner, C. E.; Shea, K. J., Synthesis and First Molecular Structure of a Bis-2-spiro-1-boraadamantane Derivative. *Org. Lett.* **2004,** *6* (3), 313-316.
11. Imada, Y.; Nishida, M.; Kutsuwa, K.; Murahashi, S.-I.; Naota, T., Palladium-Catalyzed Asymmetric Amination and Imidation of 2,3-Allenyl Phosphates. *Org. Lett.* **2005,** *7* (26), 5837-5839.
12. Peil, S.; Guthertz, A.; Biberger, T.; Fürstner, A., Hydrogenative Cyclopropanation and Hydrogenative Metathesis. *Angew. Chem. Int. Ed.* **2019,** *58* (26), 8851-8856.
13. Bolte, B.; Odabachian, Y.; Gagosz, F., Gold(I)-catalyzed rearrangement of propargyl benzyl ethers: a practical method for the generation and in situ transformation of substituted allenes. *J. Am. Chem. Soc.* **2010,** *132* (21), 7294-6.
14. Spencer, J. A.; Jamieson, C.; Talbot, E. P. A., One-Pot, Three-Step Synthesis of Cyclopropylboronic Acid Pinacol Esters from Synthetically Tractable Propargylic Silyl Ethers. *Org. Lett.* **2017,** *19* (14), 3891-3894.
15. Kusakabe, T.; Kawai, Y.; Kato, K., Total Synthesis of (+)-Gregatin B and E. *Org. Lett.* **2013,** *15* (19), 5102-5105.
16. Mukai, C.; Itoh, R., Grubbs catalyst-mediated cycloisomerization of allenenes. *Tetrahedron Lett.* **2006,** *47* (24), 3971-3974.
17. Groß, T.; Metz, P., An Efficient Gold-Catalyzed Domino Process for the Construction of Tetracyclic Ketoethers. *Chem. Eur. J.* **2013,** *19* (44), 14787-14790.
18. Gao, F.; Hoveyda, A. H., α-Selective Ni-Catalyzed Hydroalumination of Aryl- and Alkyl-Substituted Terminal Alkynes: Practical Syntheses of Internal Vinyl Aluminums, Halides, or Boronates. *J. Am. Chem. Soc.* **2010,** *132* (32), 10961-10963.
